# Novel Insights on Obligate Symbiont Lifestyle and Adaptation to Chemosynthetic Environment as Revealed by the Giant Tubeworm Genome

**DOI:** 10.1093/molbev/msab347

**Published:** 2021-12-06

**Authors:** André Luiz de Oliveira, Jessica Mitchell, Peter Girguis, Monika Bright

**Affiliations:** 1 Department of Functional and Evolutionary Ecology, University of Vienna, Vienna, Austria; 2 Department of Organismic and Evolutionary Biology, Harvard University, Cambridge, MA, USA

**Keywords:** vestimentiferan, host–symbiont interaction, organismal evolution, comparative genomics/transcriptomics

## Abstract

The mutualism between the giant tubeworm *Riftia pachyptila* and its endosymbiont *Candidatus* Endoriftia persephone has been extensively researched over the past 40 years. However, the lack of the host whole-genome information has impeded the full comprehension of the genotype/phenotype interface in *Riftia*. Here, we described the high-quality draft genome of *Riftia*, its complete mitogenome, and tissue-specific transcriptomic data. The *Riftia* genome presents signs of reductive evolution, with gene family contractions exceeding expansions. Expanded gene families are related to sulfur metabolism, detoxification, antioxidative stress, oxygen transport, immune system, and lysosomal digestion, reflecting evolutionary adaptations to the vent environment and endosymbiosis. Despite the derived body plan, the developmental gene repertoire in the gutless tubeworm is extremely conserved with the presence of a near intact and complete Hox cluster. Gene expression analyses establish that the trophosome is a multifunctional organ marked by intracellular digestion of endosymbionts, storage of excretory products, and hematopoietic functions. Overall, the plume and gonad tissues both in contact to the environment harbor highly expressed genes involved with cell cycle, programed cell death, and immunity indicating a high cell turnover and defense mechanisms against pathogens. We posit that the innate immune system plays a more prominent role into the establishment of the symbiosis during the infection in the larval stage, rather than maintaining the symbiostasis in the trophosome. This genome bridges four decades of physiological research in *Riftia*, whereas it simultaneously provides new insights into the development, whole organism functions, and evolution in the giant tubeworm.

## Introduction

The discovery of the giant tubeworm *Riftia pachyptila* (Jones 1981) at deep-sea hydrothermal vents on the Galapagos Spreading center in 1977 (Corliss et al. 1979) has initiated the onset of a continuous torrent of studies (Childress and Fisher 1992; Nelson and Fisher 1995; Stewart and Cavanaugh 2006; Bright and Lallier 2010; Childress and Girguis 2011; Hilário et al. 2011). With its enormous size (Fisher et al. 1988; Hessler et al. 1988; Shank et al. 1998), rapid cell proliferation (Pflugfelder et al. 2009), seemingly fast growth (Lutz et al. 1994, 2001), but short life (Klose et al. 2015) one of the most puzzling findings was the lack of a digestive system in an animal with a highly unusual body plan (Jones 1981). Descriptions of mouth- and gutless pogonophoran relatives go back a century (Caullery 1914). The first vestimentiferans *Lamellibrachia barhami* Webb, 1969 and *Lamellibrachia luymesi* van der Land and Nørrevang, 1975 were described already a few years earlier than *Riftia*. However, it was the discovery of *Riftia*, thriving in an apparently poisonous hydrothermal vent environment, which sparked the discovery of the first-described chemosynthetic animal–microbe symbiosis (Cavanaugh et al. 1981); an association in which *Riftia*, without a mouth or a gut, relies on the sulfide oxidizing chemoautotrophic symbionts for nutrition (Cavanaugh et al. 1981; Felbeck 1981; Arp and Childress 1981, 1983; Rau 1981a, 1981b).

Despite the fact that neither the animal host, nor the symbiont, nor the intact association are amenable to long-term cultivation, *Riftia* is easily one of the best studied deep-sea animals which have consistently led to major discoveries (reviewed by Bright and Lallier [2010]). Crucial was the development of various devices to measure chemical and physical parameters directly in the deep sea to understand the abiotic conditions under which this tubeworm thrives at vigorous diffuse vent flow (Hessler et al. 1988; Shank et al. 1998; Luther et al. 2001; Le Bris et al. 2003; Mullineaux et al. 2003; Le Bris, Govenar, et al. 2006; Le Bris, Rodier, et al. 2006). Unprecedented and equally important was the development of high-pressure flow-through systems to simulate in situ conditions in the lab (Quetin and Childress 1980; Girguis et al. 2000).There has been probably no deep-sea animal with more resourceful experimental approaches applied in situ and ex situ than *Riftia*, for example, catheterized tubeworms under flow-through pressure (Felbeck and Turner 1995), artificial insemination and developmental studies under pressure (Marsh et al. 2001), predation experiments with mesh cages in situ (Micheli et al. 2002), hydraulically actuated collection devices of tubeworm aggregations (Hunt et al. 2004; Govenar et al. 2005), artificial plastic tube deployments (Govenar and Fisher 2007), pressurized experiments (Goffredi et al. 1997; Shillito et al. 1999; Girguis et al. 2000, 2002), and finally, various in situ settlement devices for tubeworm larvae (Mullineaux et al. 2000, 2020; Nussbaumer et al. 2006). These innovative experiments associated with four decades of research taught us about many aspects of *Riftia*’s evolution and biology.

**Fig. 1.**
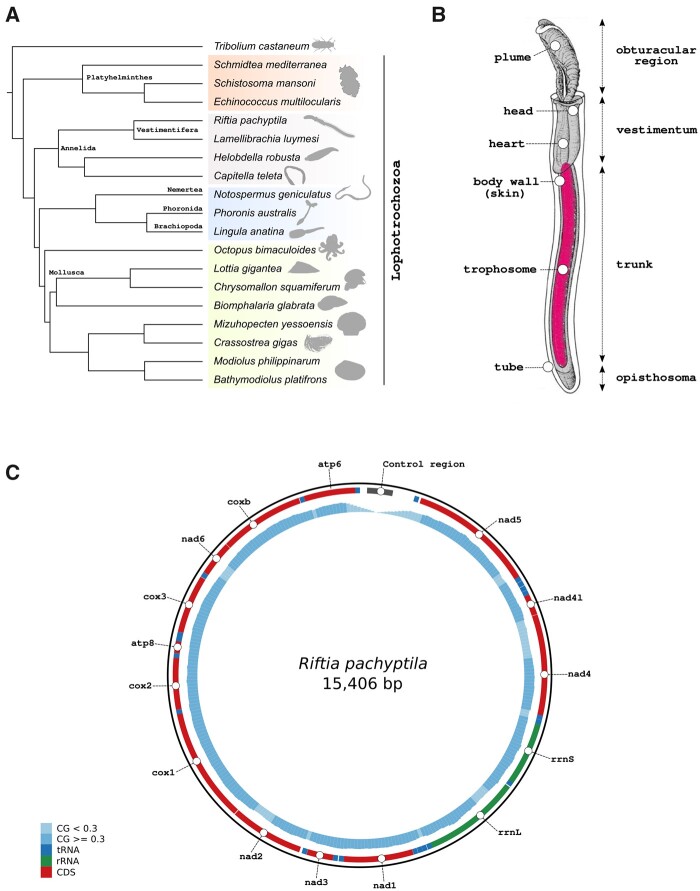
Overview of *Riftia pachyptila* body plan anatomy, phylogenetic placement, and mitochondrial genome. (*A*) Phylogenetic placement of *Riftia pachyptila* within Lophotrochozoa. *Riftia* together with *Lamellibrachia* form the clade Vestimentifera, a group of marine animals living in chitinous tubes and lacking a digestive tract. Animal silhouettes were downloaded from http://phylopic.org/. Tree topology was obtained through phylogenomic analysis. (*B*) Schematic drawing of *Riftia pachyptila*. The first part of the body, the obturacular region, contains the highly vascularized plume, whereas the head, heart, and gonads are located in the second body part, the vestimentum. The trunk region, third body part, harbors the trophosome (organ that houses the symbiotic bacteria), and the body wall (skin). The posterior part, the opisthosoma is the fourth and last body region of the tubeworm. Schematic drawing was modified from Nussbaumer et al. (2006). (*C*) Schematic representation of *Riftia pachyptila* mitochondrial genome, including the complete control region. CG-content and tRNA genes are represented by the blue histograms and boxes, respectively.

After many microanatomical studies accompanied by heated, highly controversial phylogenetic discussions, the question of who the closest relatives of *Riftia* are was ultimately solved by traditional cladistic and novel molecular analyses (Fauchald and Rouse 1997; McHugh 1997; Halanych et al. 2001; Rouse 2001; Schulze 2002). They showed that vestimentiferans are lophotrochozoan polychaetae worms within Annelida (fig. 1*A*) (Polychaeta, Siboglinidae, Vestimentifera) (Pleijel et al. 2009). Similar to many other polychaetes, *Riftia* is gonochoristic with internal fertilization and undergoes a biphasic life cycle with a pelagic phase including indirect development through spiral cleavage and a trochophore larvae (Marsh et al. 2001). The benthic phase is marked by the uptake of the symbiont into the metatrochophore larvae and growth into an adult, which completely reduces its mouth, gut, and anus. Instead, a unique mesodermal nutritional organ, the trophosome, functionally replaces the digestive system (Nussbaumer et al. 2006; Bright et al. 2013). The adult body is organized into four distinct regions, the obturacular region, the vestimentum, the trunk, and the opisthosoma (fig. 1*B*). The anterior obturacular region of the animal projects a vascularized branchial plume, which is responsible for the sequestration of nutrients and gas exchange, followed by the vestimentum, a muscular head region enclosing the heart, brain, the excretory organ, and the gonopores. The trunk region, the single elongated first segment, harbors the trophosome and the gonads. The posterior part, the opisthosoma, contains a typical segmented annelid region with serially arranged chaetae (Bright et al. 2013). It is so far unknown how this unusual body plan lacking the entire digestive system is reflected in their developmental genes and signaling pathways. Gutless parasitic tapeworms, for example, have lost many developmental genes including all ParaHox genes (Tsai et al. 2013).

The trophosome of *Riftia*, a soft multilobed and highly vascular tissue, houses a polyclonal endosymbiotic population dominated by one genotype of *Candidatus* Endoriftia persephone, a chemoautotrophic gammaproteobacteria (Robidart et al. 2008; Gardebrecht et al. 2012; Polzin et al. 2019) that oxidizes sulfur compounds via oxygen and nitrate and, in turn, harnesses that energy to fix dissolved inorganic carbon (or DIC, which includes carbon dioxide and bicarbonate) to organic matter. Briefly, the trophosome is far removed, and has no direct contact with external environment, so the host presumably provides all of the inorganic nutrients to the symbionts. This primarily occurs via the highly vascular brachial plume, which takes up oxygen and hydrogen sulfide (H_2_S) from the external environment and transports these to the trophosome via a complex and unique complement of hemoglobins (Arp and Childress 1981, 1983; Arp et al. 1987; Zal et al. 1996, 1997; Bailly et al. 2002; Flores et al. 2005). DIC is also taken up by *Riftia*, which is unusual as carbon dioxide is an animal respiratory waste product. However, in this case the worm must provide additional DIC to the symbionts for *net* carbon fixation, and does so by accumulating DIC in the blood (Goffredi et al. 1997, 1999). Moreover, physiological studies have shown that *Riftia* also takes up nitrate (also unusual for an animal), and in turn the symbionts reduce it to organic nitrogen (Hentschel and Felbeck 1993; Girguis et al. 2000). In return, the host is nourished through the symbiont releasing organic matter and symbiont digestion, which occurs prior to bacteriocyte death in the periphery of the trophosome lobules (Felbeck 1985; Hand 1987; Felbeck and Jarchow 1998; Bright et al. 2000; Hinzke et al. 2019). Despite four decades of research, key questions about trophosome function remain, including but not limited to: 1) which of the two nutritional modes is more important (organic matter release or symbiont digestion; Bright et al. 2000) and 2) the mechanisms that underlie organic nitrogen synthesis and distribution between the symbionts and the host.

Despite the highly derived annelid body plan, symbiotic lifestyle, and over 40 years of extensive physiological research, whole-genome information of *Riftia* has been lacking. Here, we generated a high-quality genome draft and distinct tissue-specific transcriptomes of the giant gutless tubeworm *Riftia*. By analyzing the genome and transcriptomes of *Riftia* in a comparative framework, we highlight many evolutionary adaptations related to the obligate symbiotic lifestyle and survival in the deep-sea hydrothermal vent environment. The *Riftia* genome, together with a transcriptome and proteome study (Hinzke et al. 2019), a transcriptome study on the close relative *Ridgeia piscesae* (Nyholm et al. 2012), the genomic resources available for other close related tubeworms (*L. luymesi*—short *Lamellibrachia*; *Paraescarpia echinospica*—short *Paraescarpia*) (Li et al. 2019; Sun et al. 2021), and an extensive body of research broadens our understanding of one of the most conspicuous models for host–symbiont interaction and of the biology of Vestimentifera. Most importantly, we show that the developmental gene repertoire is conserved, and that besides the well-known nutritional aspect of the trophosome, its mesodermal origin brought an inherited suite of functions such as, hematopoiesis, endosomal digestion of endosymbionts, and storage of excretory products likely adapted to serve host–symbiont physiological interactions. Although the innate immune system is apparently downregulated in the presence of the symbiont, it is highly active in the remaining body directly exposed, or connected through openings to, to the environment.

## Results and Discussion

### *Riftia* Represents the Most Complete Annelid Genome to Date Including a Complete Mitogenome

To assess the whole-genome content of the giant tubeworm (Jones 1981), we sequenced a single individual from the hydrothermal vent site Tica, East Pacific Rise 9°50′N region, with approximately 87-fold coverage using Pacific Biosciences Sequel system (supplementary figs. 1–3 and supplementary table 1, Supplementary Material online). We found the haploid genome size (560.7 Mb with a N50 length of ∼2.8 Mb) to be smaller than previous genome-size estimates (Bonnivard et al. 2009) (table 1 and supplementary fig. 4, Supplementary Material online). The *Riftia* GC value is 40.49%, and the repeat content accounts for 29.99% of the total length of the genome with most of the repetitive landscape dominated by interspersed and unclassified lineage-specific elements (35.2%) (supplementary fig. 5, Supplementary Material online). After genome postprocessing, we identified a total of 25,984 protein coding genes with homologue, transcriptome, ab initio, and gene expression evidence. The BUSCO4 (Simão et al. 2015) genome completeness score is 99.37%. These numbers render *Riftia* the most complete annelid genome to date (Simakov et al. 2013; Li et al. 2019; Martín-Durán et al. 2020; Sun et al. 2021) (supplementary fig. 6, Supplementary Material online).

The complete reconstruction of siboglinid mitochondrial genomes including the AT-rich control region has been notoriously difficult (Li et al. 2015). In this case, we were able to obtain it due to deep long read sequencing. The 15,406 bp circular mitochondrial genome contains all expected 13 coding sequence genes, two ribosomal RNA genes, and the 22 tRNAs, typical of bilaterian mitogenomes (fig. 1*C* and supplementary fig. 7, Supplementary Material online) (Boore 1999). In contrast to two other *Riftia* reference mitogenomes (Jennings and Halanych 2005; Li et al. 2015), we recovered the full control region (D-loop), yielding a mitochondrial genome longer than those previously reported. The gene order and the number of genes are conserved among all three *Riftia* and other siboglinids reference mitogenomes, though there are size differences that are most likely due to the incomplete nature of previously published genomes.

### The Developmental Gene Repertoire in Gutless *Riftia* Is Conserved

Because of the lack of molecular information on the development of cell types and the evolution of the vestimentiferan body plan, we identified and annotated a suite of key developmental genes and signaling pathway-related genes in the giant tubeworm genome. We found that key genes involved in the development of the digestive tract in metazoans (Hejnol and Martín-Durán 2015; Nielsen et al. 2018), such as *goosecoid*, *brachyury*, *foxA*, and all three ParaHox genes, *xlox*, *cdx*, and *gsx*, present in the *Riftia* and *Lamellibrachia* genomes (supplementary figs. 8–10, Supplementary Material online). The conservation of these genes in vestimentiferans is apparently not only crucial for developmental processes but also serves the microphagous nutrition in settled larvae until nourishment by the symbionts takes over in juveniles (Nussbaumer et al. 2006).

**Fig. 2.**
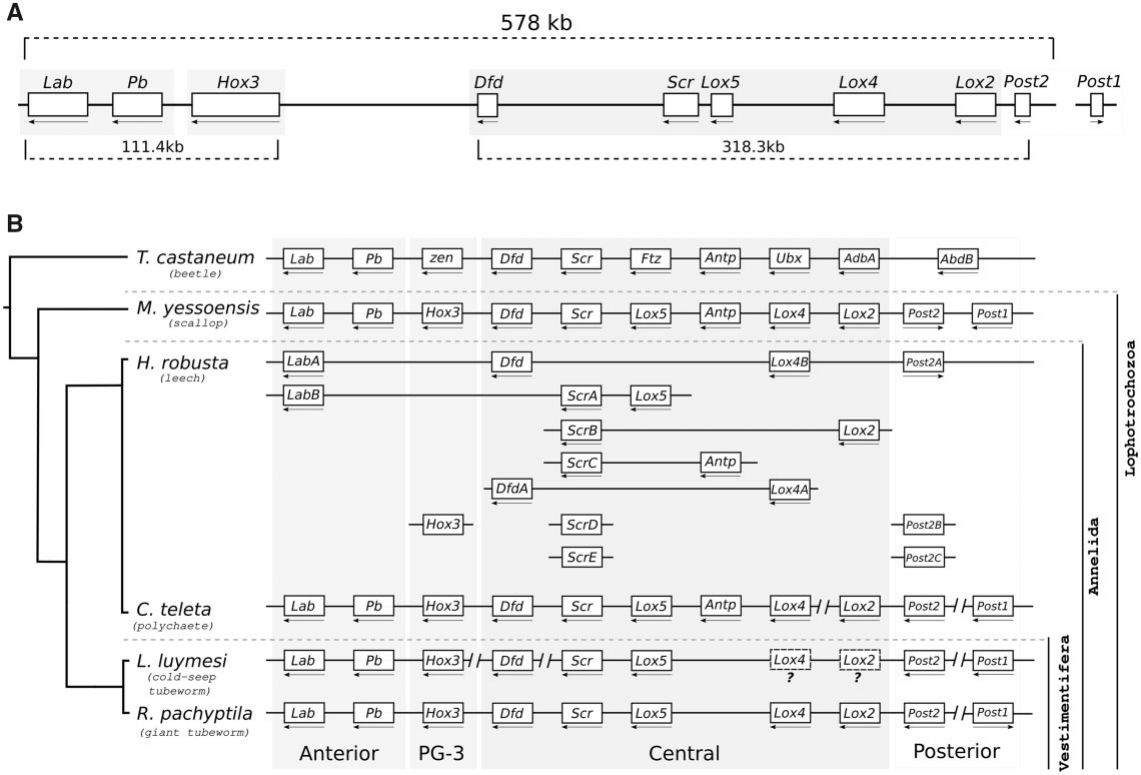
The Hox gene complement of *Riftia pachyptila* and selected metazoans. (*A*) Hox cluster organization in the genome of *Riftia pachyptila*. Nine out of the ten Hox genes are located in one single genomic scaffold. *Hox7* is missing from the giant tubeworm genome. Arrows indicate direction of transcription. Only the longest gene model is shown. (*B*) Hox cluster present in selected metazoans. Part of the central Hox class is missing from the cold-seep tubeworm *Lamellibrachia* (but see supplementary note 2, Supplementary Material online). *Helobdella* and *Capitella* clusters are based on Simakov et al. (2013), whereas *Mizuhopecten* cluster is from Wang et al. (2017).

The Hox cluster (∼578 kb in size—fig. 2*A*), homeodomain-containing transcription factors (TFs) with roles in anterior–posterior axial identity in metazoans (Pearson et al. 2005; Duboule 2007), is nearly intact and complete in the giant tubeworm genome (supplementary figs. 8 and 9 and supplementary note 2, Supplementary Material online). The same complement and synteny were identified in the chromosomal-level genome of *Paraescarpia* (Sun et al. 2021), attesting the good completeness and contiguity of the *Riftia* genome. We did not identify *hox7* in *Riftia*, indicating a secondary loss of this gene in the giant tubeworm, a pattern also observed in other lophotrochozoan representatives such as phoronids (Luo et al. 2018) and bivalves (Gerdol et al. 2015; Calcino et al. 2019). *Hox7*, *lox*2, and *lox5* are missing from *Lamellibrachia* genome suggesting a possible loss of the central Hox cluster elements (fig. 2*B*), contradicting recent results (Sun et al. 2021) (but see supplementary note 2, Supplementary Material online). The Hox-like elements, homeotic genes equally important for body plan specification and developmental processes, *gbx*, *evx*, *mox*, *mnx*, *en*, and *dlx* were also found in the giant tubeworm genome. *Engrailed* (*En*) and *even-skipped* (*Evx*) have two and four copies, respectively (supplementary fig. 8, Supplementary Material online).

Few signaling pathways are required to control cell-to-cell interactions and produce the plethora of cell types and tissues in Metazoa (Pires-daSilva and Sommer 2003) among them TGFβ, Wnt, Notch, and Hedgehog (Moustakas and Heldin 2009; Ingham et al. 2011; Holstein 2012; Massagué 2012; Niehrs 2012; Gazave et al. 2017) (supplementary figs. 11–15, Supplementary Material online), The *Riftia* genome contains 14 TGFβ genes, including *nodal* and its antagonist *lefty*, the latter previously assumed to be a deuterostome innovation (Simakov et al. 2015). *Notch* and *hedgehog* are present as single copy genes in the *Riftia* genome as well as in *Lamellibrachia*, however, the *notch* receptor *jagged* is missing from both tubeworms. *Jagged* is present in the annelids *Capitella teleta*, *Helobdella robusta*, and *Platynereis dumerilii* (Gazave et al. 2017), suggesting a secondary loss in Vestimentifera. Patched and dispatched genes, membrane receptors for the hedgehog ligand (Ingham et al. 2011) are present in *Riftia* with the dispatched genes expanded in vestimentiferans. In *Riftia*, we identified the 12 expected Wnt ligands (*Wnt3* has been shown to be lost in the Protostomia lineage) and their receptors frizzled, smoothened and sFRP (Holstein 2012). There is a genetic linkage of *Wnt1*, *6*, *9*, and *10* in *Riftia* akin to the gastropod *Lottia gigantea* and the fruit fly *Drosophila melanogaster* (Cho et al. 2010), reaffirming the ancient protostomian ancestral conserved linkage. The remaining eight Wnt genes in *Riftia* are disorganized on eight different scaffolds. Overall, despite the highly derived body plan, *Riftia* presents a deep conservation of the developmental gene toolkit akin to many distinct bilaterian animals.

### The *Riftia* Genome Is Characterized by Reductive Evolution

Multiple lines of evidence point to a relatively small genome, with gene family contractions exceeding expansions in *Riftia*, indicative of reductive evolution. The giant tubeworm genome is approximately 168 Mb smaller than *Lamellibrachia*, its relative from cold hydrocarbon seeps whose genome is approximately 688 Mb with a N50 of 373 kb (Li et al. 2019). The difference can be attributed to the increased number of repeat elements and protein coding genes in the cold seep tubeworm (38,998 gene models and repetitive content of 36.92%; supplementary note 1 and supplementary fig. 16, Supplementary Material online).

To identify clusters of orthologous genes shared among the two vestimentiferans *Riftia* and *Lamellibrachia*, the polychaete *C. teleta* (herein called *Capitella*), and the clitellid *Helobdella robusta* (herein called *Helobdella*) (Simakov et al. 2013), we employed tree-based orthology inferences (Emms and Kelly 2019). The annelid core genome, the collection of orthogroups shared among the four annelids, contains 6,349 cluster of orthologous genes. Less than half of them represent the vestimentiferan core genome (2,883 orthogroups) shared between *Riftia* and *Lamellibrachia*. Interestingly, the number of shared orthogroups between the *Riftia-Helobdella* (17) and -*Capitella* (116) pairs are smaller than those between *Lamellibrachia*-*Helobdella* (89) and -*Capitella* (349) pairs, indicating that *Riftia* contains a more derived gene repertoire than its close relative *Lamellibrachia*.

To further investigate the important processes of gene losses and gains, known to shape animal evolution (Fernández and Gabaldón 2020; Guijarro-Clarke et al. 2020), identify expanded protein domains, taxonomically restricted genes, and positively selected genes in *Riftia*, we employed multilevel comparative approaches involving statistical analysis, taxon rich orthology inferences (*N* = 36), and sensitive similarity searches (supplementary table 2 and supplementary note 3, Supplementary Material online). The *Riftia* genome shows a net reduction of gene numbers with only 734 expanded but 1,897 contracted gene families, whereas the evolutionary history of *Lamellibrachia* is characterized by gene gains. Notably, the average expansion value of gene families in *Riftia* is the lowest among the four selected annelids herein analyzed (supplementary fig. 17, Supplementary Material online). A total of 8,629 lineage-specific genes (∼33.21% of the total) were identified in *Riftia*. Compared with the giant tubeworm, *Lamellibrachia* contains more lineage-specific genes (10,262–26.31% of the total).

The contracted gene families are not restricted to any specific biological process, as revealed by our gene ontology (GO) term enrichment analysis (*N* = 18) (supplementary fig. 18, Supplementary Material online). Rather it appears that the giant tubeworm genome is undergoing a broad reduction in gene content. Among the contracted gene families are genes controlling the transcriptional machineries (supplementary fig. 19 and supplementary table 3, Supplementary Material online). TFs are proteins with sequence-specific DNA-binding domains that control gene transcription and tissue identity (Schmitz et al. 2016). To gain understanding into the repertoire of TFs in *Riftia*, we annotated and classified genes in the tubeworm genome present in five major groups of TFs (bzip, homeobox, nuclear factor, bHLH, and zinc-finger) with sensitive similarity searches. The giant tubeworm presents the lowest number of TFs within the analyzed annelids (414), supporting our gene family analysis (discussed below). The cold-seep tubeworm genome contains a similar complement size as *Riftia* (423), with *Capitella* (551) and *Helobdella* (568) presenting a higher number of TF genes, comparatively. These results point to pervasive TF losses in the Vestimentifera lineage (supplementary note 3, Supplementary Material online).

### Expanded and Lineage-Specific Gene Families in *Riftia*

Despite overall genome reduction, the *Riftia* genome exhibits, there is also a variety of expanded gene families (supplementary note 3, supplementary fig. 20, and supplementary table 4, Supplementary Material online). These expanded families are enriched with GO terms associated with sulfur metabolism, membrane transport, and detoxification of xenobiotic, for example, foreign substances (xenobiotic transmembrane transporter activity, galactosylceramide sulfotransferase activity, CoA-transferase activity) (Gamage et al. 2006), detoxification of hydrogen peroxide as antioxidative stress response (glutathione catabolic and biosynthetic processes) (Espinosa-Diez et al. 2015), neurotransmitter- and ion channel-related functions (sodium symporter activity), oxygen transport (oxygen binding, hemoglobin complex), endosomal degradation (lysozyme activity), and secretion of chitin (chitin binding, protein glycosylation) (discussed with more details later).

Genes involved in the production of extracellular components of vestimentiferans such as the cuticle and the basal matrixes as well as the tube and chaetae (Gardiner and Jones 1994) were found in expanded families of *Riftia* (as well as *Lamellibrachia*), some of which are specific to either *Riftia* or *Lamellibrachia* (supplementary note 3, supplementary figs. 21–24, and supplementary tables 5 and 6, Supplementary Material online). Additionally, integrated genomic, transcriptomic, and proteomic analyses in *Paraescarpia* revealed a fairly similar scenario (Sun et al. 2021), pointing to common and shared molecular mechanisms involved with tube formation in cold-seep and vent vestimentiferans.

The *Riftia* genome contains expanded protein domains related to several high-molecular mass proteins such as laminin, nidogen, and collagen. These proteins are part of extracellular matrix secreted basally from epithelia, also known to regulate cellular activity and growth in other animals (Timpl and Brown 1996). In *Riftia*, extensive short collagen fibers are found below the epidermis, extending between muscles cells, and building the matrix of the obturaculum. In addition, long helically arranged collagen fibers are the main component of the cuticle apically secreted from the epidermis (Gardiner and Jones 1994). Importantly, many genes involved in chitin production, a biopolymer part of the hard protective tube secreted from pyriform glands of the vestimentum, trunk, body wall, and opisthosoma (Gardiner and Jones 1993), are taxonomically restricted to the *Riftia* lineage. Expectedly, we identified in the vestimentum and body wall tissues of *Riftia* several tissue-specific genes (TSGs) involved in the chitin metabolism responsible for the tube production as well as dissolution (supplementary figs. 25 and 26, Supplementary Material online). Although the specific gland type responsible for dissolution of tube material has yet to be identified, we suggest that in *Riftia* the straight tube, that can reach up to 3 m in length and 5 cm in diameter (Gaill and Hunt 1986; Grassle 1987; Fisher et al. 1988), can only widen in diameter to accommodate growth of the worm when tube material is dissolved and newly secreted, which agrees with the distribution of many TSG involved with tube biosynthesis. Overall, our findings of these expanding gene families as well as gene expression patterns underline the importance of chitin in *Riftia*, which is considered one of the fastest growing invertebrates (Lutz et al. 1994, 2001). In order to achieve these high growth rates, *Riftia* needs to both digest and remodulate its own tube with astonishing speed.

Furthermore, the multilevel comparative analyses revealed an enrichment of GO terms in the lineage-specific *Riftia* genes involved with the control of the chromosome condensation and nucleosome assembly, and positively selected genes related to tumor suppression (*PIN2/TERF1*-interacting telomerase inhibitor) and transcription initiation (*TFIIB*- and *-D*) (Roeder 1996; Zhou and Lu 2001). Interestingly, in *Lamellibrachia smad4* (Li et al. 2019), which is a tumor suppressor and TF, is under positive selection, suggesting a common vestimentiferan evolutionary adaption responsible for controlling the chromatin-remodeling events and the extraordinarily cell proliferation rates in these two tubeworms (supplementary table 7 and supplementary note 3, Supplementary Material online) (Pflugfelder et al. 2009).

The protein annotation of the rapidly evolving expanded gene families in *Riftia* identified members of the complement system involved in innate immunity and self-, nonself-recognition (sushi repeat domain-containing protein) (Kirkitadze and Barlow 2001). *Riftia* contains the greatest number of sushi-domain-containing proteins among lophotrochozoans, presenting a total of 42 copies which are organized either in genomic clusters or dispersed as single elements throughout the genome (supplementary figs. 4 and 27, Supplementary Material online). Of these, only 40 are shared with the cold seep tubeworm *Lamellibrachia*, pointing to a lineage-specific expansion at the base of Vestimentifera. Sushi genes, a common component of hemocytes (i.e., immune cells with phagocytic function; Pila et al. 2016), have been implicated in the mediation of the host–symbiont tolerance in the bobtail squid *Euprymna scolopes*—bioluminescent *Aliivibrio fischeri* association (McAnulty and Nyholm 2017). Although the rapid evolution of these proteins in *Riftia* and *Lamellibrachia* suggests similar evolutionary adaptations to the tubeworm/endosymbiont mutualism, the absence of any significant expressions in adult tissues rather point to their involvement in recognition of the symbiont during transmission in the larval stage or to potential pathogen recognition upregulated upon exposure.

### Substrate Transport for Energy Conservation and Biosynthesis Is Supported by Lineage-Specific Adaptations and Parallel Evolutionary Events in *Riftia*

**Fig. 3.**
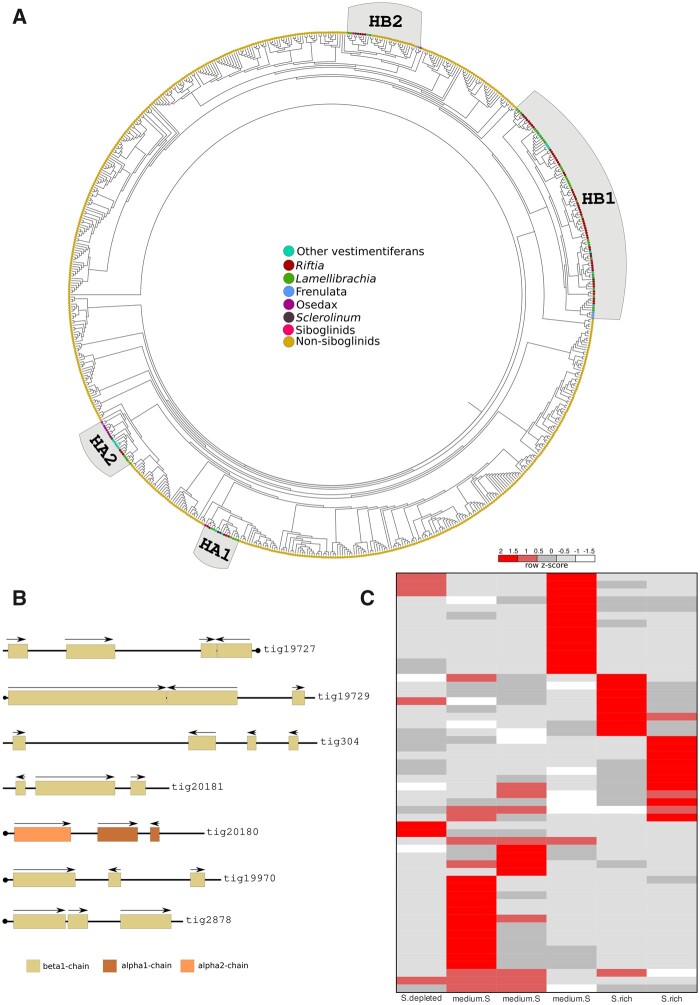
Expanded hemoglobin complement in *Riftia pachyptila*. (*A*) Midpoint rooted phylogeny of 693 *Riftia*, annelid and metazoan hemoglobin genes, using Belato et al. (2019) as backbone. Colored circles correspond to different annelid taxa and metazoans. (*B*) Seven genomic hemoglobin clusters are present in the *Riftia* genome. Arrows indicate the direction of transcription. Scaffolds with a circle on the end indicate the presence of Hb genes in the terminal end of the genomic segment. Only the longest gene models are shown. Colors represent the different hemoglobin chains. (*C*) Heat map expression of hemoglobins in the trophosome under three experimental conditions (obtained from Hinzke et al. [2019]): medium sulfide (medium.S), sulfide rich (S.rich), and sulfide depleted (S.depleted).

As an adaptation to the sulfidic vent environment, and in support of a symbiotic lifestyle, the respiratory pigments in *Riftia*, and other vestimentiferans such as *Lamellibrachia*, bind noncompetitively and reversibly to oxygen and sulfide, simultaneously providing a key substrate for chemosynthesis by the symbionts while also averting the sulfidic inhibition of the hosts’ mitochondrial oxidative chain reactions (Arp and Childress 1983; Terwilliger et al. 1985). Our previous gene family evolution analysis identified an expansion of hemoglobins in the giant tubeworm genome compared with other nonvestimentiferan lophotrochozoans (supplementary fig. 21 and supplementary table 6, Supplementary Material online and fig. 3*A*). Additionally, a recent genome study found a massive expansion of β1-hemoglobin in the cold seep tubeworm *Lamellibrachia* (Li et al. 2019). To gain better insights in the evolution of Hb and linker genes in the Vestimentifera lineage, we employed thorough comparative genomics, phylogenetics, domain composition, and gene quantification analyses. The genomic arrangement of the giant tubeworm Hb genes indicates that they were originated through a series of tandem duplications, totaling seven distinct genomic clusters (fig. 3*B*). We annotated 26 extracellular Hbs and six linker genes in the *Riftia* genome (supplementary figs. 28 and 29 and supplementary note 4, Supplementary Material online). Twenty-two Hb genes were phylogenetically placed in the β1-Hb group, surpassing previous estimates of the β1-Hb complement in the giant tubeworm (Bailly et al. 2002; Sanchez et al. 2007; Hinzke et al. 2019). α2- and β2-Hbs are found as single copy genes, whereas α1-Hb group contains two paralogous genes. The sulfide-binding ability of the *Riftia* Hbs is associated with the occurrence of free cysteine residues in one α2 and one β2 Hb genes (Bailly et al. 2002), as well as the formation of persulfide groups on linker chains (Zal et al. 1998; Bailly et al. 2002). Our results show that seven additional paralogous genes belonging to the β1-Hb group contain the putative free-cysteine residues, which were confirmed through multiple sequence alignments and homology model generation (supplementary figs. 30 and 31 and supplementary note 4, Supplementary Material online). Additionally, it has been hypothesized that zinc ions, rather than free-cysteine residues, are responsible for the H2S binding and transport on vestimentiferan α2 chains (Flores et al. 2005). We identified the three conserved histidine residues (B12, B16, and G9), predicted to bind zinc moieties, in *Riftia* Hb genes. However, we observed variations within the *Lamellibrachia* α2 genes. A broader comparison of α2-Hb genes belonging to different annelid taxa challenged the hypothesis of zinc sulfide-binding mechanisms for H2S in siboglinids and vestimentiferans (Li et al. 2019). Our results, solely based on the conservation of histidine residues, corroborate Flores et al. (2005) hypothesis that zinc residues may be involved in the sequestration and transport of hydrogen sulfide at least on the giant tubeworm.

To investigate the gene expression dynamics of the newly and previously identified Hb paralogs in *Riftia*, we analyzed published transcriptomes sampled from *Riftia*’s trophosomes containing sulfur-rich to sulfur-depleted symbionts (Hinzke et al. 2019). Hb gene expression showed great variation, indicating a more specialized role of the Hbs according to the environmental chemical fluctuations in the unstable deep-vent ecosystem (fig. 3*C* and supplementary note 4 and supplementary table 8, Supplementary Material online). Taken together, these results suggest a more complex system coordinating oxygen-sulfide sequestration and distribution in the giant tubeworm tissues. The Hb complement of *Riftia* and *Lamellibrachia* is similar and unique among annelids and lophotrochozoans, in respect to gene numbers and distribution, indicating a Vestimentifera synapomorphy.

As the endosymbionts require carbon dioxide (CO_2_) for fixing inorganic carbon, the transport of CO_2_ and the conversion of its alternative forms (e.g., bicarbonate; HCO_3_^−^) is mediated by another class of enzymes, the carbonic anhydrases (CAs) (Shively et al. 1998; Cian et al. 2003). We found ten CA genes in the *Riftia* genome, from which seven are tandemly arrayed in two genomic clusters (supplementary fig. 32, Supplementary Material online). A similar CA complement in *Riftia* (nine genes) was found in a recent study (Hinzke et al. 2019). To better understand the diversity of CA genes, we analyzed tissue-specific transcriptomes and found at least five CA genes are membrane bound with three of them moderately/highly expressed in the trophosome, indicating that HCO_3_^−^ conversion to CO_2_ and diffusion across the bacteriocyte membrane might be a common process in the trophosome, as suggested previously (Sanchez et al. 2007; Bright and Lallier 2010; Hinzke et al. 2019). Tandem duplications and tissue-specific CA expression linked to the intracellular supply of CO_2_ to endosymbionts have been recently reported in deep-sea bivalves (Ip et al. 2021), showing remarkable resemblance to our findings. Taken together, our results show that the transport of essential compounds to the chemoautotrophic endosymbionts and the maintenance of the mutualistic relationship are driven by lineage-specific and parallel evolutionary events.

### Trait and Gene Losses Are Compensated by the Endosymbionts

The loss of the digestive system requires nourishment through the symbiont. The mechanisms of carbon transfer between the endosymbiont and *Riftia* were shown to be through the fast release of fixed carbon from the symbiont and uptake into host tissue, as well as, through symbiont digestion prior death of the bacteriocytes (Felbeck 1985; Hand 1987; Felbeck and Jarchow 1998; Bright and Lallier 2010; Hinzke et al. 2019). We found corroborating evidence for the uptake of released organic carbon from the symbiont based on the enrichment of GO terms and tissue specificity of succinate-semialdehyde complex genes and nuclear-encoded proteins of the inner mitochondrial membranes (including the tricarboxylate mitochondrial carrier responsible for the transport of succinate) (Majd et al. 2018) in the trophosome (supplementary fig. 33 and supplementary table 9, Supplementary Material online). These results suggest an increased movement of cytosolic succinate through the mitochondrial membrane, possibly increasing the ATP production via the oxidative metabolism. These findings corroborate previous findings and support the involvement of this molecule for nourishment in *Riftia* from its endosymbiont (Felbeck and Jarchow 1998).

Evidence of digestion was revealed with tissue-specific transcriptome analyses which allowed us to identify the genes involved in the successive stages of lysosomal-associated degradation of symbionts (supplementary fig. 33 and supplementary table 8, Supplementary Material online). The expression of genes associated with endosomal activity, the expression of several lysosomal-associated hydrolases (supplementary figs. 34 and 35, Supplementary Material online), vacuolar ATPases, and small Ras-related GTPases (rab genes) (supplementary fig. 36 and supplementary note 5, Supplementary Material online) in the trophosome, is indicative of lysosomal-associated degradation of symbionts, as suggested earlier in ultrastructural studies, which describe the presence of primary lysosomes and symbionts in different lytic stages in *Riftia* (Bright et al. 2000; Bright and Sorgo 2005; Hinzke et al. 2019). The high expression of cathepsins and/or legumains in the trophosomes of the cold-seep tubeworms *Lamellibrachia* and *Paraescarpia* (Li et al. 2019; Sun et al. 2021) strongly indicates that endosymbiont lysosomal digestion route is widespread in Vestimentifera. In addition, we detected specific genes in the trophosome which are associated with actin cytoskeleton dynamics (ARP2/3) known to be essential for endosomal dynamics (Kast and Dominguez 2017) (supplementary fig. 33 and supplementary table 9, Supplementary Material online). Recent de novo tissue-specific transcriptomes and gene expression quantification of the host and symbiont support the digestive route of nutrition in *Riftia*/Endoriftia symbiosis (Hinzke et al. 2019).

**Fig. 4.**
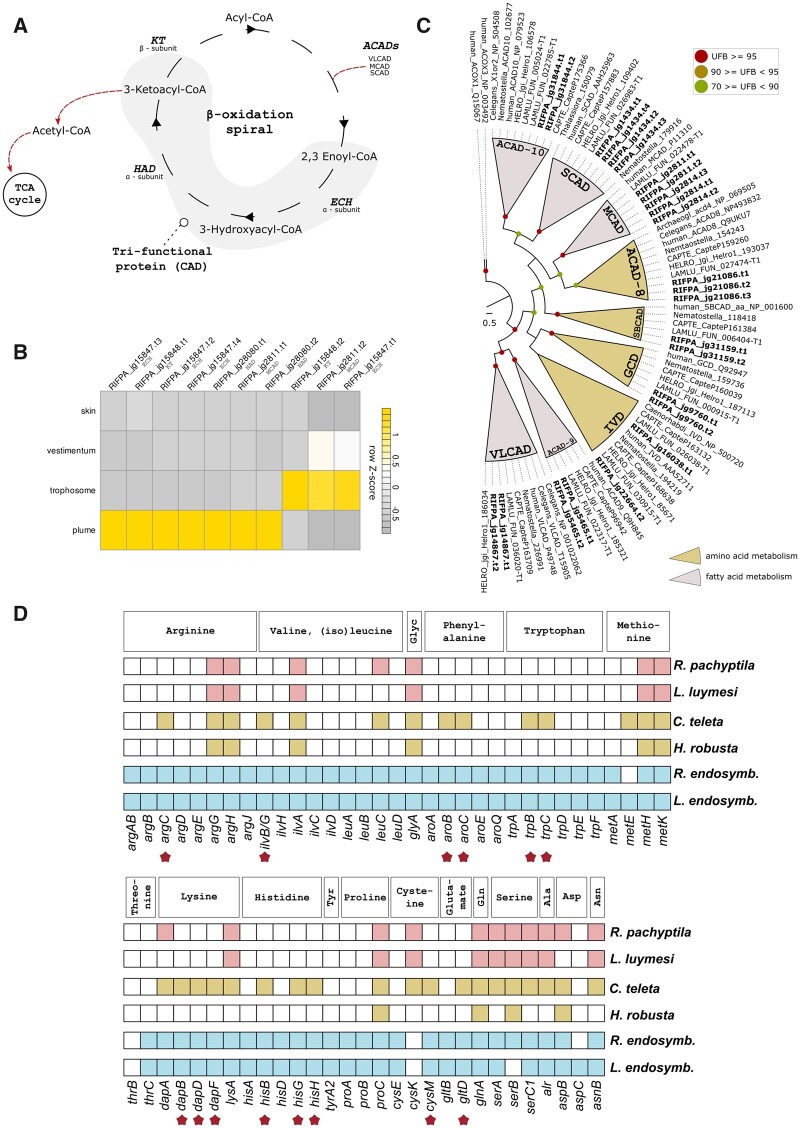
Amino acid and fatty acid biosynthesis in *Riftia pachyptila*. (*A*) A schematic representation of mitochondrial FAO. The fatty acid degradation is performed in four enzymatic steps involving the membrane bound mitochondrial trifunctional protein and acyl-CoA dehydrogenases. The resulting acetyl-CoA is further oxidized in the TCA cycle. (*B*) Expression profile of FAO genes. Color coding reflects the expression patterns based on row *Z*-score calculations. (*C*) Maximum-likelihood phylogenetic tree inference of the ACAD genes using 1,000 rapid bootstrap replicates. The branch support values are represented by the colored circles in the tree nodes. Accession numbers for NCBI database are displayed after the species names and homologs were retrieved from a previous study (Swigoňová et al. 2009). *Capitella*, *Helobdella*, and *Lamellibrachia* gene identification are derived from the publicly available annotated genomes. (*D*) Key enzymes related to amino acid biosynthesis identified in *Riftia*, selected annelids and two tubeworm endosymbiont genomes. The identification of the genes was performed with KEGG and orthology reconfirmed with similarity searches against the ncbi-nr protein database. *Riftia* and *Lamellibrachia* lack many amino acid biosynthesis genes indicating nutritional dependence on their endosymbionts. Stars represent genes present in the free-living polychaete *Capitella* and Endoriftia but absent in *Riftia* (based on Li et al. [2019] and Tokuda et al. [2013] schemes).

Furthermore, genes involved in the transport of fatty acyl units into the mitochondrial matrix (e.g., carnitine/acylcarnitine carrier) (Indiveri et al. 2011), tricarboxylic acid (TCA) cycle, oxidative phosphorylation, antioxidant systems (i.e., superoxide dismutase II genes and methionine sulfoxide reductases) (supplementary fig. 38, Supplementary Material online), and key players of the fatty acid β-oxidation (FAO) (fig. 4*A*–*C*), showed tissue specificity in the trophosomal tissue (supplementary table 9 and supplementary note 5, Supplementary Material online). FAO is a central and deeply conserved energy-yielding process that fuels the TCA cycle and oxidative phosphorylation (Houten et al. 2016). As *Riftia* relies solely on its endosymbionts for sustenance, the metabolism of fatty acids in the trophosome is certainly linked to the bacterial digestion in this tissue, which is corroborated by a previous proteomic study (Hinzke et al. 2019). Altogether, the results point to different modes of nutrient transfer in the trophosome involving the translocation of released nutrients from symbiont to host through succinate, and the digestion of the symbionts by lysosomal enzymes followed by the degradation of fatty acids using the mitochondrial β-oxidation pathway.

It has been previously described that deep-sea tubeworms are reliant on their endosymbionts for nutrition (Li et al. 2019; Yang et al. 2020). To further explore the nutrient interdependence of *Riftia* and its endosymbiont, we screened the genome of giant tubeworm and selected annelids for key enzymes related to amino acid biosynthesis (supplementary table 10, Supplementary Material online). We found that *Riftia*, together with cold-seep tubeworm *Lamellibrachia* and the parasitic leech *Helobdella*, lacks many key enzymes related to amino acid biosynthesis when compared with close free-living polychaete relative *Capitella* (fig. 4*D*). The identified key enzymes in the giant tubeworm are involved in the biosynthesis of at least 14 amino acids (fig. 4*D*) similar to *Paraescarpia* and *Lamellibrachia* hosts (Li et al. 2019; Yang et al. 2020), whereas Endoriftia possesses the metabolic capability of synthesize all essential and nonessential amino acids. The complete gene toolkit for amino acid biosynthesis is also found in the *Lamellibrachia* endosymbiont (Li et al. 2019; fig. 4*D*) but it is more reduced in the endosymbiont of *Paraescarpia* (which lacks the ability to synthesize threonine and tyrosine) (Yang et al. 2020). Genes involved with amino acid biosynthesis are constitutively expressed across the giant tubeworm tissues, with enzymes related to arginine and glycine metabolism highly expressed in the trophosome (supplementary fig. 39, Supplementary Material online). These findings suggest that loss of key enzymes in mutualistic vestimentiferans as well as a parasitic leech may be due to the beneficial and parasitic relationships, respectively allowing for compensated gene loss, compared with free-living polychaetes.

Overall, endosomal-associated digestion of endosymbionts seems to be a hallmark of intracellular digestion accomplished in the mesodermal trophosome of vestimentiferans, such as *Riftia*, *Lamellibrachia*, and *Paraescarpia* (Nussbaumer et al. 2006; Hinzke et al. 2019; Li et al. 2019; Sun et al. 2021) (see also below). This process serves the host nutrition as well as the control of the symbiont population density during host growth, known from many other symbioses (Angela E. Douglas 2010). In addition, the symbiont provides the host with released organic carbon. Although we do not know yet which partner controls this mode of nutritional translocation, both the evolutionary adaptation of endosymbiont digestion in a mesodermal tissue as well as carbon release contributed to trait loss in one partner compensated by the other (Ellers et al. 2012), and consequently has made *Riftia* obligatorily associated with its symbiont.

### Hematopoiesis Operates in the Trophosome of *Riftia*

Hematopoiesis, the production of blood cells and pigments, is still a poorly understood process in vestimentiferans. The heart body, a mesodermal tissue in the dorsal blood vessel of vestimentiferans has been hypothesized to be the site of hemoglobin biosynthesis (Schulze 2002). The presence of many TSGs in the trophosome related to 5-aminolevulinate synthase, porphyrin metabolism, and metal ion binding indicate that this tissue harbors the enzymatic machinery necessary for heme biosynthesis. Heme is an integral part of hemoglobin molecules, which is synthesized in a seven multistep pathway that begins and ends in the mitochondrion. To fully characterize the heme biosynthesis pathway in the giant tubeworm, we screened the *Riftia* genome for the presence of the seven universal enzymes required to synthesize the heme (supplementary fig. 40 and supplementary note 5, Supplementary Material online). The giant tubeworm contains all the seven enzymes present as single copy in its genome with recognizable orthologs in the annelids *Lamellibrachia*, *Capitella*, and *Helobdella*. Gene expression analysis showed that the key enzymes present in the heme biosynthetic pathway are moderately/highly expressed in the trophosome, supporting the GO enrichment analysis (supplementary figs. 40 and 33, Supplementary Material online). The heme biosynthesis in the trophosome is further corroborated by the presence of TSGs in this tissue related to phosphoserine aminotransferase and the mitochondrial coenzyme A transporter, which act as an important cofactor in the final step of heme synthesis and in the transport of coenzyme A into the mitochondria, respectively (Schneider et al. 2000; Fiermonte et al. 2009). These findings confirm the involvement of the mesodermal trophosome in hemoglobin metabolism and suggest that this organ is the site hematopoiesis. In the frenulate *Oligobrachia mashikoi*, the visceral mesoderm also strongly expresses globin subunits based on in situ hybridization and semiquantitative RT–PCR (Nakahama et al. 2008), but in this siboglinid, the visceral mesoderm is organized as simple peritoneum surrounding the endodermal trophosome (Southward 1993).

### Excretory Products Are Stored in the Trophosome of *Riftia*

The finding of TSGs in the trophosome related to the biosynthesis of nitrogen-containing compounds and in the transport of ornithine (supplementary tables 9 and 11, Supplementary Material online) agrees with the high levels of uric acid and urease activity in this host tissue, as previously reported (Cian et al. 2000; Minic and Hervé 2003).To explore the nitrogen metabolism pathways in *Riftia*, we identified and quantified the gene expression of several enzymes related to the purineolytic/uricolytic, purine/pyrimidine, taurine, and the polyamine pathways, as well as the urea and ammonia cycles (supplementary figs. 41–45 and supplementary note 6, Supplementary Material online).

**Fig. 5.**
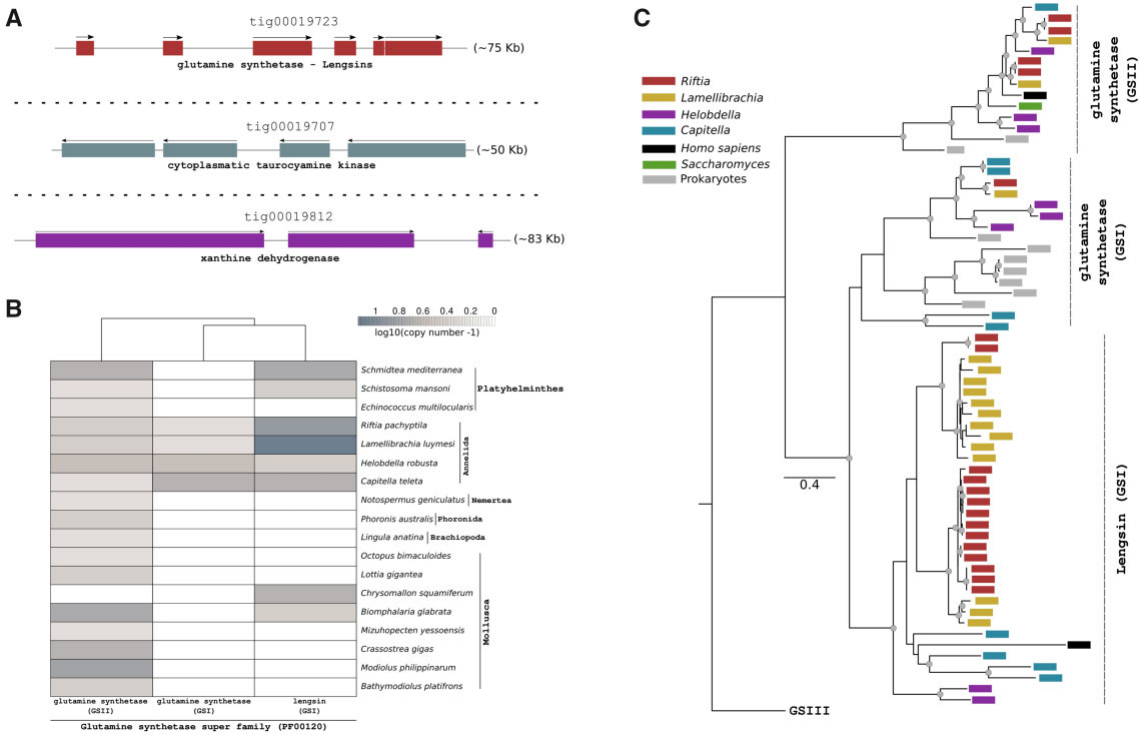
Genomic clusters of important genes related to the nitrogen metabolism in *Riftia pachyptila*, and phyletic distribution and phylogeny of glutamine synthetase genes among selected metazoans. (*A*) Genomic organization of important genes related to nitrogen metabolism in *Riftia*. Arrows indicate the direction of transcription. Only the longest gene models are shown. (*B*) Distribution of glutamine synthase-related genes in the giant tubeworm, closely related annelids, and selected lophotrochozoans. (*C*) Maximum-likelihood phylogenetic tree inference of members of the glutamine synthetase superfamily using 1,000 ultrafast bootstrap replicates. Colored boxes correspond to different annelids, vertebrates, yeast, and prokaryotes.

Most of the identified genes are found as single copy in the giant tubeworm genome (supplementary note 6 and supplementary figs. 41–45, Supplementary Material online); however, we identified the presence of three chromosomal clusters harboring glutamine synthetase, cytoplasmatic taurocyamine kinase, and xanthine dehydrogenase/oxidase genes (fig. 5*A*). These enzymes are involved in the ammonia, urea, and uricolytic pathways, respectively. *Riftia* and *Lamellibrachia* contain the highest number of glutamine synthetase genes in the herein investigated lophotrochozoan genomes (fig. 5*B*). Seven out of the nine glutamine synthetase genes present in *Riftia* belong to the group I and the remaining to the group II (fig. 5*C*). Interestingly, only annelid orthologs are found to be phylogenetically close to the prokaryotic group I, indicating a secondary loss of this genes in the remaining lophotrochozoan lineages (see supplementary fig. 43, Supplementary Material online, for the expanded version of the phylogenetic tree). An expanded set of lengsin genes, an ancient class I glutamine synthetase family (Wyatt et al. 2006), is present in Vestimentifera (seven copies in *Riftia* and 13 in *Lamellibrachia*). Some members of the newly identified lengsins are also highly expressed in the trophosome, suggesting that these enzymes might play a role in mitigating toxicity of urea, ammonia, and other nitrogenous compounds (Wyatt et al. 2006).

The identification of five cytoplasmatic taurocyamine kinase genes, four organized in a genomic cluster (fig. 5*A*), surpasses previous reports (in which only one cytoplasmatic gene was identified; supplementary fig. 44, Supplementary Material online) (Uda et al. 2005). In accordance with a recent study (Hinzke et al. 2019) and contrasting previous biochemicals investigations on the de novo pyrimidine and polyamine biosynthesis in *Riftia* (Minic et al. 2001; Minic and Hervé 2003), we identified the trifunctional CAD protein in the genome of the tubeworm reinforcing the notion that *Riftia* can catalyze the first steps of the pyrimidine synthesis independently of its endosymbionts (supplementary note 6, Supplementary Material online). These results are not unforeseen, since during the aposymbiotic phase (i.e., *Riftia*’s fertilized egg until the settled larva) pyrimidine metabolism plays a fundamental role in the development and growth of the animal.

We also found that the key enzymes of the uricolytic pathway and urea cycle are highly active in the trophosome (supplementary fig. 41 and supplementary table 8, Supplementary Material online). These results are consistent with enzymatic/light micrograph studies, which show that the trophosome contains high concentration of ammonia, urea, creatinine, and uric acid crystals in the periphery of the lobules (Cian et al. 2000), and with a more recent transcriptomic and metaproteomic study (Hinzke et al. 2019). Surprisingly, we only identified in the complete and closed (de Oliveira AL, in review) and previous endosymbiont genome drafts the subunit-A of the urea transporter (*urtA*), with the four remaining subunits missing (*urtBCDE*) (Veaudor et al. 2019). Since all five subunits are required for a proper function of the urea transporter, these results challenge the idea of an active shuttle of urea from the host to the endosymbiont (Robidart et al. 2008).

### Cell Proliferation and Cell Death Interplay with Innate Immunity in *Riftia*

To better understand how fast growth (Lutz et al. 1994) fueled by high proliferation rates (Pflugfelder et al. 2009) and innate immunity act in *Riftia* in tissues exposed to the environment and in the endosymbiont-housing trophosome, we characterized the key molecular components, and their gene expressions, of important pathways related to cell cycle signaling (supplementary fig. 46, Supplementary Material online), Toll-like receptor/MyD88 (supplementary figs. 47–49, Supplementary Material online), as well as the apoptotic (supplementary figs. 50–57, Supplementary Material online) and autophagic (i.e., macroautophagy) cell death events (supplementary fig. 58, Supplementary Material online). The *Riftia* genome similar to other investigated lophotrochozoans and closely related annelids harbors all the key components of these conserved pathways (supplementary note 7, Supplementary Material online) (Zhang, Fang, et al. 2012; Sun et al. 2017; Luo et al. 2018; Li et al. 2019; Ip et al. 2021). We did not identify any extensive remodeling (i.e., gene family expansions and contractions) of the immune (with the exception of sushi genes) and programed cell death components in the giant tubeworm genome, as shown to be important in the maintenance of host–symbiont interactions in deep-sea mussels and clams (Sun et al. 2017; Ip et al. 2021).

Overall, *Riftia*’s gonad and plume tissues are highly active in cell proliferation and programed cell death. Subject to potential pathogen infections through the gonopore opening and direct contact to the vent water (Jones 1981), respectively, these tissues show the entire suite of genes involved in the innate immunity recognition with TLRs, downstream cellular immune responses, as well as apoptosis, autophagy, and endosomal-related genes. These results were additionally supported by the GO enrichment analyses in the female gonad and plume tissues (supplementary figs. 59 and 60, Supplementary Material online). The trophosome, in contrast, despite the remarkably high bacterial population density (Powell and Somero 1986; Bright and Sorgo 2005) does not show any striking upregulation of TLR for endosymbiont recognition, nor cell proliferation, nor programed cell death pathways (at least not in the classical sense; see Hinzke et al. 2019). Instead, we found few moderately/highly expressed genes present in the immune system (*irak2* and 4, *tab1*, *tak1*, *mkk3/6*), cell cycle (cyclin A, B2, D2, *cdk4*), apoptotic (*cas2*, *cas8*, *birc8*), and autophagic (*becn1*, *atg2b-7-8-16*) pathways in the trophosome of *Riftia*. Interestingly, a previous study suggested that immune-related genes were significantly more expressed in the trophosome in relation to other symbiont-free tissues in the siboglinid *Ridgeia piscesae*, positing a more important role of the immune system in the host-endosymbiont homeostasis (Nyholm et al. 2012).

Few other individual components of the innate immune system, that is, bactericidal permeability-increasing proteins and pattern recognition receptors, have been implicated in symbiont population control in tubeworms (Nyholm et al. 2012; Hinzke et al. 2019). However, based on our broad gene expression analyses, we argue that the host immune system does not play a major role in taming the endosymbiont population in the trophosome, as previously suggested (Hinzke et al. 2019). Furthermore, immunohistochemical and ultrastructural cell cycle analyses identified apoptotic and proliferative events in the trophosome (Pflugfelder et al. 2009), indicating that despite the overall low expression of gene markers related to these pathways described herein, these events occur in this tissue. In which extent these different pathways interact to shape the host/symbiont interactions and to maintain tissue homeostasis remains to be shown, however, it is clear that multiple and not mutually exclusive programed cell-death, immune-related, and proliferative events (supplementary note 7, Supplementary Material online) are acting on the trophosome.

### From Phenotype to Genotype and Back

After 40 years of intensive research, we are now finally able to integrate the obtained genome and tissue-specific transcriptome information with the current body of knowledge on the phenotype to better understand the genotype–phenotype interplay in the giant tubeworm. The *R. pachyptila* genome is characterized by reductive evolution with broad gene family contractions exceeding gene family expansions. Compared with the close relative *L. luymesi* (*Lamellibrachia* live at longer-lived and less physiologically taxing hydrocarbon seeps), *Riftia* exhibits a more derived gene repertoire for important traits related to symbiosis and the highly disturbed and stressful hydrothermal vent habitat they inhabit in the deep sea.

The mutualism between *Riftia* and its symbiont has not transited from individuality of symbiotic partners to a new integrated organism (Szathmáry and Smith 1995) because it lacks mutual dependency (Kiers and West 2015; West et al. 2015). *Riftia* is, in fact, one of the few examples known in which dependency is asymmetric with a facultative horizontally transmitted symbionts, which have the capacity to live with or without the host. The *Riftia* host, however, is obliged to partner with the symbiont or else they cannot thrive. Therefore, the host’s fitness is strictly tied to the persistence of this association over ecological and evolutionary time scales. The genome data now clearly show the peculiarities and divergencies in *Riftia*’s genotype compared with closely related free-living annelids and other lophotrochozoans, as well as which evolutionary adaptations of the host genotype ensure the maintenance of the association.

We found that despite the drastic morphological remodeling during its early development leading to the mouth-, gutless adult animal, *Riftia* retained the highly conserved developmental gene repertoire present in other lophotrochozoans and distant related animals. These results can be interpreted as counterintuitive considering that the adult body plan alone provides little unambiguous evidence of the vestimentiferan phylogenetic relationship. These animals were initially compared with deuterostomes (Caullery 1914) and considered related to hemichordates (Beklemishev 1944) as well as protostomes, but so unique that new phyla were erected to accommodate them (i.e., Pogonophora and Vestimentifera) (reviewed by Rouse [2001] and Pleijel et al. [2009]). The conservation of the developmental gene toolkit probably reflects the developmental constraints into the necessary to go step by step through deterministic stereotypic spiral cleavage and larval development (Nielsen 2004). Akin to other polychaetes, the endoderm is necessary not only later for feeding functions, also seen in the metatrochophore larvae prior symbiont infection (Nussbaumer et al. 2006), but also to develop most mesodermal tissue.

Combining the genomic information with tissue-specific transcriptomes allows us to hypothesize that the mesodermal trophosome (Nussbaumer et al. 2006; Bright et al. 2013) is a multifunctional organ with ancestral inherited functions such as hematopoiesis. This trait, we hypothesize belongs to the functional repertoire known from mesodermal chloragogen (extravasal tissue surrounding the gut and blood vessels) derived from the visceral mesoderm in annelids like the trophosome in vestimentiferans (Nussbaumer et al. 2006; Bright et al. 2013). In fact, van der Land and Nørrevang suggested already in 1975, long before the symbionts were detected, that the trophosome in *L. luymesi* is the nutritive chloragogen tissue (Van der Land 1975). Although overall knowledge is fragmentary, it has been suggested that hematopoiesis in annelids is carried out by visceral as well as somatic mesoderm (Hartenstein 2006; Grigorian and Hartenstein 2013). In various polychaete species, it was localized in particular in the (extravasal) chloragogen tissue, the (intravasal) heart body (Potswald 1969; Friedman and Weiss 1980; Braunbeck and Dales 1984; Fischer 1993), or the somatic peritoneum (Eckelbarger 1976). Our data support the production of hemoglobin in the trophosome. Whether coelomocytes, known to be the immunocompetent cells of eucoelomates (Vetvicka and Sima 2009) including annelids (Dales 1964; Salzet et al. 2006; Cuvillier-Hot et al. 2014), and the hemocytes also develop from trophosomal tissue appears to be likely but remains to be verified.

The trophosome, however, further shows adaptations to new functions such as the well-known intracellular digestion through endosomal-like maturation of symbiosomes, as well as the processing of ammonia and storage of nitrogen waste analogous to the vertebrate liver. Most aquatic invertebrates, including annelids, are virtually ammoniotelic secreting ammonia (Larsen et al. 2011). Surprisingly, *Riftia* employs an additional ureotelic metabolism similar to terrestrial invertebrates and vertebrates converting toxic ammonia to urea/and or uric acid. Specifically, we found the entire set of genes for a complete urea cycle known to detoxify ammonia in the *Riftia* genome, with most of them upregulated in the trophosome. Therefore, we hypothesize that the trophosome share similar functions to the liver of vertebrates: instead of secreting nitrogenous waste products through kidneys like in vertebrates or nephridia in annelids, the trophosome was found to store large amounts of uric acid and urea. Uric acid and urea can be utilized as a bioavailable source of N via the catabolic arm of the urea cycle yielding ${\text{NH}}_{4}^{+}$ and CO_2_. Given the lack of urea transporters in the symbiont’s genome, and the presence of active ureases in the trophosome host tissue, this suggests that both the synthesis and breakdown of uric acid and urea is under host control.

What factor(s) might lead to the evolution of this physiological capacity to sequester and metabolize urea and uric acid? It has been shown in other symbioses that the exchange of bioavailable N between symbiotic partners plays an important role in recycling bioavailable N, such as in coral-dinoflagellate symbiosis that show an almost complete retention of bioavailable N (Tanaka et al. 2018). At many deep-sea vents, including those where *Riftia* thrive, bioavailable N is limited as ammonium and free amino acids are found in pM concentrations (Johnson et al. 1988). Moreover, *Riftia* are unable to ingest particulate matter so they cannot derive nitrogen from detritus. However, an abundant source of N is nitrate, which is found in deep seawater and can be reduced to ammonium by some microbes (Girguis et al. 2000). Previous studies (Hentschel and Felbeck 1993; Girguis et al. 2000) found that *Riftia* take up nitrate from their environment, and the symbionts reduce nitrate to ammonium for symbiont and host growth and biosynthesis. However, the *Riftia* host’s ability to produce urea means that if can sequester bioavailable N that is only available to the host. At first glance, limiting symbiont access to N might be considered a way to control symbiont growth, as seen in cnidarian-Symbiodiniaceae (Xiang et al. 2020). This latter scenario, however, seems unlikely as there is ample bioavailable N (in the form of ammonium) throughout the trophosome in both freshly collected and experimentally tested worms (De Cian et al. 2000; Girguis et al. 2000). Rather, it seems plausible that *Riftia*’s production of urea allows the host to store and sequester N in a stable, largely nontoxic form. Whether urea is mobilized and provided to the host and symbionts during time of low N availability has yet to be experimentally tested, but this physiological capacity is another example of the remarkable adaptions found within that host, which allow it to modulate the rapid environmental changes found at vents and continue to provide for its own and the symbionts’ metabolic demands.

Although the physiological and evolutionary aspects of tubeworm endosymbiosis have been sufficiently addressed over the past 40 years, the molecular mechanisms regulating host and symbiont interactions in siboglinids are still not fully understood. An immuno-centric view has been explored to explain the maintenance and regulation of the endosymbiont population in the giant tubeworm trophosome (Nyholm et al. 2012; Hinzke et al. 2019). Our results, contrary to the expectations, indicate that genes involved with the innate immune responses are downregulated in the trophosome (e.g., Toll-like receptor/MyD88) or in adult tubeworm tissues (e.g., sushi). These results suggest that the innate immune system plays a more prominent role into the establishment of the symbiosis during the infection in the larval stage, rather than preservation of the mutualism during the juvenile/adult life cycle. The control of the endosymbiont population in the trophosome is mainly achieved by the upregulation of endosomal and lysosomal hydrolases resulting in the active digestion of the endosymbionts (a “mowing” process as described by Hinzke et al. 2019).

The giant tubeworm genome establishes a unique and unprecedent hallmark bridging more than four decades of physiological research in *Riftia*, whereas it simultaneously provides new insights into the development, whole organism function and evolution of one of the most studied models for metazoan–symbiont interaction. We envisage that the resources generated herein foster many hypothesis-driven research pointing toward a more complete understanding of the genotype/phenotype interface in the *Riftia* and closely related taxa.

## Materials and Methods

A detailed methods section is available in supplementary materials and methods and supplementary figure 61, Supplementary Material online. A brief overview of the bioinformatics pipeline follows below.

### Biological Material and Sequencing

*Riftia* genome DNA was obtained from a piece of vestimentum tissue belonging to single worm collected at the hydrothermal vent site Tica, East Pacific Rise (Alvin dive 4839, 9°50.398′N, 104°17.506′W, 2,514 m depth, 2016) (supplementary figs. 1 and 2, Supplementary Material online). PacBio libraries were generated with Sequel technology using the purified *Riftia* DNA. Tissue-specific transcriptomes were obtained from two specimens collected at Guaymas Basin, one female from the vent site Rebecca’s Roost (SuBastian dive 231, 27°0.645′N, 111°24.418′W, 2,012 m depth, 2019) and one male from a vent site close to Big Pagoda (SuBastian dive 233, 27°0.823′N, 111°24.663′W, 2,015 m depth, 2019) (supplementary figs. 1 and 2, Supplementary Material online). The eight-stranded paired-end tissue-specific transcriptomes (2×150 pb) were sequenced using Illumina NovaSeq SP technology.

### Genome Assembly and Processing

The five *Riftia* PacBio libraries were mapped against a custom database built with *Riftia* mitochondrial genome and its complete Endoriftia genome using minimap v2.17-r941 (Li 2018). Genome assembly was performed with canu v1.8 (Koren et al. 2017) with optimized parameters. Genome preprocessing, polishing, haplotig removal, and contamination screening were performed with arrow v2.3.3 (https://github.com/pacificbiosciences/genomicconsensus), purge_dups (https://github.com/dfguan/purge_dups), and blobtools v1.1.1 (Laetsch and Blaxter 2017), respectively. Mitochondrial and endosymbiont genome assemblies were executed with flye v2.5 (Kolmogorov et al. 2019). Annotation of the mitochondrial genome was performed with MITOS2 and GeSeq (Bernt et al. 2013; Tillich et al. 2017).

### Transcriptome Assembly and Processing

The removal of adapter sequences and quality filtering of reads from the raw transcriptome databases were performed with bbduk v38.42 (https://sourceforge.net/projects/bbmap/). De novo and genome-guided transcriptome assemblies were performed with transabyss v.2.0.1 and STAR v2.7.1a—Stringtie v2.0.6, respectively (Robertson et al. 2010; Dobin et al. 2013; Kovaka et al. 2019). Endoriftia contamination, if present, was removed from the transcriptomes using BlastN v2.8.1+ (Camacho et al. 2009). A global de novo transcriptome was generated with corset and Lace (https://github.com/Oshlack/Lace) (Davidson and Oshlack 2014).

### Gene Prediction and Annotation

The repeat landscape of *Riftia* genome was identified combining a custom giant tubeworm RepeatModeler v2.0 library followed by the masking of the repetitive elements with RepeatMasker v4.0.9 (Smit AFA, Hubley R, Green P, *RepeatMasker Open-4.0*). Ab initio gene prediction was performed with Augustus v3.3.3 aided with hint files (Stanke and Morgenstern 2005; Hoff and Stanke 2018). Only gene models with homology, orthology, and gene expression evidence were kept. Protein annotation was performed with Interproscan v5.39-77.0, RNAscan-SE 2.0.5, signalP v5.0b, and pfam_scan.pl (Jones et al. 2014; Lowe and Chan 2016; Almagro Armenteros et al. 2019).

### Identification of Gene Toolkits in *Riftia*

*Riftia* protein sequences were searched against well-curated catalog of developmental genes, amino and fatty acid biosynthesis, endocytosis-, apoptosis, autophagy-, and immune-related genes using BlastP v2.8.1+ and KEGG Automatic Annotation server (https://www.genome.jp/kegg/kaas/) (Moriya et al. 2007). Additionally, protein domain information was retrieved from pfam_scan.pl and Interpro results. Homology of the identified genes was confirmed through phylogenetic inferences using iqtree v1.6.11 combining ModelFinder, tree search, 1,000 ultra-fast bootstrap, and SH-aLRT test replicates (Nguyen et al. 2015; Kalyaanamoorthy et al. 2017; Hoang et al. 2018). The protein diagrams were drawn using IBS v.1.0.3 software (Liu et al. 2015), and the clustered heatmaps generated with the R package pheatmap (v1.0.12). Quantification of the gene expression levels was performed with kallisto v0.46.1 (Bray et al. 2016).

### Orthology, Gene Family Analysis, and Positively Selected Genes

To assess *Riftia*, *Lamellibrachia* and Annelida lineage-specific genes, orthology inferences using selected nonbilaterian, deuterostome, lophotrochozoan, ecdysozoans, representatives (*N* = 36) were performed with Orthofinder v2.3.8 (Emms and Kelly 2019). To identify statistically significant gene family expansions/contractions in *Riftia* compared with other lophotrochozoans, a second round of orthology was performed using 18 lophotrochozoan representatives and *Tribolium castaneum* as outgroup. Finally, to identify the gene family core within Annelida, a last instance of orthofinder v2.3.8 was invoked using the *C. teleta*, *H. robusta*, *L. luymesi*, and *R. pachyptila*. Only the longest isoform for each gene was used in the analysis. Nonsynonymous (Ka) and synonymous (Ks) substitution rates were calculated with the stand-alone version of KaKs_calculator v.2 and HyPhy v. 2.5.15 (Pond et al. 2005; Wang et al. 2010; Zhang, Xiao, et al. 2012). Only single-copy genes (1:1 orthologs) without any inconsistencies between the nucleotide and protein sequences were used in the analyses. Contracted and expanded gene families in the giant tubeworm genome were identified using CAFE v4.2.1 (De Bie et al. 2006; Han et al. 2013) using a calibrated starting tree produced by Phylobayes v4.1b (Lartillot et al. 2013). The contracted/expanded gene families were annotated with Interproscan v5.39-77.0 and the enrichment analysis for GO was performed with topGO v2.36.0 using Fisher’s exact test against the *R. pachyptila* background (i.e., complete set of *Riftia* genes) coupled with weight01 algorithm. Rapidly evolving gene families in *Riftia* were annotated using PANTHER HMM scoring tool v2.2 with PANTHER_hmmscore database v15 (Mi et al. 2017). Protein domain contractions and expansions were found using iterative two-tailed Fisher’s exact (supplementary file 2, Supplementary Material online) test applied to pfam_scan.pl results. The obtained *P* values were corrected using Benjamini and Hochberg method (Benjamini and Hochberg 1995) and only domains with a significant *P* value of <0.01 were further investigated.

### Hemoglobin Evolution

The predicted *Riftia* hemoglobin (Hb) protein sequences were interrogated for the presence of the globin domain (PF00042) with hmmalign v3.1b2 (Mistry et al. 2013) and proteins without a hit were excluded from the analyses. Manual inspection and characterization of the signature diagnostic residues/motifs in the hemoglobin chain and linker sequences were performed following previous works (Belato et al. 2019). Phylogenetic analyses were carried out as described in the section “Identification of Gene Toolkits in *Riftia.*” The resulting trees were midpoint rooted using Figtree (http://tree.bio.ed.ac.uk/software/figtree/). Additionally, to investigate the hemoglobin gene expression across different environmental conditions (sulfur rich, sulfur depleted, and medium), we downloaded six publicly available trophosome transcriptomes from SRA (https://www.ncbi.nlm.nih.gov/sra) (accession nos. SRR8949066–SRR8949071). The transcriptome libraries were preprocessed as described in the section “Transcriptome Assembly and Processing.” *Riftia* Hb sequence was modeled using the Prime program implemented in the Schrödinger Drug Discovery (v2020.2) software suite. All illustrations of structures were made with PyMol v2.4 (https://pymol.org/2/).

### Comparative Tissue-Specific Transcriptome

The *Riftia* transcriptome libraries were pseudoaligned against the merged filtered AUGUSTUS gene models with kallisto v.0.46.1 (Bray et al. 2016) to collect the gene expression data expressed as TPM counts (transcripts per million). Normalization within and across tissues was independently performed before calculating the tissue specificity tau values (see https://rdrr.io/github/roonysgalbi/tispec/f/vignettes/UserGuide.Rmd). To mitigate possible sex-specific differences in the gene expression levels, tau calculations were performed using only the tubeworm female tissues. The absolutely TSGs (genes expressed only in a single tissue defined by a tau value of 1) were submitted to enrichment analyses for GO with topGO as mentioned in the section “Orthology, Gene Family Analysis, and Positively Selected Genes.” 

## Supplementary Material

Supplementary data are available at *Molecular Biology and Evolution* online.

## Supplementary Material

msab347_Supplementary_Data
