# Supplementary material for: Novel Insights on Obligate Symbiont Lifestyle and Adaptation to Chemosynthetic Environment as Revealed by the Giant Tubeworm Genome
Source: Mol Biol Evol. 2021 Dec 6;39(1):msab347. doi: 10.1093/molbev/msab347 (PMC8789280; doi:10.1093/molbev/msab347)
Supplement: msab347_Supplementary_Data [file msab347_supplementary_data.zip › Supplementary_File1_revised.docx]

**Supplementary Information**

**Novel insights on obligate symbiont lifestyle and adaptation to chemosynthetic environment as revealed by the giant tubeworm genome**

1. André Luiz de Oliveira¹, Jessica Mitchell², Peter Girguis², Monika Bright¹

¹ Department of Functional and Evolutionary Ecology, University of Vienna, Austria

² Department of Organismic and Evolutionary Biology, Harvard University, Cambridge, MA, USA

**Supplementary information**

[**SUPPLEMENTARY FIGURES** 6](#_Toc87268796)

[**Supplementary figure 1 | Maps of *Riftia* sampling sites.** 6](#_Toc87268797)

[**Supplementary figure 2 | Vent sites.** 7](#_Toc87268798)

[**Supplementary figure 3 | Long read sequencing of *Riftia* genome.** 8](#_Toc87268799)

[**Supplementary Figure 4 | *Riftia* genome size.** 10](#_Toc87268800)

[**Supplementary Figure 5 | *Riftia* interspersed repeat landscape.** 11](#_Toc87268801)

[**Supplementary Figure 6 | Genome assembly assessment.** 12](#_Toc87268802)

[**Supplementary figure 7 | Mitochondrial genome** 13](#_Toc87268803)

[**Supplementary figure 8 | Phylogeny of Hox, ParaHox and Hox-like genes** 15](#_Toc87268804)

[**Supplementary figure 9 | Phylogeny of Hox and ParaHox genes** 18](#_Toc87268805)

[**Supplementary figure 10 | Expression of selected genes involved in the development of the digestive tract** 18](#_Toc87268806)

[**Supplementary figure 11 | Phylogeny of TGF-beta genes.** 20](#_Toc87268807)

[**Supplementary figure 12 | Phylogeny of Notch ligand and receptor genes** 20](#_Toc87268808)

[**Supplementary figure 13 | Phylogeny of Hedgehog ligand and receptor genes** 22](#_Toc87268809)

[**Supplementary figure 14 | Phylogeny of Wnt ligands** 24](#_Toc87268810)

[**Supplementary figure 15 | Phylogeny of Wnt receptor genes (Frizzled)** 25](#_Toc87268811)

[**Supplementary figure 16 | Shared orthogroups in Annelida** 26](#_Toc87268812)

[**Supplementary figure 17 | Gene gain and loss in selected lophotrochozoans.** 26](#_Toc87268813)

[**Supplementary figure 18 | Gene set enrichment analysis with topGO using CAFE contract families in *Riftia*** 28](#_Toc87268814)

[**Supplementary figure 19 | Distribution of transcription factors among lophotrochozoans** 30](#_Toc87268815)

[**Supplementary figure 20 | Gene set enrichment analysis with topGO using CAFE expanded families in *Riftia*** 31](#_Toc87268816)

[**Supplementary figure 21 | Distribution of *Riftia* expanded/contracted PFAM domains among selected lophotrochozoans.** 32](#_Toc87268817)

[**Supplementary figure 22 | Gene set enrichment analysis with topGO using *Riftia pachyptila* lineage-specific genes** 33](#_Toc87268818)

[**Supplementary figure 23 | Gene set enrichment analysis with topGO using *Lamellibrachia luymesi* lineage-specific genes** 36](#_Toc87268819)

[**Supplementary figure 24 | PFAM functional domains shared among selected deep vent lophotrochozoans and cluster analysis of selected *Riftia* expanded domains.** 37](#_Toc87268820)

[**Supplementary figure 25 | Gene set enrichment analysis with topGO using absolutely vestimentum specific TAU genes** 40](#_Toc87268821)

[**Supplementary figure 26 | Gene set enrichment analysis with topGO using absolutely body wall (skin) specific TAU genes** 42](#_Toc87268822)

[**Supplementary figure 27 | Phylogeny and genomic organisation of sushi genes in *Riftia pachyptila*** 43](#_Toc87268823)

[**Supplementary figure 28 | Phylogeny of Riftia and Lamellibrachia globin and linker genes** 46](#_Toc87268824)

[**Supplementary figure 29 | Multiple sequence alignments and gene expression of *Riftia* linker genes** 47](#_Toc87268825)

[**Supplementary figure 30| Homology model generation for Riftia haemoglobin** 48](#_Toc87268826)

[**Supplementary figure 31 | Multiple sequence alignment of *Riftia* and *Lamellibrachia* haemoglobin genes** 49](#_Toc87268827)

[**Supplementary figure 32 | Carbonic anhydrase genes in *Riftia pachyptila*** 50](#_Toc87268828)

[**Supplementary figure 33 | Gene set enrichment analysis with topGO using absolutely trophosome specific TAU genes** 53](#_Toc87268829)

[**Supplementary figure 34 | Phylogeny of cathepsins** 55](#_Toc87268830)

[**Supplementary figure 35 | Gene expression of cathepsins** 56](#_Toc87268831)

[**Supplementary figure 36 | Gene expression of lysosomal-associated hydrolases.** 58](#_Toc87268832)

[**Supplementary figure 37 | Phylogeny and gene expression of endosomal genes** 59](#_Toc87268833)

[**Supplementary figure 38 | Phylogeny and gene expression of SOD genes** 61](#_Toc87268834)

[**Supplementary figure 39 | Gene expression of enzymes related to amino acid biosynthesis** 63](#_Toc87268835)

[**Supplementary figure 40 | Gene expression of enzymes related to haem biosynthesis** 64](#_Toc87268836)

[**Supplementary figure 41 | Overview and gene expression of genes involved in the nitrogen metabolism in *Riftia*.** 65](#_Toc87268837)

[**Supplementary figure 42 | Gene expression of genes involved in the purine and pyrimidine pathways in *Riftia*.** 67](#_Toc87268838)

[**Supplementary figure 43 | Phylogeny of glutamine synthetase genes in selected metazoans.** 70](#_Toc87268839)

[**Supplementary figure 44 | Phylogeny and gene expression of taurocyamine kinase genes** 71](#_Toc87268840)

[**Supplementary figure 45 | Gene expression of genes involved in the ammonia assimilation cycle and polyamine pathway in *Riftia*.** 73](#_Toc87268841)

[**Supplementary figure 46 – Cell cycle pathway and gene expression of cyclins and cyclin-dependent kinase genes.** 75](#_Toc87268842)

[**Supplementary figure 47 | Overview of Toll-like receptor/MyD88 immune system pathway in *Riftia.*** 76](#_Toc87268843)

[**Supplementary figure 48 | Toll-like gene domain composition and gene expression**. 78](#_Toc87268844)

[**Supplementary figure 49 - Phylogeny of Toll-like genes.** 80](#_Toc87268845)

[**Supplementary figure 50 | Caspase and paracaspase domain composition and gene expression.** 81](#_Toc87268846)

[**Supplementary figure 51 | Phylogeny of caspases and paracaspases** 83](#_Toc87268847)

[**Supplementary figure 52 | Gene expression of DED and BCL2 domain -containing proteins.** 85](#_Toc87268848)

[**Supplementary figure 53 | Phylogeny and gene expression of TNF ligands** 86](#_Toc87268849)

[**Supplementary figure 54 | Phylogeny and gene expression of TNF receptors.** 88](#_Toc87268850)

[**Supplementary figure 55 | Domain composition of IAP genes** 91](#_Toc87268851)

[**Supplementary figure 56 | Gene expression of IAP domain-containing proteins** 91](#_Toc87268852)

[**Supplementary figure 57 | Phylogeny of IAP genes** 92](#_Toc87268853)

[**Supplementary figure 58 | Overview of autophagy pathway in *Riftia* and gene expression of autophagy-related genes.** 96](#_Toc87268854)

[**Supplementary figure 59 | Gene set enrichment analysis with topGO using absolutely plume specific TAU genes** 98](#_Toc87268855)

[**Supplementary figure 60 | Gene set enrichment analysis with topGO using absolutely gonad specific TAU genes** 100](#_Toc87268856)

[**Supplementary figure 61 | Bioinformatic workflow used in this study** 101](#_Toc87268857)

[**SUPPLEMENTARY NOTES** 103](#_Toc87268858)

[**Supplementary note 1 | *Riftia* genome and transcriptome sequencing, assembling and assessment** 103](#_Toc87268859)

[**Supplementary note 2 | Developmental genes and signalling molecules** 105](#_Toc87268860)

[**Supplementary note 3 | Orthology and gene family analyses** 107](#_Toc87268861)

[**Supplementary note 4 | Haemoglobin evolution** 113](#_Toc87268862)

[**Supplementary note 5 | Comparative tissue-specific transcriptomics and gene expression** 116](#_Toc87268863)

[**Supplementary note 6 | Nitrogen metabolism and excretion** 127](#_Toc87268864)

[**Supplementary note 7 | Cell proliferation, innate immune system, apoptosis, and autophagy** 132](#_Toc87268865)

[**SUPPLEMENTARY MATERIAL AND METHODS** 140](#_Toc87268866)

[**1 GENOME SEQUENCING AND ASSEMBLY** 140](#_Toc87268867)

[**1.1 Sample collection, genomic DNA extraction and Sequencing Strategy** 140](#_Toc87268868)

[**1.2 Genome pre-processing** 140](#_Toc87268869)

[**1.3 Genome assemblies** 140](#_Toc87268870)

[**1.4 Genome post-processing** 141](#_Toc87268871)

[**1.4.1 Polishing** 141](#_Toc87268872)

[**1.4.2 Purging haplotigs and contig overlaps** 141](#_Toc87268873)

[**1.4.3 Contamination screening with blobtools** 141](#_Toc87268874)

[**1.5 Mitochondrial genome assembly and annotation** 142](#_Toc87268875)

[**2 TRANSCRIPTOME SEQUENCING AND ASSEMBLY** 142](#_Toc87268876)

[**2.1 Sample collection, total RNA extraction and Sequencing Strategy** 142](#_Toc87268877)

[**2.3 Transcriptome assembly** 143](#_Toc87268878)

[**2.3.1 *De novo* transcriptome assembly** 143](#_Toc87268879)

[**2.3.2 Reference-based assembly** 143](#_Toc87268880)

[**2.4 *De novo* transcriptome post-processing** 143](#_Toc87268881)

[**2.4.1 Removal of endosymbiont contamination** 143](#_Toc87268882)

[**2.4.2 Generating a *de novo* global non-redundant transcriptome** 144](#_Toc87268883)

[**2.5 Prediction of the coding sequence regions** 144](#_Toc87268884)

[**3 GENOME ANNOTATION** 144](#_Toc87268885)

[**3.1 Identification of interspersed repetitive regions and low complexity DNA** 144](#_Toc87268886)

[**3.2 *Ab initio* gene predictions** 145](#_Toc87268887)

[**3.3 Filtering AUGUSTUS gene model predictions** 145](#_Toc87268888)

[**3.4 Protein annotation** 145](#_Toc87268889)

[**3.5 Protein family analyses (PFAM)** 146](#_Toc87268890)

[**3.6 *Riftia* gene toolkits essential for development, homeostasis, and body patterning** 146](#_Toc87268891)

[**3.6.1 Antennapedia class** 146](#_Toc87268892)

[**3.6.2 Transcription factors** 146](#_Toc87268893)

[**3.6.3 Signalling, amino/fatty acid biosynthesis, endocytosis-, autophagy-, apoptosis- and immune-related gene toolkits** 146](#_Toc87268894)

[**3.6.4 Haemoglobin gene identification, characterisation, phylogenetic inferences and gene expression quantification** 147](#_Toc87268895)

[**3.6.5 Homology model generation of *Riftia* Hb** 147](#_Toc87268896)

[**4 COMPARATIVE GENOMICS AND GENE FAMILIES ANALYSES** 148](#_Toc87268897)

[**4.1 Orthology** 148](#_Toc87268898)

[**4.2 Gene family expansions/contractions with CAFE** 148](#_Toc87268899)

[**4.3 Gene family expansion/contraction with PFAM** 149](#_Toc87268900)

[**4.4 Clustering of *Riftia* expanded PFAM-containing genes with clans** 149](#_Toc87268901)

[**4.5 Synonymous and non-synonymous substitution rates analyses** 150](#_Toc87268902)

[**5 GENE EXPRESSION ANALYSES** 150](#_Toc87268903)

[**5.1 Gene expression quantification and identification of absolutely tissue specific genes** 150](#_Toc87268904)

[**5.2 Gene set enrichment analyses** 150](#_Toc87268905)

[**REFERENCES** 150](#_Toc87268906)

[**LIST OF BIOINFORMATIC COMMANDS** 170](#_Toc87268907)

# **SUPPLEMENTARY FIGURES**

**
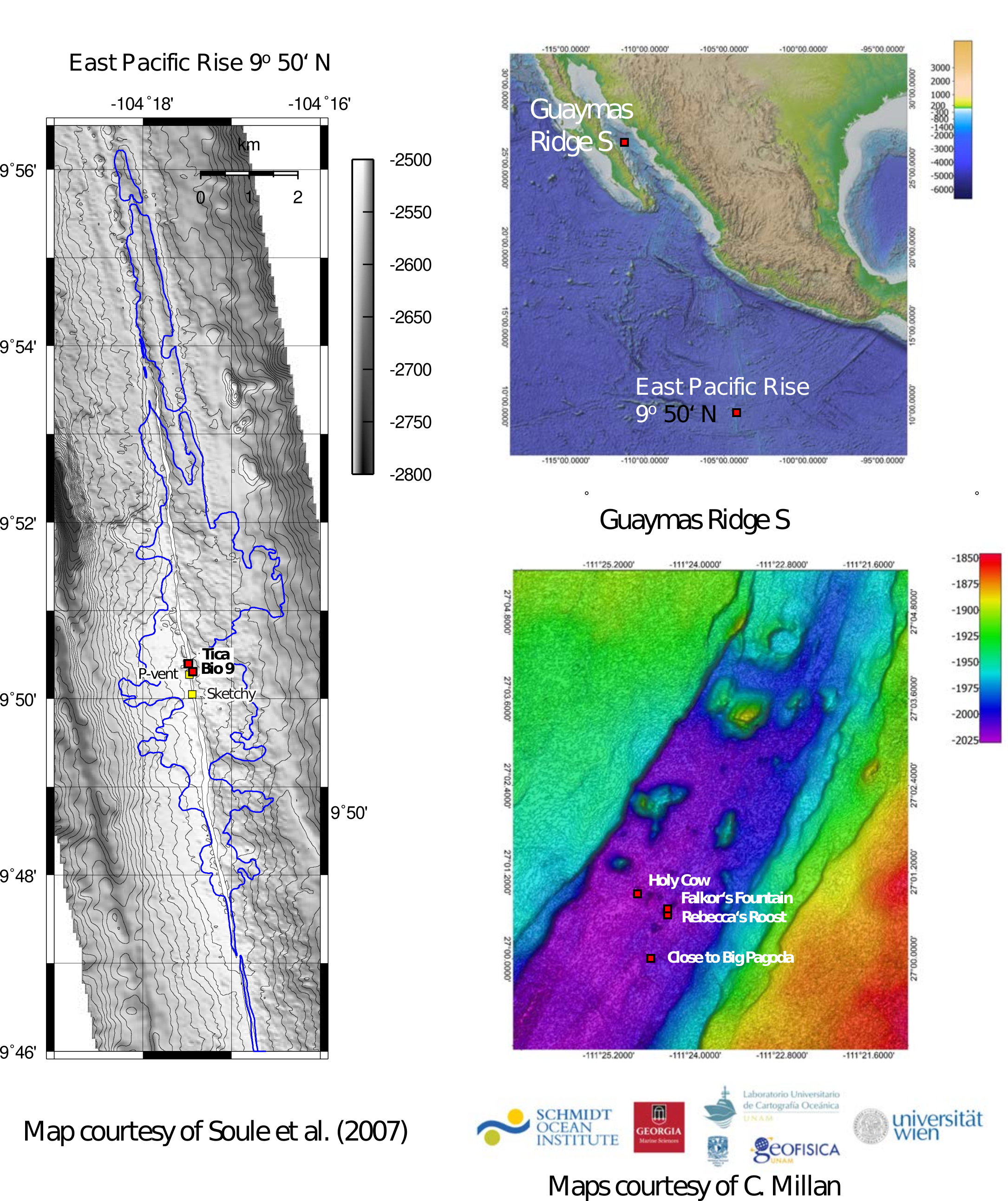
****Supplementary figure 1 | Maps of *Riftia* sampling sites.** The *Riftia* genome samples were collected from Tica vent site, located along the East Pacific Rise at ~9.50°N; *Riftia* transcriptomic samples came from vent sites Rebecca’s Roost and Big Pagoda, Guaymas Basin.


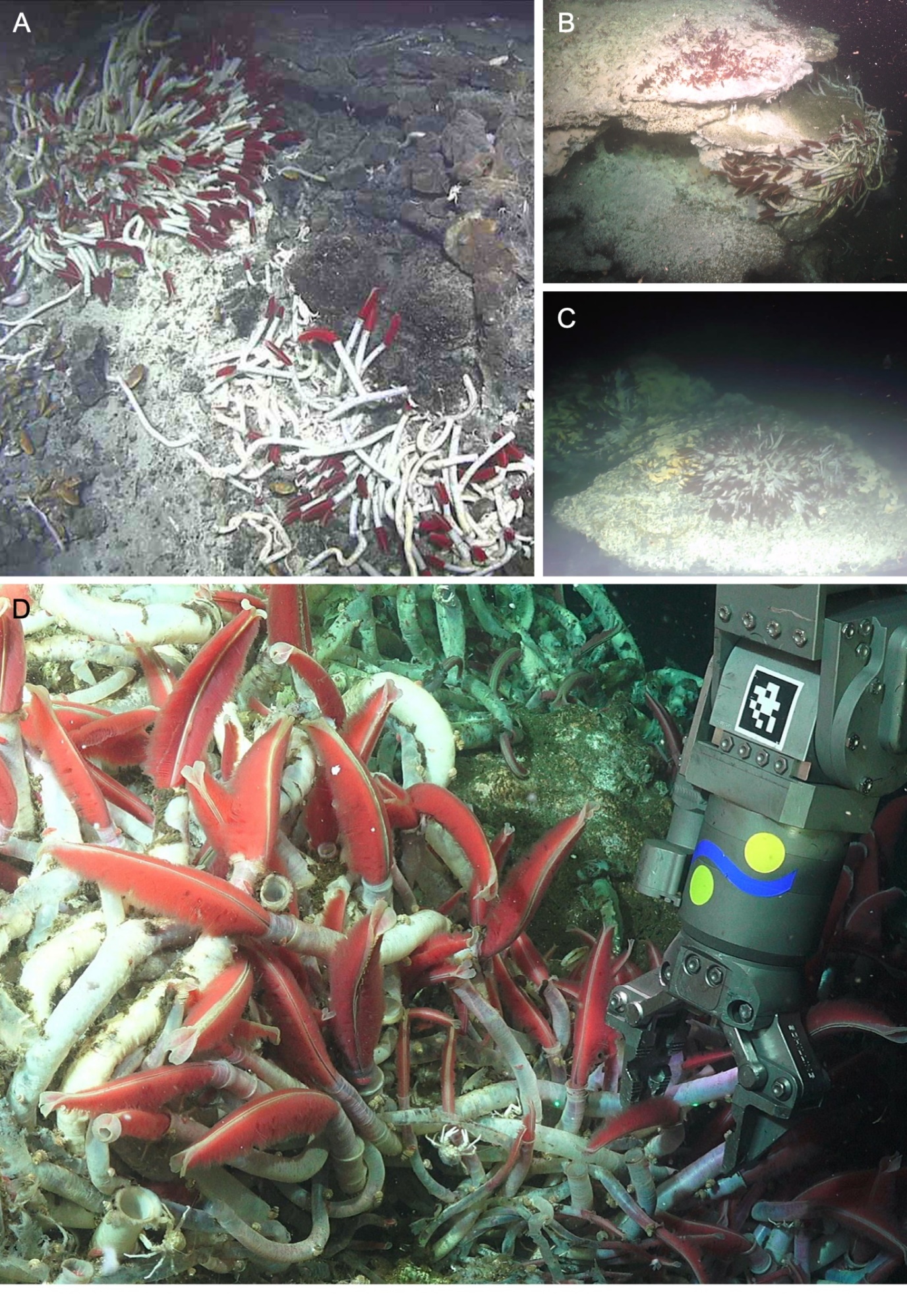


**Supplementary figure 2 | Vent sites. A** the Tica vent site, East Pacific Rise (*HOV Alvin* dive #4839, 2016). **B** Rebecca’s Roost vent site, Guaymas Basin, 2019 (*ROV SuBastian* dive #231, 2019). **C** and **D** sites close to Big Pagoda, Guaymas Basin, 2019 (*ROV SuBastian* dive #233, 2019).


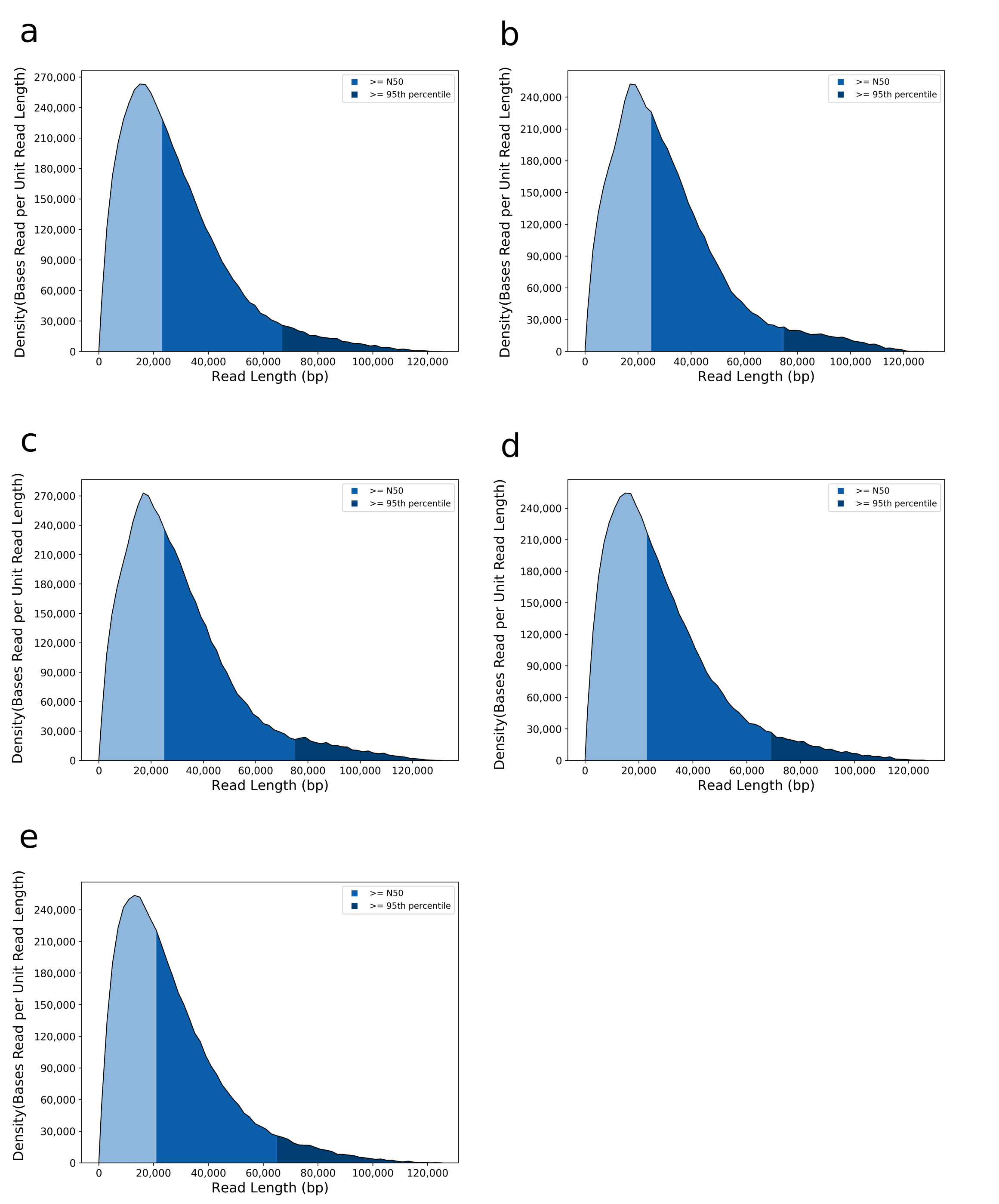


**Supplementary figure 3 | Long read sequencing of *Riftia* genome. a-e,** Pacific Biosciences™ Sequel sequencing performance of the five *Riftia* genomic libraries. The density (number of sequencing bases per read length) distribution is shown for each of the five libraries. Shades of blue indicate the number of sequenced bases present in reads smaller (light blue) and longer (blue, dark blue) than the N50 read value. The dark blue area corresponds the top 5% of the sequenced bases present in the longest read lengths. Note that half of the sequenced bases in all libraries are present in reads > 20kb.


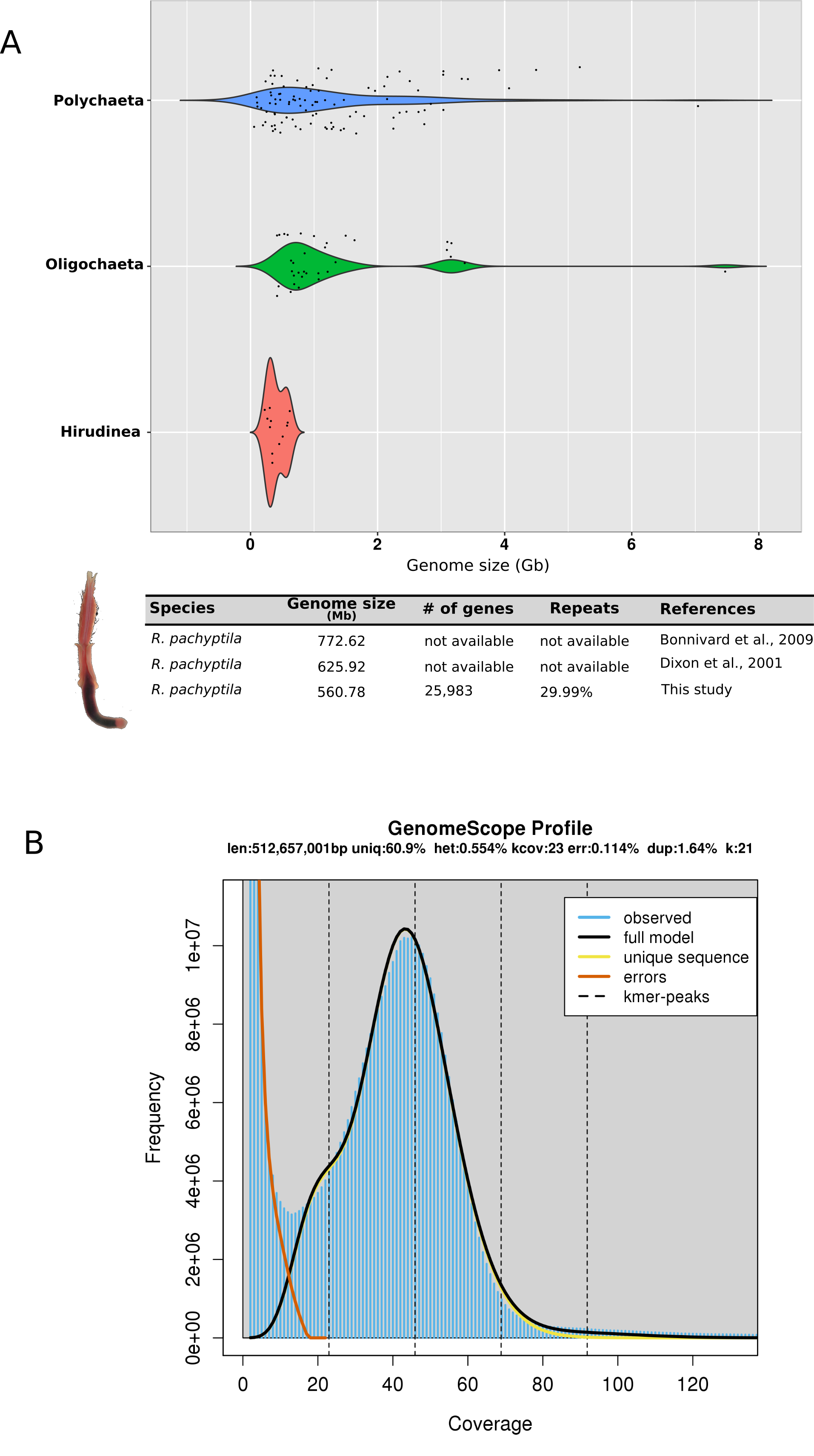


**Supplementary Figure 4 | *Riftia* genome size.** **A**, Violin plots of annelid genomes obtained from the Genome Size Database (<http://www.genomesize.com/>). Haploid DNA contents (C-values, in picograms) were converted to base pairs following Dolezel formula (Doležel et al., 2003). Kernel probability density values for the different annelid class-level taxa are shown (width of the plot). Individual data points, i.e., annelid genome sizes, are represented by black dots randomly scattered over the graph. The violin plots depict the range of the genome sizes (horizontal axis) and their frequencies (vertical axis) in the different annelid class-level taxa. B, GenomeScope estimations of genome heterozygosity, repeat content and size based on Canu (Koren et al. 2017) output results using a kmer-based statistical approach (<http://qb.cshl.edu/genomescope/>). The *Riftia* genome size estimation from the current study is markedly smaller than from the previously published studies.


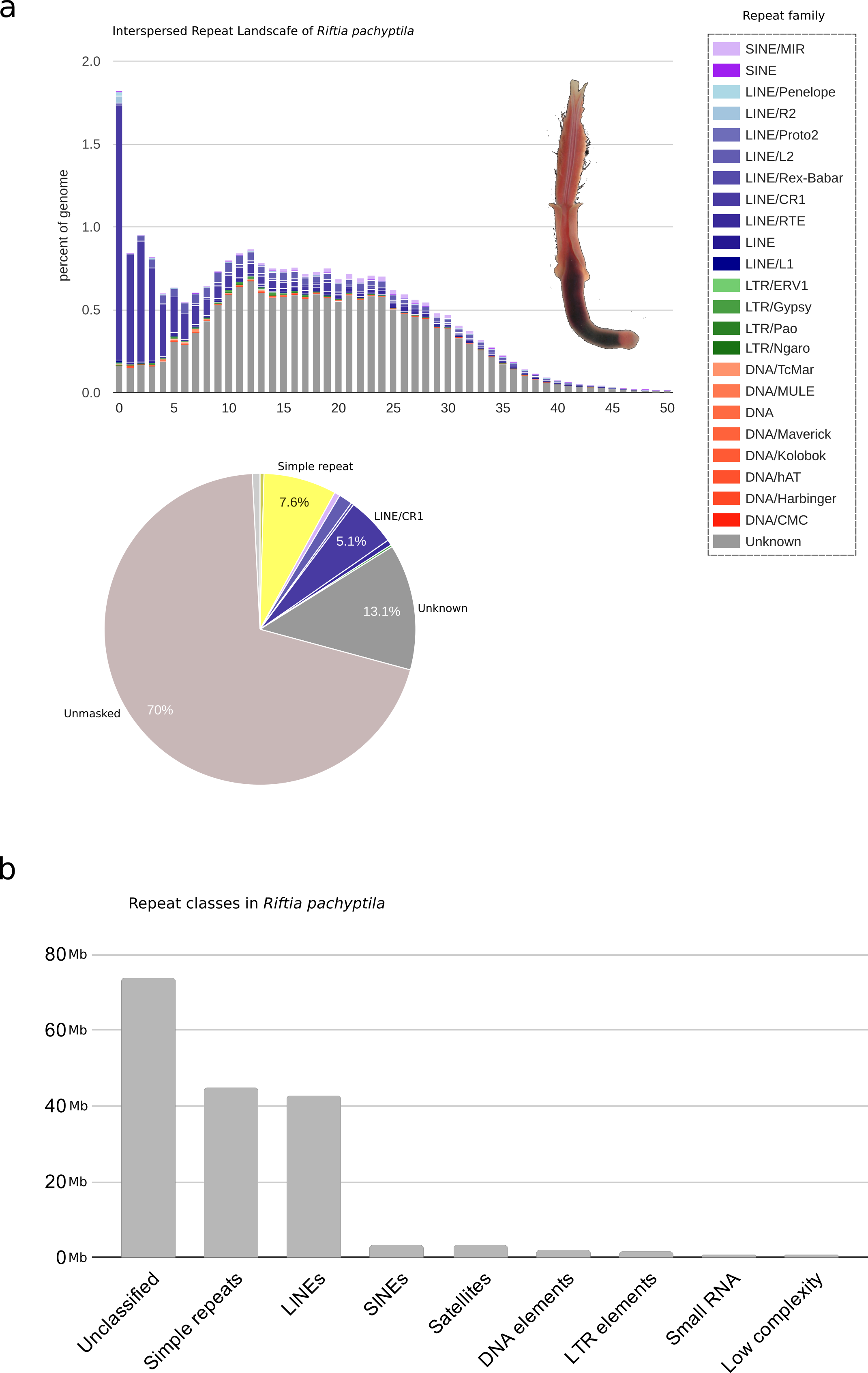


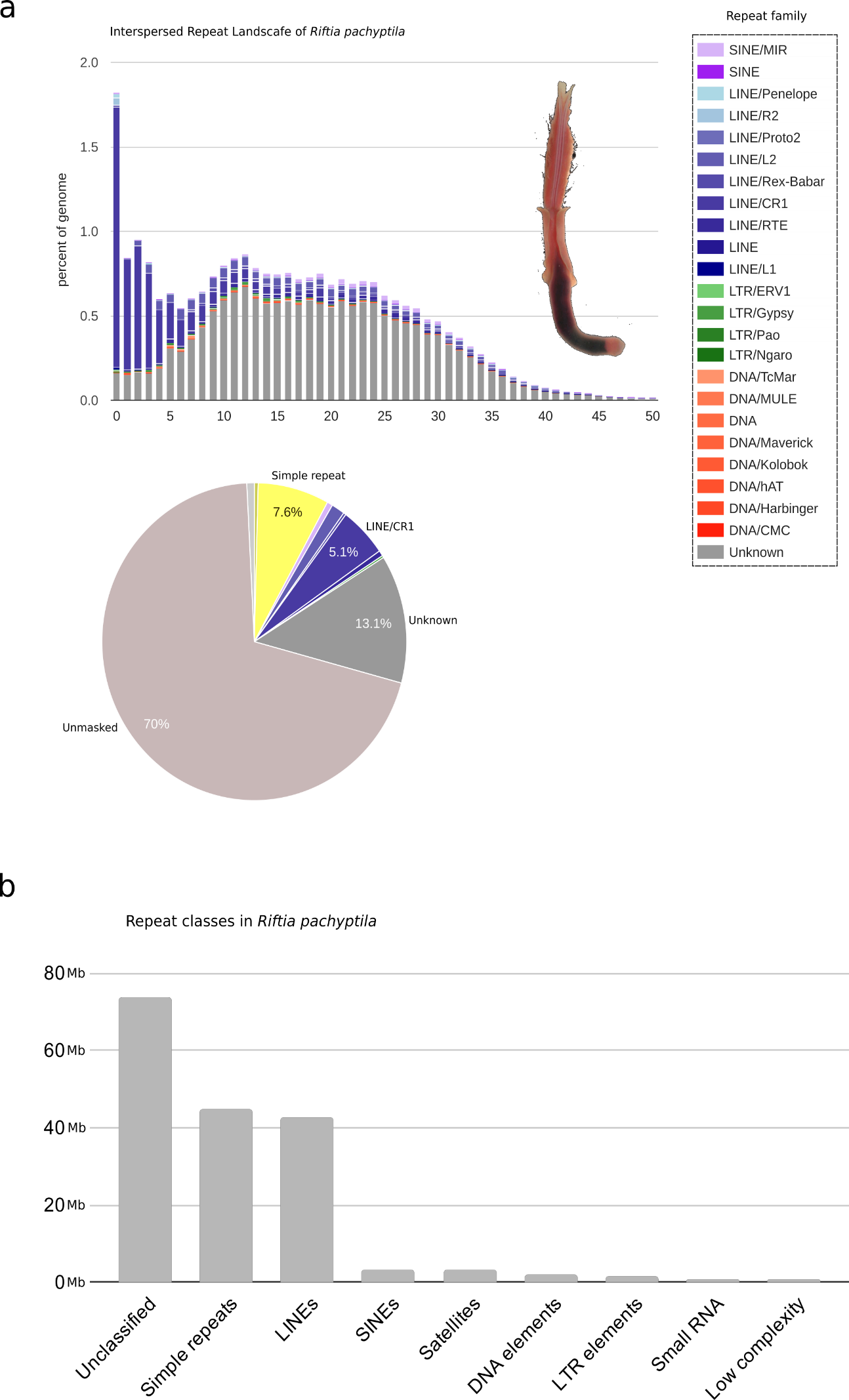


**Supplementary Figure 5 | *Riftia* interspersed repeat landscape. A,** The interspersed repeat landscape of *Riftia* *pachyptila* based on Kimura distances. The percentage of transposable elements in the tubeworm genome (y-axis) are clustered based on the Kimura values (x-axis). The pie chart shows the fraction of the *Riftia* genome covered by the different repeat families. Note that the repeat landscape is dominated by unclassified lineage-specific elements (i.e., unknown class). **B,** The number of repeat elements (in megabases) assigned to the main repeat classes in *Riftia.*


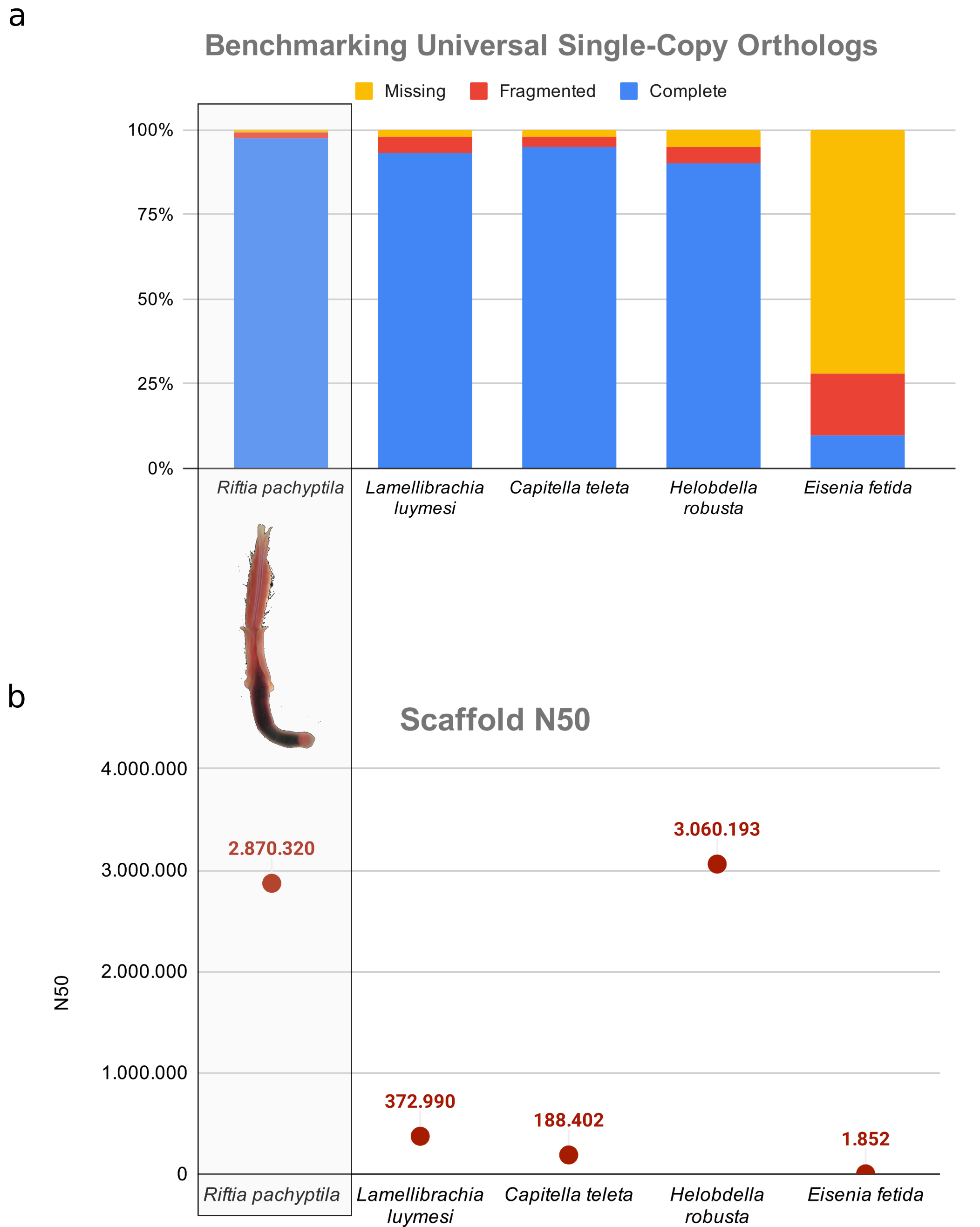


**Supplementary Figure 6 | Genome assembly assessment. A,** Metazoan BUSCO4 genome assembly scores of *Riftia pachyptila* and other four publicly available annelid genomes (Simakov et al. 2013; Paul et al. 2018; Li et al. 2019). **B,** scaffold N_50_ values of *Riftia* and four other publicly available annelid genomes. The giant tubeworm genome presents the most complete genome up to date. The scaffold N_50_ length of *Riftia* is similar to the leech *Helobdella robusta*.


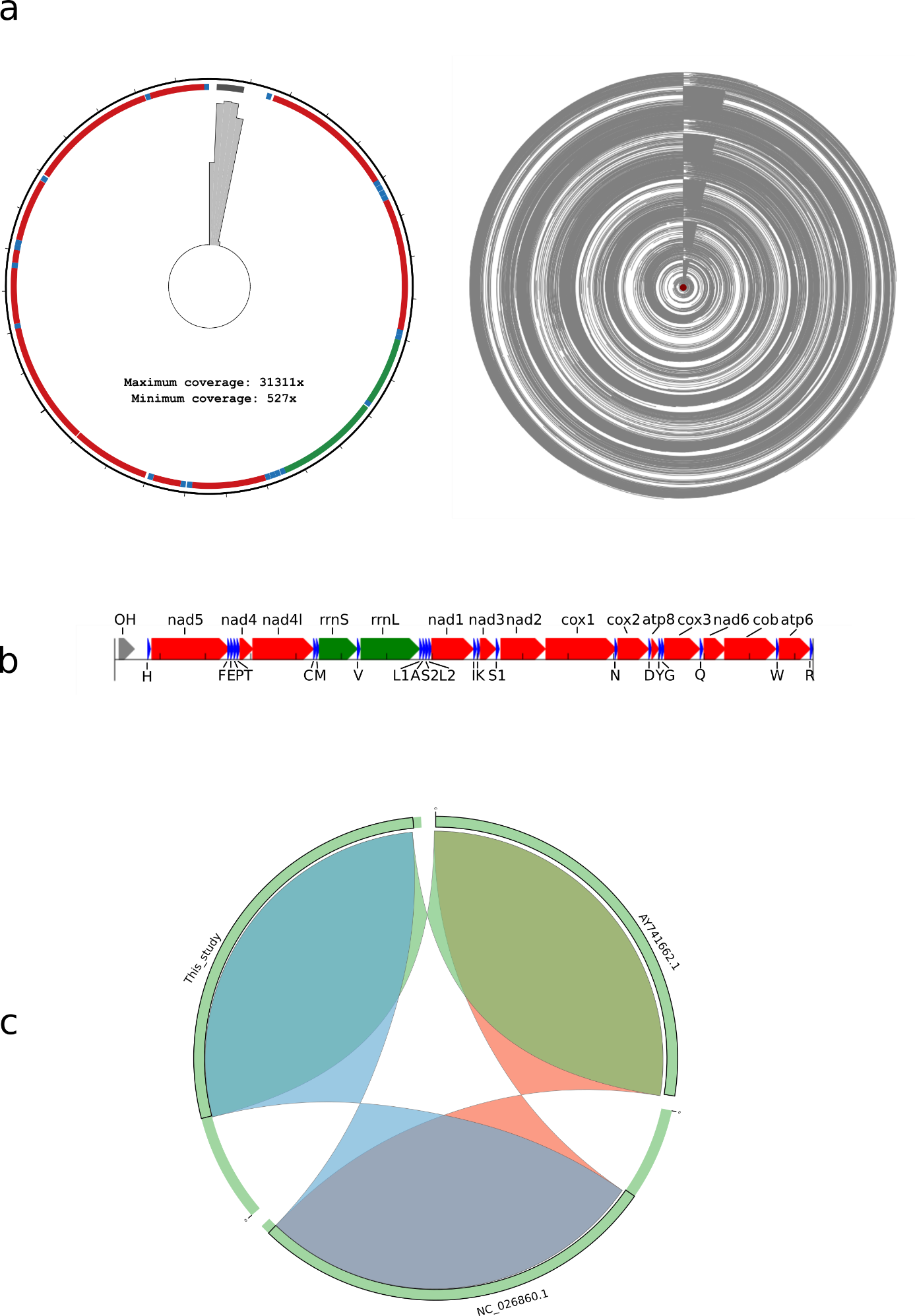


**Supplementary figure 7 | Mitochondrial genome**. **A**, Coverage plots of the PacBio long reads across the mitochondrial genome produced by circos and ConcatMap tools (<https://github.com/darylgohl/ConcatMap>). A total of 43,009 long reads mapped against the closed *Riftia* mitogenome with coverage levels ranging from 31,311x (matching the repetitive D-loop region) to 527x. **B**, Linear representation of the *R. pachyptila* mitochondrial genome showing the 13 coding sequencing regions, 2 rRNA and 22 tRNA genes. **C**, Large scale synteny blocks among the closed giant tubeworm mitochondrial genome provided in this study and other two publicly available references (AY741662 and NC_026860). The three mitochondrial reference genomes are virtually identical (>99% identity) with most of the mismatches located in the control region (D-loop). No rearrangements nor break of synteny were identified.

**
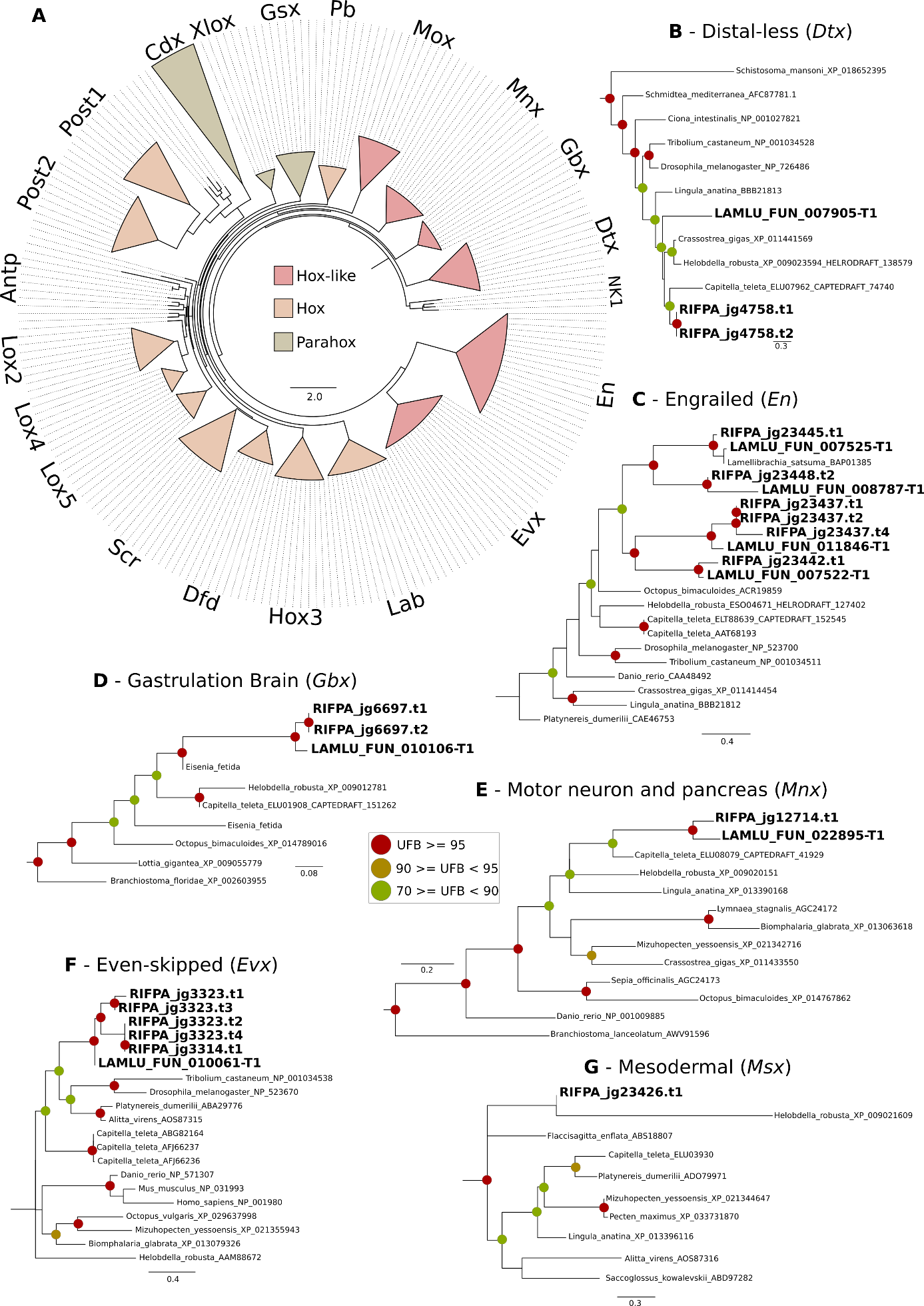
****Supplementary figure 8 | Phylogeny of Hox, ParaHox and Hox-like genes**. **A,** Maximum-likelihood phylogenetic tree inference of the homebox genes belonging to Hox, ParaHox and Hox-like families. The different ANTP genes were collapsed for an easier visualization. The different colours represent Hox, ParaHox and Hox-like gene members. NK1 genes were used as outgroup. **B-G,** Expanded tree inferences of Hox-like ANTP genes. The branch support values are represented by the coloured circles in the tree nodes. Red circles represent ultrafast bootstrap values >= 95. Yellow circles represent ultrafast bootstrap values >= 90 and < 95. Green circles represent ultrafast bootstrap values < 90 and >= 70. Ultrafast bootstrap values smaller than 70 are not shown. Accession numbers for NCBI database are displayed after the species names.

**
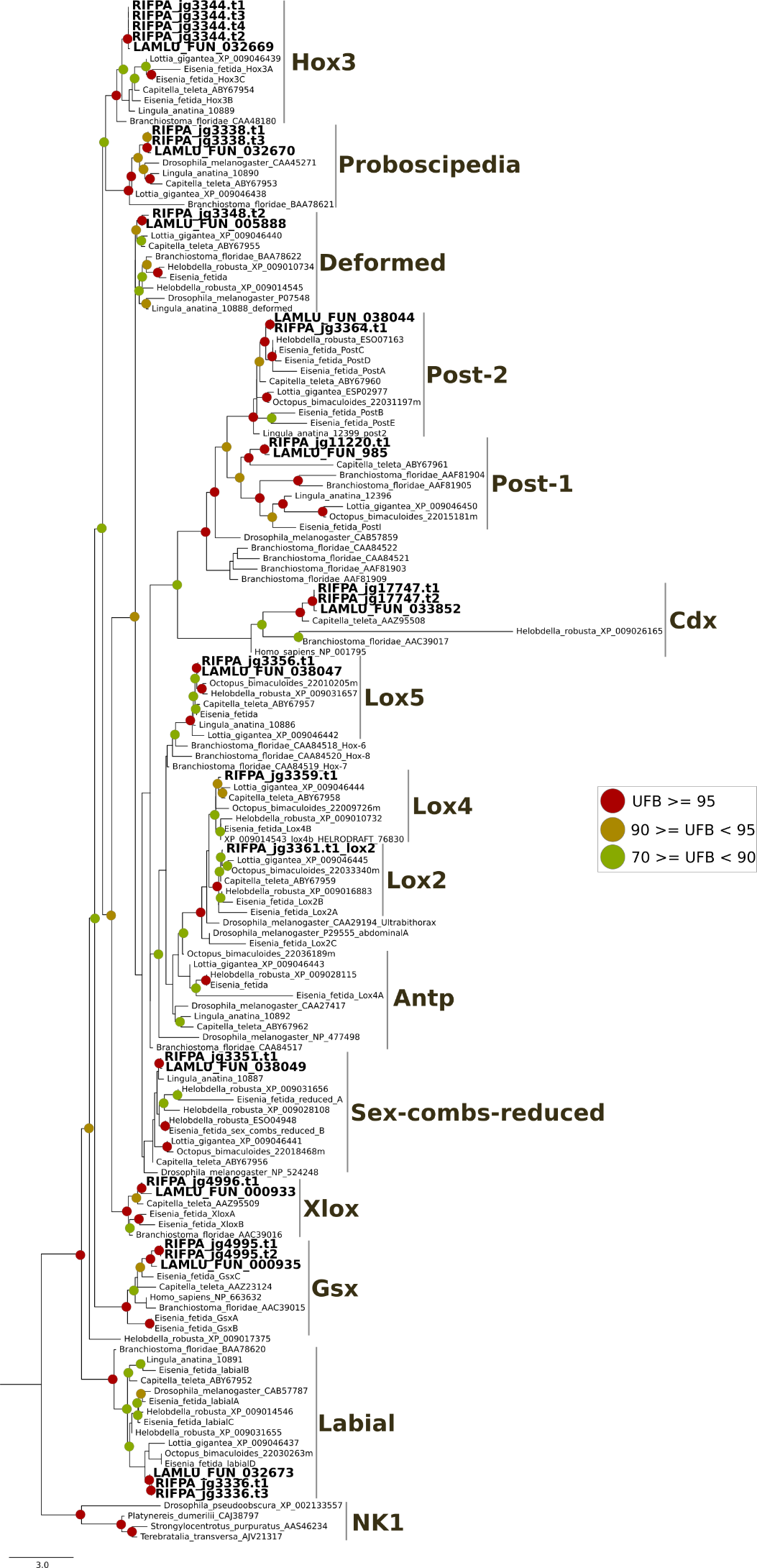
**

**Supplementary figure 9 | Phylogeny of Hox and ParaHox genes**. Maximum-likelihood phylogenetic tree inference of the Hox and ParaHox genes using 1000 ultrafast bootstrap replicates. The branch support values are represented by the coloured circles in the tree nodes. Red circles represent ultrafast bootstrap values >= 95. Yellow circles represent ultrafast bootstrap values >= 90 and < 95. Green circles represent ultrafast bootstrap values < 90 and >= 70. Ultrafast bootstrap values smaller than 70 are not shown. *Riftia pachyptila* contains 10 out of the 11 lophotrochozoan Hox genes (*Hox7* is missing) and all the three ParaHox (*Cdx*, *Xlox* and *Gsx*). NK1 genes were used as outgroup. Accession numbers for NCBI database are displayed after the species names.

**
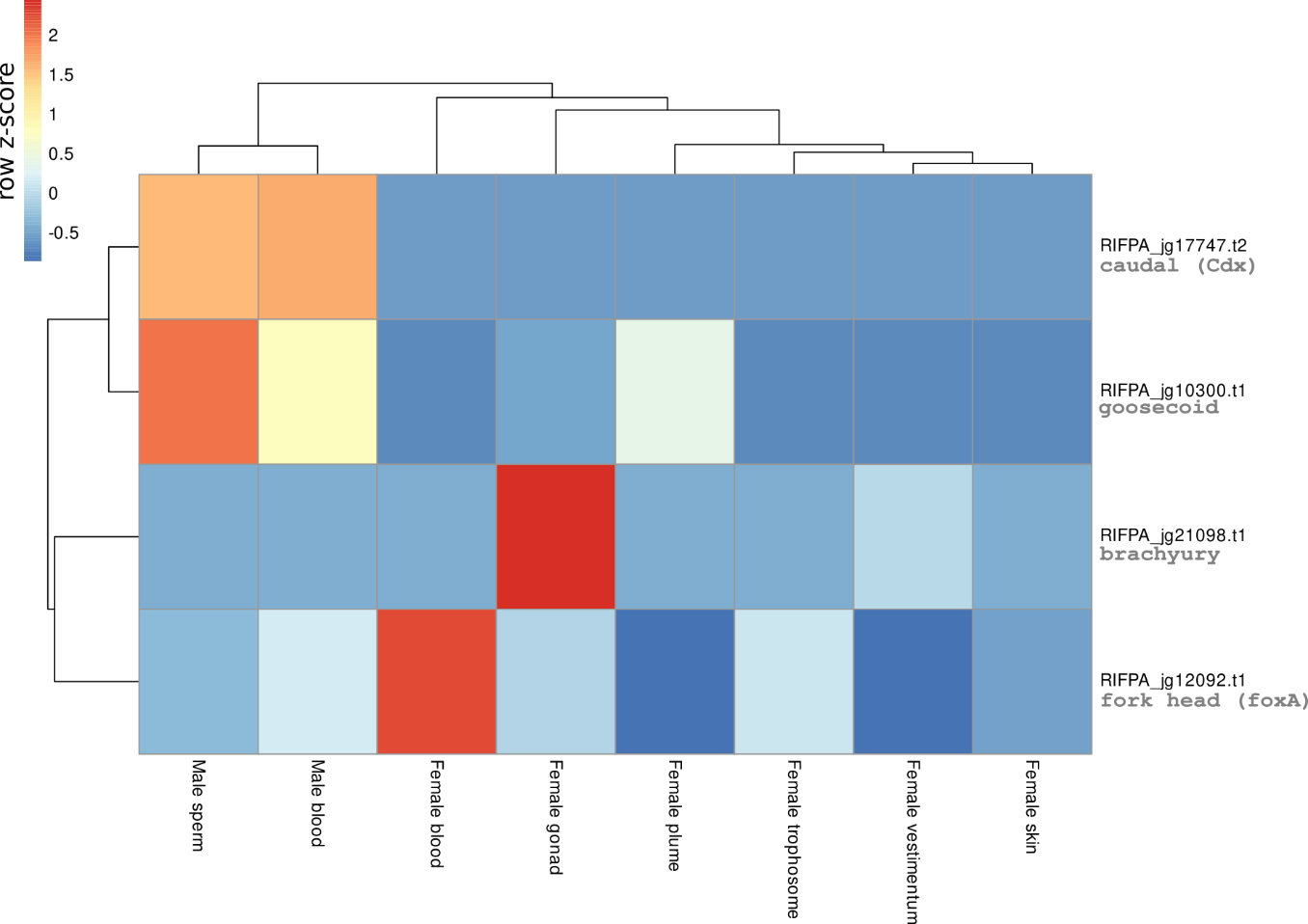
**

**Supplementary figure 10 | Expression of selected genes involved in the development of the digestive tract.** Expression profile of select genes involved in the development of the digestive tract in *Riftia pachyptila*. Colour coding reflects the expression patterns based on row Z-score calculations. The Parahox genes *Xlox* and *Gsx*, responsible for the patterning of the digestive tract in metazoans, did not show any expression on the eight adult *Riftia* tissues. All developmental genes involved in the digestive tract patterning are lowly expressed in the adult tissues.

**
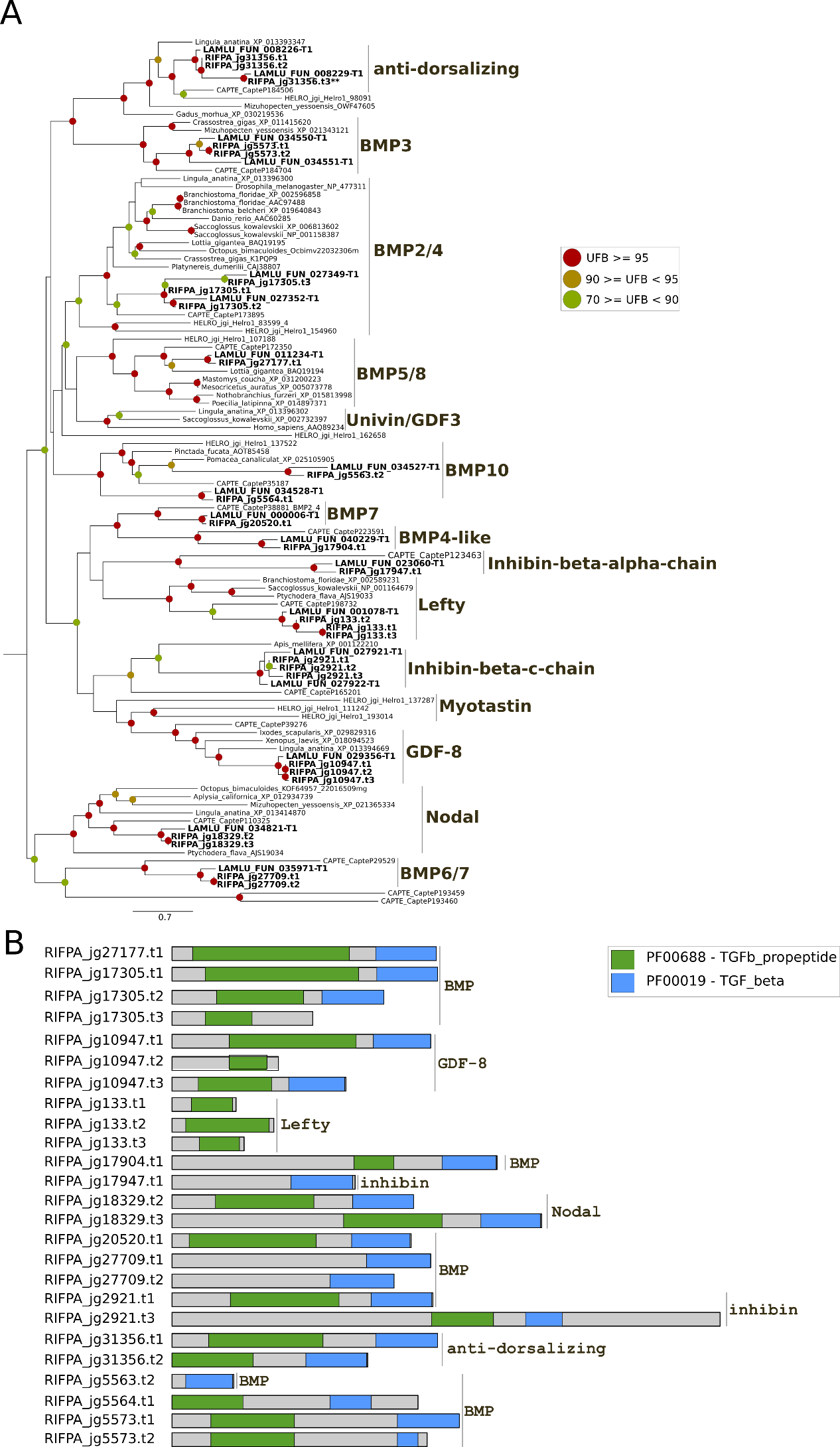
**

**Supplementary figure 11 | Phylogeny of TGF-beta genes. A,** Mid-rooted maximum-likelihood phylogenetic tree inference of the TGF-beta genes using 1000 ultrafast bootstrap replicates. The branch support values are represented by the coloured circles in the tree nodes. Red circles represent ultrafast bootstrap values >= 95. Yellow circles represent ultrafast bootstrap values >= 90 and < 95. Green circles represent ultrafast bootstrap values < 90 and >= 70. Ultrafast bootstrap values smaller than 70 are not shown. **B,** Domain composition of TGF-beta ligands based on PFAM database. Note that *lefty* gene does not contain the typical TGF_beta domain characteristic of the TGF-beta family.** no TGFb_propeptide and TGF_beta protein domains could be identified in this protein. Accession numbers for NCBI database are displayed after the species names. *Capitella, Helobdella* and *Lamellibrachia* gene identification are derived from the publicly available annotated genomes.

**
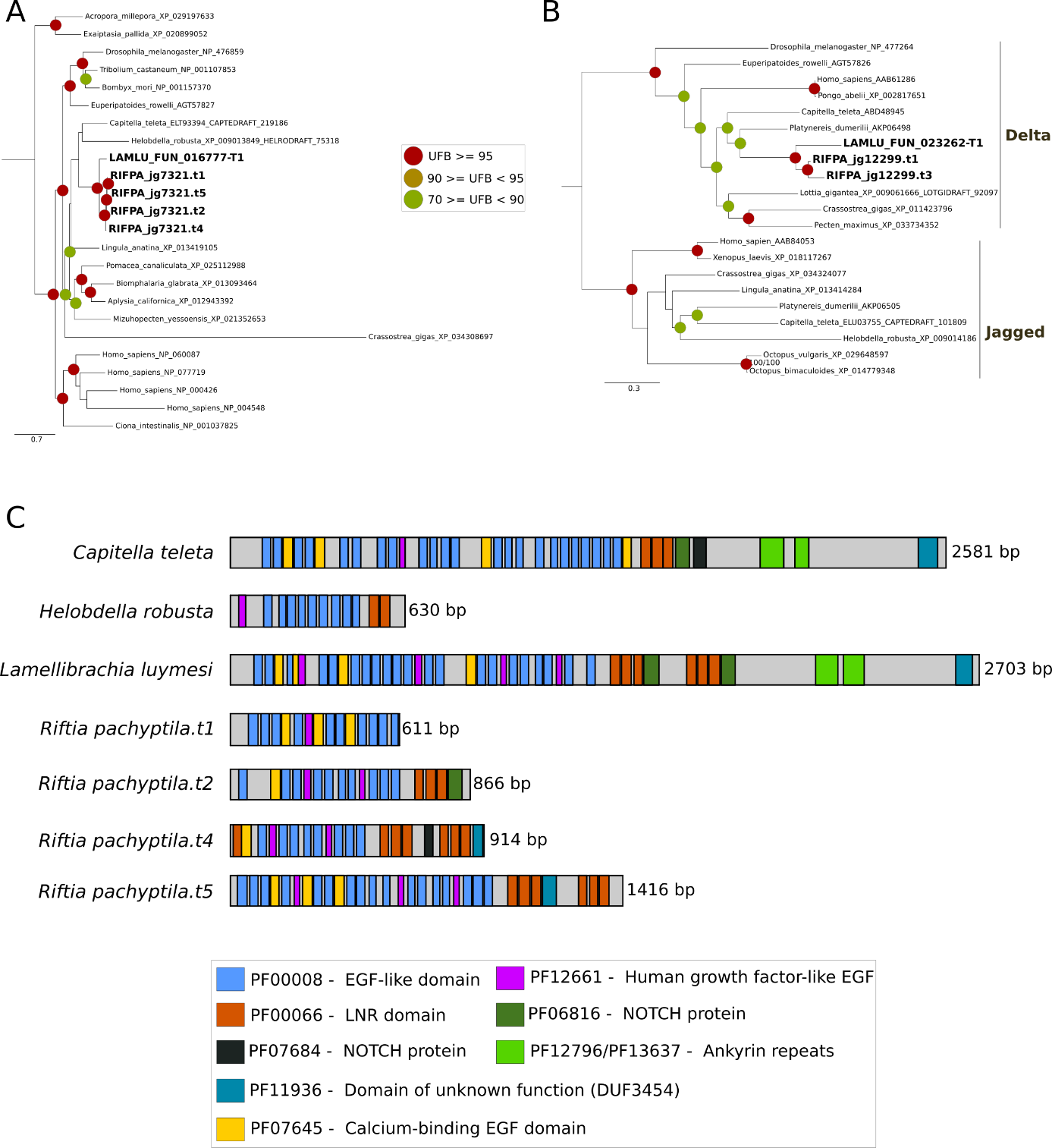
**

**Supplementary figure 12 | Phylogeny of Notch ligand and receptor genes. A-B,** Mid-rooted maximum-likelihood phylogenetic tree inference of the Notch ligand and receptor genes using 1000 ultrafast bootstrap replicates. The branch support values are represented by the coloured circles in the tree nodes. Red circles represent ultrafast bootstrap values >= 95. Yellow circles represent ultrafast bootstrap values >= 90 and < 95. Green circles represent ultrafast bootstrap values < 90 and >= 70. Ultrafast bootstrap values smaller than 70 are not shown. **B,** Domain composition of Notch ligands based on PFAM database. Accession numbers for NCBI database are displayed after the species names. *Capitella, Helobdella* and *Lamellibrachia* gene identification are derived from the publicly available annotated genomes.

**
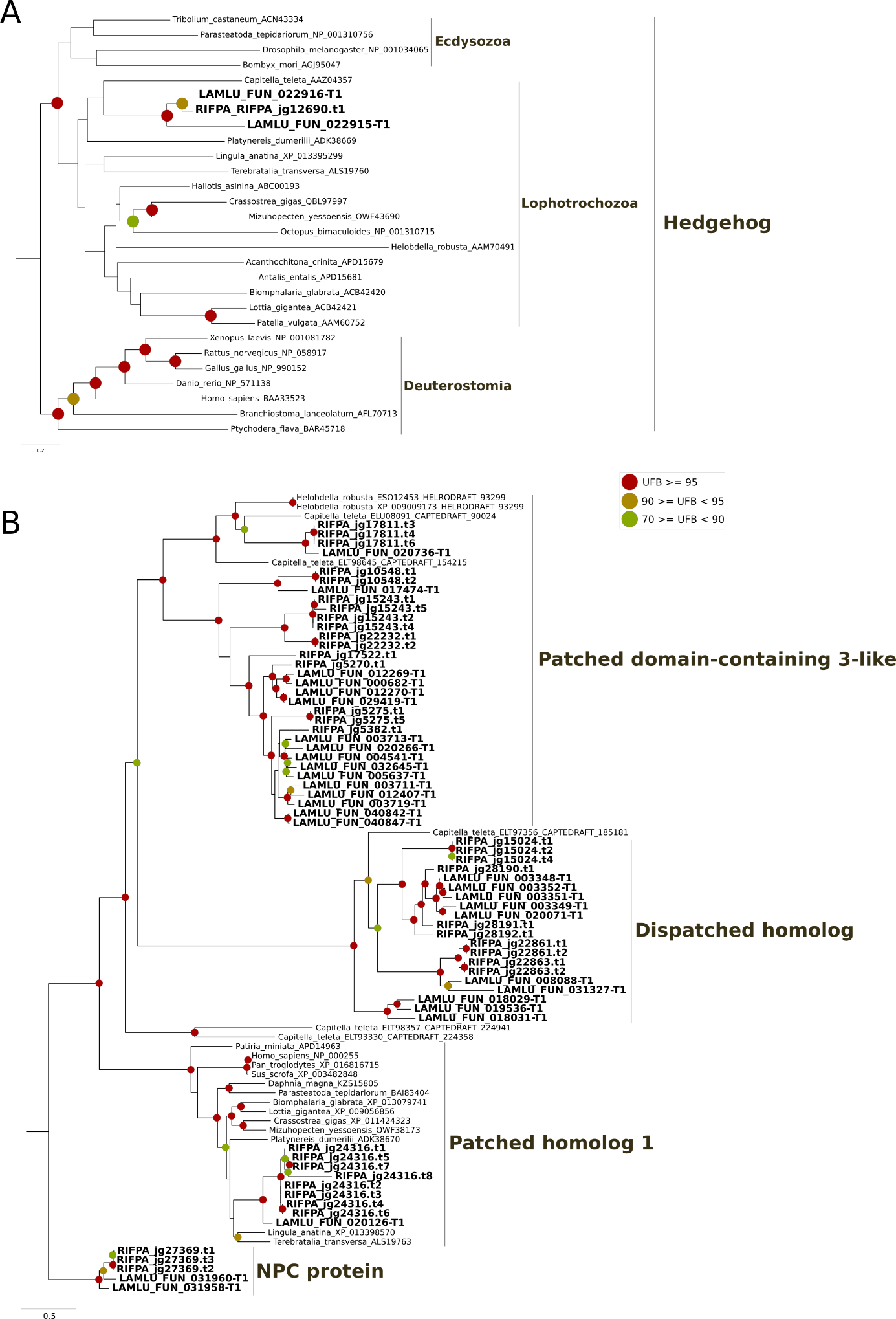
**

**Supplementary figure 13 | Phylogeny of Hedgehog ligand and receptor genes. A-B,** Maximum-likelihood phylogenetic tree inference of the Hedgehog ligand and receptor genes (*patched* and *dispatched*) using 1000 ultrafast bootstrap replicates. Dispatched receptors are expanded in the two tubeworm genomes. The branch support values are represented by the coloured circles in the tree nodes. Red circles represent ultrafast bootstrap values >= 95. Yellow circles represent ultrafast bootstrap values >= 90 and < 95. Green circles represent ultrafast bootstrap values < 90 and >= 70. Ultrafast bootstrap values smaller than 70 are not shown. Tubeworm Niemann-Pick C (NPC) proteins were used as outgroup. Accession numbers for NCBI database are displayed after the species names. *Capitella, Helobdella* and *Lamellibrachia* gene identification are derived from the publicly available annotated genomes.

**
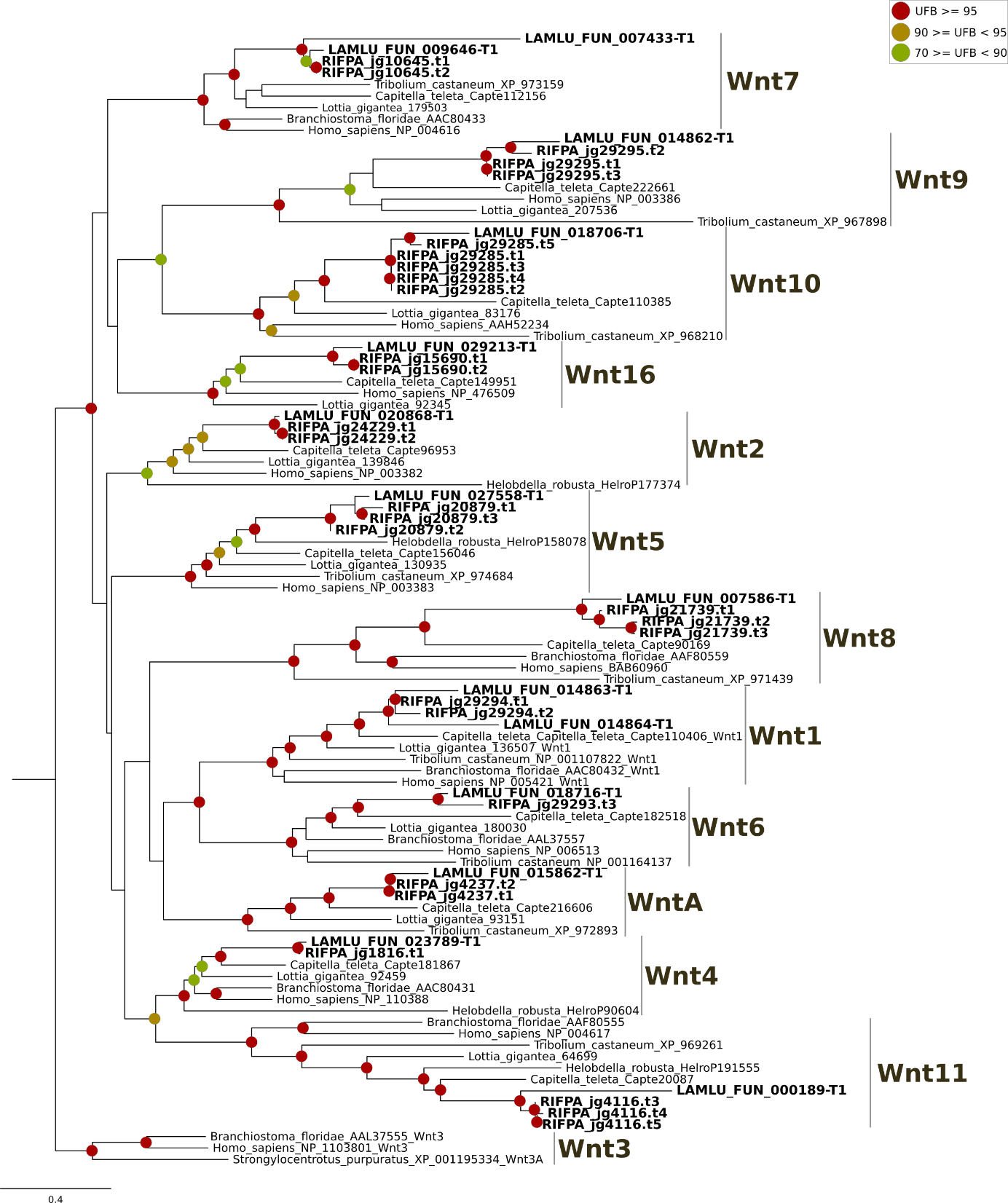
****Supplementary figure 14 | Phylogeny of Wnt ligands**. Maximum-likelihood phylogenetic tree inference of the Wnt ligand genes using 1000 ultrafast bootstrap replicates. *Riftia* contains all expected Wnt genes. The branch support values are represented by the coloured circles in the tree nodes. Red circles represent ultrafast bootstrap values >= 95. Yellow circles represent ultrafast bootstrap values >= 90 and < 95. Green circles represent ultrafast bootstrap values < 90 and >= 70. Ultrafast bootstrap values smaller than 70 are not shown. Deuterostome *Wnt3* genes were used as outgroup. Accession numbers for NCBI database are displayed after the species names. *Capitella, Helobdella* and *Lamellibrachia* gene identification are derived from the publicly available annotated genomes.

**
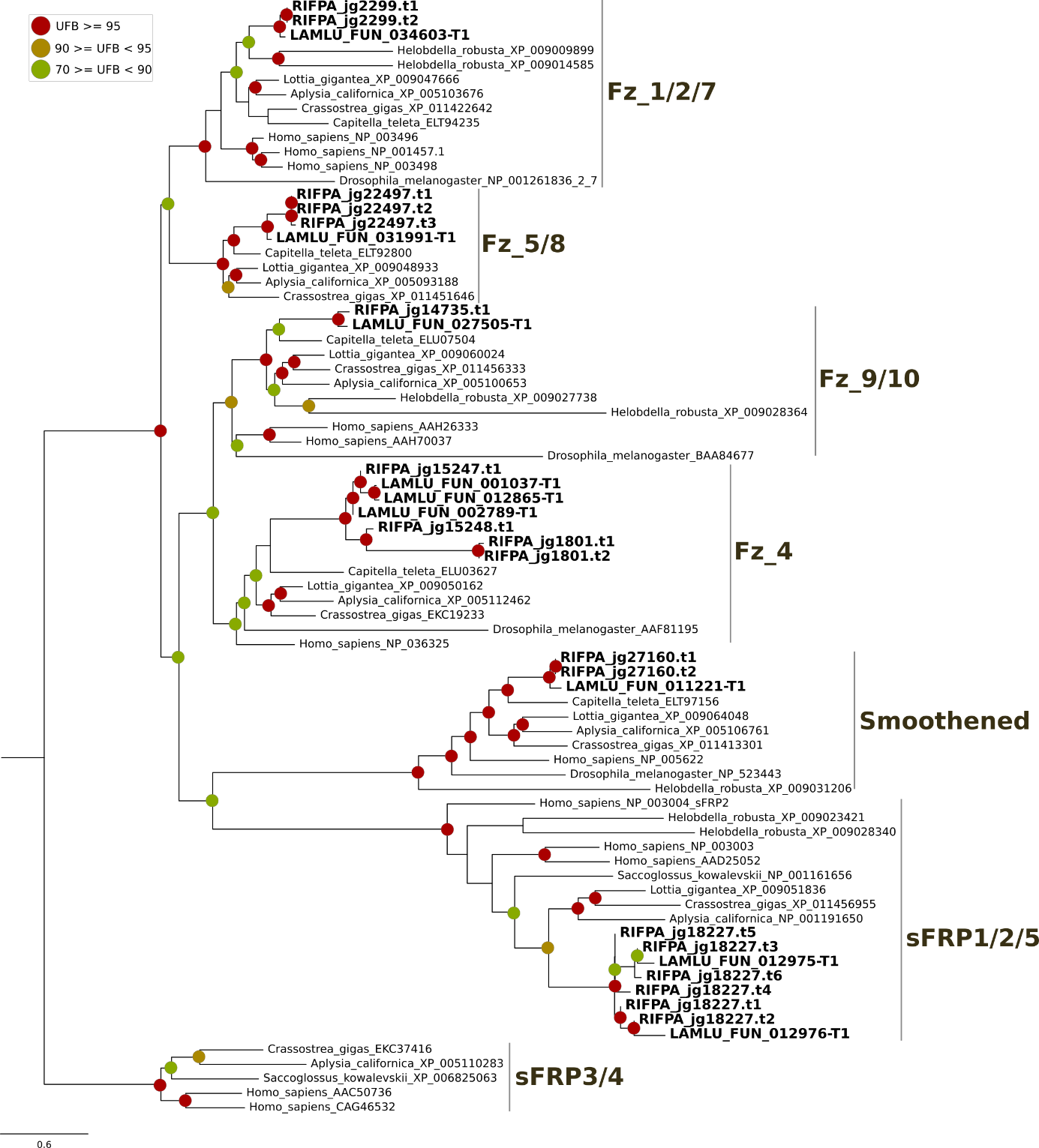
****Supplementary figure 15 | Phylogeny of Wnt receptor genes (Frizzled)**. Maximum-likelihood phylogenetic tree inference of the Wnt receptor genes using 1000 ultrafast bootstrap replicates. The branch support values are represented by the coloured circles in the tree nodes. Red circles represent ultrafast bootstrap values >= 95. Yellow circles represent ultrafast bootstrap values >= 90 and < 95. Green circles represent ultrafast bootstrap values < 90 and >= 70. Ultrafast bootstrap values smaller than 70 are not shown. SFRP3/4 receptor genes were used as outgroup. Accession numbers for NCBI database are displayed after the species names.


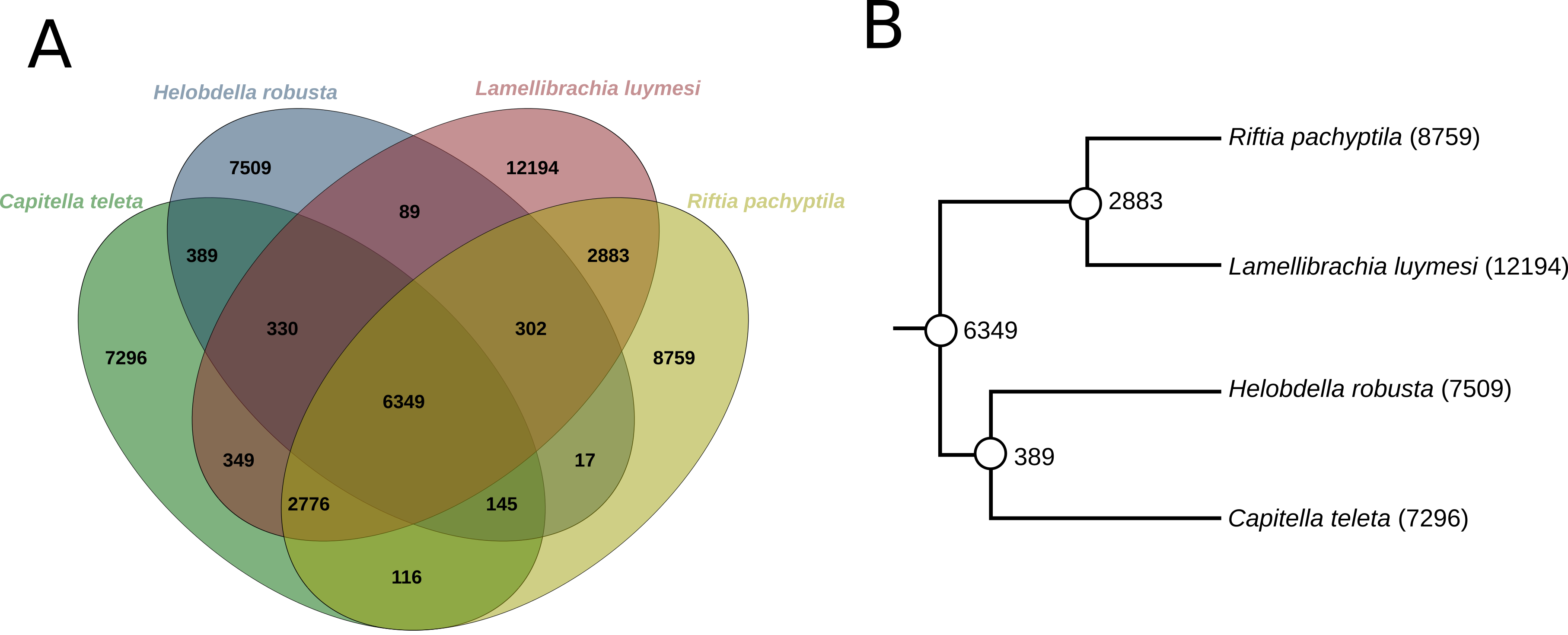


**Supplementary figure 16 | Shared orthogroups in Annelida. A**, Shared ortholog groups among *Capitella*, *Helobdella* and the two tubeworms *Lamellibrachia* and *Riftia*. The number of shared groups between *Riftia* and each of the two non-vestimentiferan worms *Helobdella* and *Capitella* is lower than the *Lamellibrachia-Helobdella* and *Lamellibrachia-Capitella* pairs indicating a more derived gene repertoire in the giant tubeworm. **B**, Annelida phylogenetic tree with the shared orthogroups mapped into the nodes. Numbers between in parenthesis represent the total number lineage-specific genes, i.e., orphan genes.


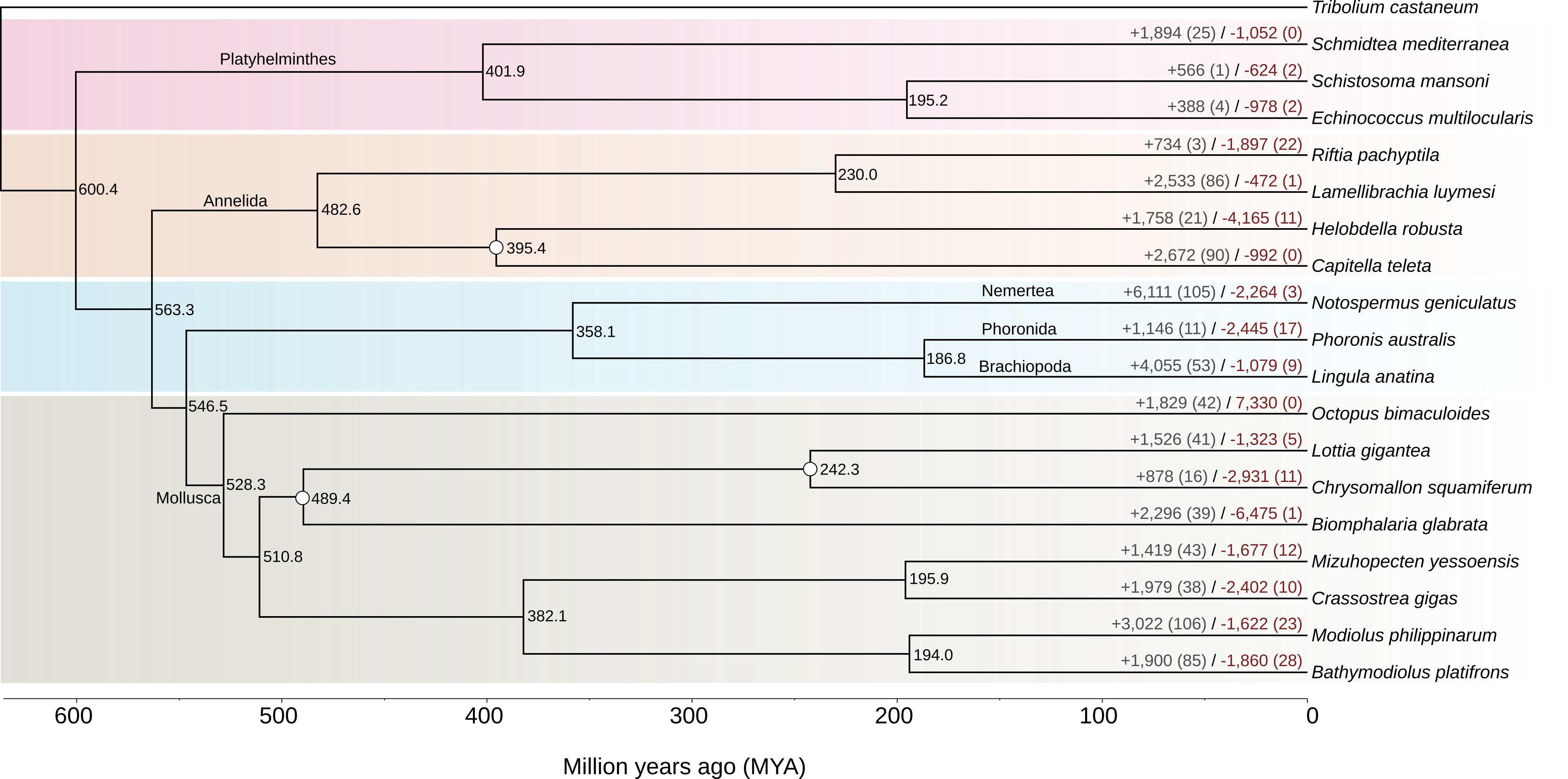


**Supplementary figure 17 | Gene gain and loss in selected lophotrochozoans.** Phylogenetic tree generated with PhyloBayes using available calibrations based on fossil records^10^ (white circles in the nodes). The ultrametric tree was obtained after 31,665 rounds discarding the initial 7,916 rounds as burn-in (25%). Gene family evolutionary history of gain and loss was analyzed with CAFE. The number of gene family gains and losses are marked by the plus (+) and minus (-) signs. *Riftia* genome shows a net reduction of gene numbers.


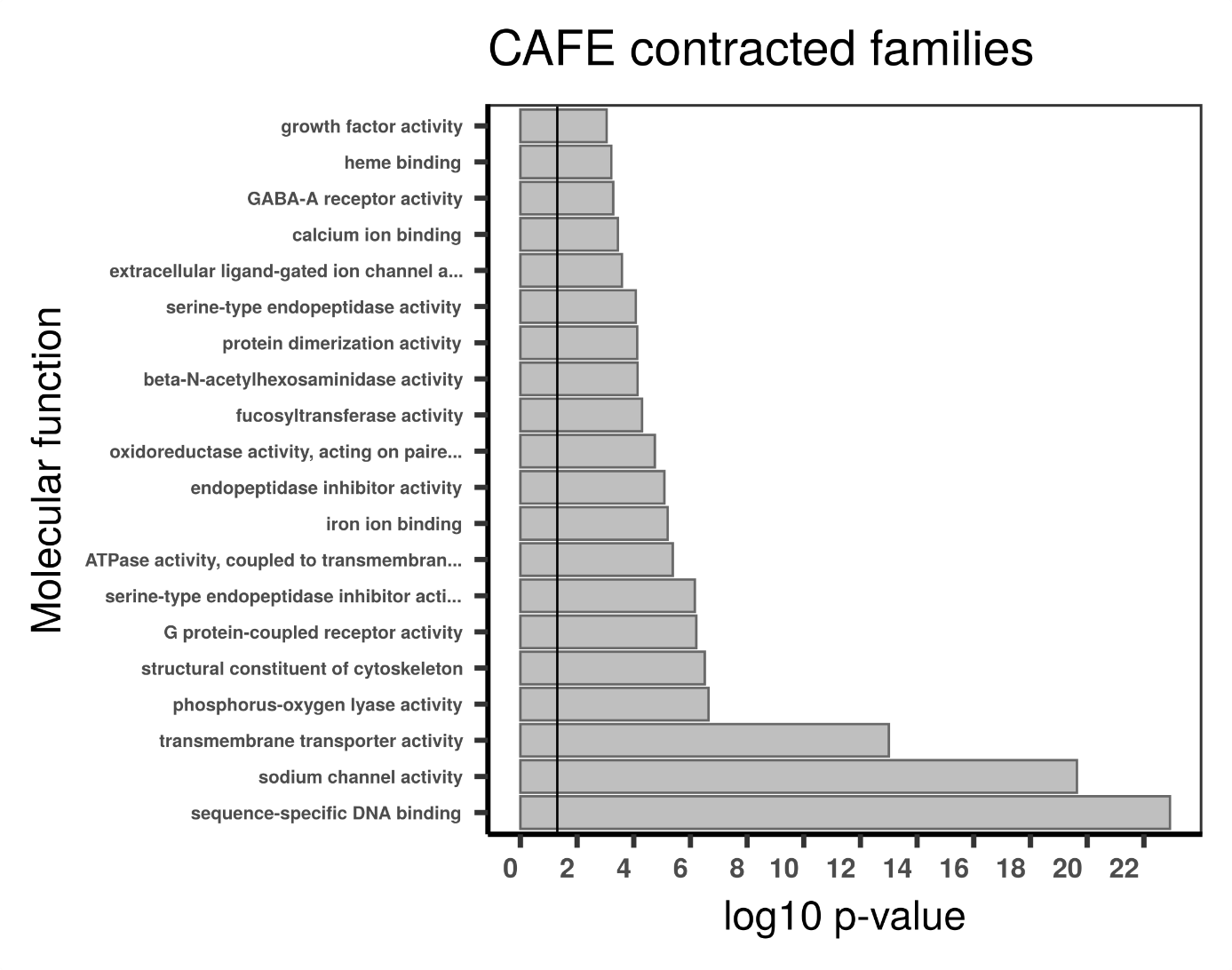

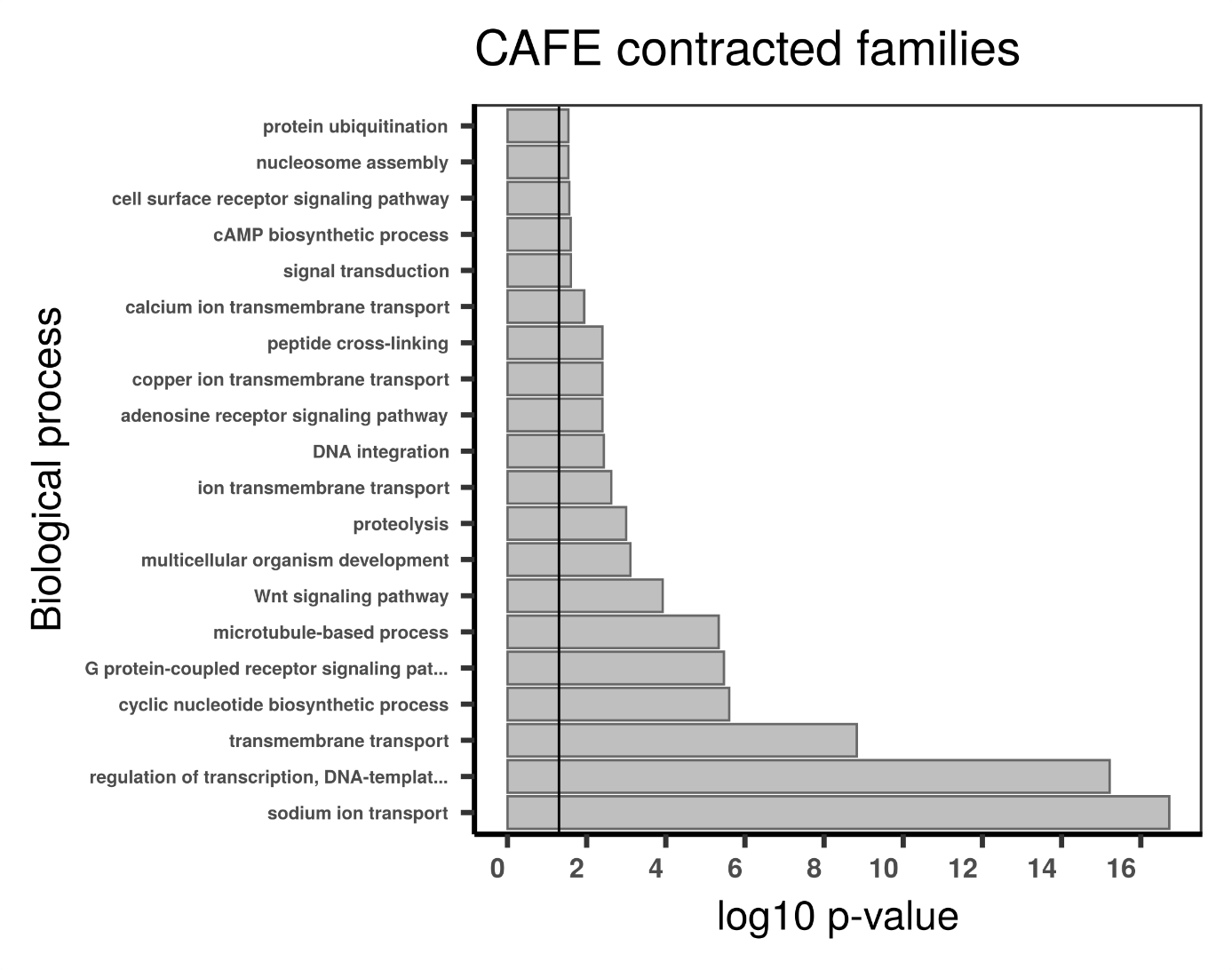


**Supplementary figure 18 | Gene set enrichment analysis with topGO using CAFE contract families in *Riftia***. Gene ontology (GO) enrichment analyses for CAFE contracted families. The graphs correspond to the three domains of ontologies: biological process (BP), molecular function (MF) and cellular component (CC). The selected genes were analysed for enrichment in specific GO categories using the TopGO program against the background (all coding sequence genes). Y-axis corresponds to enriched GO terms found in the respective domains (BP, MF and CC). X-axis correspond to the log function of Fisher p-values obtained for each one of the enriched terms. The back line denotes a p-value = 0.05. P-values greater than 1,30 (log 0.05) indicate statistically significant enriched term.
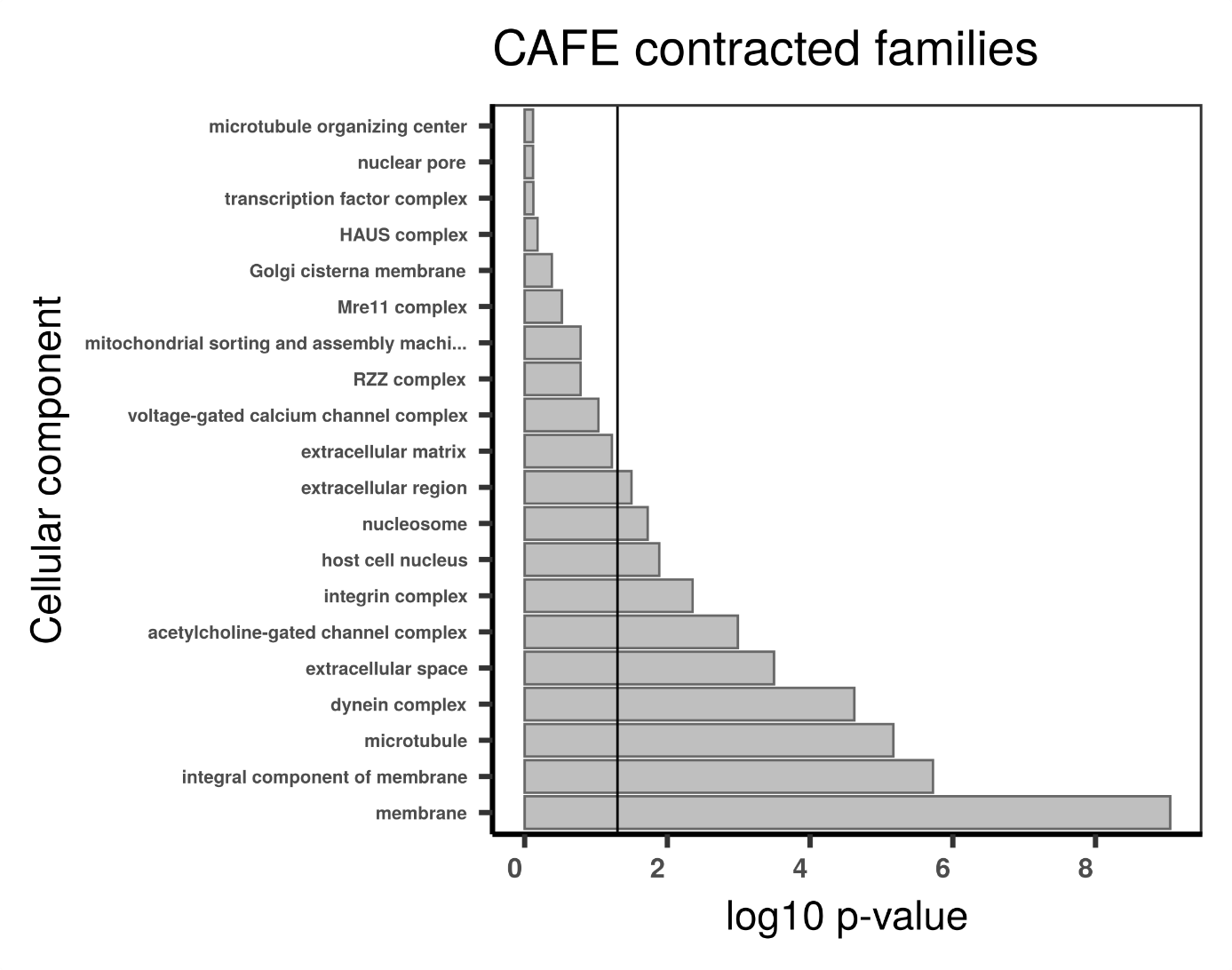
 The contracted gene families are not restricted to any specific biological process or molecular function, suggesting that the giant tubeworm genome is undergoing a broad reduction in gene content (i.e., reductive evolution).


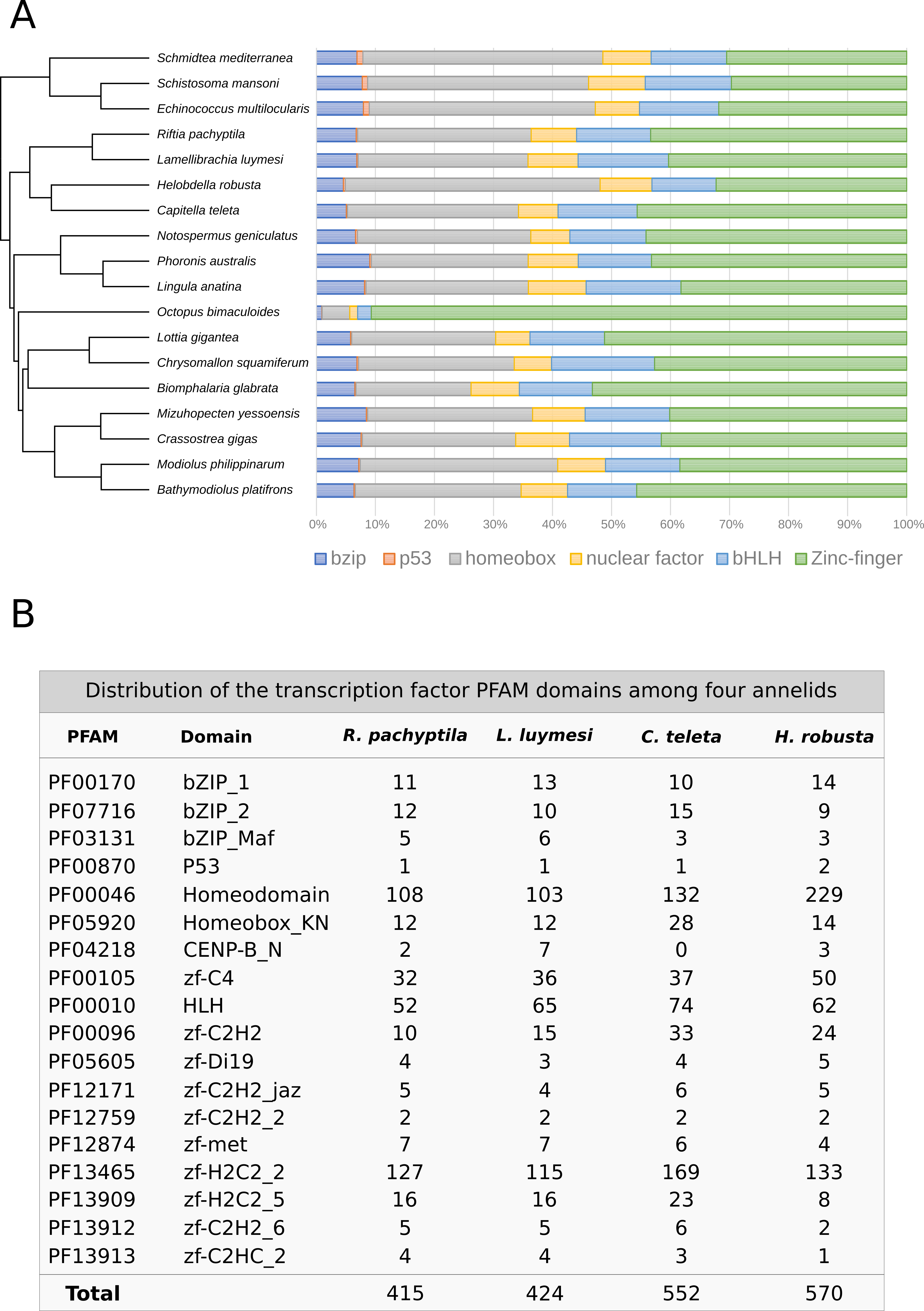


**Supplementary figure 19 | Distribution of transcription factors among lophotrochozoans** . **A,** Percent stacked barchart of the six major classes of transcription factors (TFs) among lophotrochozoans. *Riftia pachyptila* TF complement is similar to other lophotrochozoans. *Octopus bimaculoides* complement is composed of a massive expansion of zinc-finger elements, as previously described^9^. **B,** Distribution of the transcription factor PFAM domains among four annelids. *Riftia* TF complement is the lowest among the annelids.


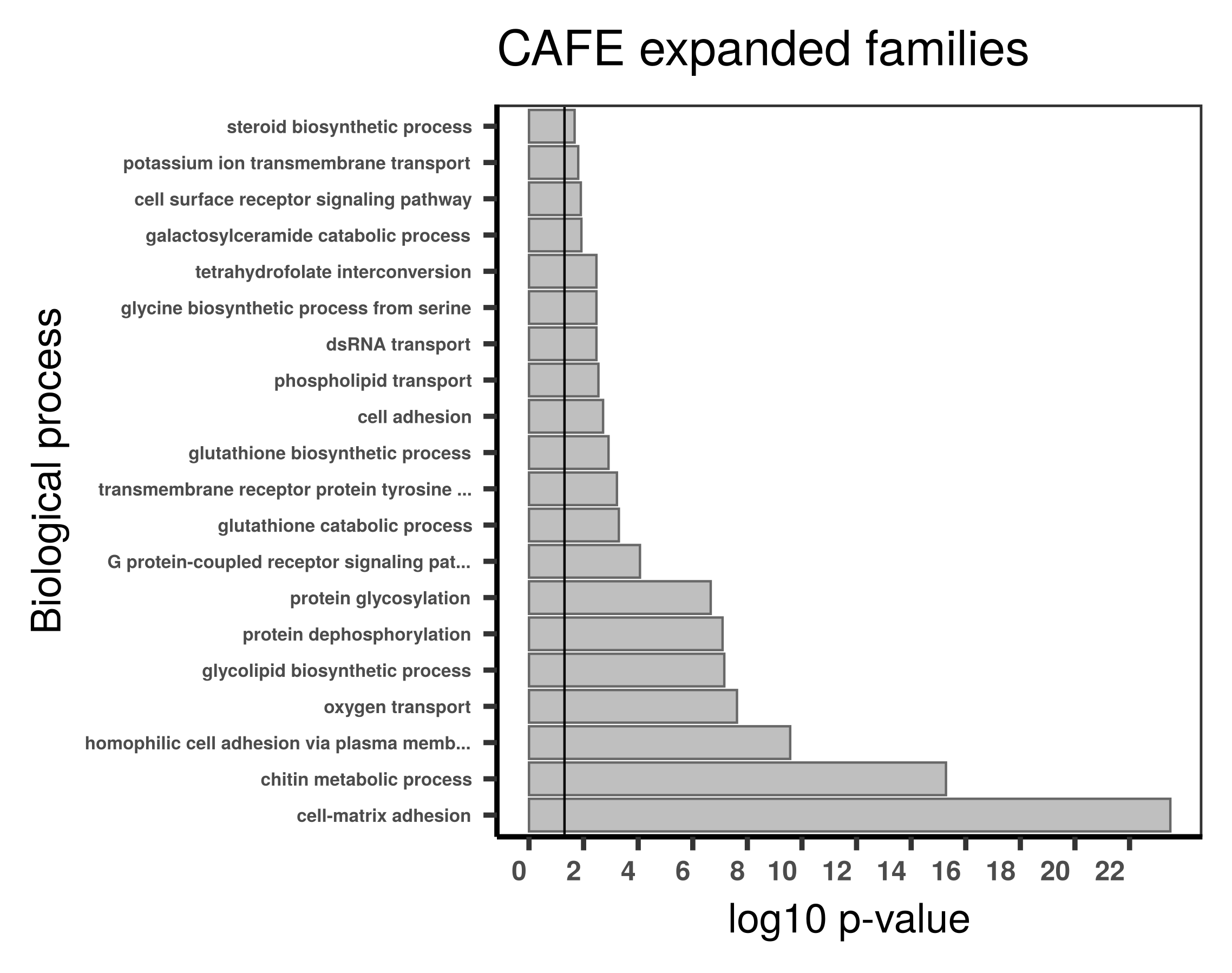


**Supplementary figure 20 | Gene set enrichment analysis with topGO using CAFE expanded families in *Riftia***.
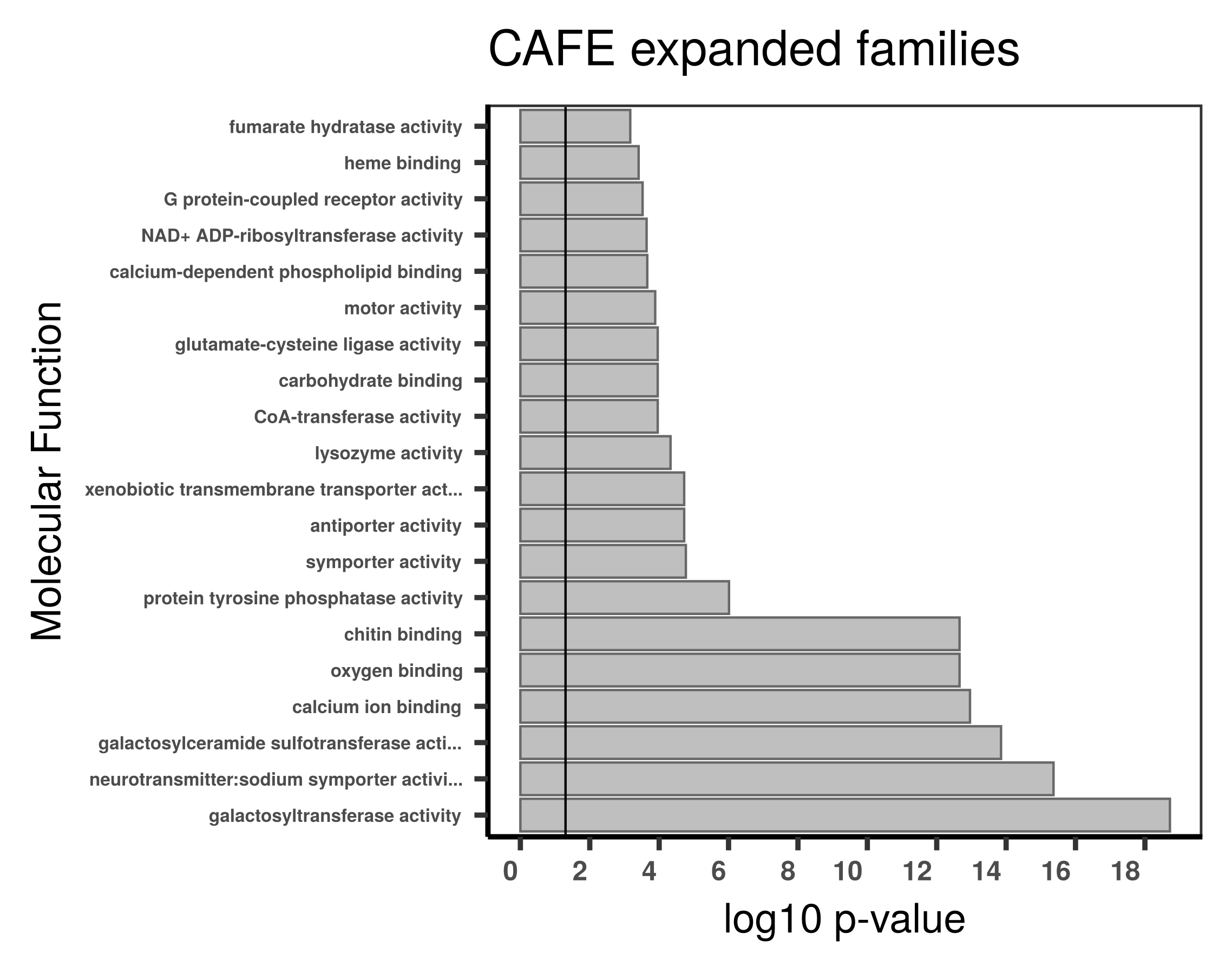

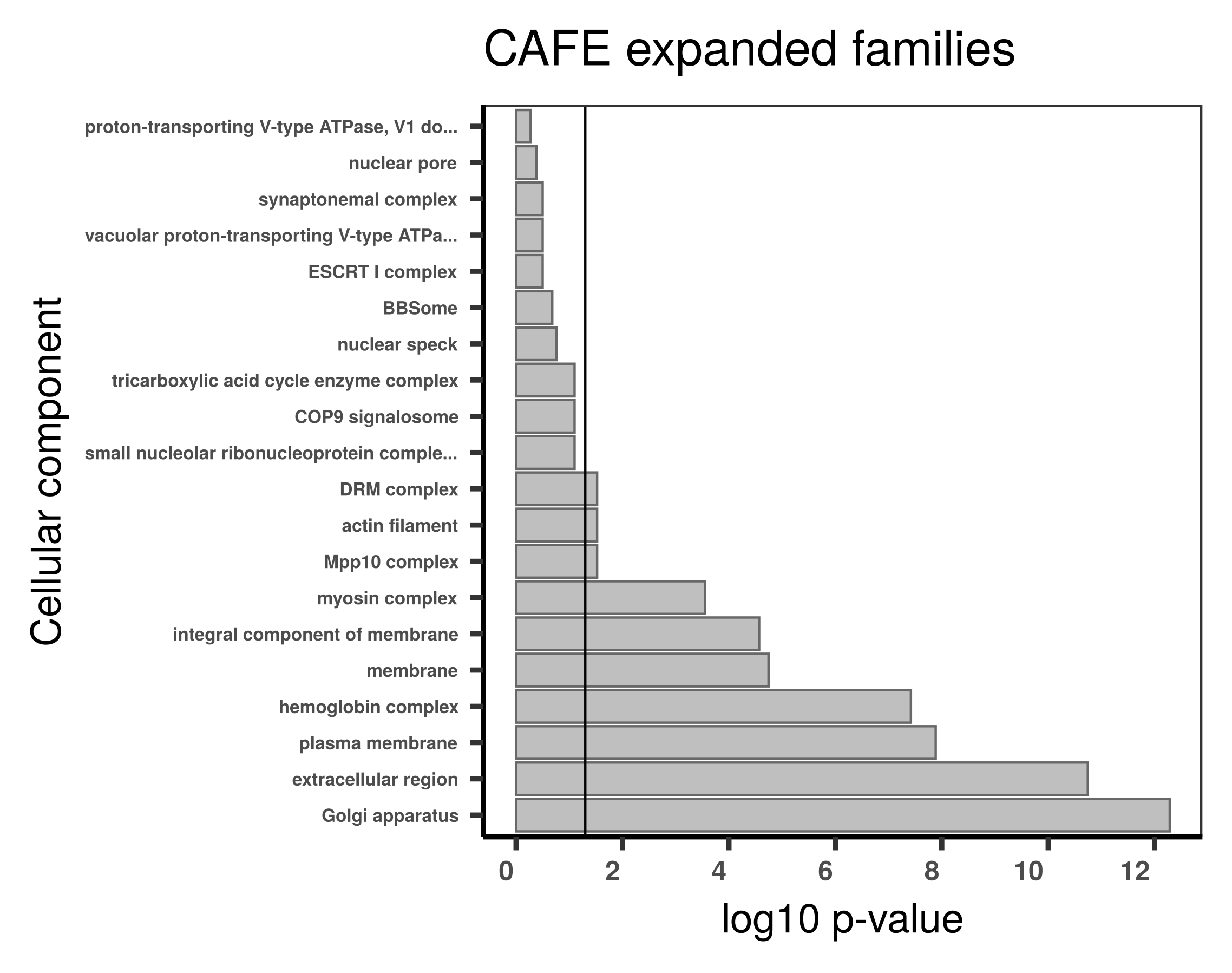
 Gene ontology (GO) enrichment analyses for CAFE expanded families. The graphs correspond to the three domains of ontologies: biological process (BP), molecular function (MF) and cellular component (CC). The selected genes were analysed for enrichment in specific GO categories using the TopGO program against the background (all coding sequence genes). Y axis corresponds to enriched GO terms found in the respective domains (BP, MF and CC). X axis correspond to the log function of Fisher p-values obtained for each one of the enriched terms. The back line denotes a p-value = 0.05. P-values greater than 1,30 (log 0.05) indicate statistically significant enriched term. Genes involved in sulphur metabolism and detoxification, anti-oxidative stress, oxygen transport, neurotransmitter- and ion channel-related functions, digestion (lysozyme activity) and secretion of chitinous material are expanded in the *Riftia pachyptila* tubeworm genome.


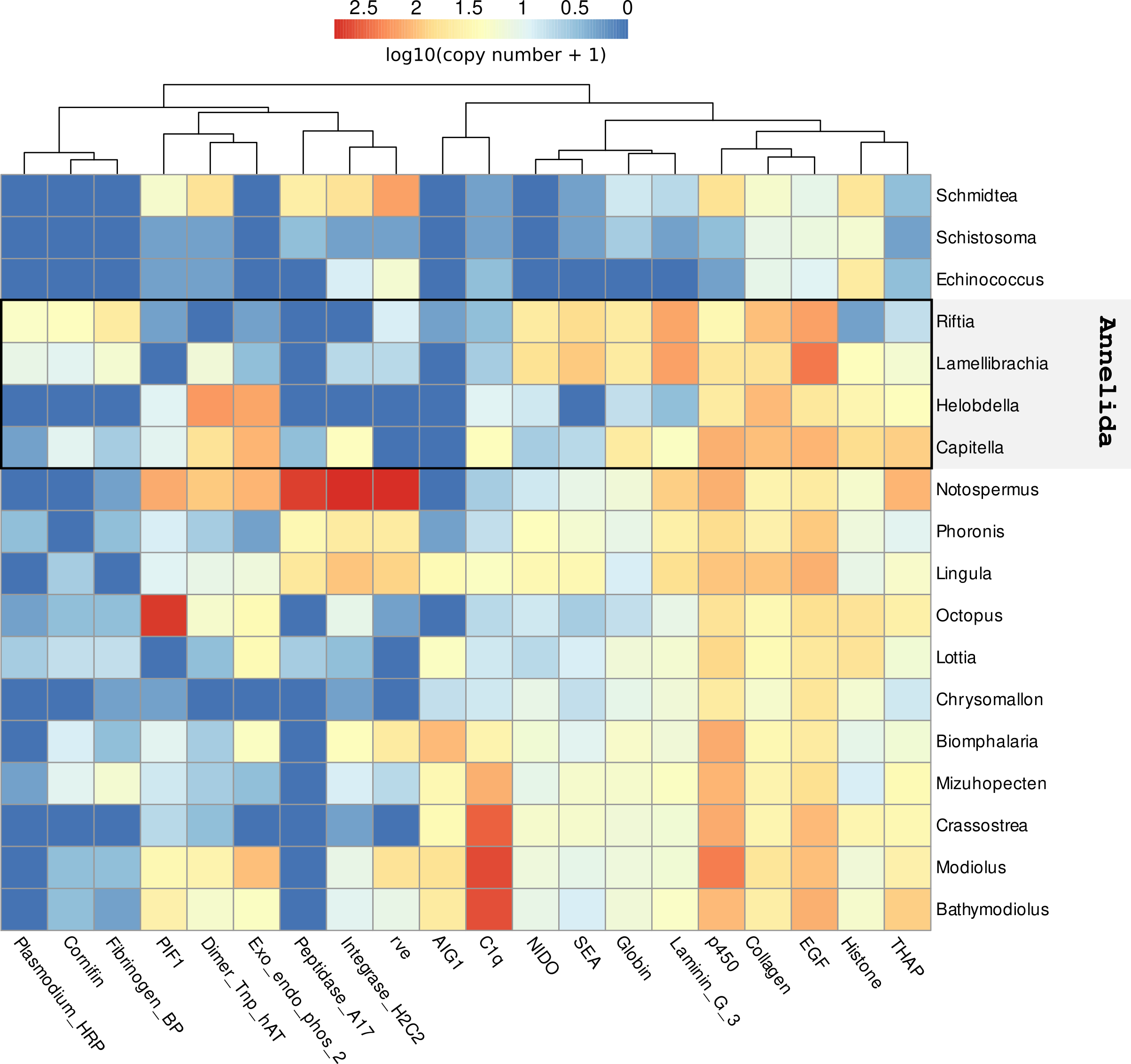


**Supplementary figure 21 | Distribution of *Riftia* expanded/contracted PFAM domains among selected lophotrochozoans.** Log distribution of the number of *Riftia* expanded/contracted PFAM domains among flatworms (*Schmidtea*, *Schistosoma*, *Echinococcus*), *molluscs* (*Octopus*, *Lottia*, *Chrysomallon*, *Biomphalaria*, *Mizuhopecten*, *Crassotrea*, *Modiolus*, *Bathymodiolus*), brachiopods (*Lingula*), nemerteans (*Notospermus*) and phoronids (*Phoronis*). PFAM domains were considered enriched or contracted in *Riftia* using two-tailed Fisher exact test (p-value < 0.01 after correction) against the outgroup average (i.e., lophotrochozoans minus *Riftia*). Multiple protein domains in a single given gene were counted only once. Expanded domains in *Riftia* include globin, NIDO, Laminin_G_3, EGF and collagen PFAMs.

**
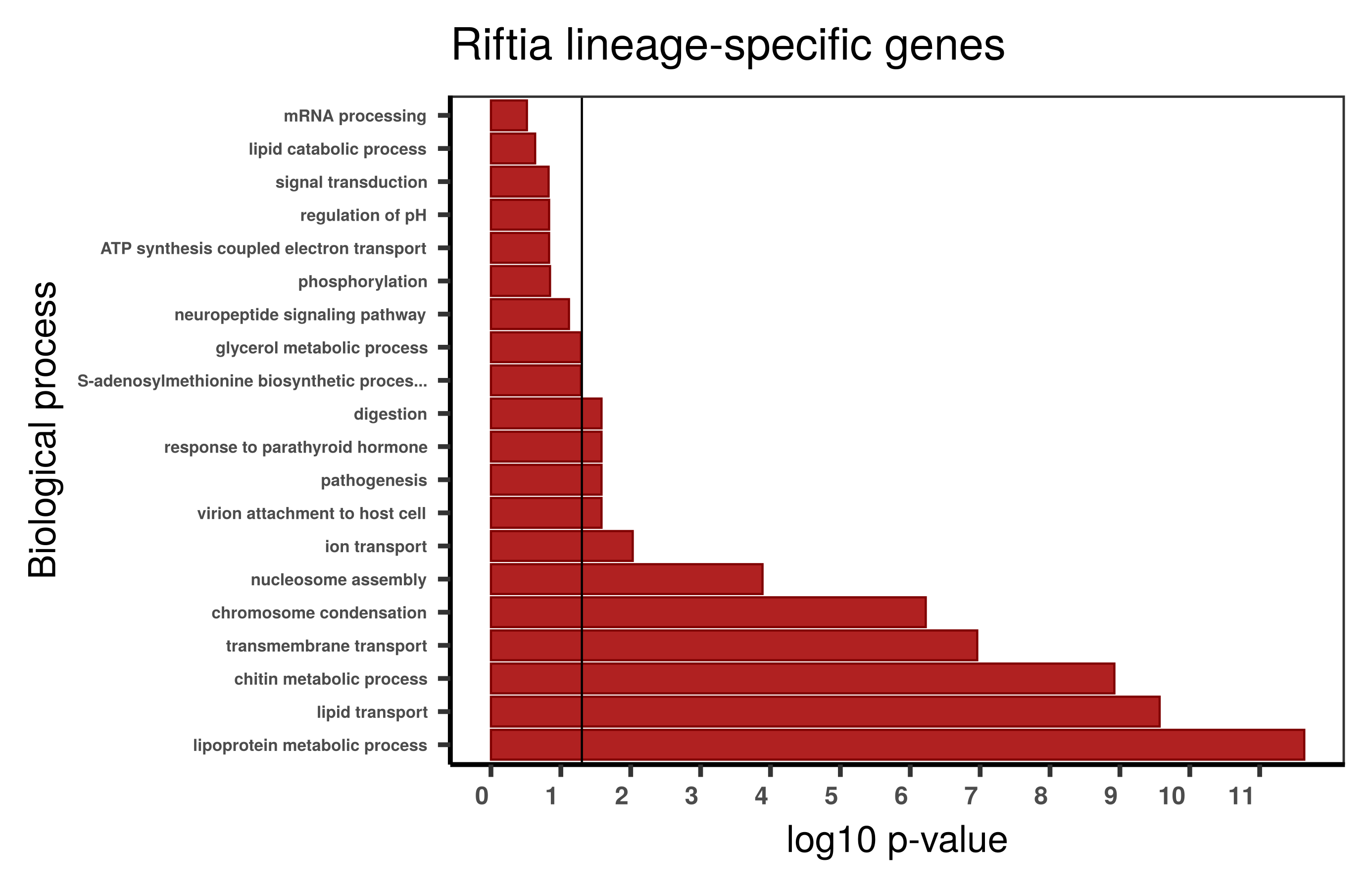
**


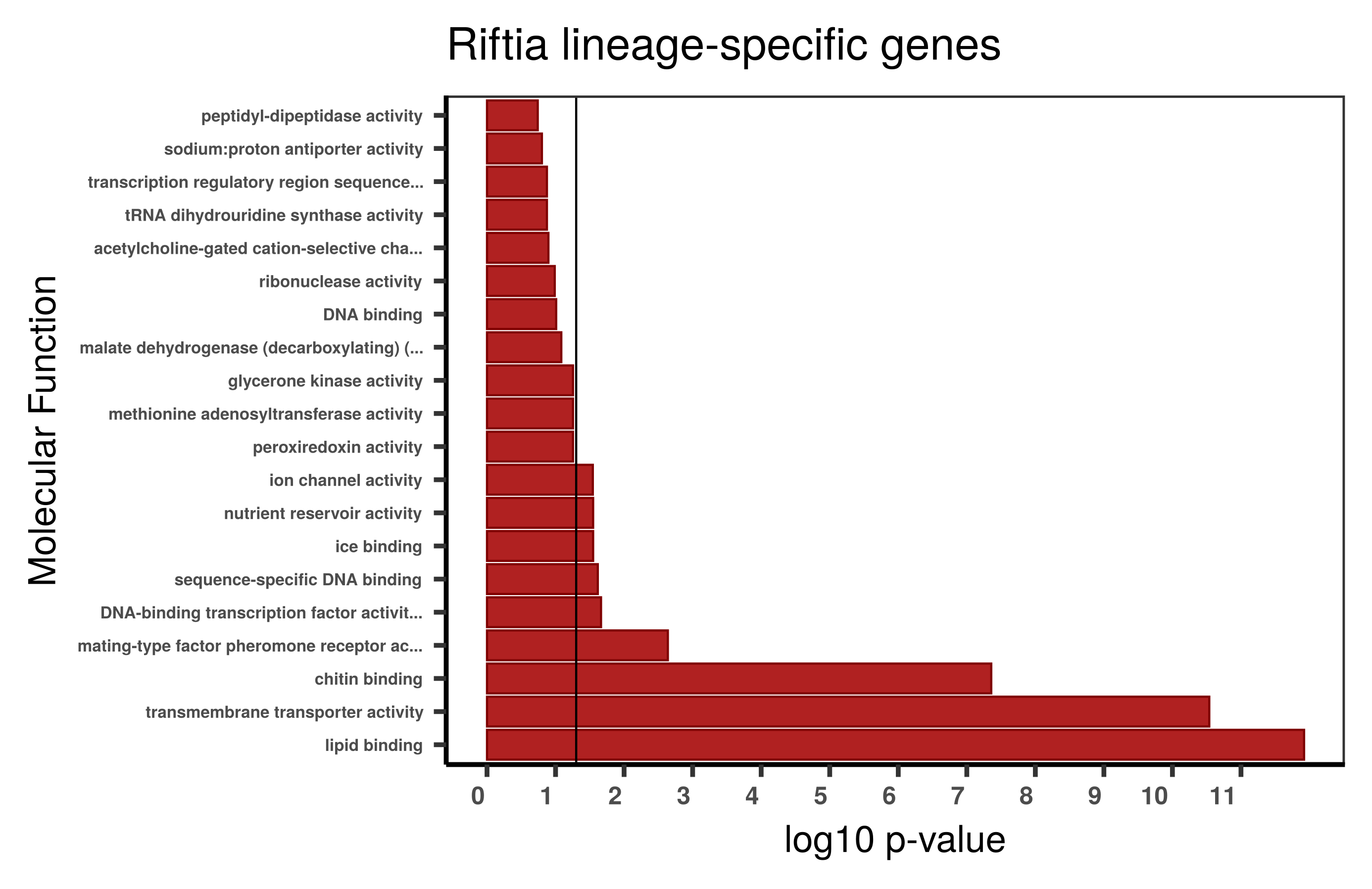


**Supplementary figure 22 | Gene set enrichment analysis with topGO using *Riftia pachyptila* lineage-specific genes**
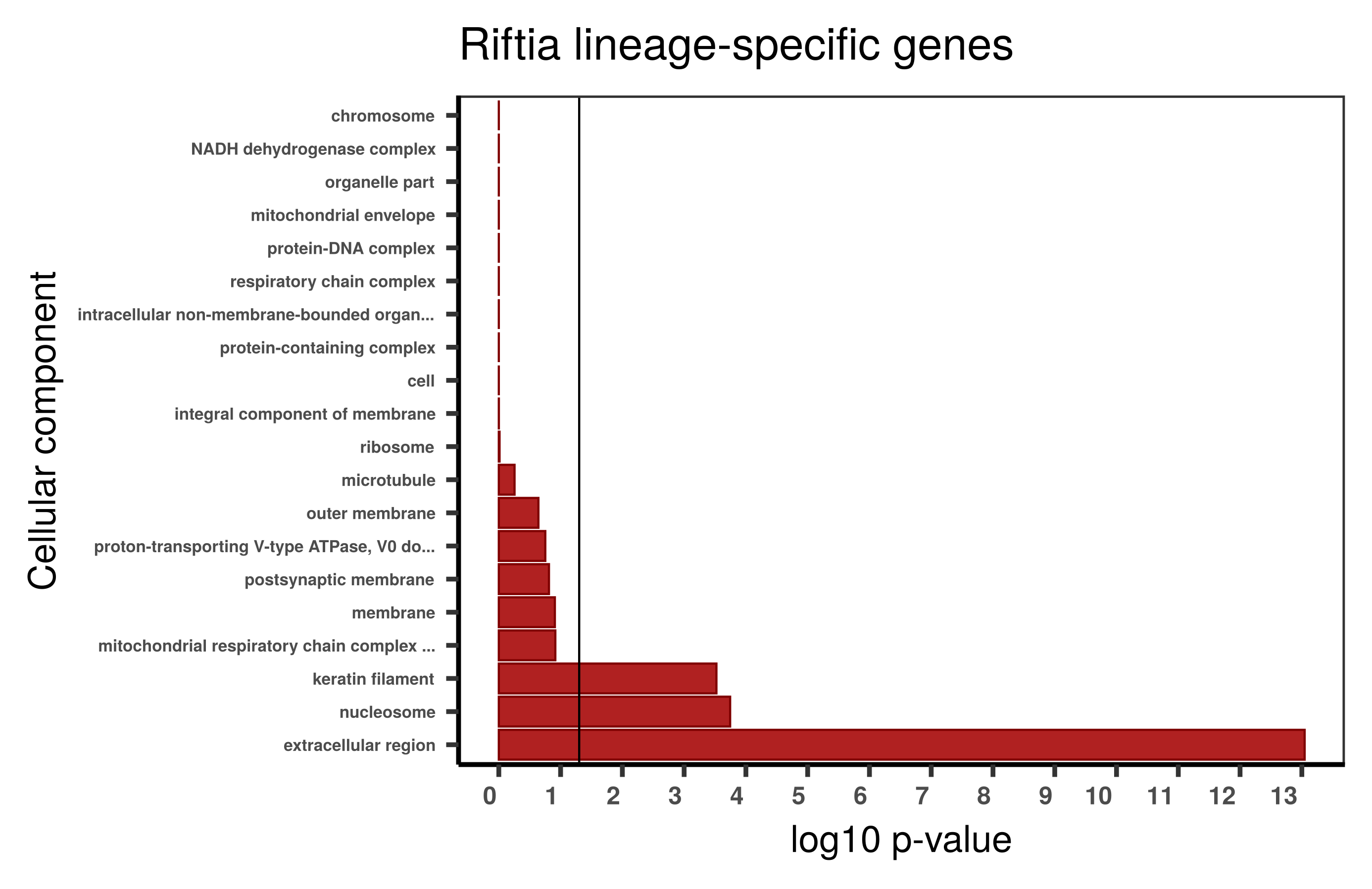
. Gene ontology (GO) enrichment analyses for *Riftia pachyptila* lineage-specific genes. The graphs correspond to the three domains of ontologies: biological process (BP), molecular function (MF) and cellular component (CC). The selected genes were analysed for enrichment in specific GO categories using the TopGO program against the background (all coding sequence genes). Y-axis corresponds to enriched GO terms found in the respective domains (BP, MF and CC). X-axis correspond to the log function of Fisher p-values obtained for each one of the enriched terms. The back line denotes a p-value = 0.05. P-values greater than 1,30 (log 0,05) indicate statistically significant enriched term. Genes involved in cell cycle and chitin production and secretion are enriched in *Riftia*.


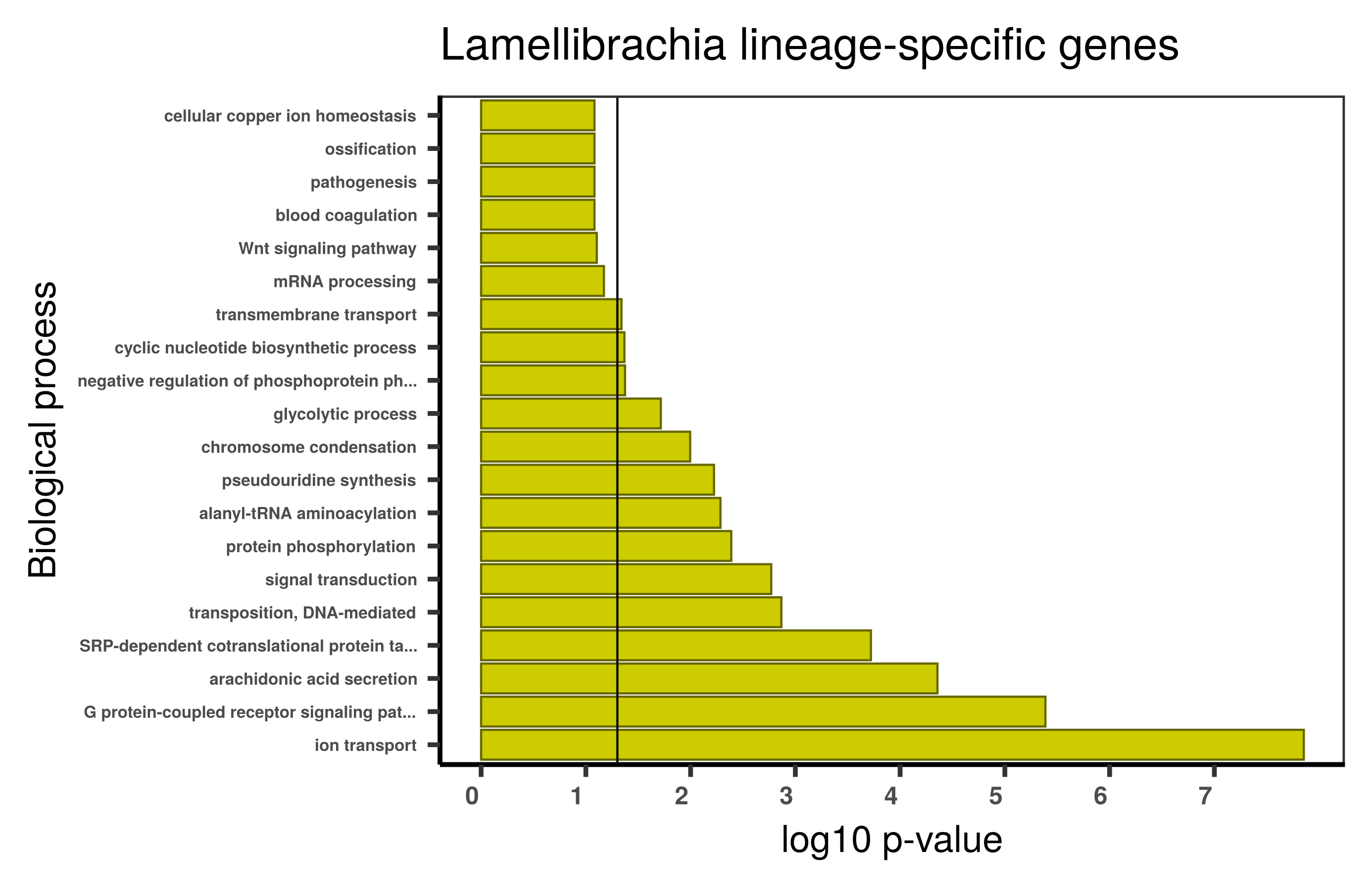


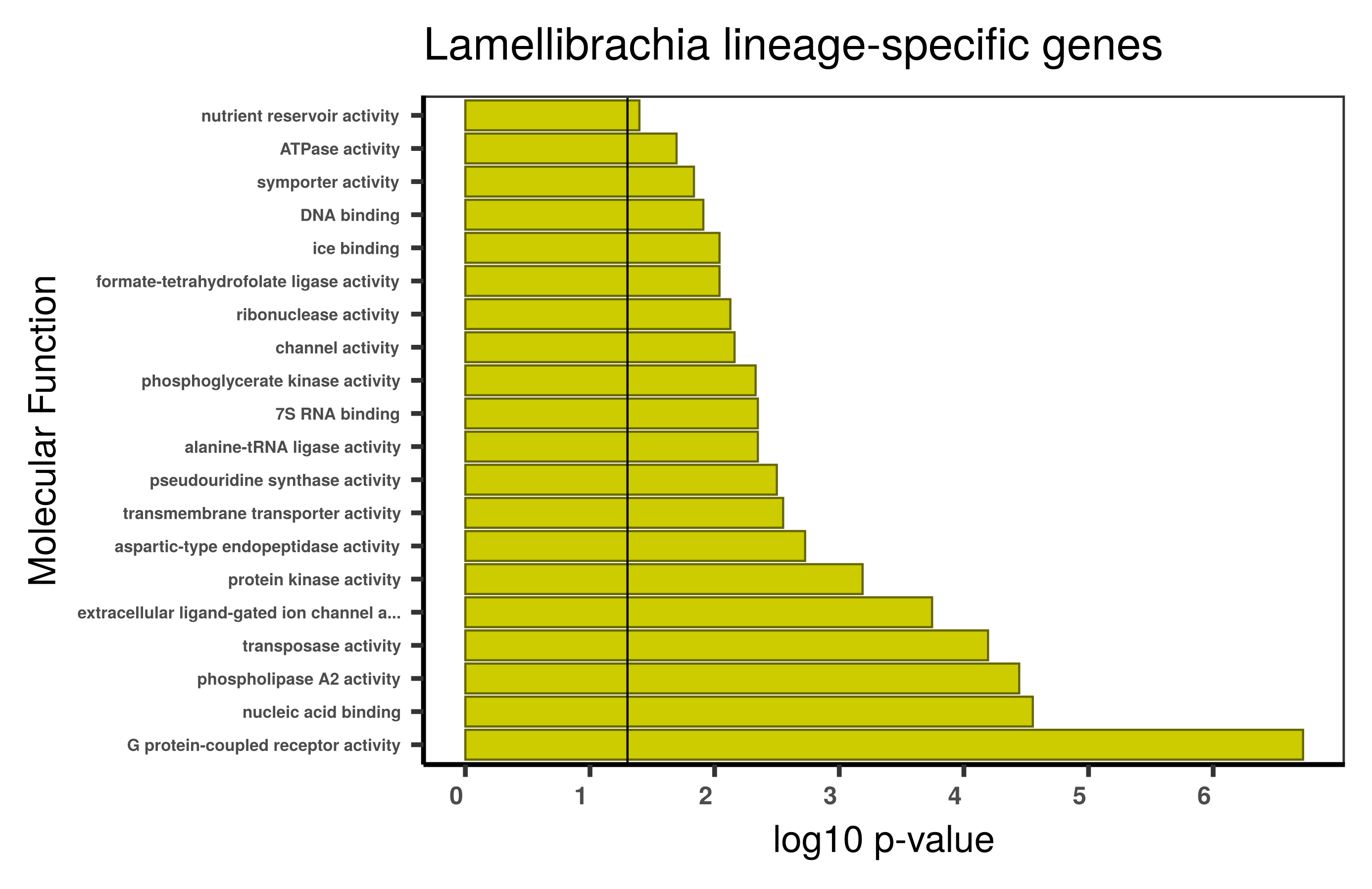


**Supplementary figure 23 | Gene set enrichment analysis with topGO using *Lamellibrachia luymesi* lineage-specific genes**
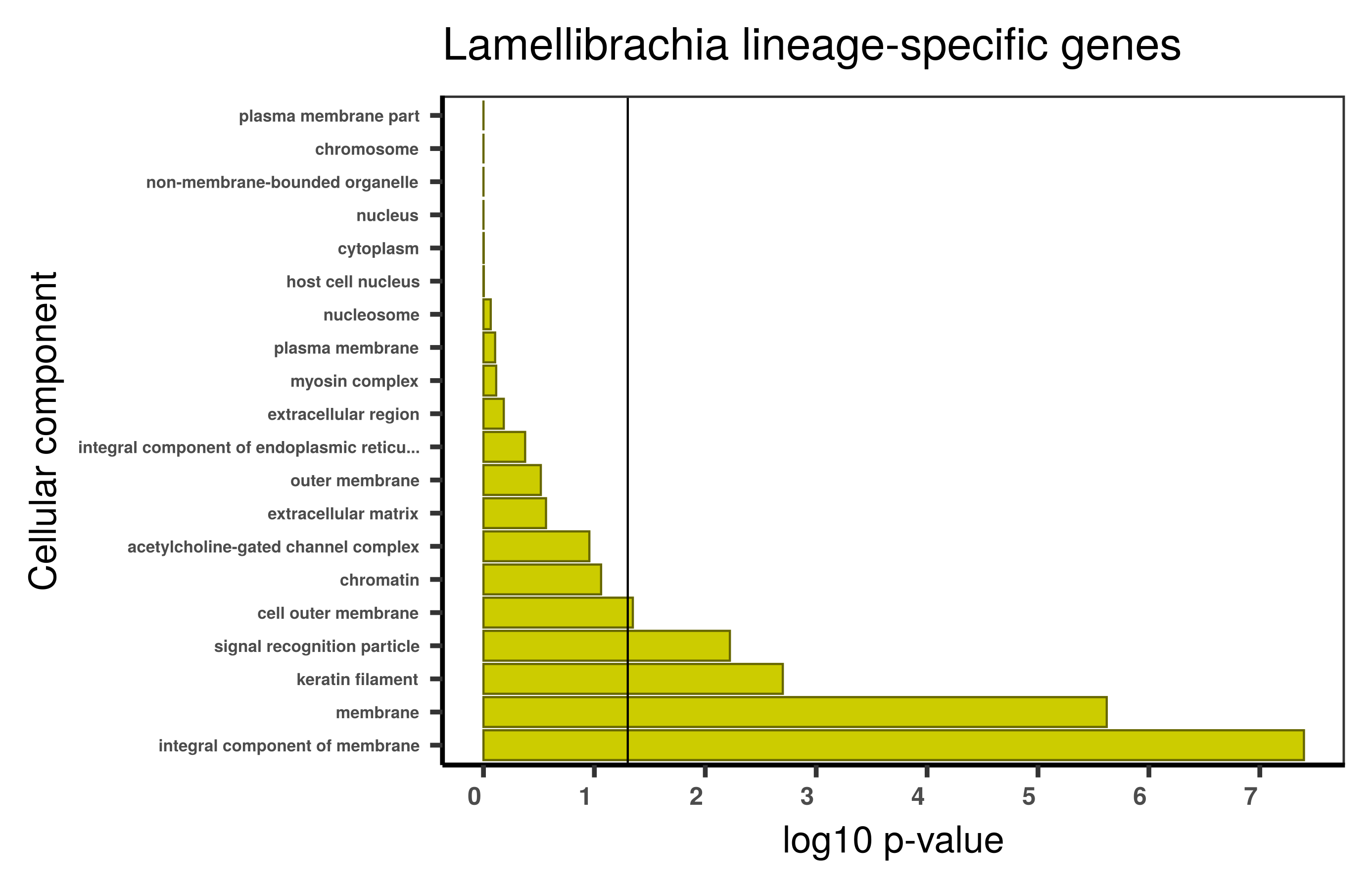
. Gene ontology (GO) enrichment analyses for *Lamellibrachia luymesi* lineage-specific genes. The graphs correspond to the three domains of ontologies: biological process (BP), molecular function (MF) and cellular component (CC). The selected genes were analysed for enrichment in specific GO categories using the TopGO program against the background (all coding sequence genes). Y-axis corresponds to enriched GO terms found in the respective domains (BP, MF and CC). X-axis correspond to the log function of Fisher p-values obtained for each one of the enriched terms. The back line denotes a p-value = 0.05. P-values greater than 1,30 (log 0,05) indicate statistically significant enriched term. Genes involved in transposase activity and cell cycle are enriched in *Lamellibrachia*.


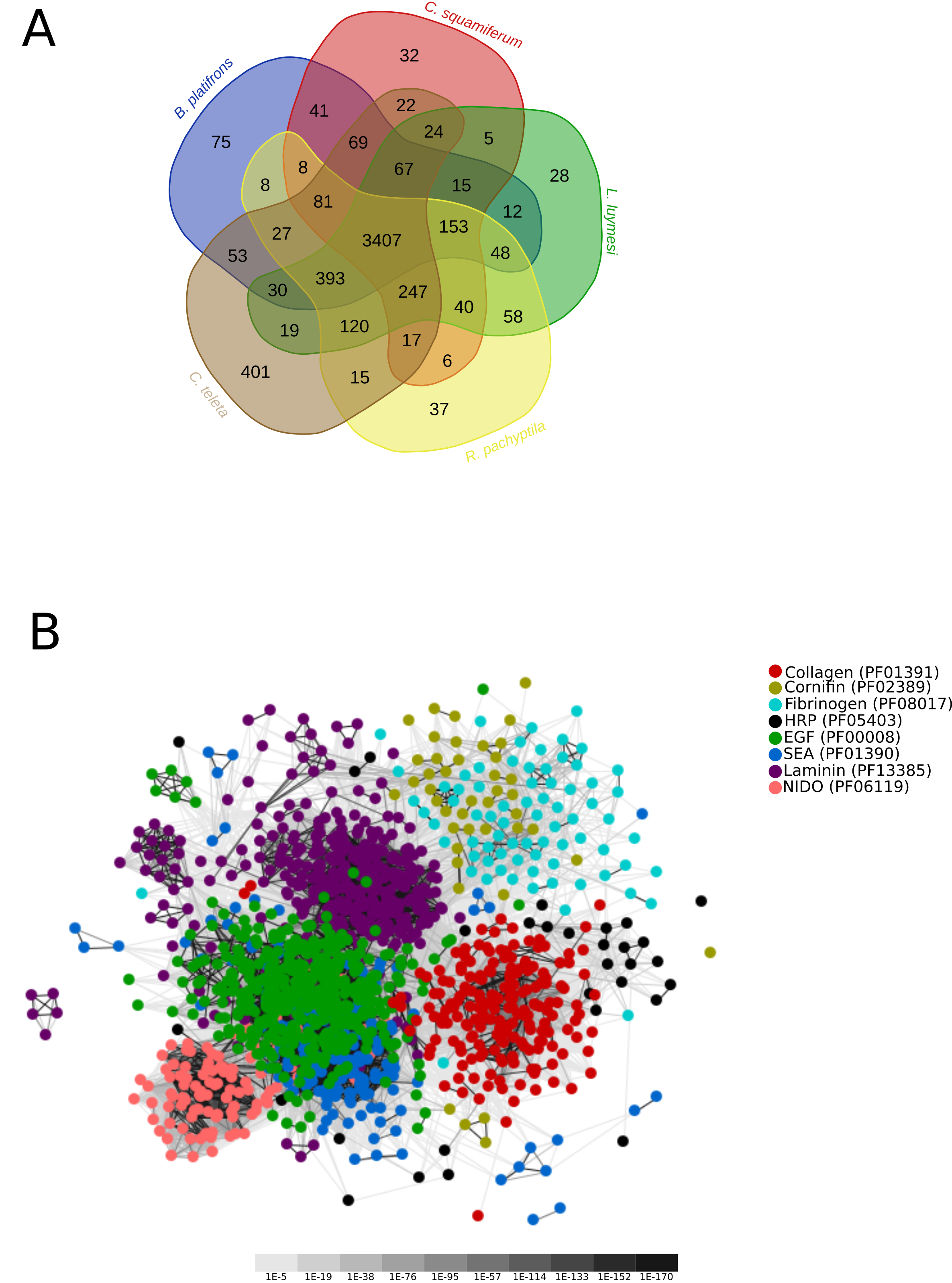


**Supplementary figure 24 | PFAM functional domains shared among selected deep vent lophotrochozoans and cluster analysis of selected *Riftia* expanded domains.** Venn diagram depicting the shared PFAM functional domains among tubeworms (*Riftia* and *Lamellibrachia*), one bivalve (*Bathymodiolus platifrons*), one gastropod (*Chrysomallon squamiferum*) and the polychaete *Capitella teleta.* **B,** 2D-cluster map of collagen, cornifin, fibrinogen, HRP, EGF, SEA, Laminin and NIDO domain-containing proteins in *Riftia*. Colours are based on the different PFAM domain. Edges correspond to blastp connections of p-value < 1e-06. The high connectivity of the cluster is probably due the presence of small repetitive protein domains in the genes (EGF, collagen), rather than evolutionary relatedness.


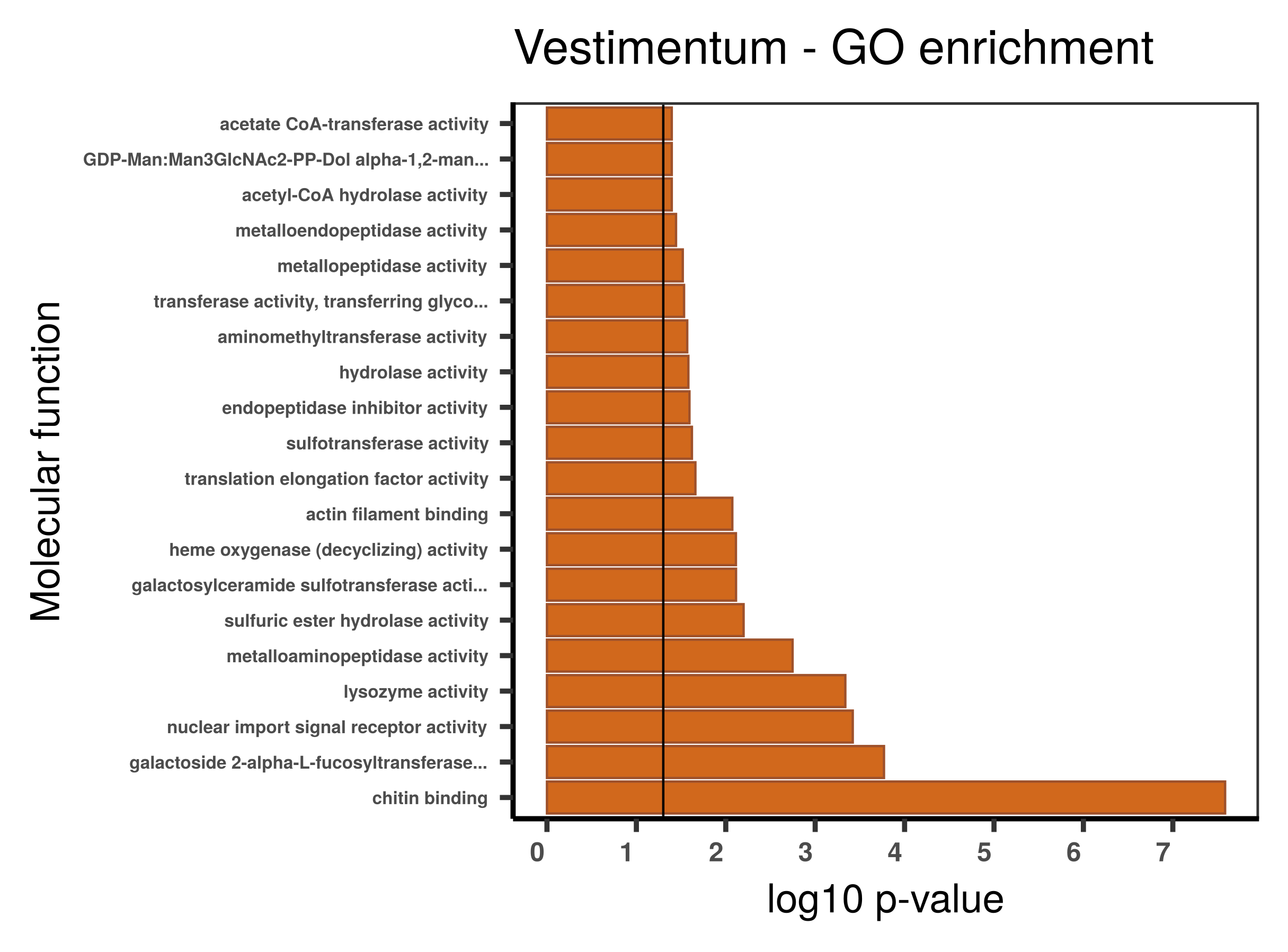

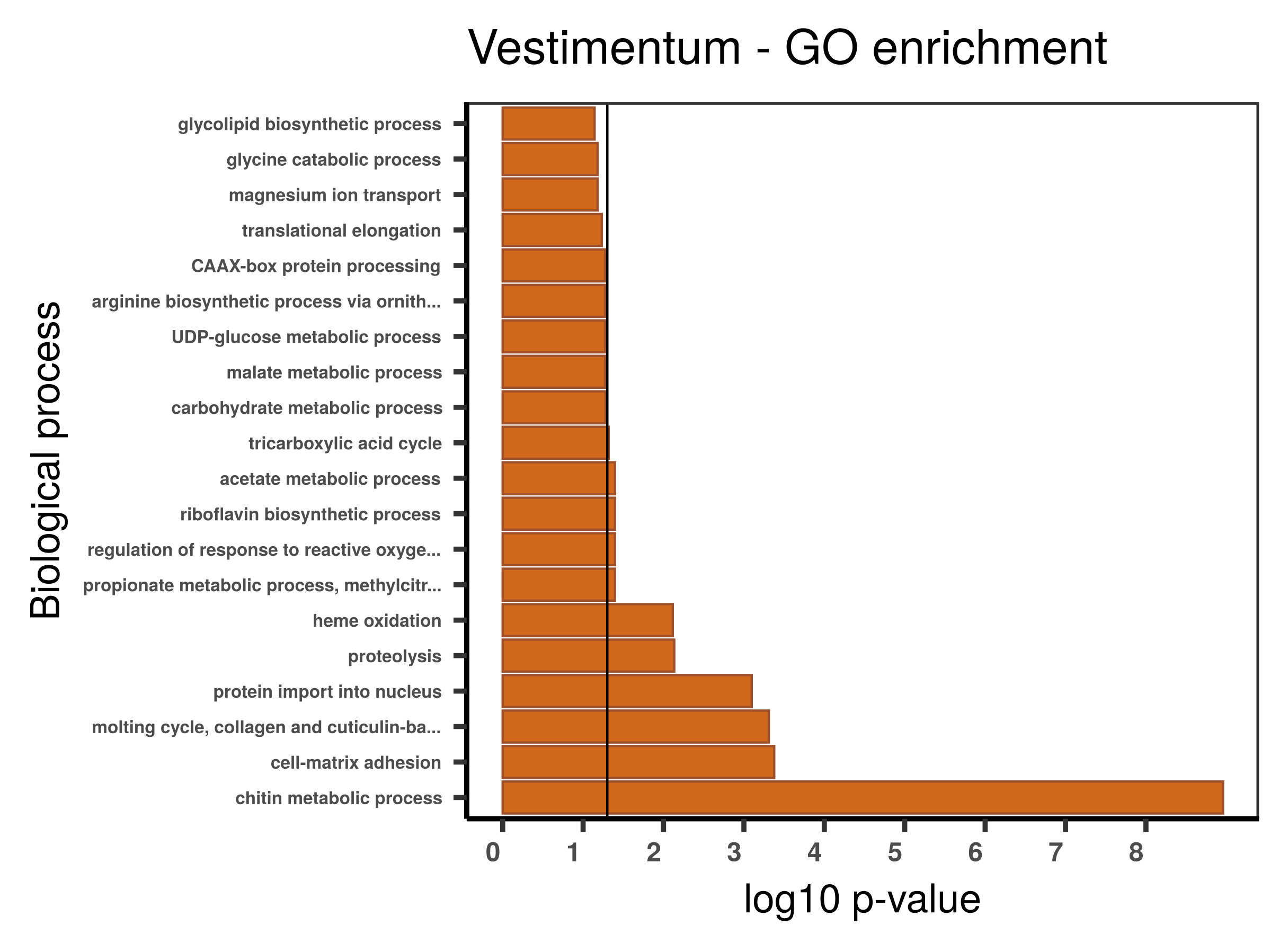


**Supplementary figure 25 | Gene set enrichment analysis with topGO using absolutely vestimentum specific TAU genes**. Gene ontology (GO) enrichment analyses for absolutely vestimentum specific TAU genes. The graphs correspond to the three domains of ontologies: biological process (BP), molecular function (MF) and cellular component (CC). The selected genes were analysed for enrichment in specific GO categories using the TopGO program against the background (all coding sequence genes). Y axis corresponds to enriched GO terms found in the respective domains (BP, MF and CC). X axis correspond to the log function of Fisher p-values obtained for each one of the enriched terms. The back line denotes a p-value = 0.05. P-values greater than 1,30 (log 0,05) indicate statistically significant enriched term. Genes involved chitin metabolism are differentially expressed in the vestimentum


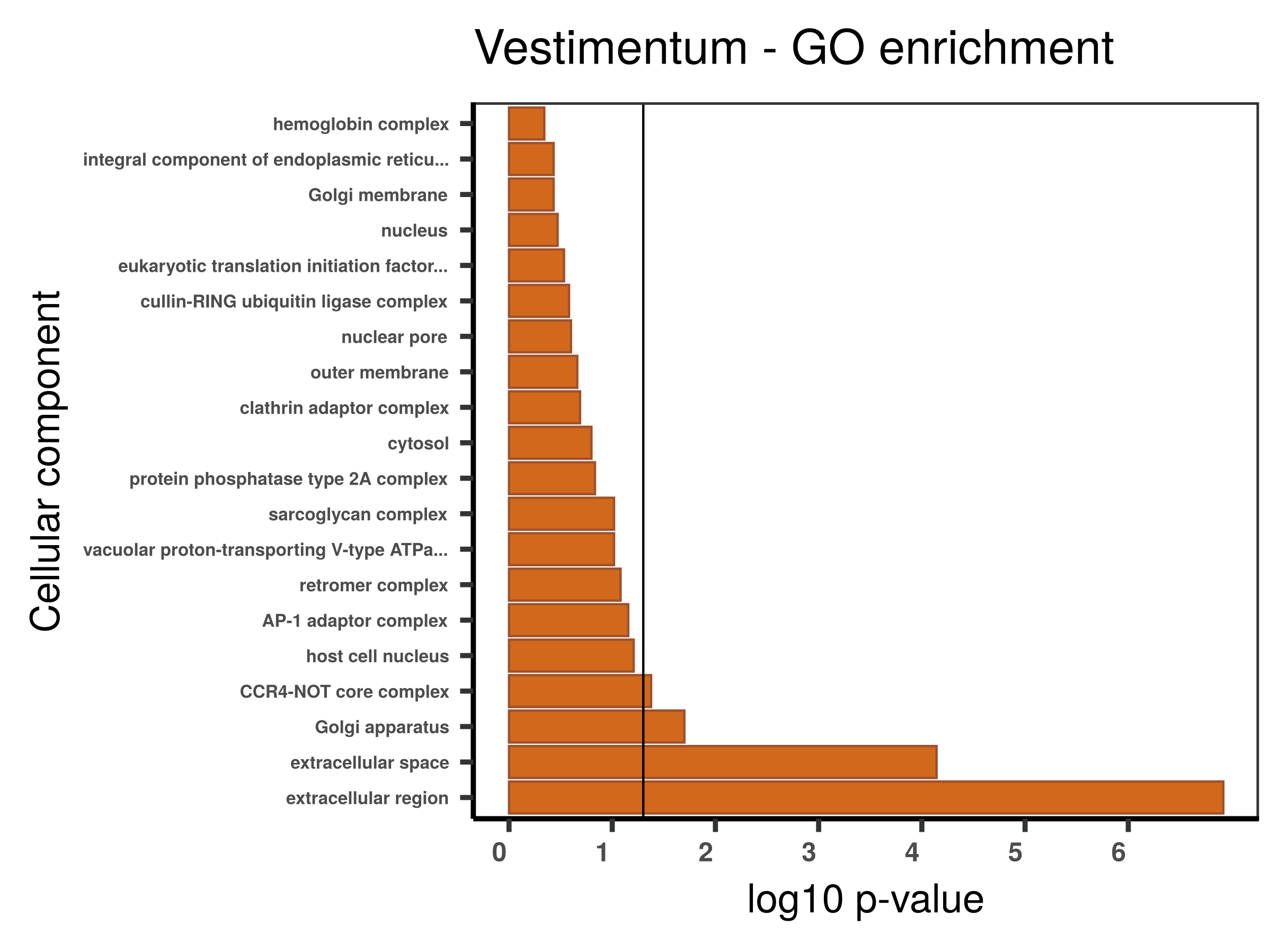


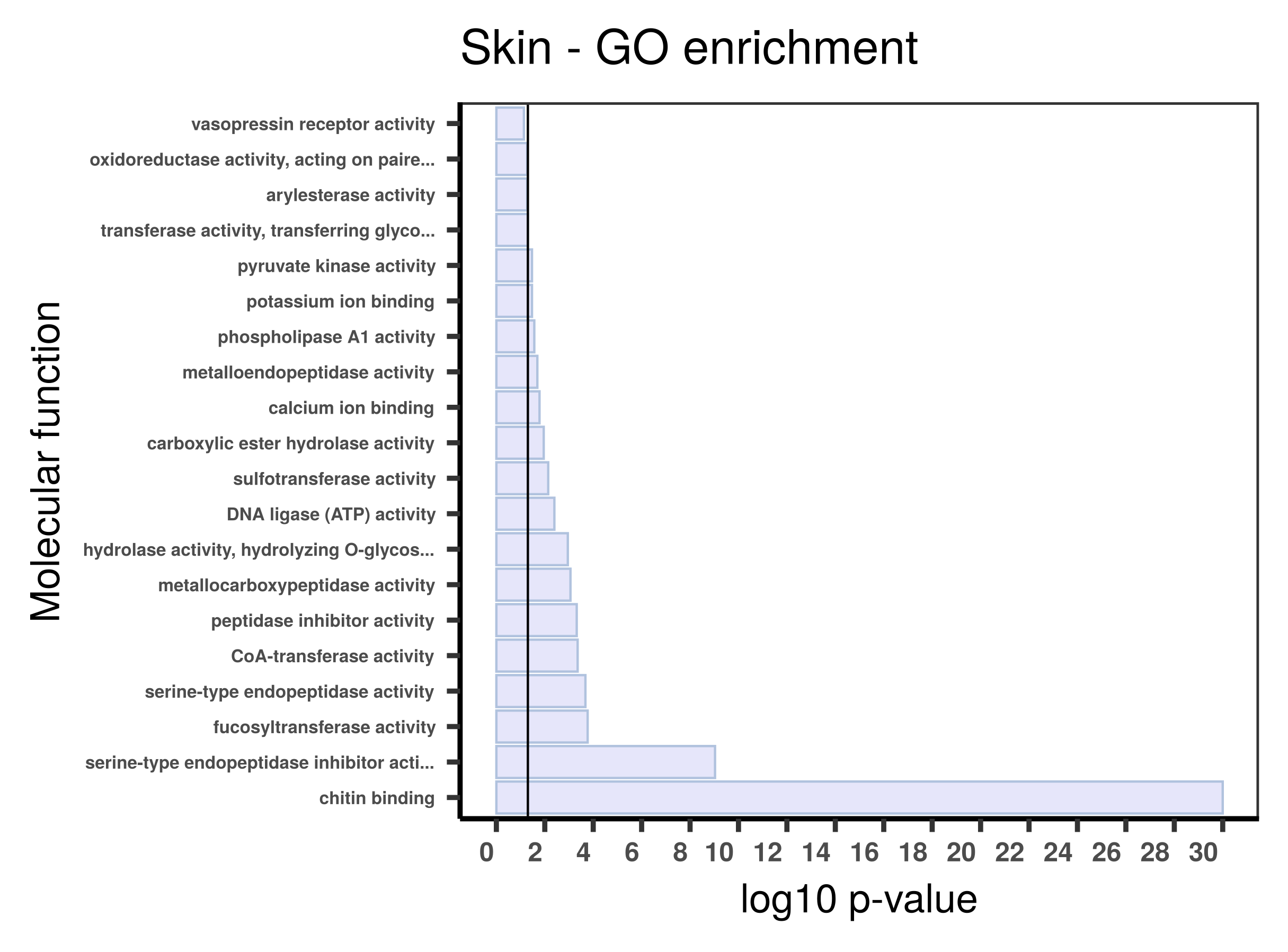

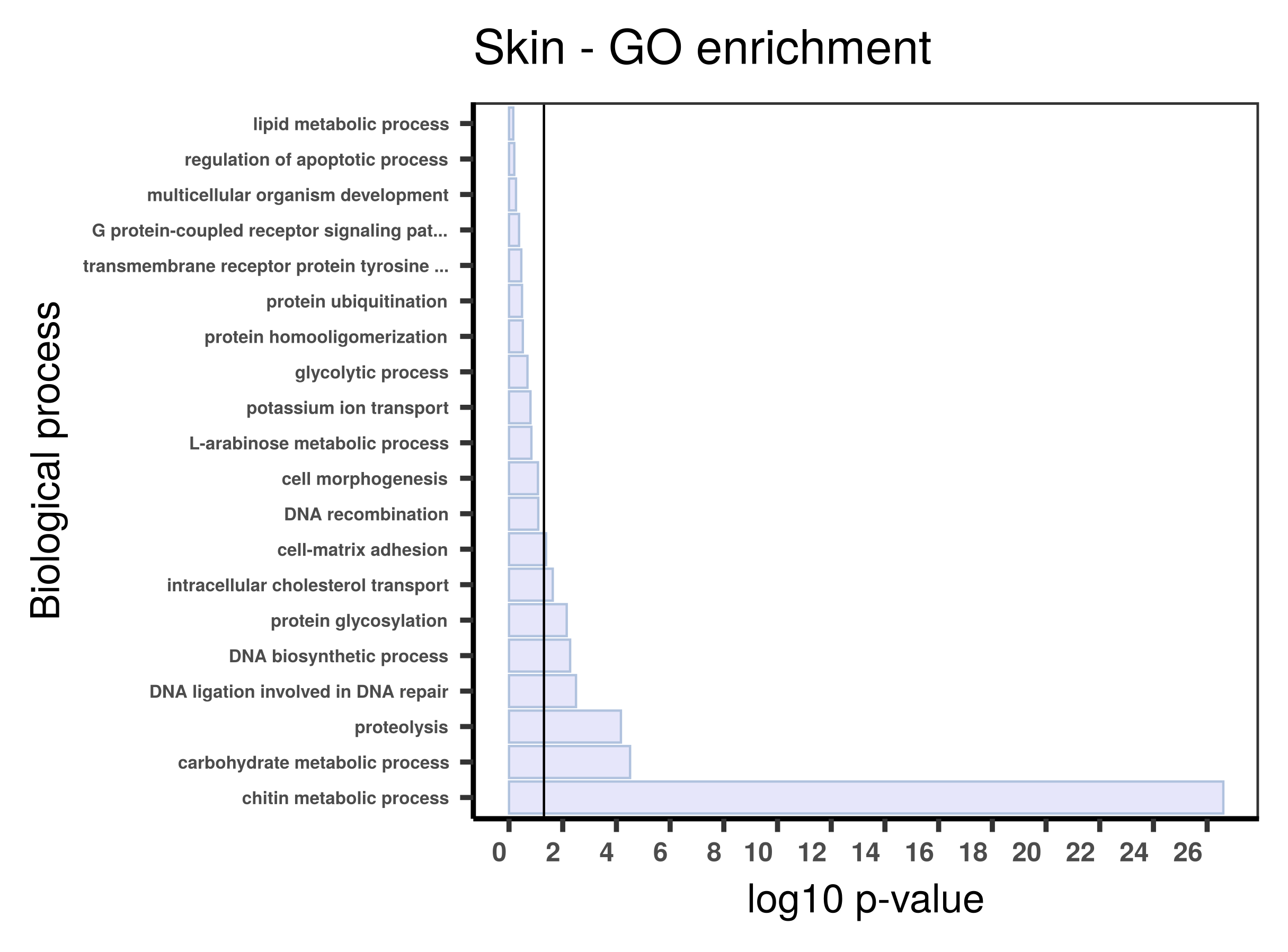


**
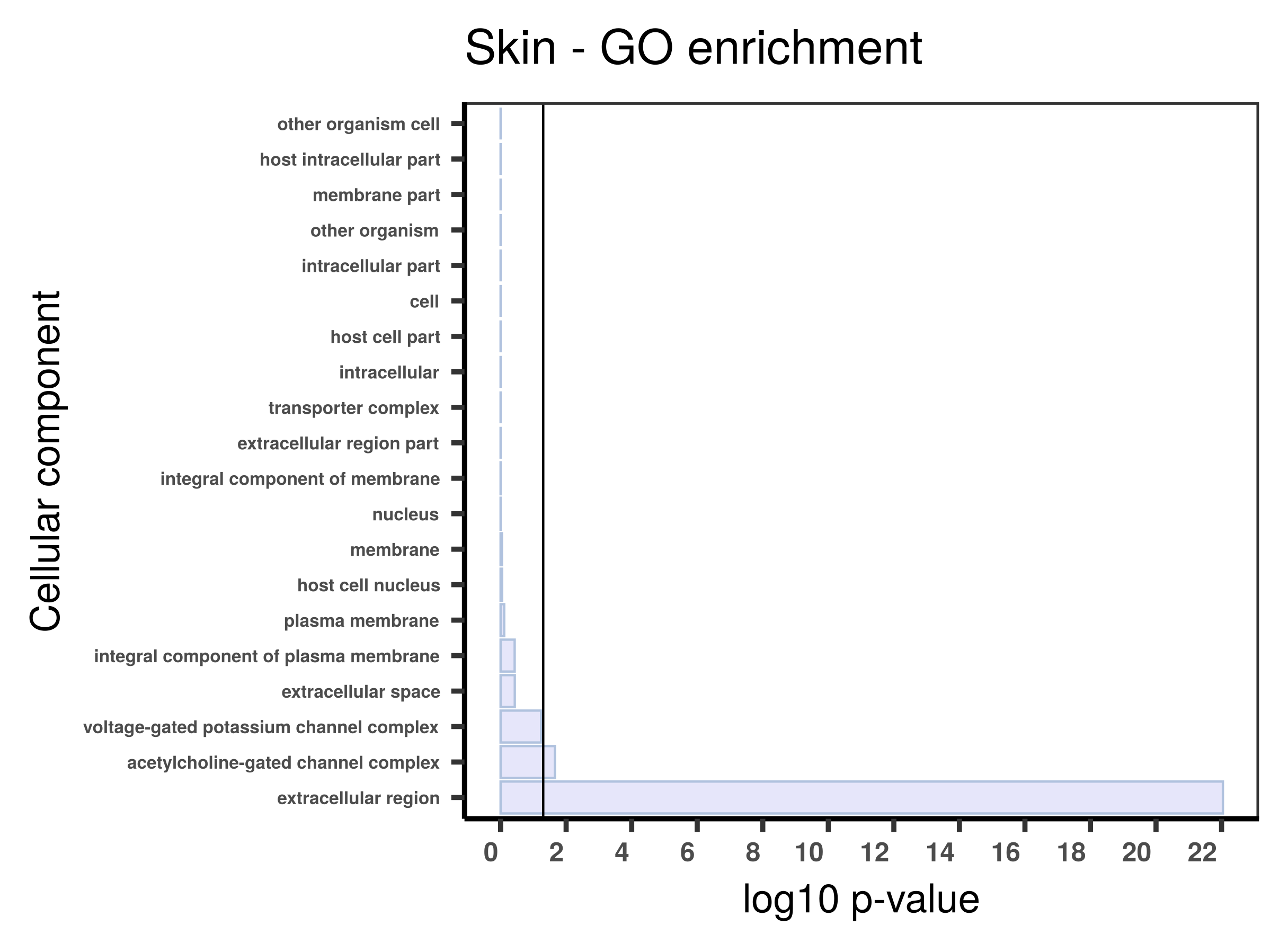
****Supplementary figure 26 | Gene set enrichment analysis with topGO using absolutely body wall (skin) specific TAU genes.** Gene ontology (GO) enrichment analyses for absolutely skin (body wall) specific TAU genes. The graphs correspond to the three domains of ontologies: biological process (BP), molecular function (MF) and cellular component (CC). The selected genes were analysed for enrichment in specific GO categories using the TopGO program against the background (all coding sequence genes). Y axis corresponds to enriched GO terms found in the respective domains (BP, MF and CC). X axis correspond to the log function of Fisher p-values obtained for each one of the enriched terms. The back line denotes a p-value = 0.05. P-values greater than 1,30 (log 0,05) indicate statistically significant enriched term. Genes involved chitin metabolism are differentially expressed in the body wall (skin).


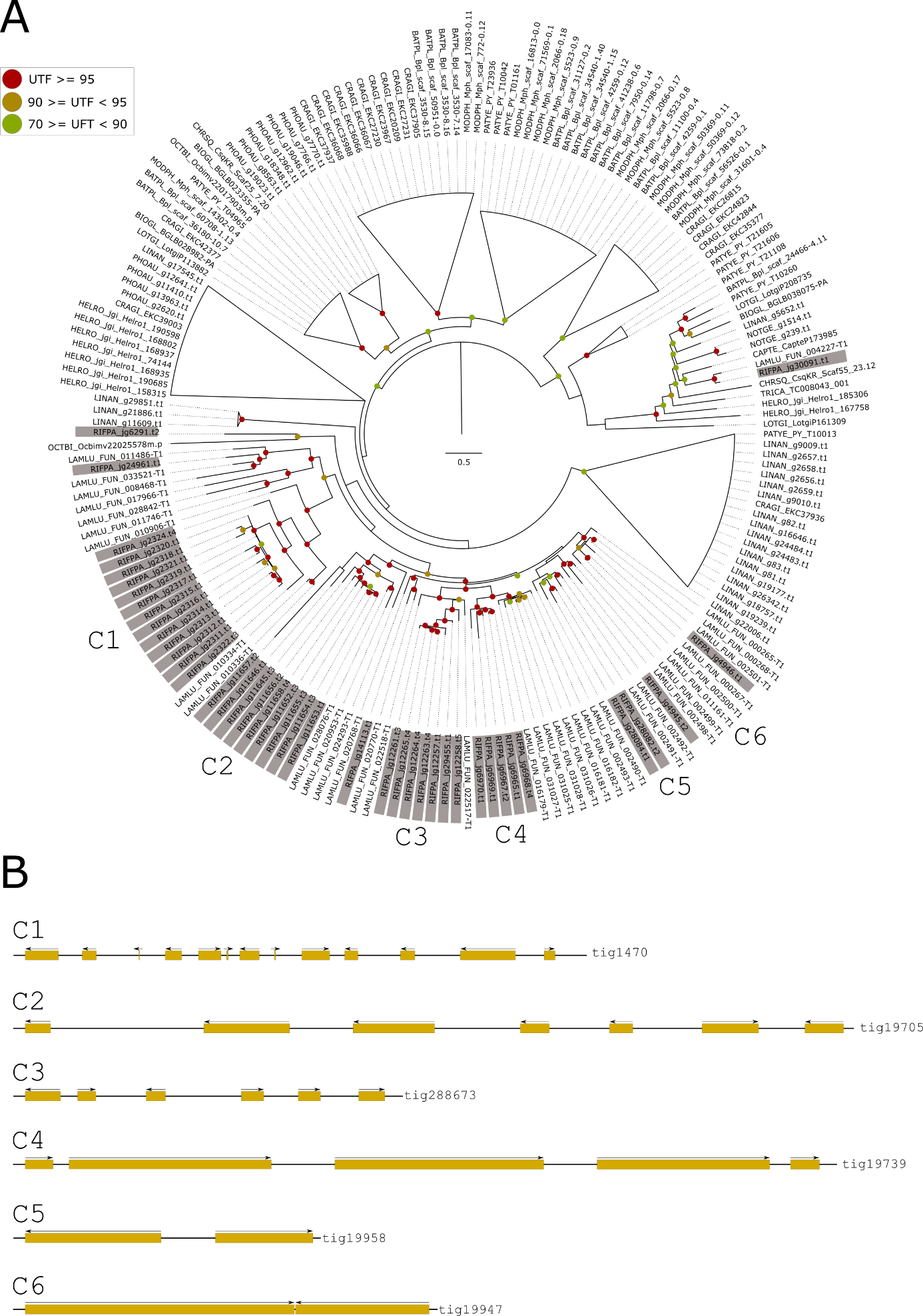


**Supplementary figure 27 | Phylogeny and genomic organisation of sushi genes in *Riftia pachyptila***. **A,** Maximum-likelihood phylogenetic tree inference of the sushi genes using 1000 ultrafast bootstrap replicates. The branch support values are represented by the coloured circles in the tree nodes. Red circles represent ultrafast bootstrap values >= 95. Yellow circles represent ultrafast bootstrap values >= 90 and < 95. Green circles represent ultrafast bootstrap values < 90 and >= 70. Ultrafast bootstrap values smaller than 70 are not shown. Gene identification is derived from the publicly available annotated lophotrochozoan genomes. *Riftia* contains the greatest number of sushi genes in the analysed lophotrochozoans. It is possible to note an independent expansion of sushi genes in the base of Vestimentifera (*Lamellibrachia* + *Riftia*). **B**, Genomic organisation of sushi genes in *Riftia* genome. Sushi genes are organised in six different genomic clusters possibly originated through a series of tandem duplications.


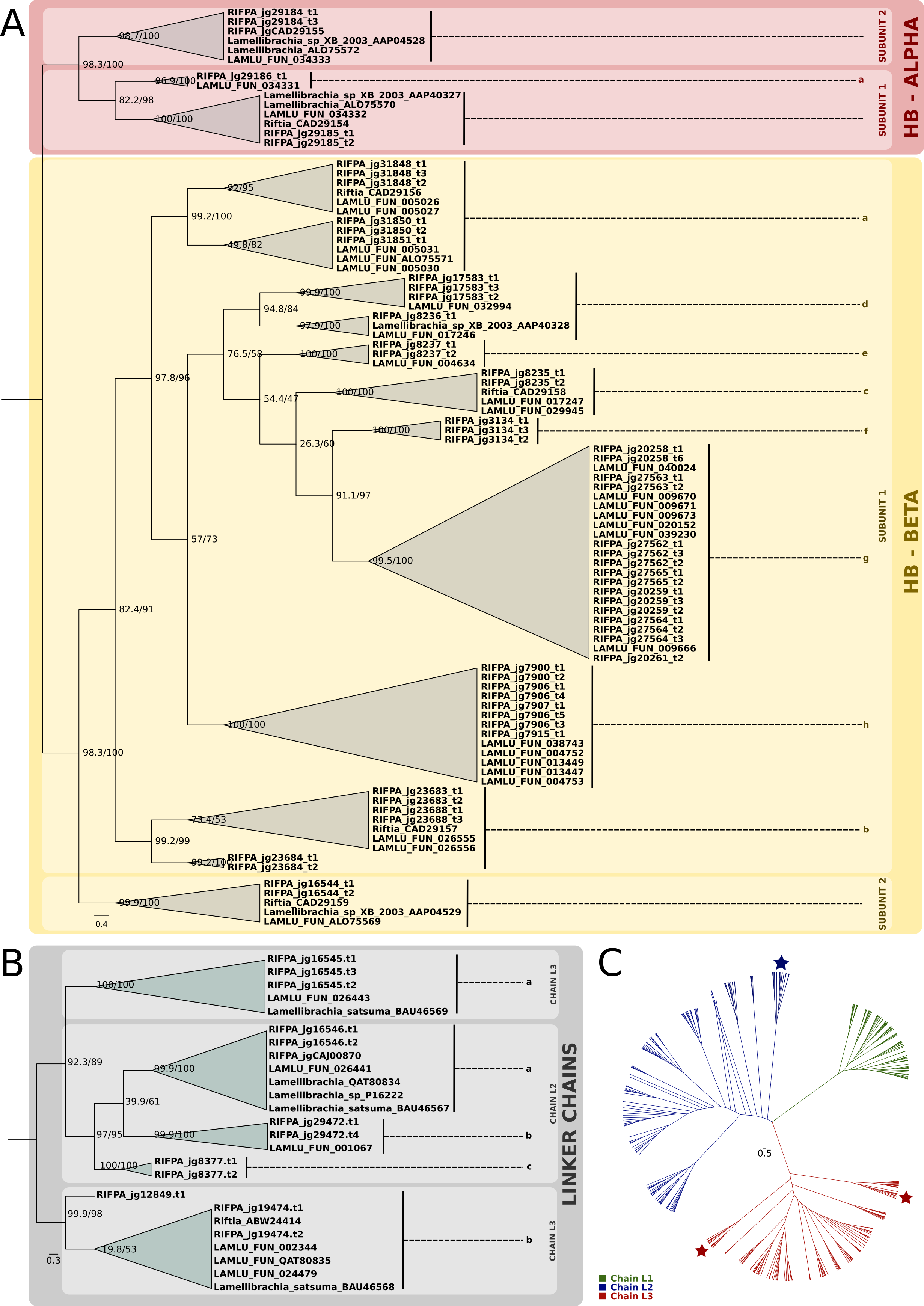


**Supplementary figure 28 | Phylogeny of Riftia and Lamellibrachia globin and linker genes**. **A,** Mid-rooted maximum-likelihood phylogenetic tree inference of the haemoglobin genes using 1000 ultrafast bootstrap replicates. Note the expansion of hemoglobin β1 genes in *Lamellibrachia* and *Riftia pachyptila*. **B,** Mid-rooted maximum-likelihood phylogenetic tree inference of the linker genes using 1000 ultrafast bootstrap replicates. **C** Unrooted maximum-likelihood phylogenetic tree inference of linker genes in a wide range of annelids and metazoans. *Riftia* linker genes belong to L3 and L2 family. *Lamellibrachia* gene identification is derived from the public annotated genome.


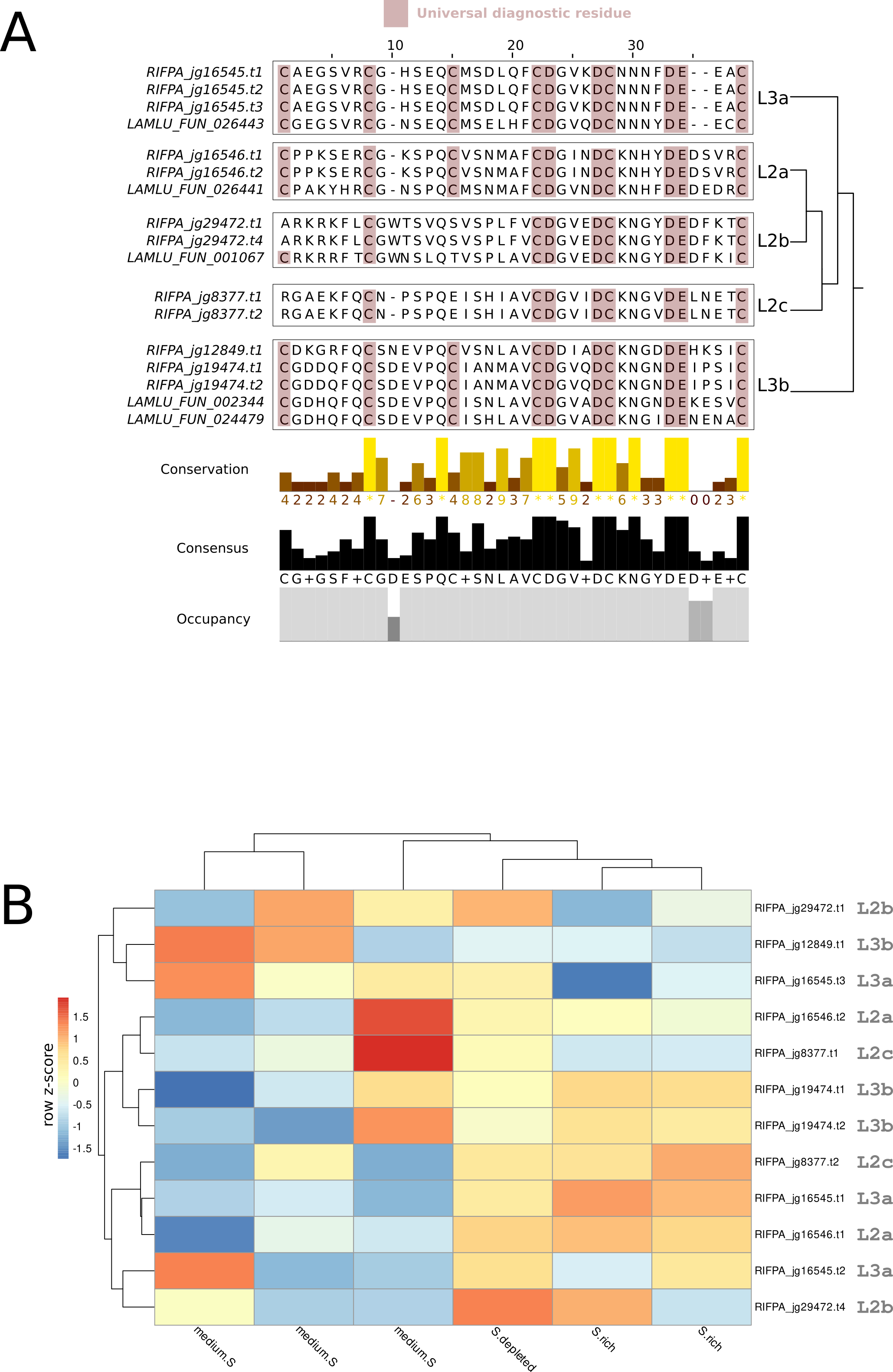


**Supplementary figure 29 | Multiple sequence alignments and gene expression of *Riftia* linker genes A,** Multiple sequence alignment, identification and characterisation of the signature diagnostic residues/motifs in the linker chains. Dark red boxes correspond to universal diagnostic residues. Histogram plots correspond to conservation and occupancy of each multiple sequence alignment column. **B,** Expression profile of linker genes in the trophosome *Riftia pachyptila*. Medium-Sulphide, Sulphide-Depleted and Sulphide-rich correspond to different environmental conditions. Colour coding reflects the expression patterns based on row Z-score calculations. Linker genes are constitutively expressed in all trophosome tissues, irrespective of the experimental conditions.

**
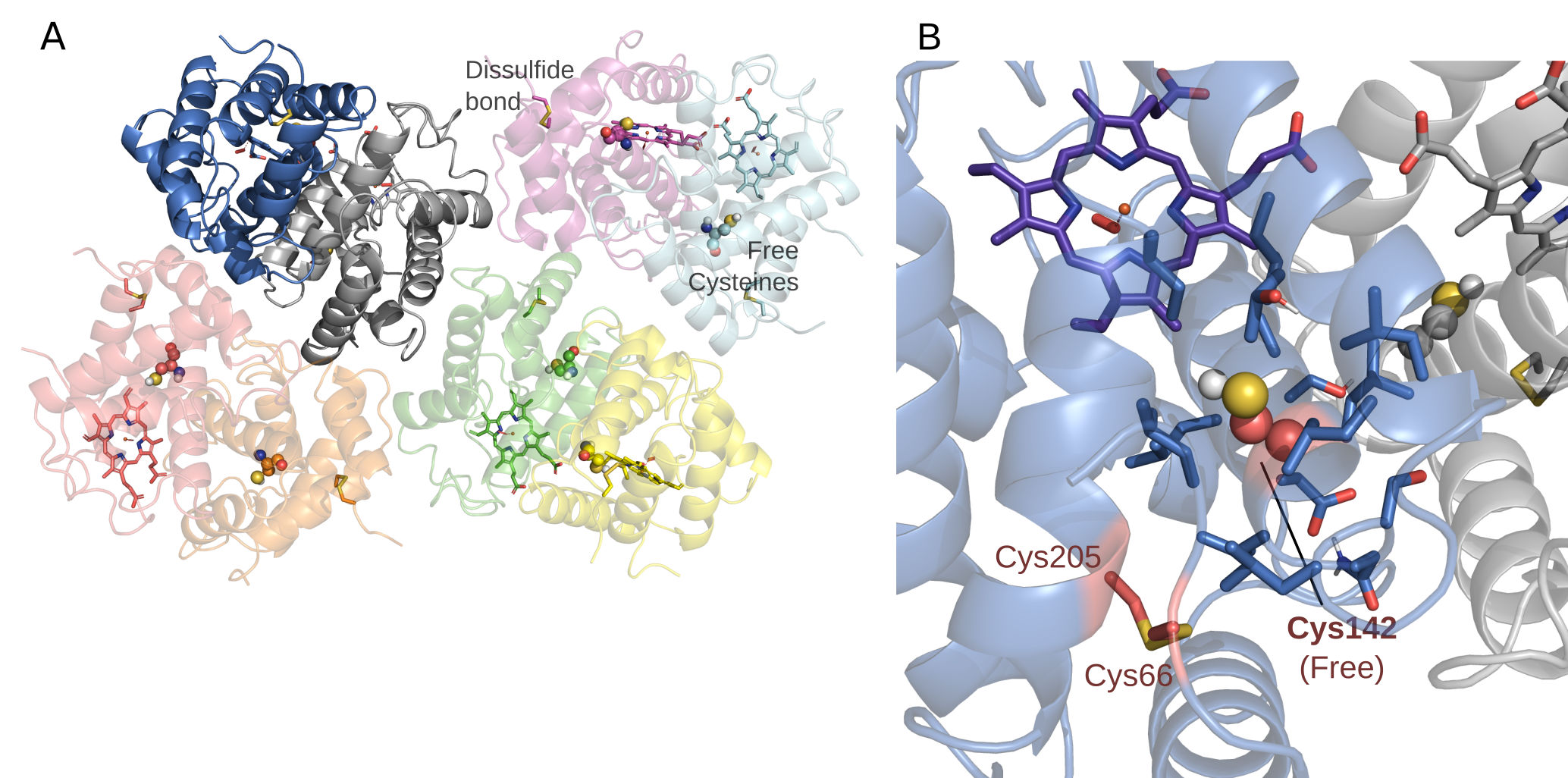
**

**Supplementary figure 30| Homology model generation for Riftia haemoglobin. A,** Haemoglobin octomeric structure of *Riftia* gene RIFPA_jg20259.t3 belonging to the β1c-Hb paralog group. **B,** Schematic representation of the putative free cysteine residue, hypothesized to bind toxic hydrogen sulphide (H2S).

**
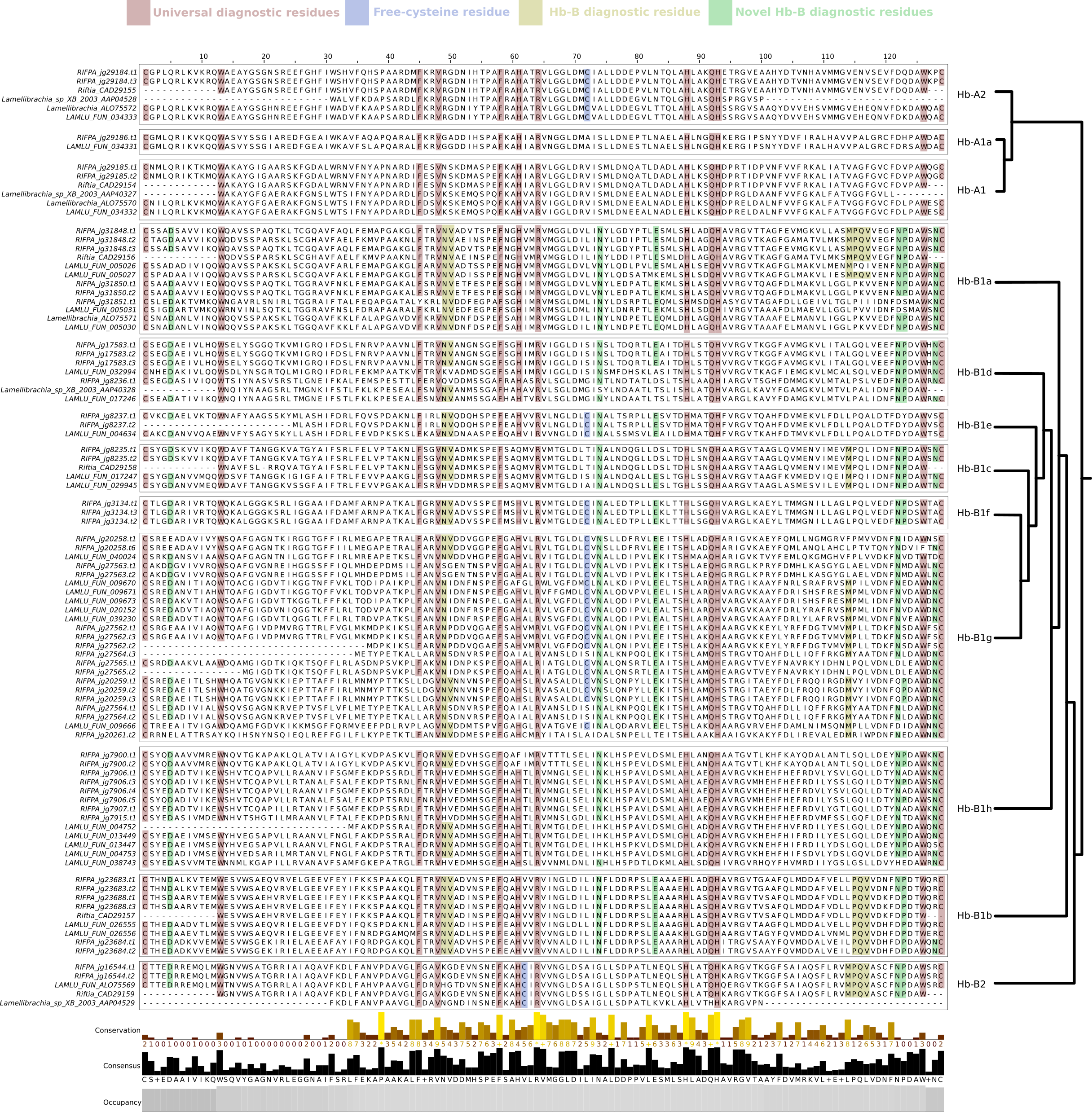
**

**Supplementary figure 31 | Multiple sequence alignment of *Riftia* and *Lamellibrachia* haemoglobin genes.** Multiple sequence alignment, identification, and characterisation of the signature diagnostic residues/motifs in the haemoglobin chains. Dark red boxes correspond to universal diagnostic residues; light purple to putative free cysteine residues; light yellow HB-β diagnostic residues; and finally, light green correspond to novel β chain residues identified in this study. Histogram plots correspond to conservation and occupancy of each multiple sequence alignment column. Putative free cysteine residues were identified in many newly described β1 genes.

**
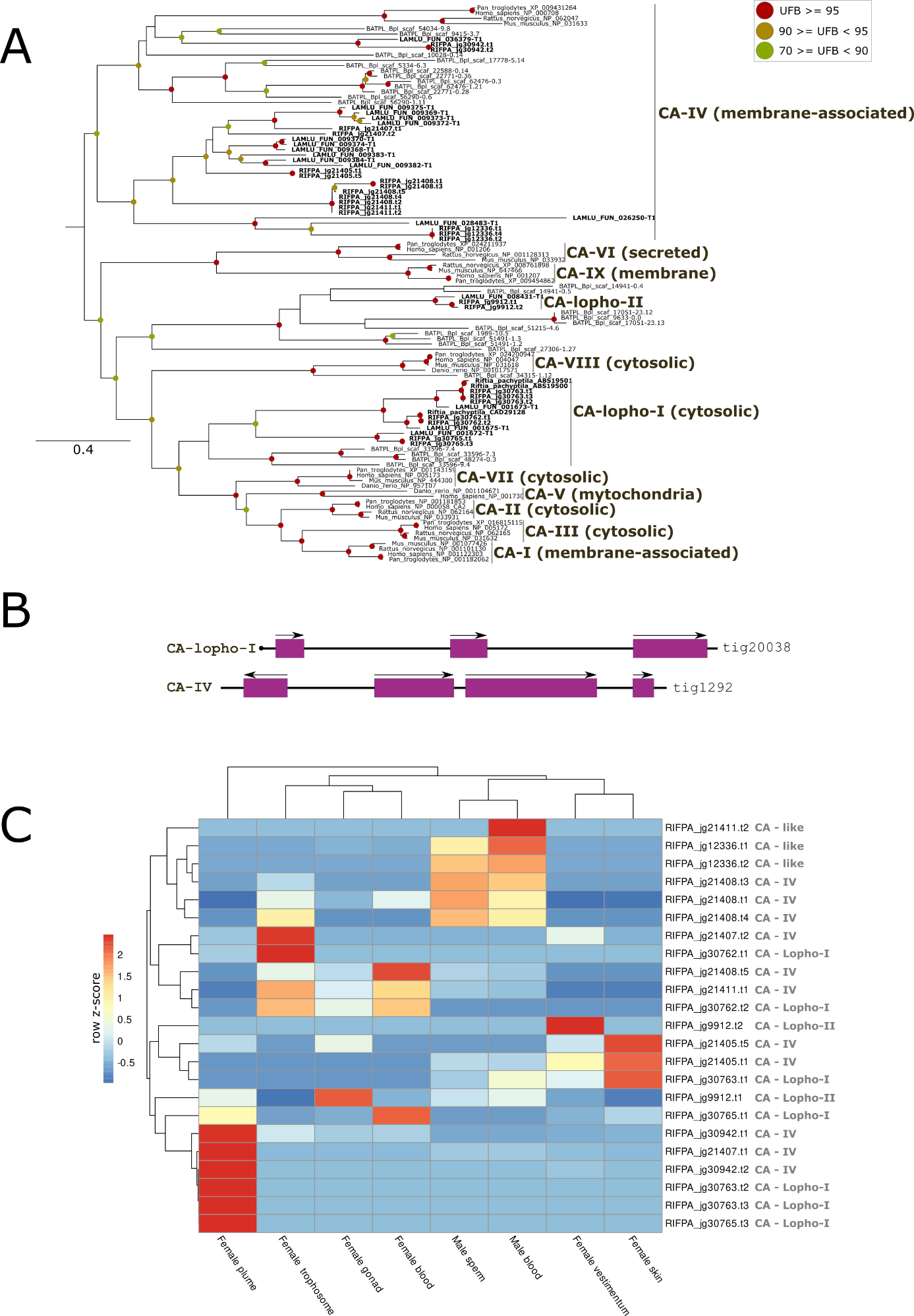
**

**Supplementary figure 32 | Carbonic anhydrase genes in *Riftia pachyptila***. **A**, Mid-rooted maximum-likelihood phylogenetic tree inference of the carbonic anhydrase genes using 1000 ultrafast bootstrap replicates. Red circles represent ultrafast bootstrap values >= 95. Yellow circles represent ultrafast bootstrap values >= 90 and < 95. Green circles represent ultrafast bootstrap values < 90 and >= 70. Ultrafast bootstrap values smaller than 70 are not shown. Gene identification is derived from the public annotated genome. **B,** Genomic organisation of seven CA gene in the giant tubeworm genome. **C,** Expression profile of carbonic anhydrase genes in eight different adult tissues of *Riftia pachyptila.*


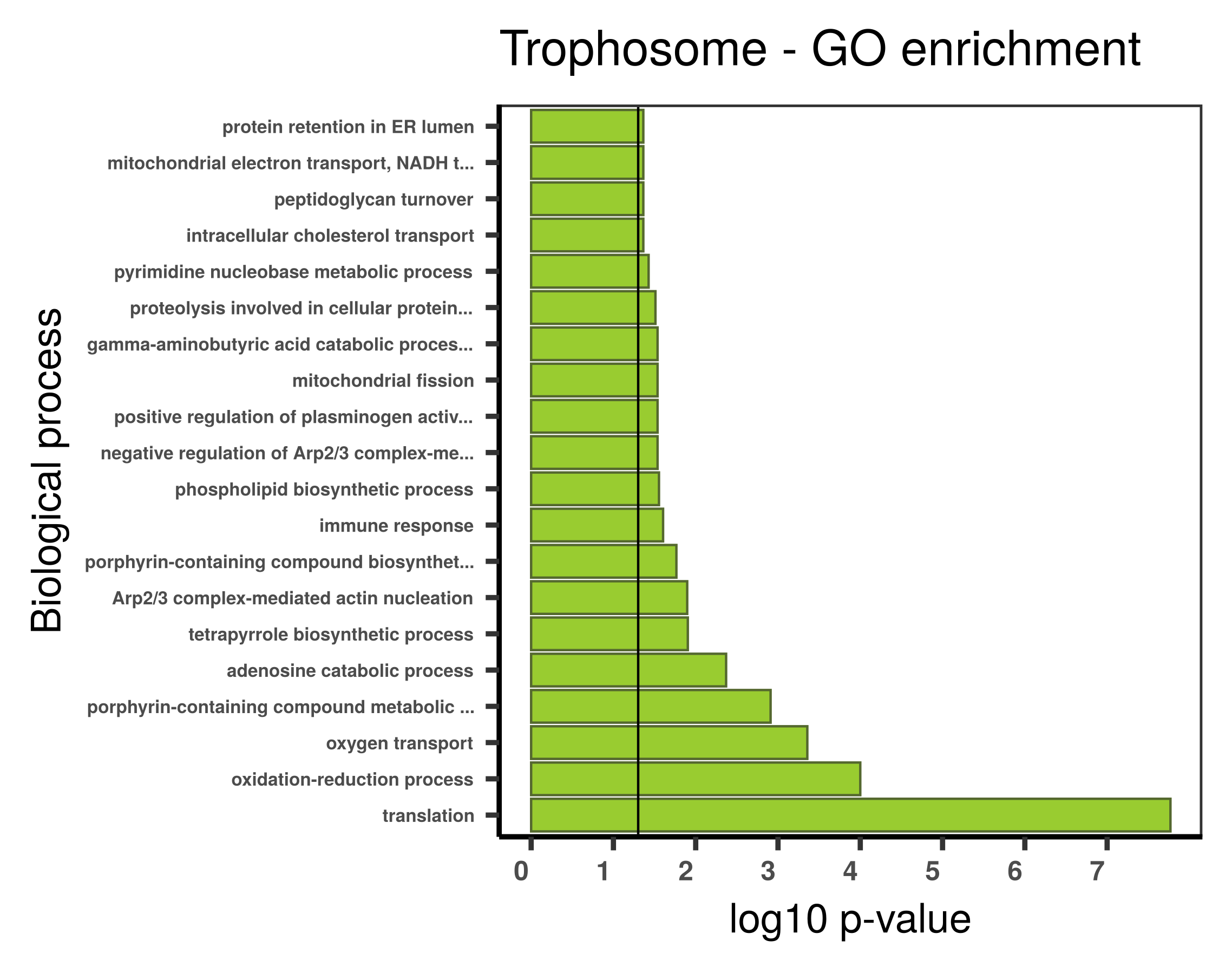


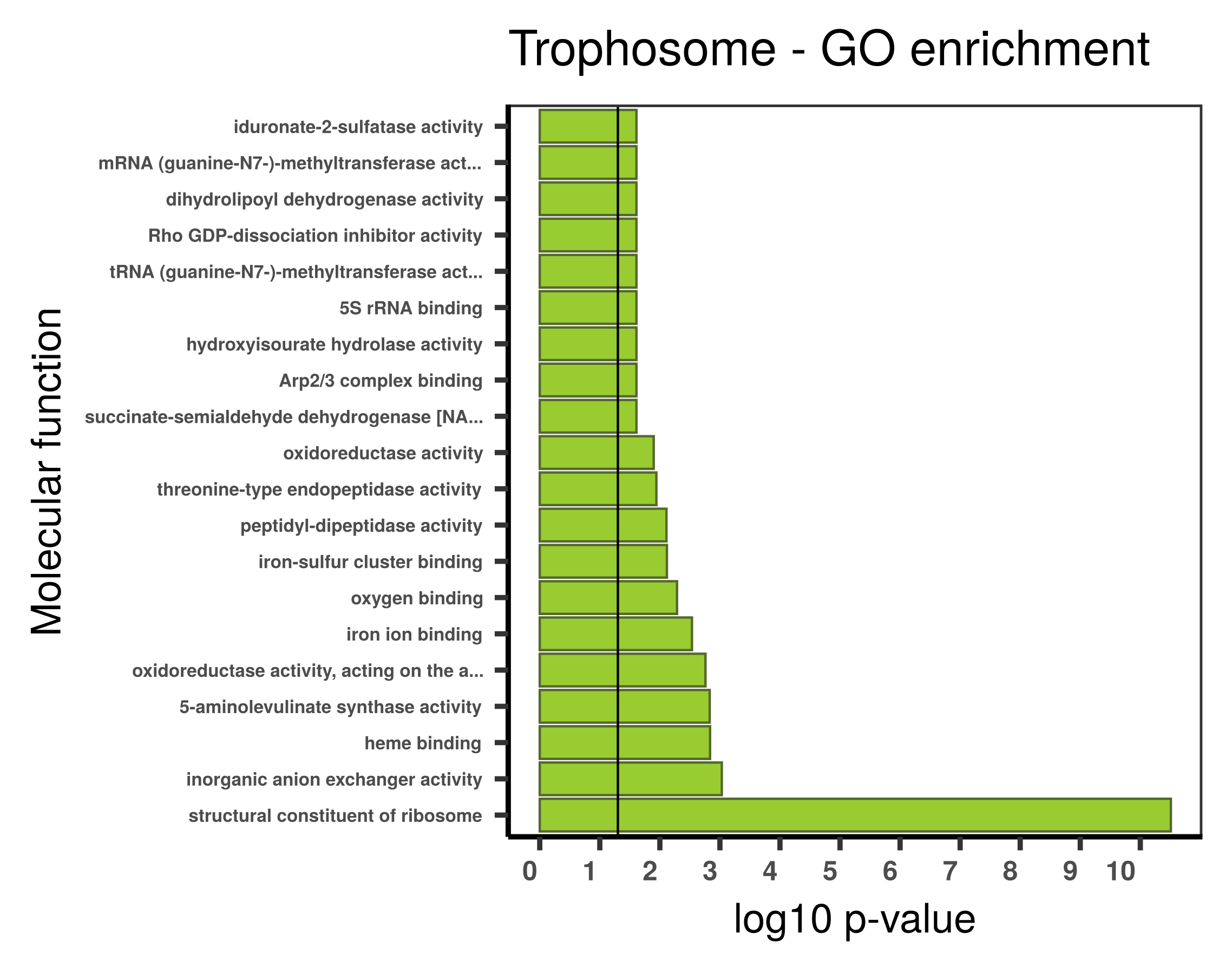


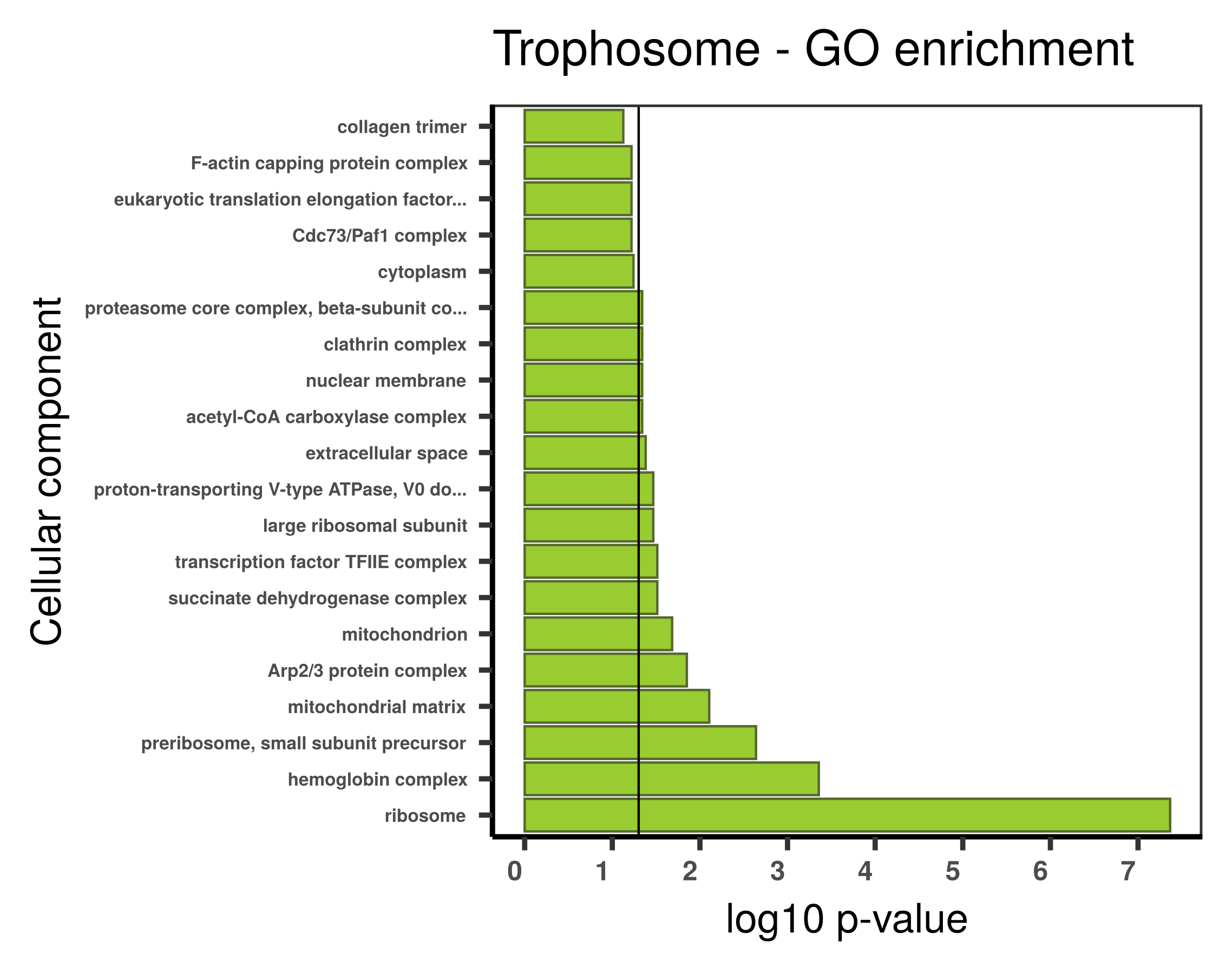


**Supplementary figure 33 | Gene set enrichment analysis with topGO using absolutely trophosome specific TAU genes.** Gene ontology (GO) enrichment analyses for absolutely trophosome specific TAU genes. The graphs correspond to the three domains of ontologies: biological process (BP), molecular function (MF) and cellular component (CC). The selected genes were analysed for enrichment in specific GO categories using the TopGO program against the background (all coding sequence genes). Y axis corresponds to enriched GO terms found in the respective domains (BP, MF and CC). X axis correspond to the log function of Fisher p-values obtained for each one of the enriched terms. The back line denotes a p-value = 0.05. P-values greater than 1,30 (log 0,05) indicate statistically significant enriched term. Genes involved chitin metabolism are differentially expressed in the body wall (skin).


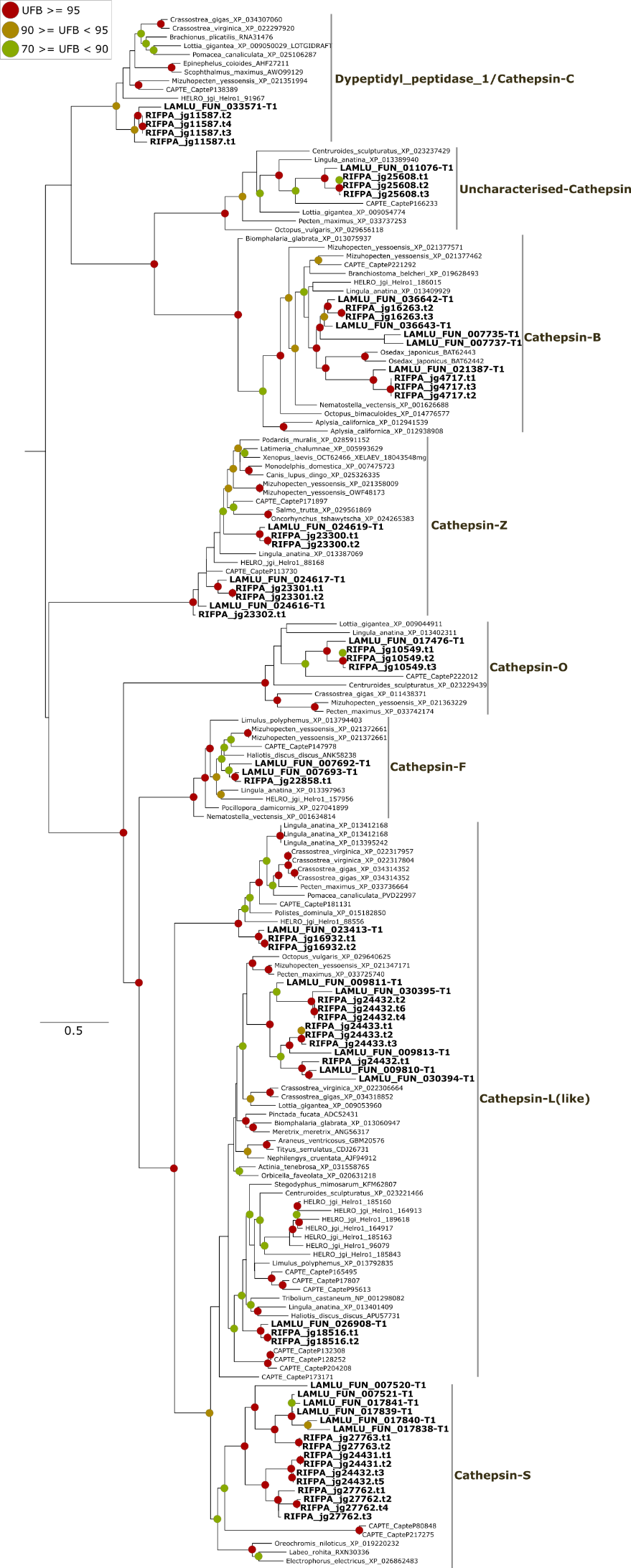


**Supplementary figure 34 | Phylogeny of cathepsins.** Mid-rooted maximum-likelihood phylogenetic tree inference of the cathepsin genes using 1000 ultrafast bootstrap replicates. The branch support values are represented by the coloured circles in the tree nodes. Red circles represent ultrafast bootstrap values >= 95. Yellow circles represent ultrafast bootstrap values >= 90 and < 95. Green circles represent ultrafast bootstrap values < 90 and >= 70. Ultrafast bootstrap values smaller than 70 are not shown. Accession numbers for NCBI database are displayed after the species names. *Capitella, Helobdella* and *Lamellibrachia* gene identification are derived from the publicly available annotated genomes.


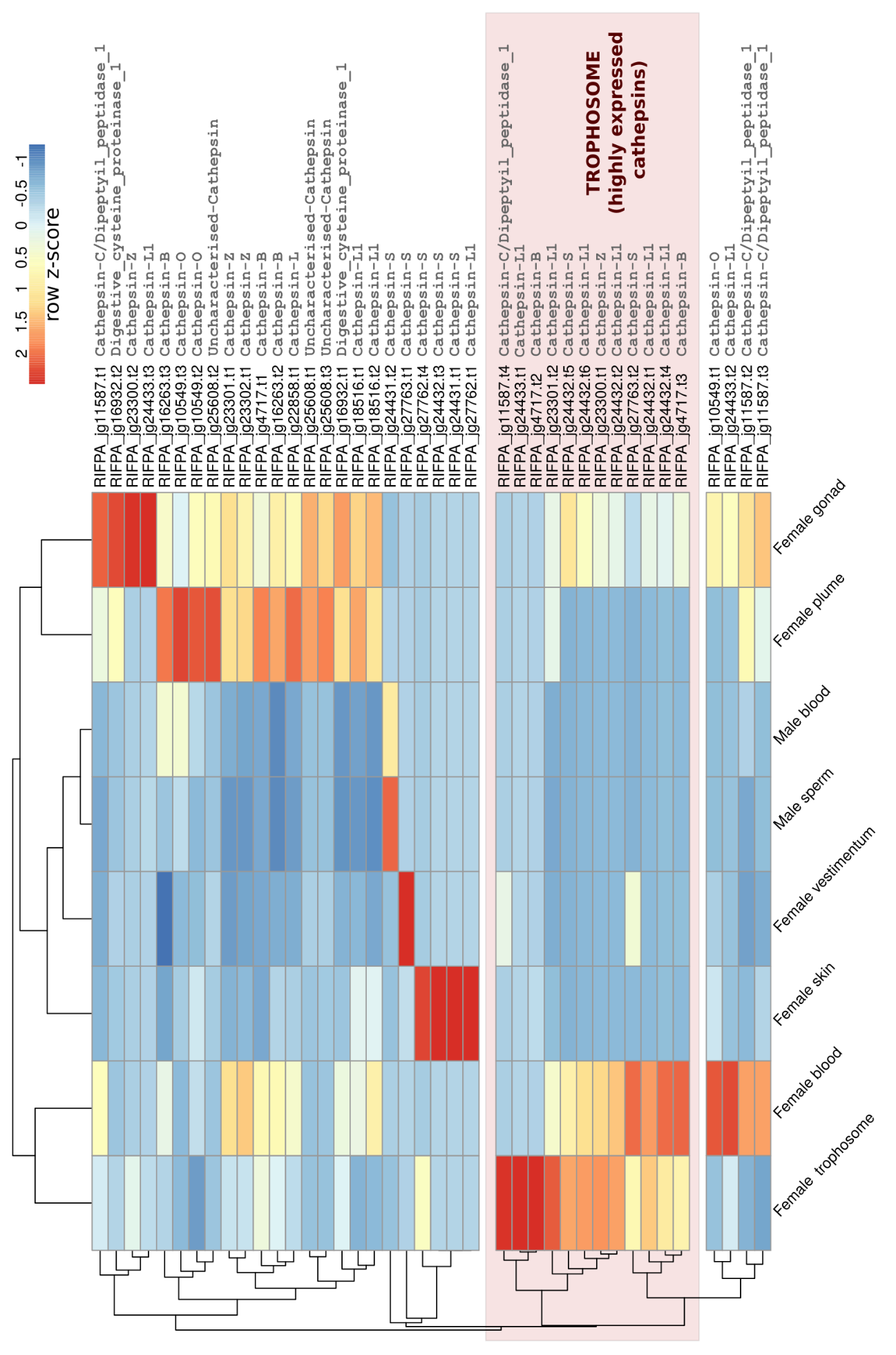


**Supplementary figure 35 | Gene expression of cathepsins.** Gene expression of cathepsin genes in eight adult tissues of *Riftia pachyptila*. Colour coding reflects the expression patterns based on row Z-score calculations. Cathepsins highly expressed in the trophosome are highlighted in light red.


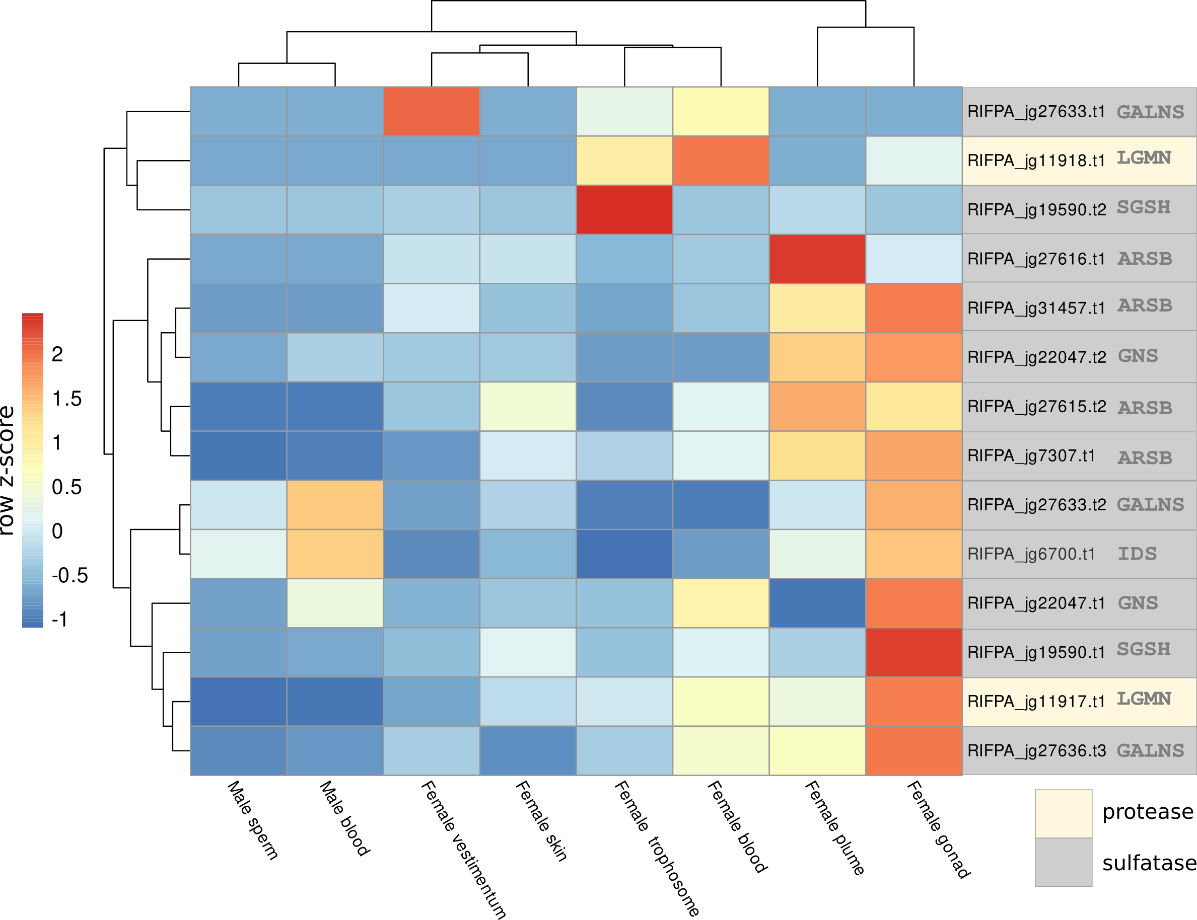


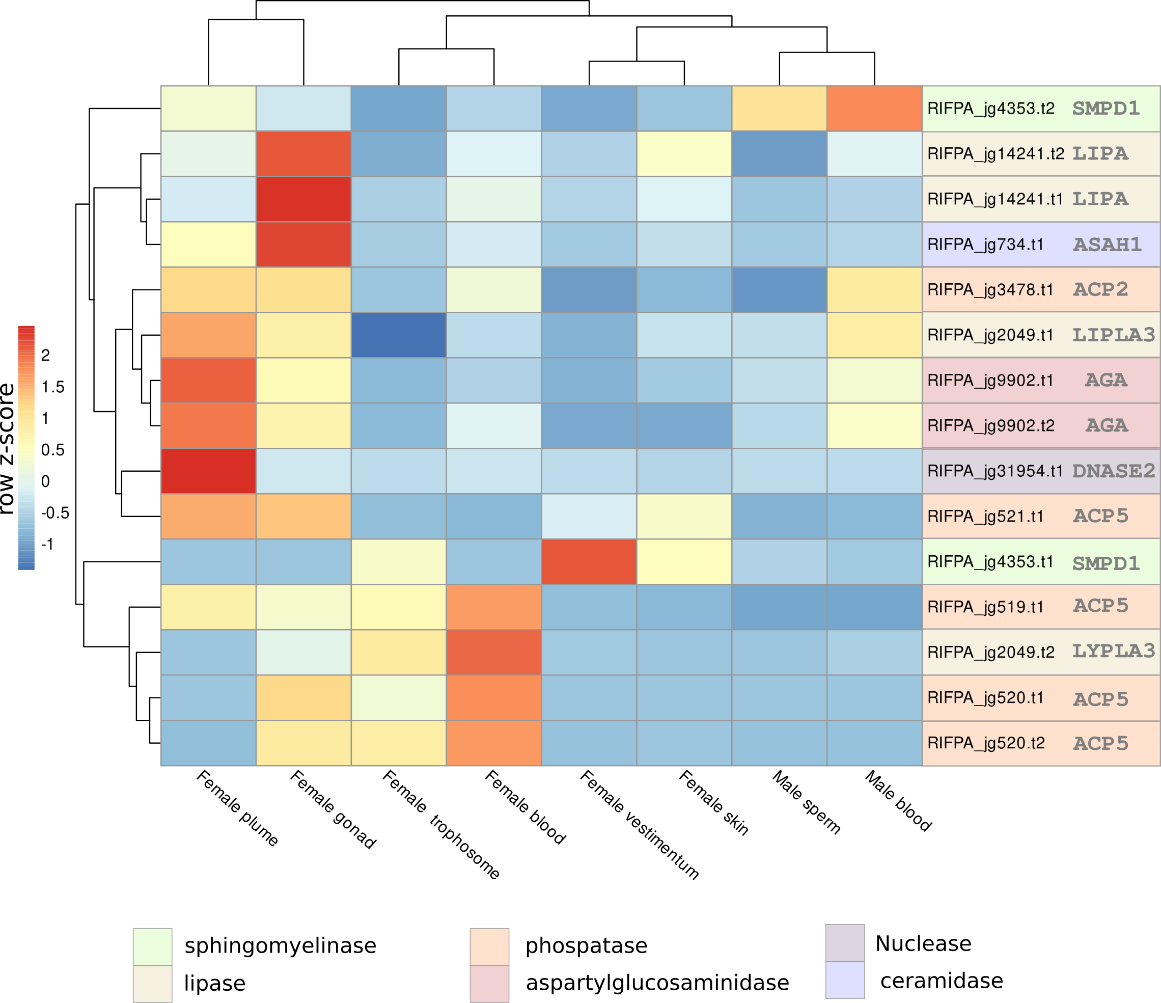


**Supplementary figure 36 | Gene expression of lysosomal-associated hydrolases.** Gene expression of lysosomal-associated hydrolase genes based on KEGG results. Genes identified on the KEGG orthology lysosome pathway (Lysosome 04142 – PATH:ko04142) were used to calculate the gene expression across the adult tubeworm tissues. Colour coding reflects the expression patterns based on row Z-score calculations.


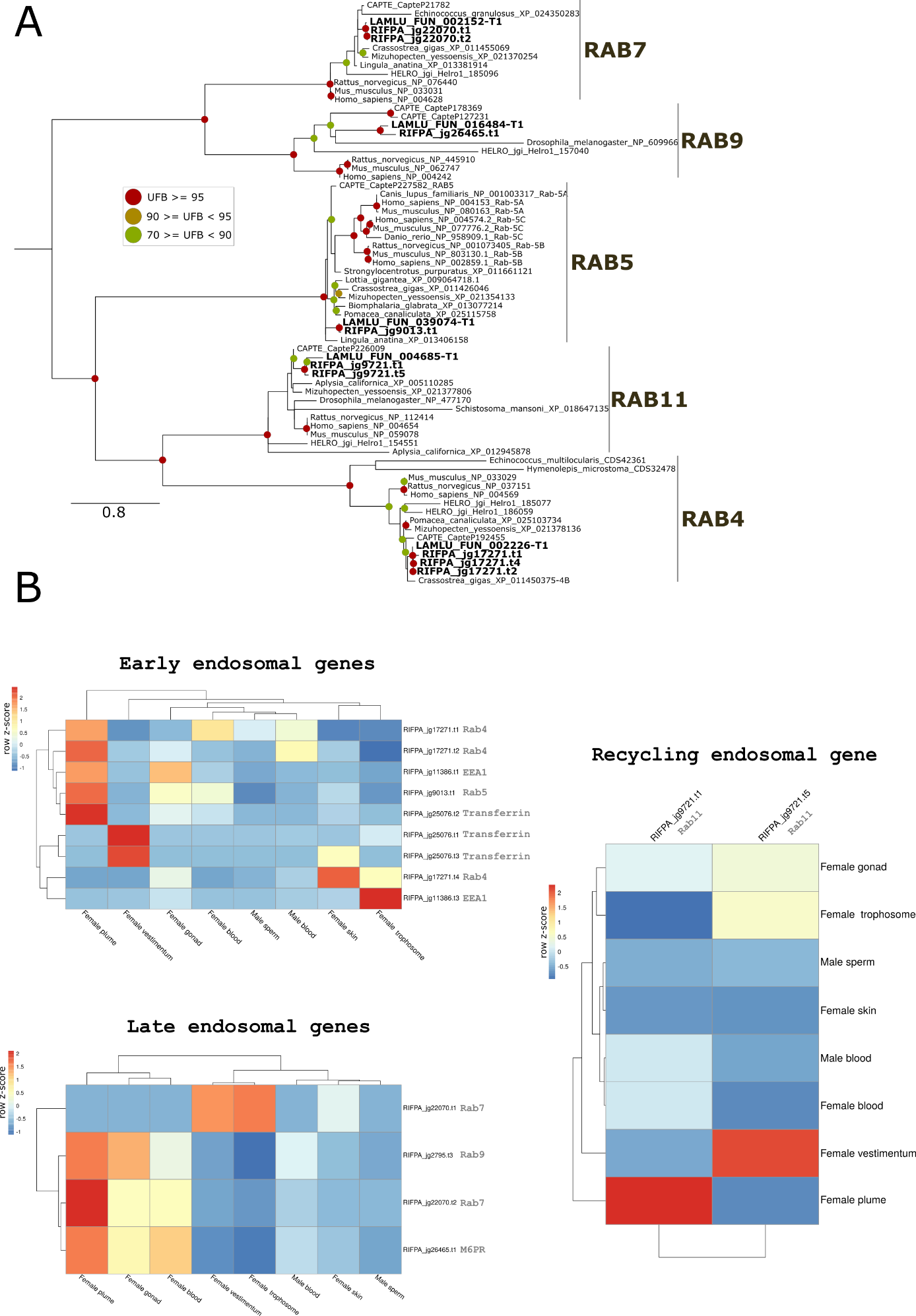


**Supplementary figure 37 | Phylogeny and gene expression of endosomal genes**. A, Mid-rooted maximum-likelihood phylogenetic tree inference of the endosomal genes using 1000 ultrafast bootstrap replicates. The branch support values are represented by the coloured circles in the tree nodes. Red circles represent ultrafast bootstrap values >= 95. Yellow circles represent ultrafast bootstrap values >= 90 and < 95. Green circles represent ultrafast bootstrap values < 90 and >= 70. Ultrafast bootstrap values smaller than 70 are not shown. Accession numbers for NCBI database are displayed after the species names. *Capitella*, *Helobdella* and *Lamellibrachia* gene identification are derived from the publicly available annotated genomes. B, Expression profile of early, late and recycling endosomal genes. Colour coding reflects the expression patterns based on row Z-score calculations.

**
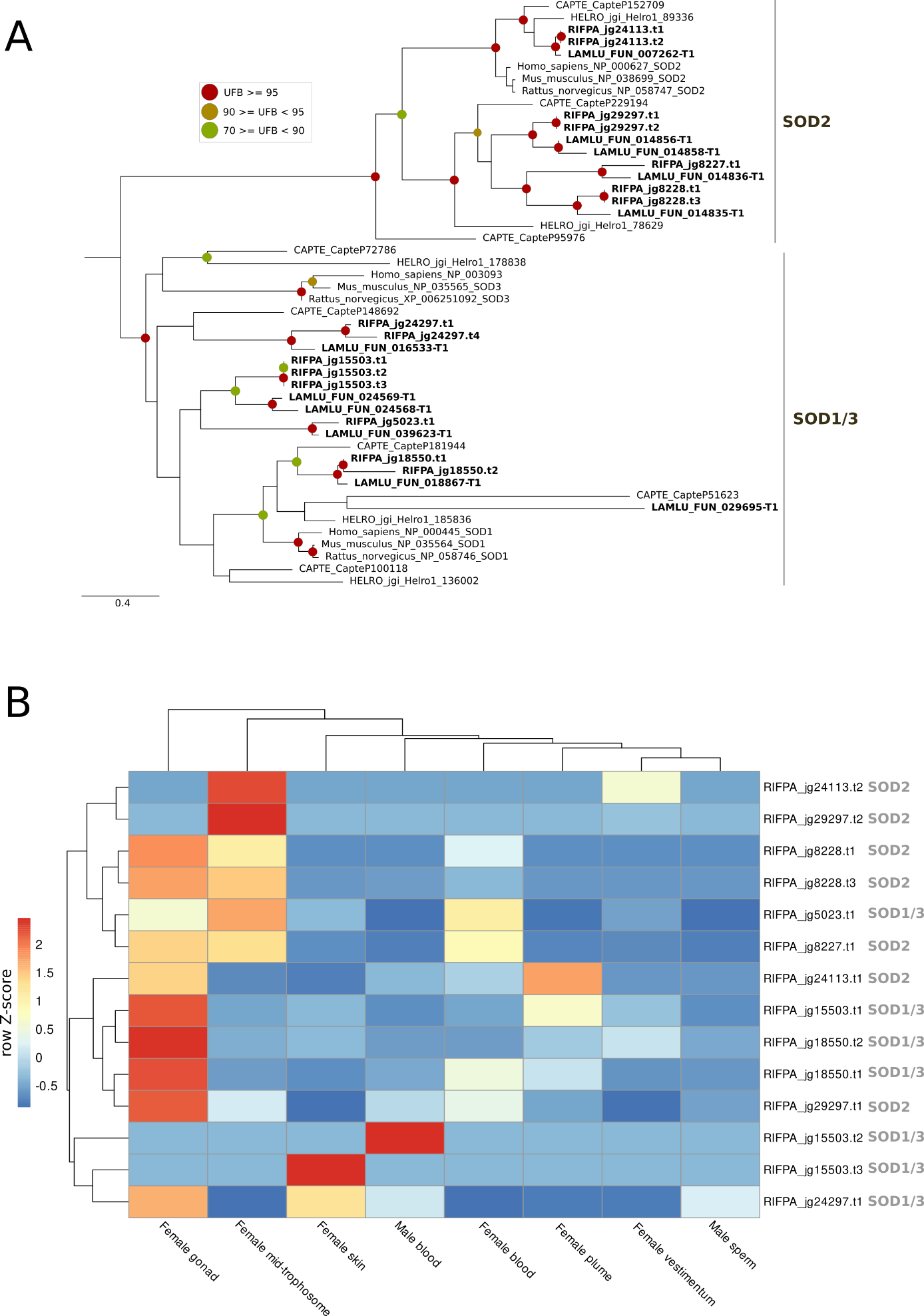
**

**Supplementary figure 38 | Phylogeny and gene expression of SOD genes**. **A.** Mid-rooted maximum-likelihood phylogenetic tree inference of the SOD genes using 1000 ultrafast bootstrap replicates. The branch support values are represented by the coloured circles in the tree nodes. Red circles represent ultrafast bootstrap values >= 95. Yellow circles represent ultrafast bootstrap values >= 90 and < 95. Green circles represent ultrafast bootstrap values < 90 and >= 70. Ultrafast bootstrap values smaller than 70 are not shown. Accession numbers for NCBI database are displayed after the species names. *Capitella, Helobdella* and *Lamellibrachia* gene identification are derived from the publicly available annotated genomes. **B,** Expression profile of SOD genes. Colour coding reflects the expression patterns based on row Z-score calculations. SOD genes are highly expressed in the trophosome indicative of oxidative stress.


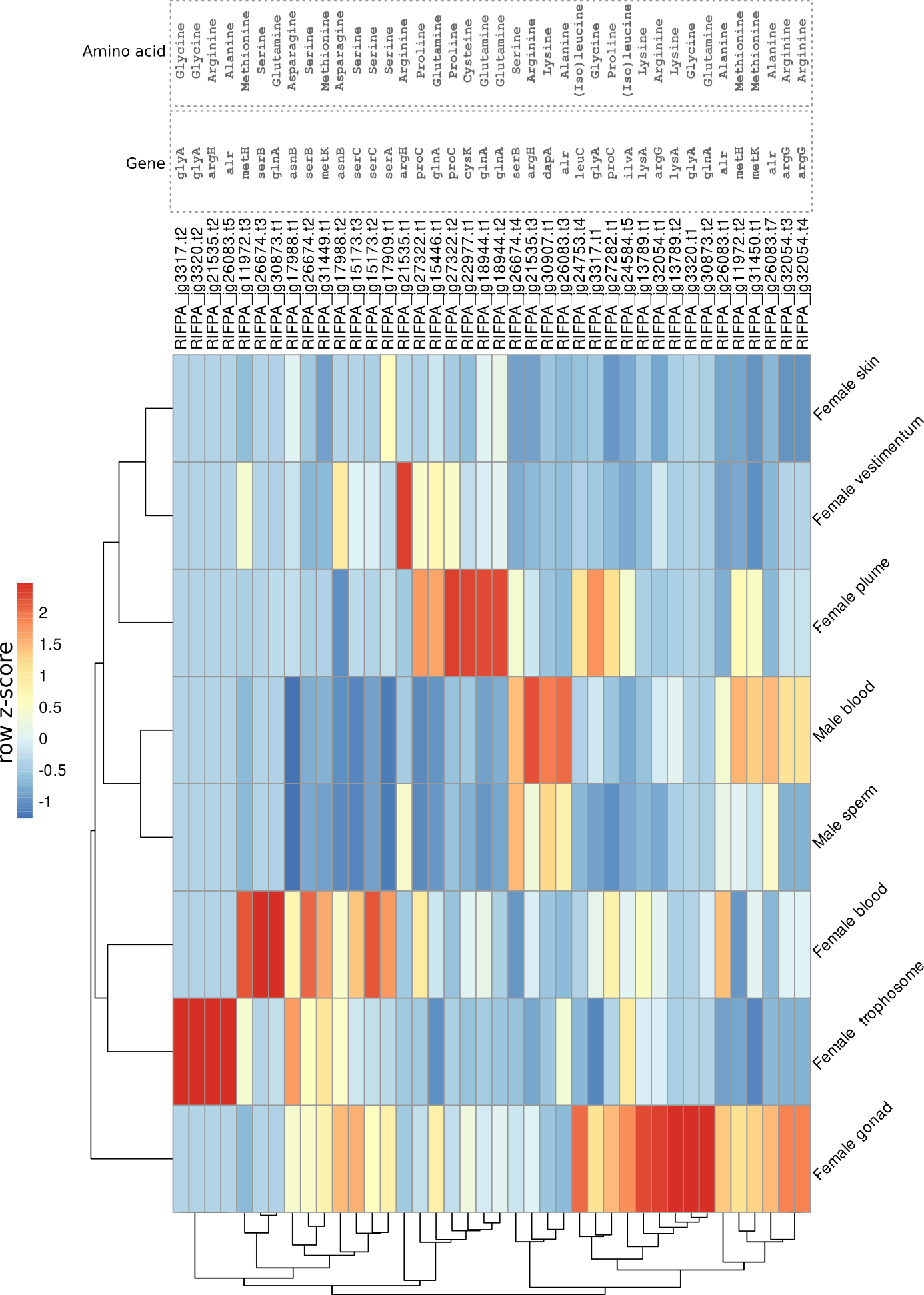


**Supplementary figure 39 | Gene expression of enzymes related to amino acid biosynthesis.** Gene expression of key enzymes related to amino acid biosynthesis genes based on KEGG results (check Supplementary Table 10). Colour coding reflects the expression patterns based on row Z-score calculations. Enzymes related to arginine and glycine metabolism are highly expressed in the trophosome.


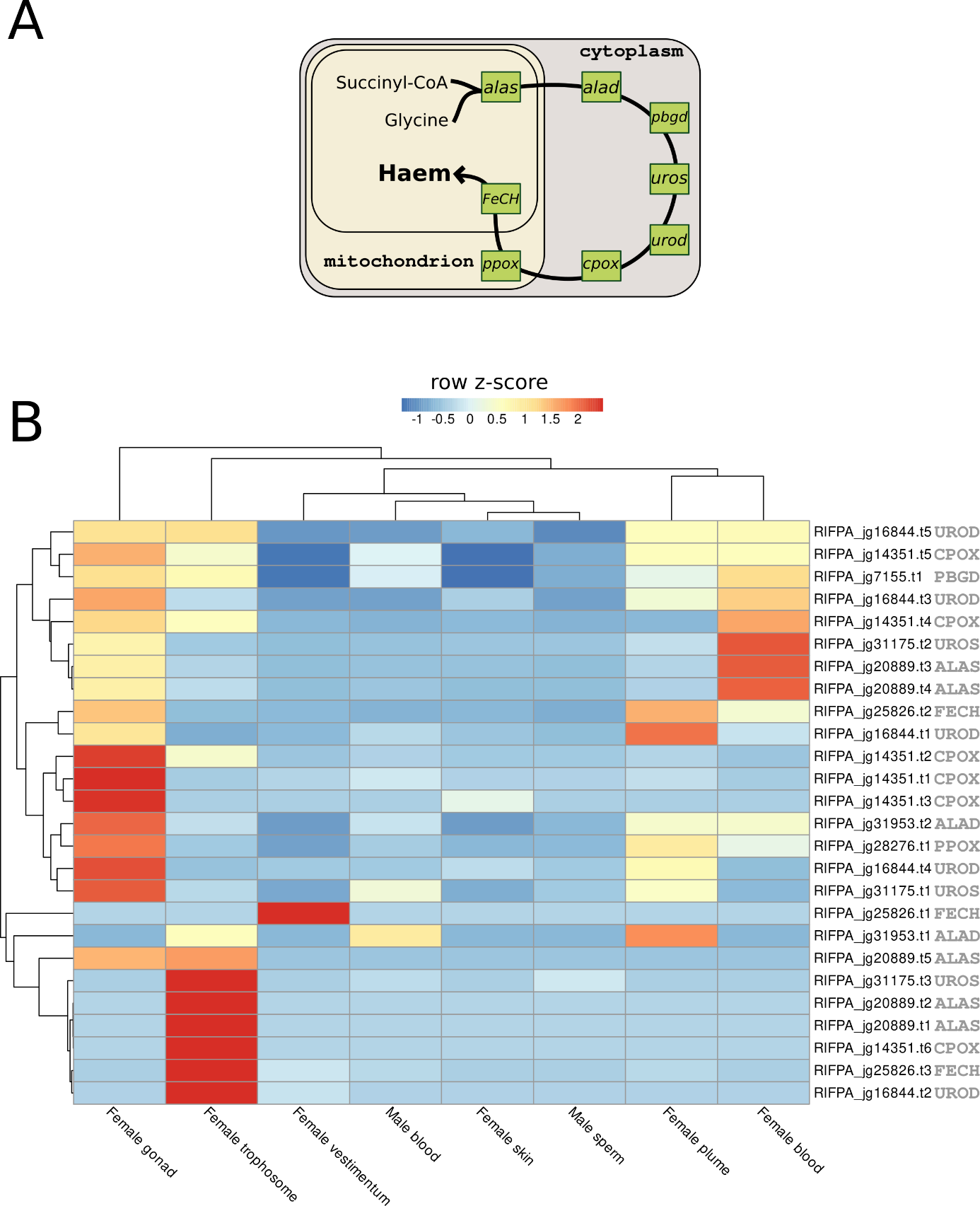


**Supplementary figure 40 | Gene expression of enzymes related to haem biosynthesis**. **A**, Scheme representing the seven conserved enzymes required for haem synthesis (adapted from Kořený et al., 2013). All enzymes were identified in the giant tubeworm genome. **B,** Gene expression of key enzymes related haem synthesis. Colour coding reflects the expression patterns based on row Z-score calculations. Enzymes related to haem synthesis are highly expressed in the trophosome suggesting that this tissue is involved with haematopoiesis in *Riftia*.

**
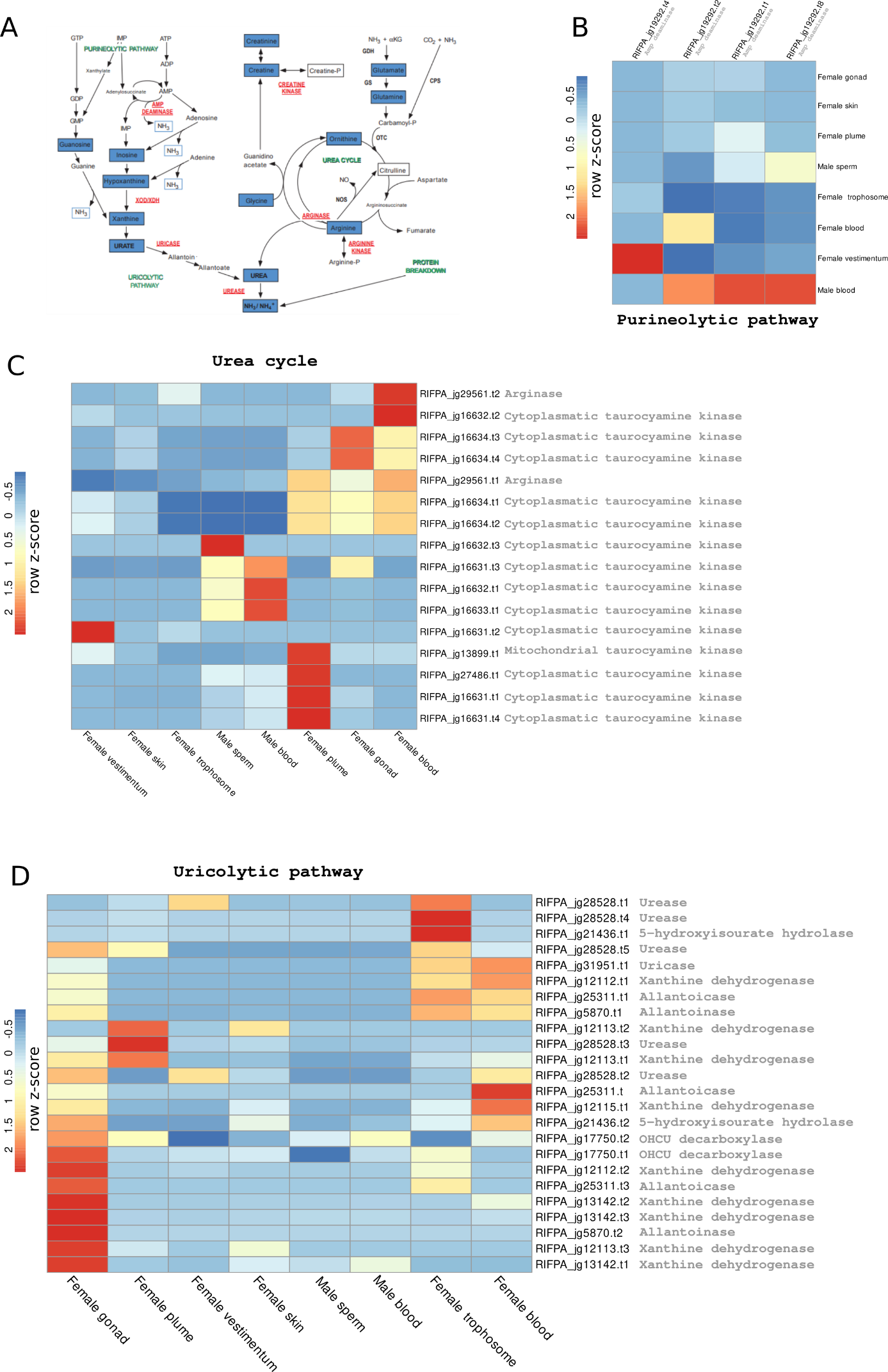
****Supplementary figure 41 | Overview and gene expression of genes involved in the nitrogen metabolism in *Riftia*.** **A,** Nitrogen metabolism and pathways leading to the main nitrogeneous end-products (obtained from Cian et al., 2000). **B – D,** Expression profile of different key enzymes related to the purineolytic, uricolytic and urea pathways. Colour coding reflects the expression patterns based on row Z-score calculations. Genes involved in the uricolytic pathway are highly expressed in the trophosome.

**
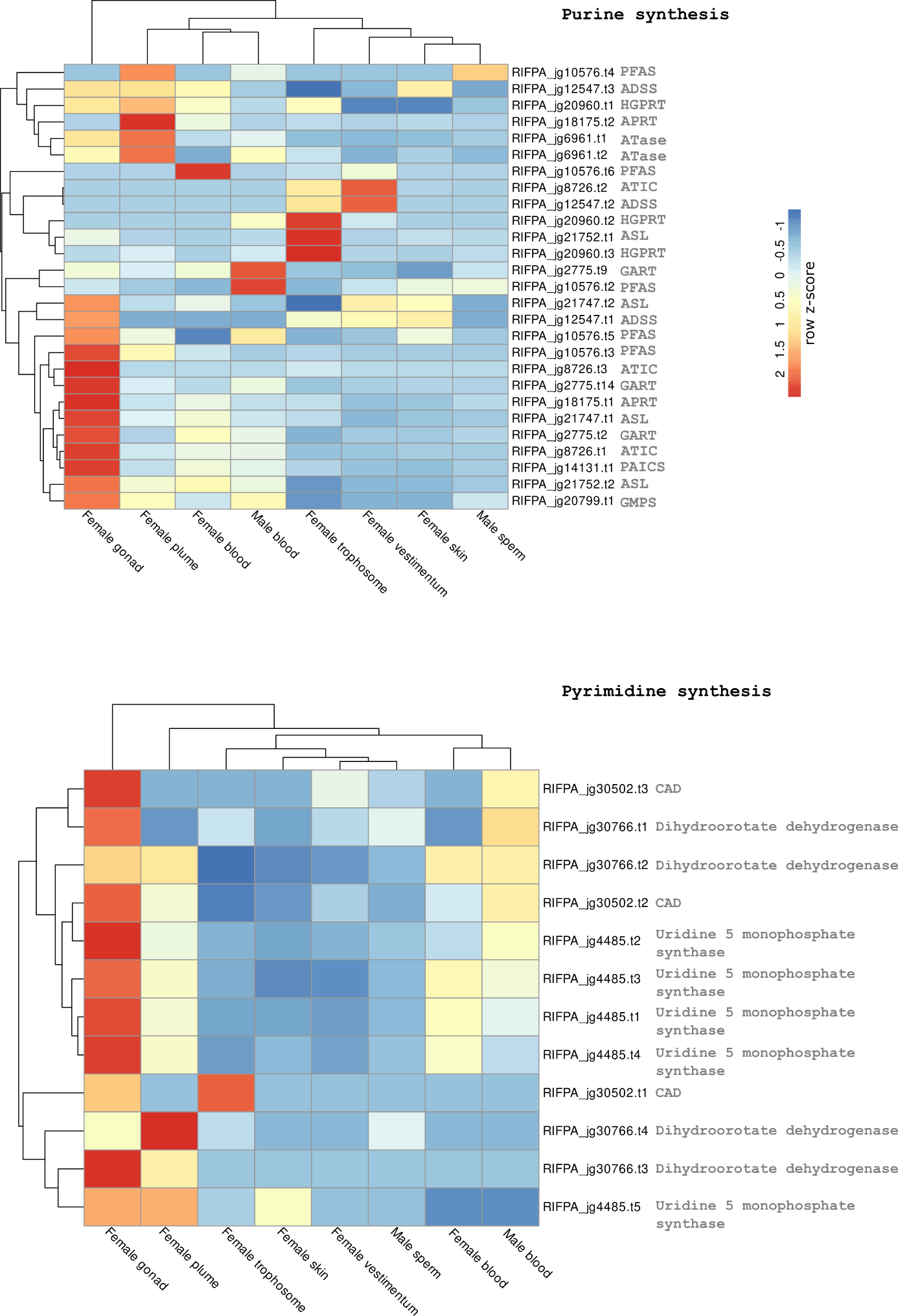
**

**Supplementary figure 42 | Gene expression of genes involved in the purine and pyrimidine pathways in *Riftia*.** Expression profile of different key enzymes related to the purine and pyrimidine biosynthesis pathways. Colour coding reflects the expression patterns based on row Z-score calculations. Abbreviations: PFAS: Phosphoribosylformylglycinamidine Synthase; ADSS: Adenylosuccinate Synthase; HGPRT: hypoxanthine phosphoribosyltransferase; APRT: adenine phosphoribosyltransferase; ATase: phosphoribosyl pyrophosphate amidotransferase; ATIC: 5-Aminoimidazole-4-Carboxamide Ribonucleotide Formyltransferase; ASL: Adenylosuccinate Lyase; ADSS: Adenylosuccinate Synthase; GART: Glycinamide Ribonucleotide Transformylase; PAICS: Phosphoribosylaminoimidazole Carboxylase And Phosphoribosylamino-imidazolesuccinocarboxamide Synthase; GMPS: Guanine Monophosphate Synthase; and CAD (Carbamoyl-Phosphate Synthetase 2, Aspartate Transcarbamylase, And Dihydroorotase)

**
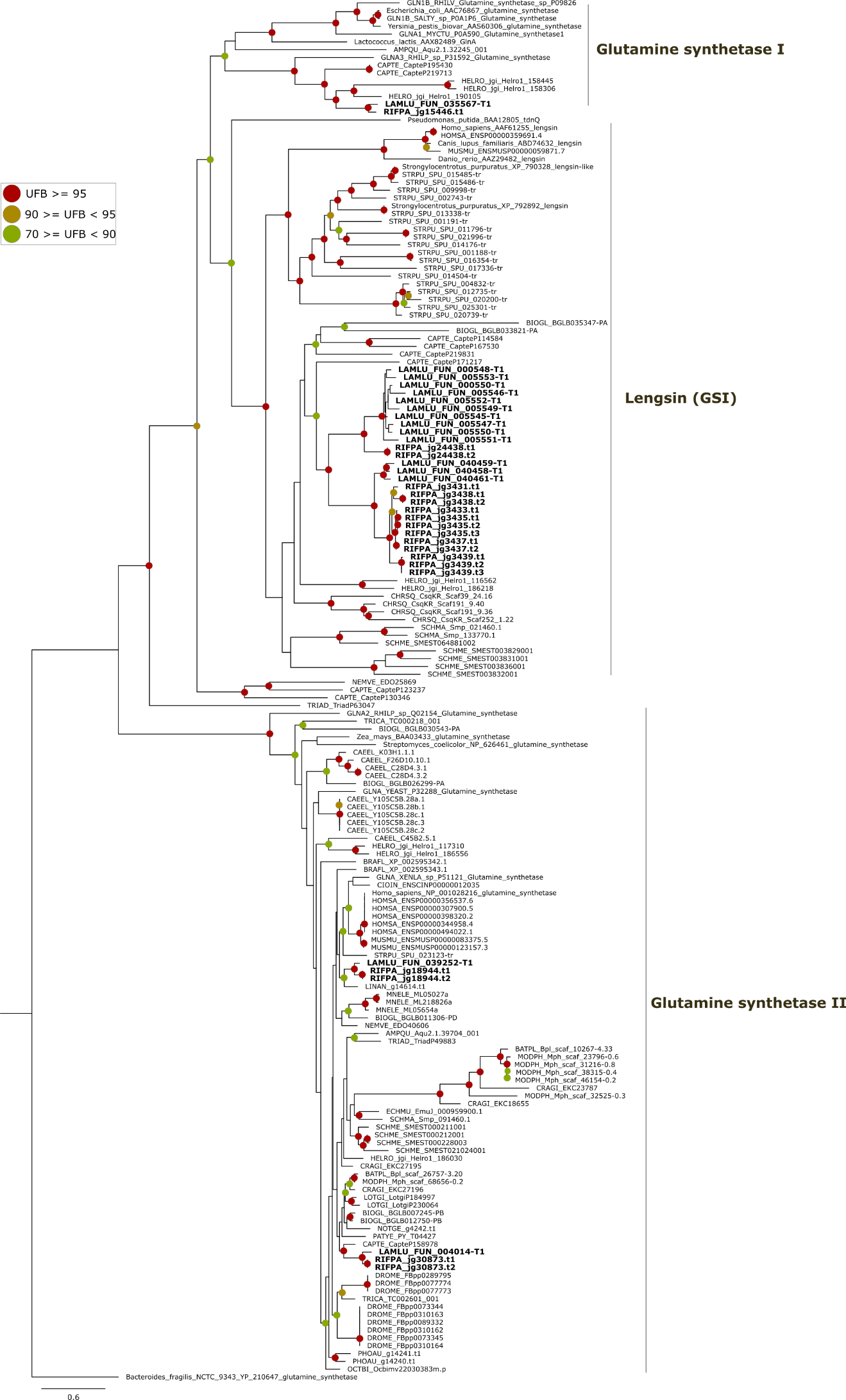
**

**Supplementary figure 43 | Phylogeny of glutamine synthetase genes in selected metazoans.** Maximum-likelihood phylogenetic tree inference of glutamine synthetase genes using 1000 ultrafast bootstrap replicates. The branch support values are represented by the coloured circles in the tree nodes. Red circles represent ultrafast bootstrap values >= 95. Yellow circles represent ultrafast bootstrap values >= 90 and < 95. Green circles represent ultrafast bootstrap values < 90 and >= 70. Ultrafast bootstrap values smaller than 70 are not shown. *Bacteroides fragilis* glutamine synthetase III gene was used as outgroup. Accession numbers for NCBI database are displayed after the species names. *Capitella, Helobdella* and *Lamellibrachia* gene identification are derived from the publicly available annotated genomes. Annelid sequences from *Riftia*, *Lamellibrachia, Capitella* and *Helobdella* are closely related to glutamine synthetase I bacterial genes.

**
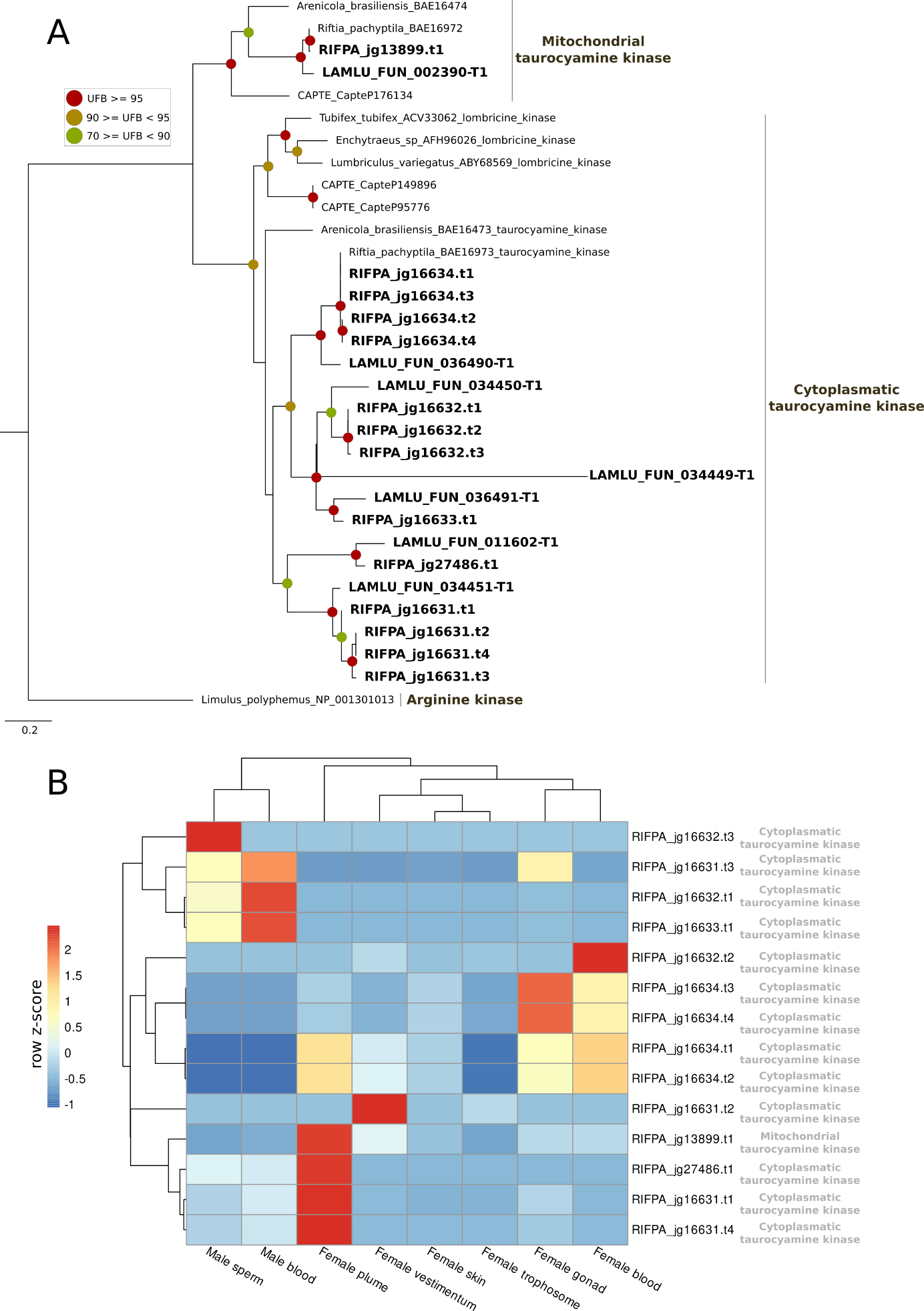
****Supplementary figure 44 | Phylogeny and gene expression of taurocyamine kinase genes**. **A.** Maximum-likelihood phylogenetic tree inference of the taurocyamine kinase genes using 1000 ultrafast bootstrap replicates. The branch support values are represented by the coloured circles in the tree nodes. Red circles represent ultrafast bootstrap values >= 95. Yellow circles represent ultrafast bootstrap values >= 90 and < 95. Green circles represent ultrafast bootstrap values < 90 and >= 70. Ultrafast bootstrap values smaller than 70 are not shown. *Limulus* arginine kinase gene was used as outgroup. Accession numbers for NCBI database are displayed after the species names. *Capitella, Helobdella* and *Lamellibrachia* gene identification are derived from the publicly available annotated genomes. *Riftia* contains five copies of cytoplasmatic taurocyamine kinase genes surpassing previous estimates. **B,** Expression profile of cytoplasmatic and mitochondrial taurocyamine kinase genes in *Riftia*. Colour coding reflects the expression patterns based on row Z-score calculations.

**
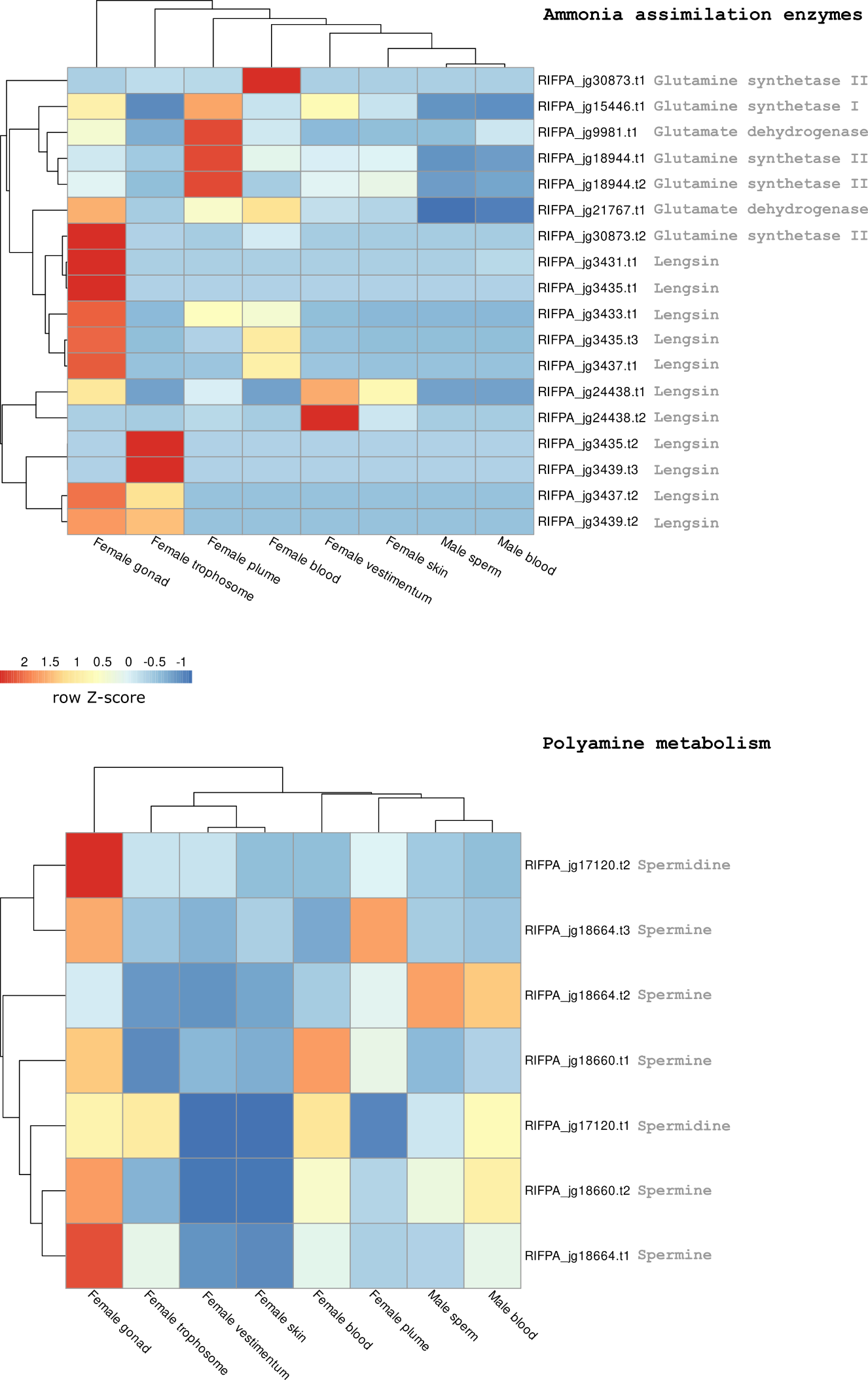
****Supplementary figure 45 | Gene expression of genes involved in the ammonia assimilation cycle and polyamine pathway in *Riftia*.** Expression profile of different key enzymes involved in the ammonia assimilation and polyamine pathway in *Riftia*. Colour coding reflects the expression patterns
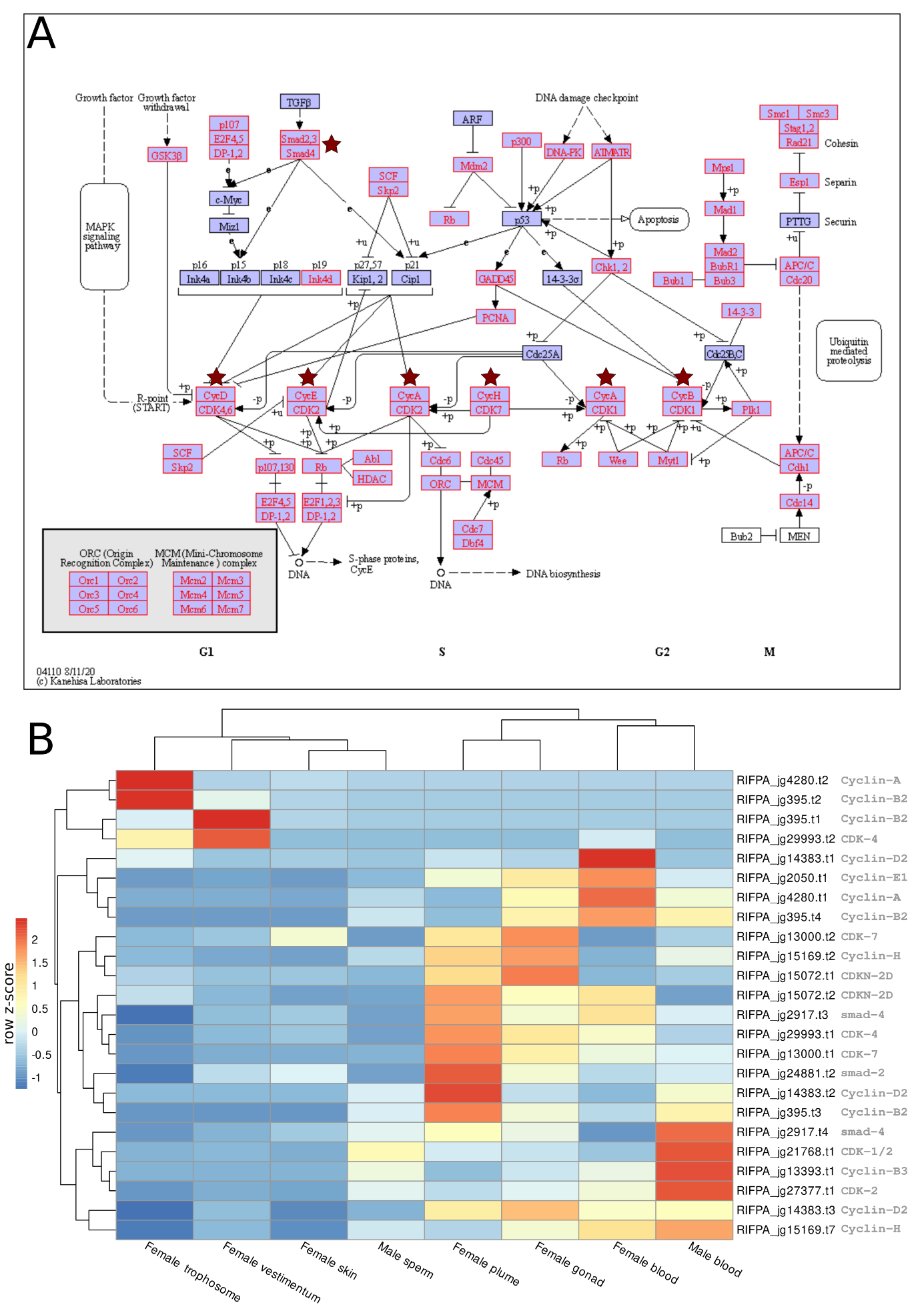
based on row Z-score calculations

**Supplementary figure 46 – Cell cycle pathway and gene expression of cyclins and cyclin-dependent kinase genes. A**, Cell cycle pathway reference based on KEGG hsa04110 entry. Highlighted boxes correspond to the tubeworm genes identified in the reference pathway. Red stars indicate the genes used in the gene expression analyses. **B,** Expression profile of cyclin and cyclin-dependent kinase genes. Colour coding reflects the expression patterns based on row Z-score calculations.


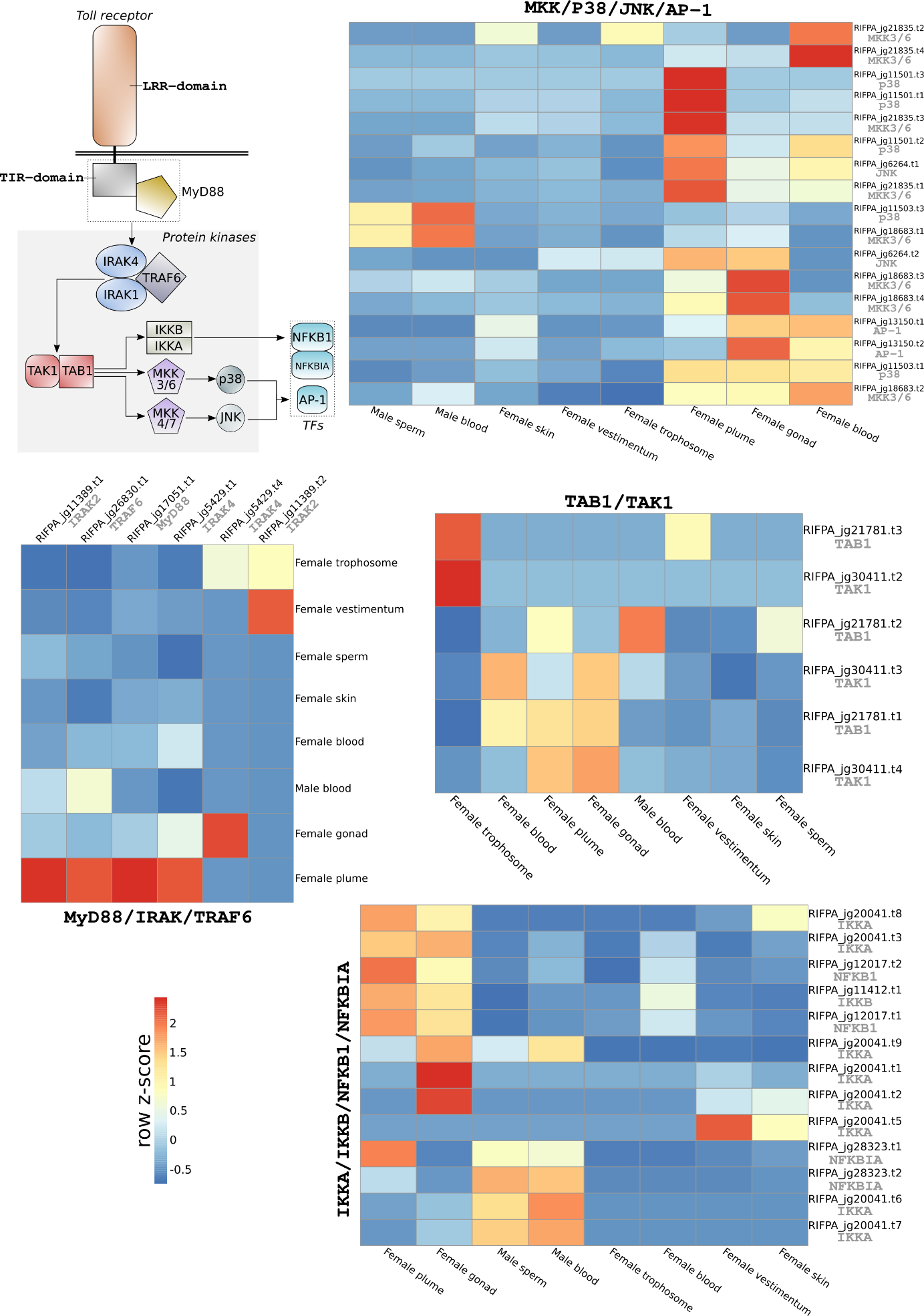


**Supplementary figure 47 | Overview of Toll-like receptor/MyD88 immune system pathway in *Riftia.*** Overview of the Toll-like receptor/MyD88 immune system pathway in *Riftia* (based on Luo et al., 2018). Expression profile of immune-related genes in the adult tissues of *Riftia pachyptila*. Colour coding in the different heatmaps reflects the expression patterns based on row Z-score calculations. It is possible to notice the high expression of immune-related genes in the plume and female gonad tissue.


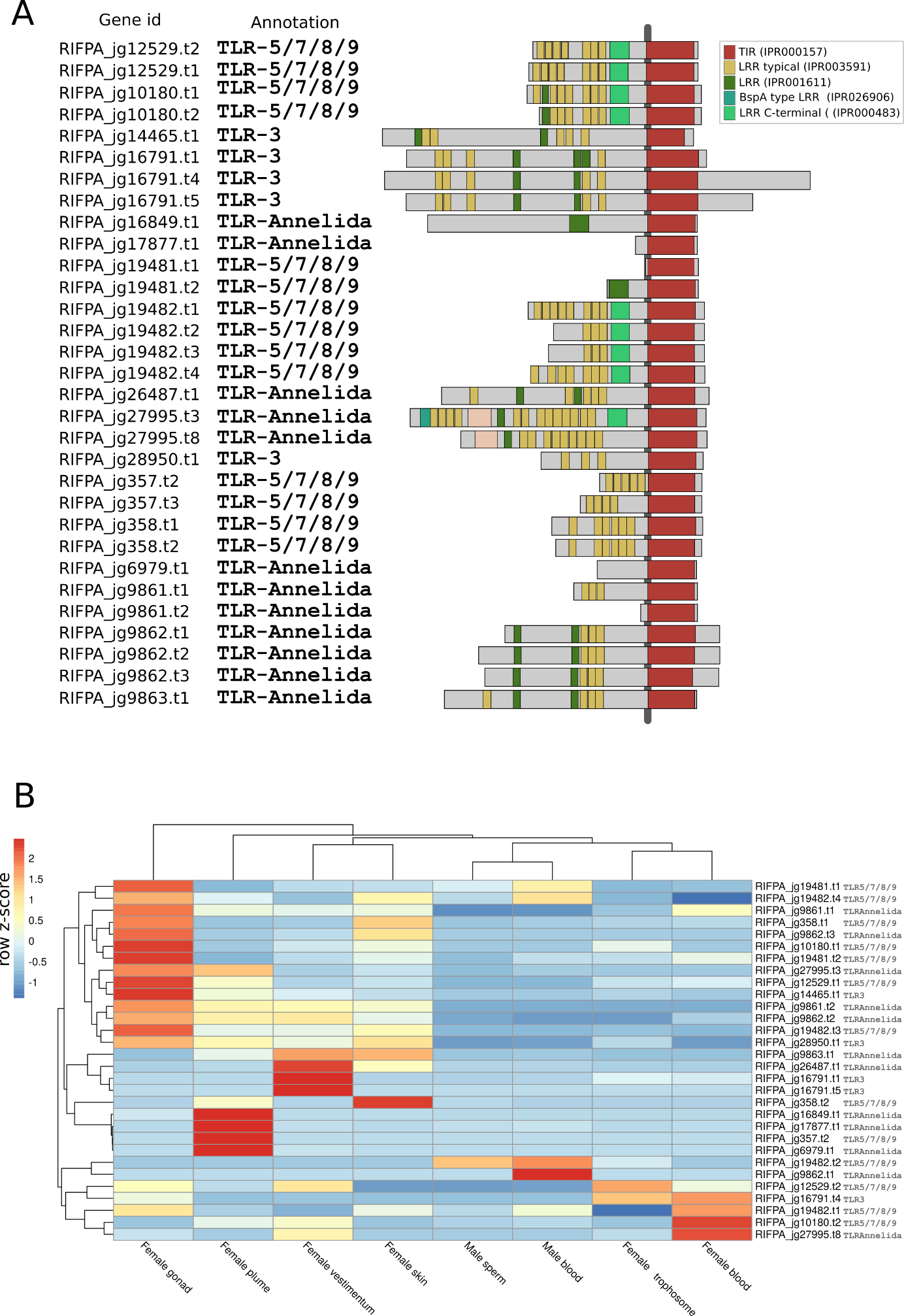


**Supplementary figure 48 | Toll-like gene domain composition and gene expression**. **A**, Domain composition of Toll-like genes based on PFAM database. Coloured boxes correspond to different protein domains found in the different Toll-like groups. **B,** Expression profile of Toll-like genes. Colour coding reflects the expression patterns based on row Z-score calculations. Toll-like genes are constitutively expressed in all *Riftia* adult tissues.

**
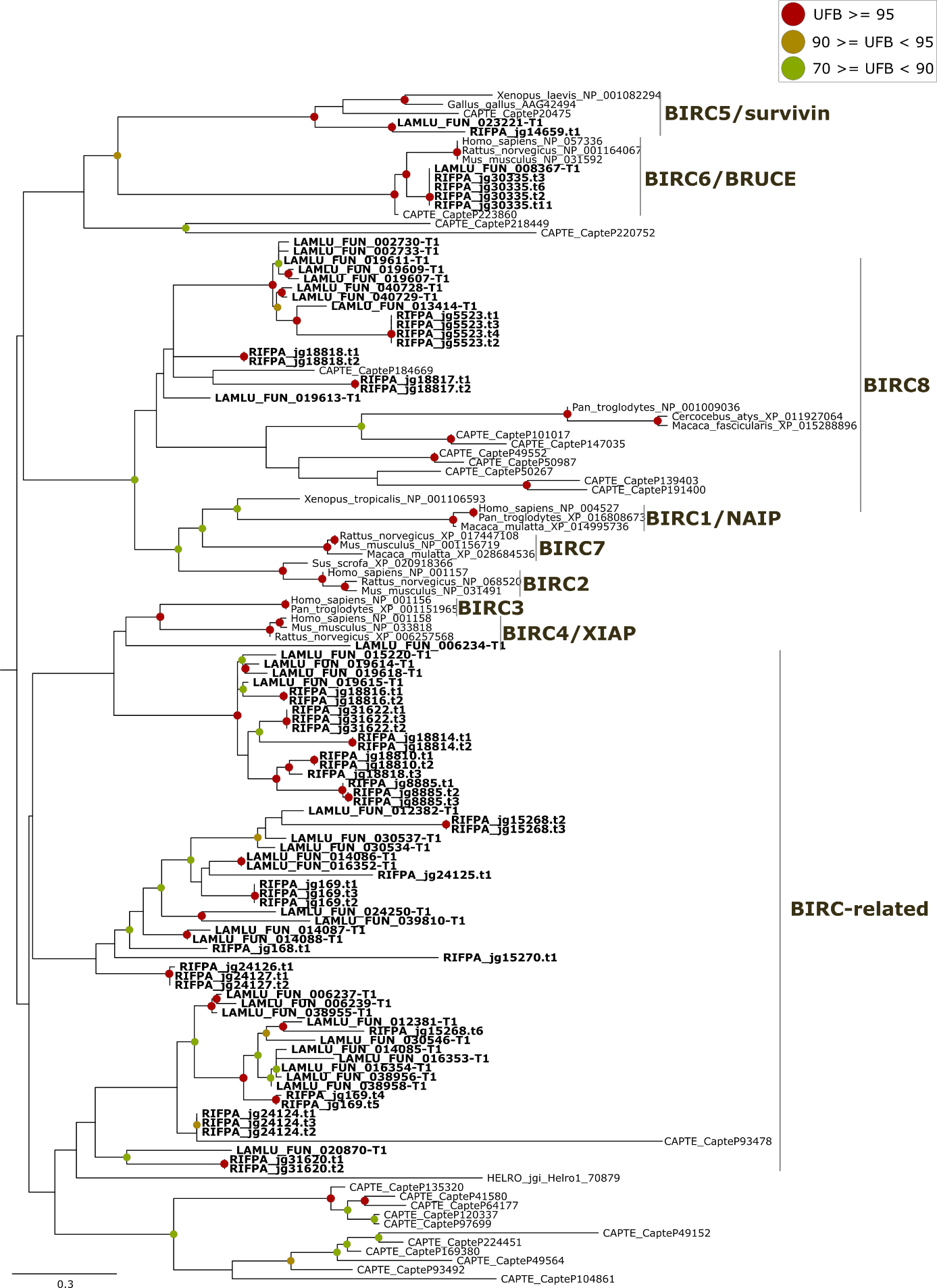
**

**Supplementary figure 49 - Phylogeny of Toll-like genes.** Maximum-likelihood phylogenetic tree inference of the Toll-like genes using 1000 ultrafast bootstrap replicates. The branch support values are represented by the coloured circles in the tree nodes. Red circles represent ultrafast bootstrap values >= 95. Yellow circles represent ultrafast bootstrap values >= 90 and < 95. Green circles represent ultrafast bootstrap values < 90 and >= 70. Ultrafast bootstrap values smaller than 70 are not shown. Vertebrate MyD88 genes were used as outgroup. Accession numbers for NCBI database are displayed after the species names. *Capitella, Helobdella* and *Lamellibrachia* gene identification are derived from the publicly available annotated genomes.


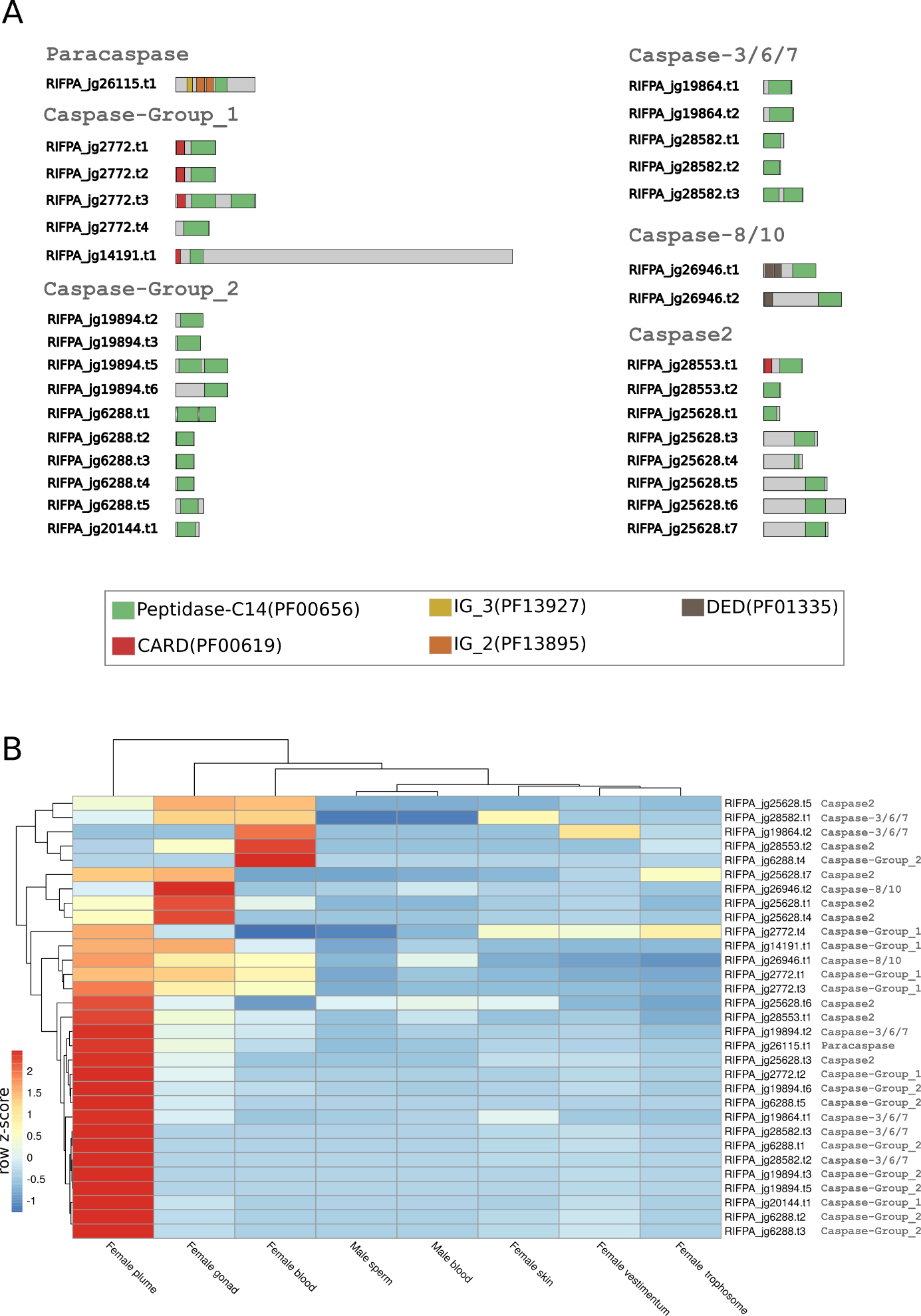
**Supplementary figure 50 | Caspase and paracaspase domain composition and gene expression. A,** Domain composition of caspases and paracaspases proteins based on PFAM database. Coloured boxes correspond to different protein domains found in the metacaspase and different caspase groups. **B,** Expression profile of caspase genes in the adult tissues of *Riftia pachyptila*. Colour coding reflects the expression patterns based on row Z-score calculations. Caspases are highly expressed in the plume and female gonad.

**
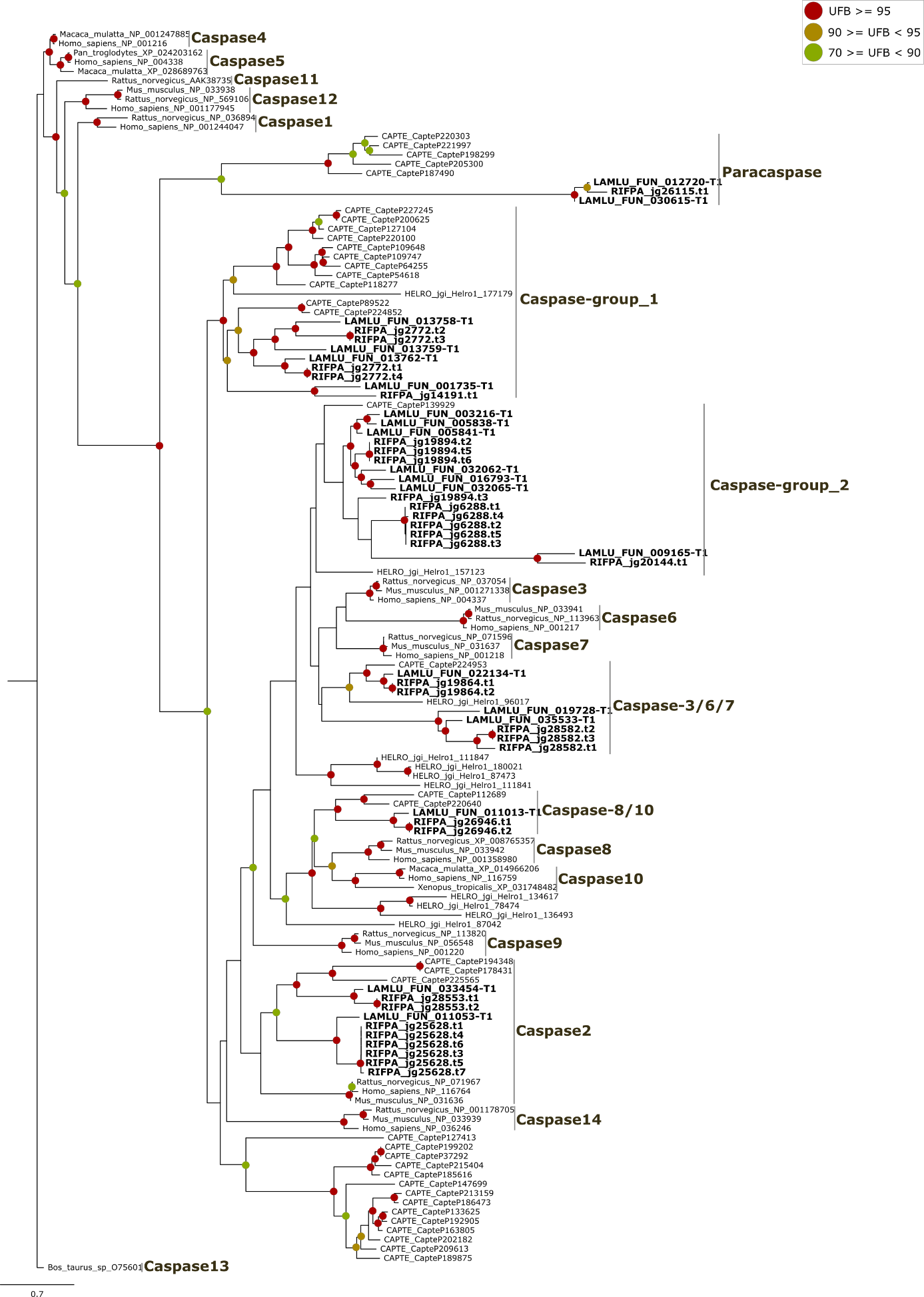
**

**Supplementary figure 51 | Phylogeny of caspases and paracaspases.** Maximum-likelihood phylogenetic tree inference of the caspases and paracaspases using 1000 ultrafast bootstrap replicates. The branch support values are represented by the coloured circles in the tree nodes. Red circles represent ultrafast bootstrap values >= 95. Yellow circles represent ultrafast bootstrap values >= 90 and < 95. Green circles represent ultrafast bootstrap values < 90 and >= 70. Ultrafast bootstrap values smaller than 70 are not shown. *Bos taurus* caspase-13 was used as outgroup. Accession numbers for NCBI database are displayed after the species names. *Capitella, Helobdella* and *Lamellibrachia* gene identification are derived from the publicly available annotated genomes.


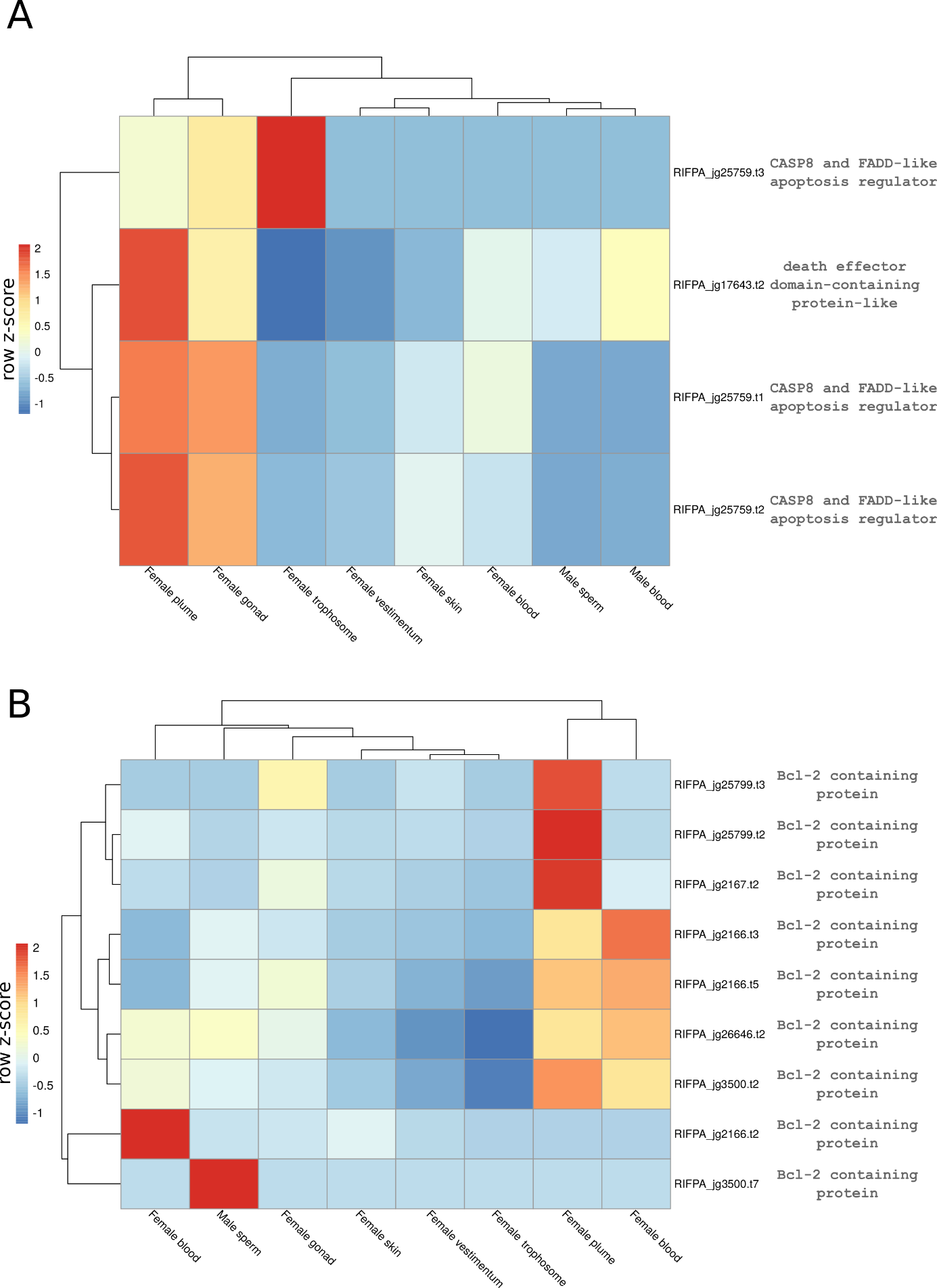


**Supplementary figure 52 | Gene expression of DED and BCL2 domain -containing proteins. A-B**, Expression profile of DED and BCL-2 domain-containing proteins obtained from the PFAM analysis. Colour coding reflects the expression patterns based on row Z-score calculations. DED- and BCL-2-domain containing proteins are highly expressed in the plume and female blood.

**
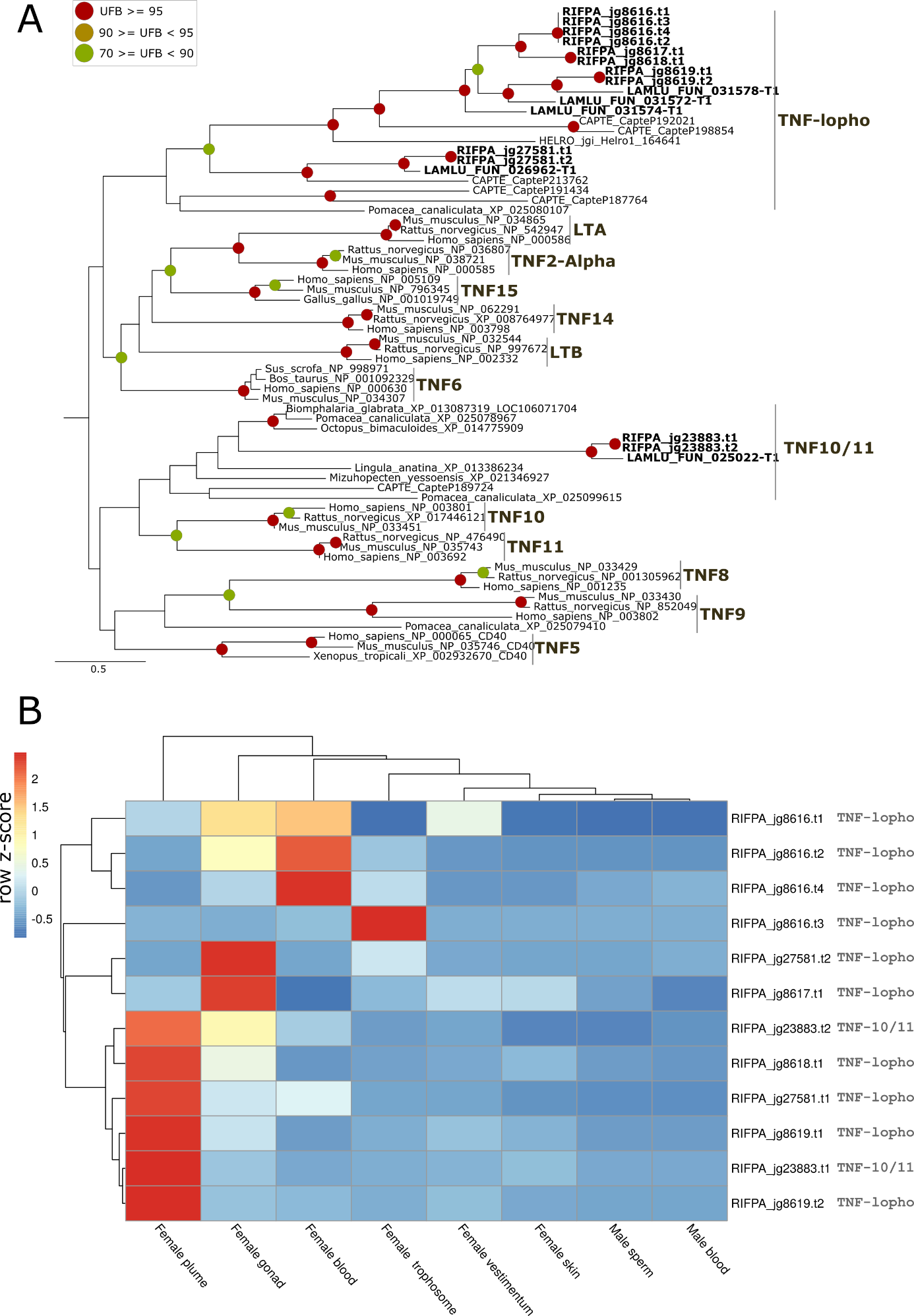
**

**Supplementary figure 53 | Phylogeny and gene expression of TNF ligands. A,** Mid-rooted maximum-likelihood phylogenetic tree inference of the TNF ligands using 1000 ultrafast bootstrap replicates. The branch support values are represented by the coloured circles in the tree nodes. Red circles represent ultrafast bootstrap values >= 95. Yellow circles represent ultrafast bootstrap values >= 90 and < 95. Green circles represent ultrafast bootstrap values < 90 and >= 70. Ultrafast bootstrap values smaller than 70 are not shown. Accession numbers for NCBI database are displayed after the species names. *Capitella, Helobdella* and *Lamellibrachia* gene identification are derived from the publicly available annotated genomes. **B,** Expression profile of TNF ligands. Colour coding reflects the expression patterns based on row Z-score calculations.


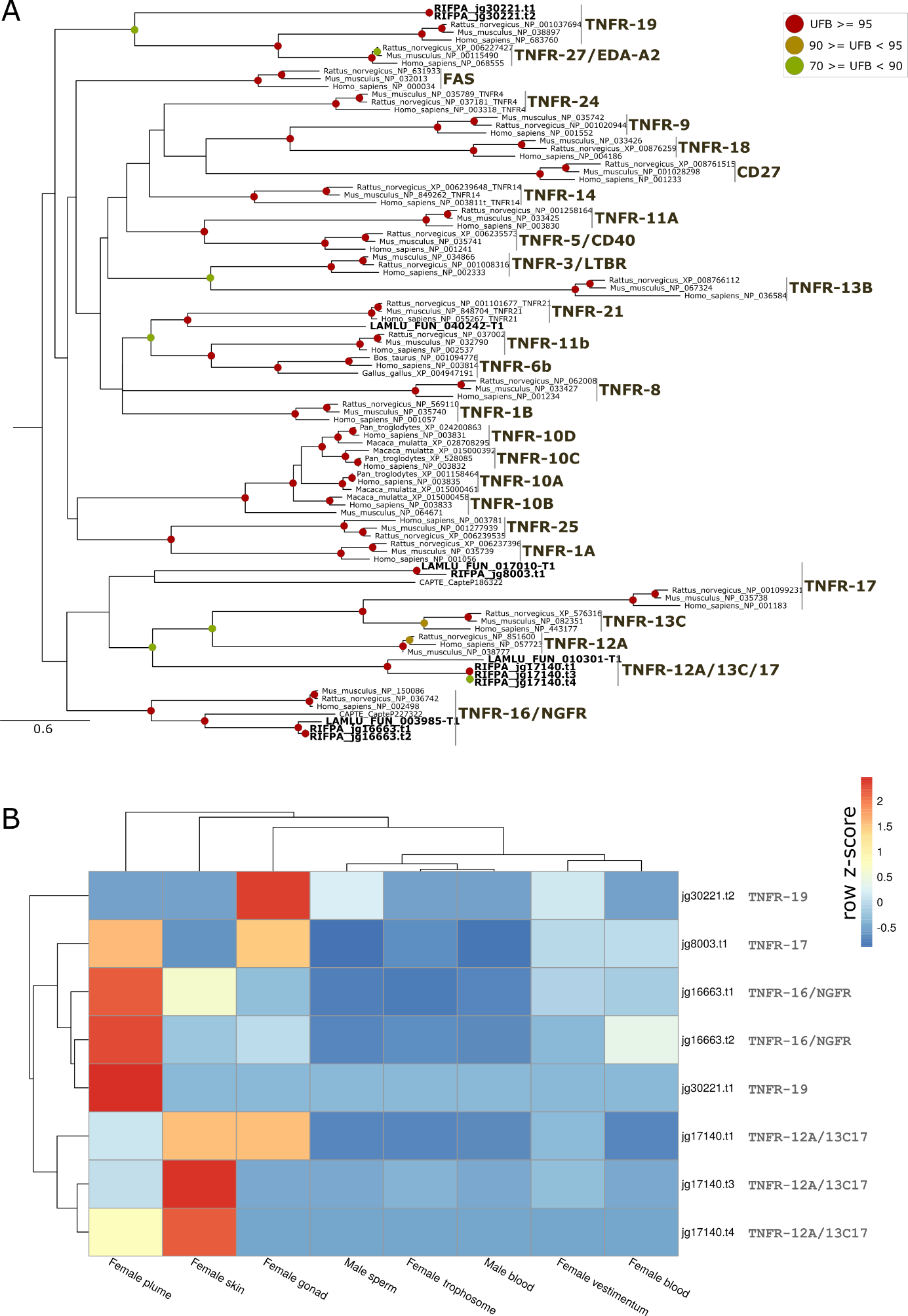


**Supplementary figure 54 | Phylogeny and gene expression of TNF receptors. A,** Mid-rooted maximum-likelihood phylogenetic tree inference of the TNF receptors using 1000 ultrafast bootstrap replicates. The branch support values are represented by the coloured circles in the tree nodes. Red circles represent ultrafast bootstrap values >= 95. Yellow circles represent ultrafast bootstrap values >= 90 and < 95. Green circles represent ultrafast bootstrap values < 90 and >= 70. Ultrafast bootstrap values smaller than 70 are not shown. Accession numbers for NCBI database are displayed after the species names. *Capitella, Helobdella* and *Lamellibrachia* gene identification are derived from the publicly available annotated genomes. **B,** Expression profile of TNF receptors. colour coding reflects the expression patterns based on row Z-score calculations.

**
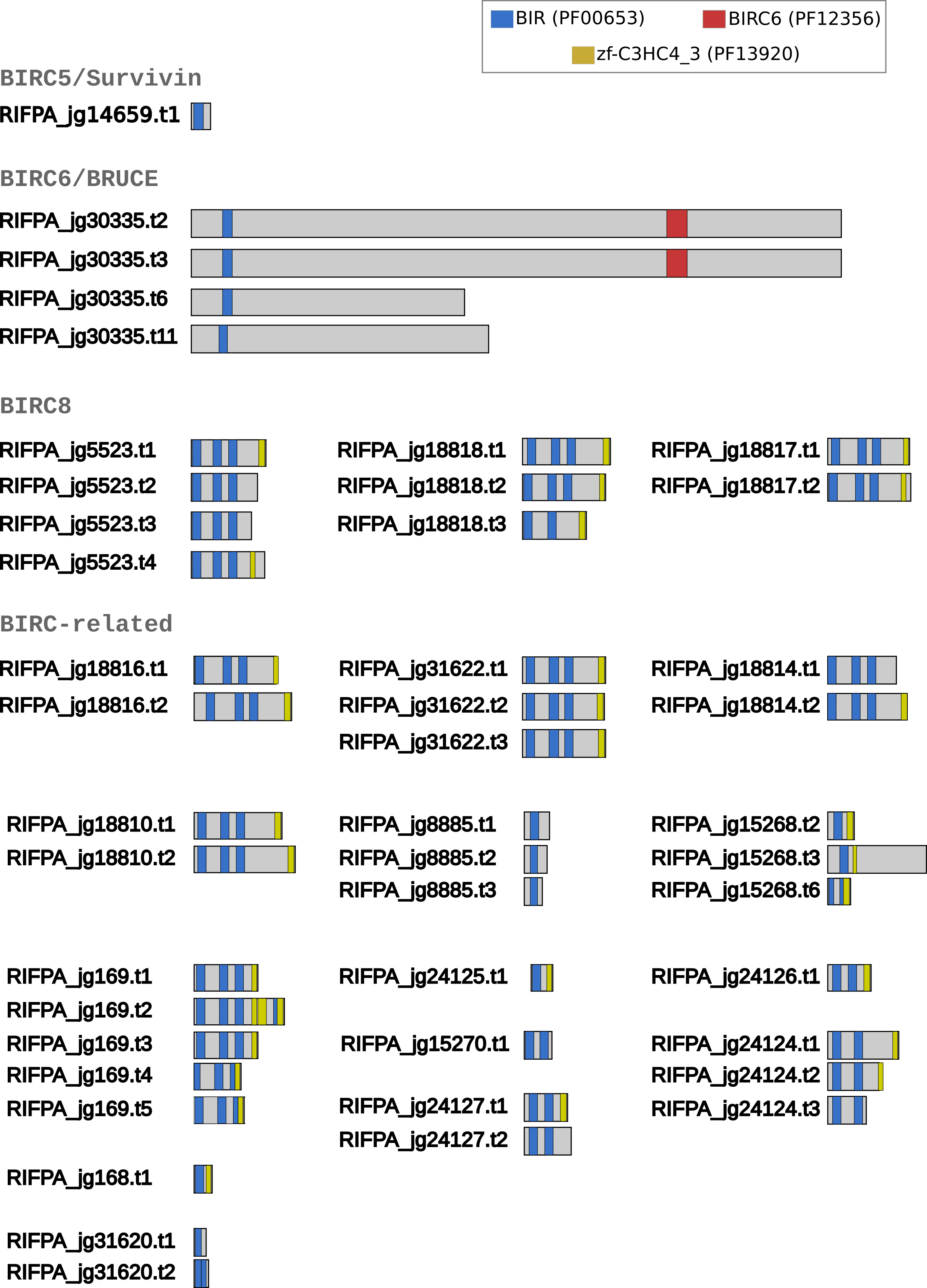
**

**Supplementary figure 55 | Domain composition of IAP genes**. Domain composition of IAP genes based on PFAM database. Coloured boxes correspond to different protein domains found in the different IAP groups. We identified a complement of 19 IAP genes on *Riftia* genome.


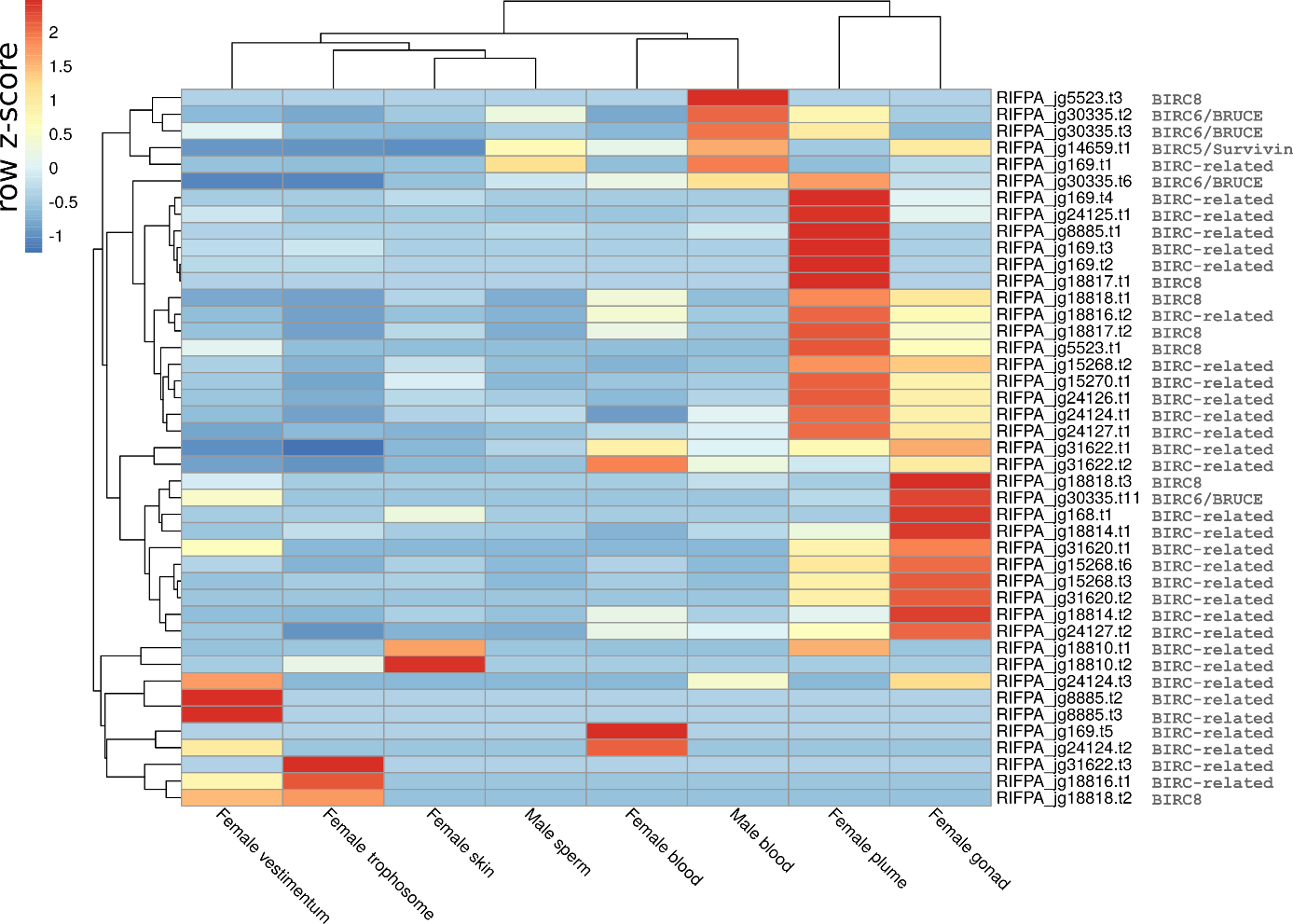


**Supplementary figure 56 | Gene expression of IAP domain-containing proteins.** Expression profile IAP domain-containing proteins obtained from the PFAM analysis. Colour coding reflects the expression patterns based on row Z-score calculations. IAP gene expression is present in all tissues, however, many IAP genes are highly expressed on the female gonad and plume tissues.

**
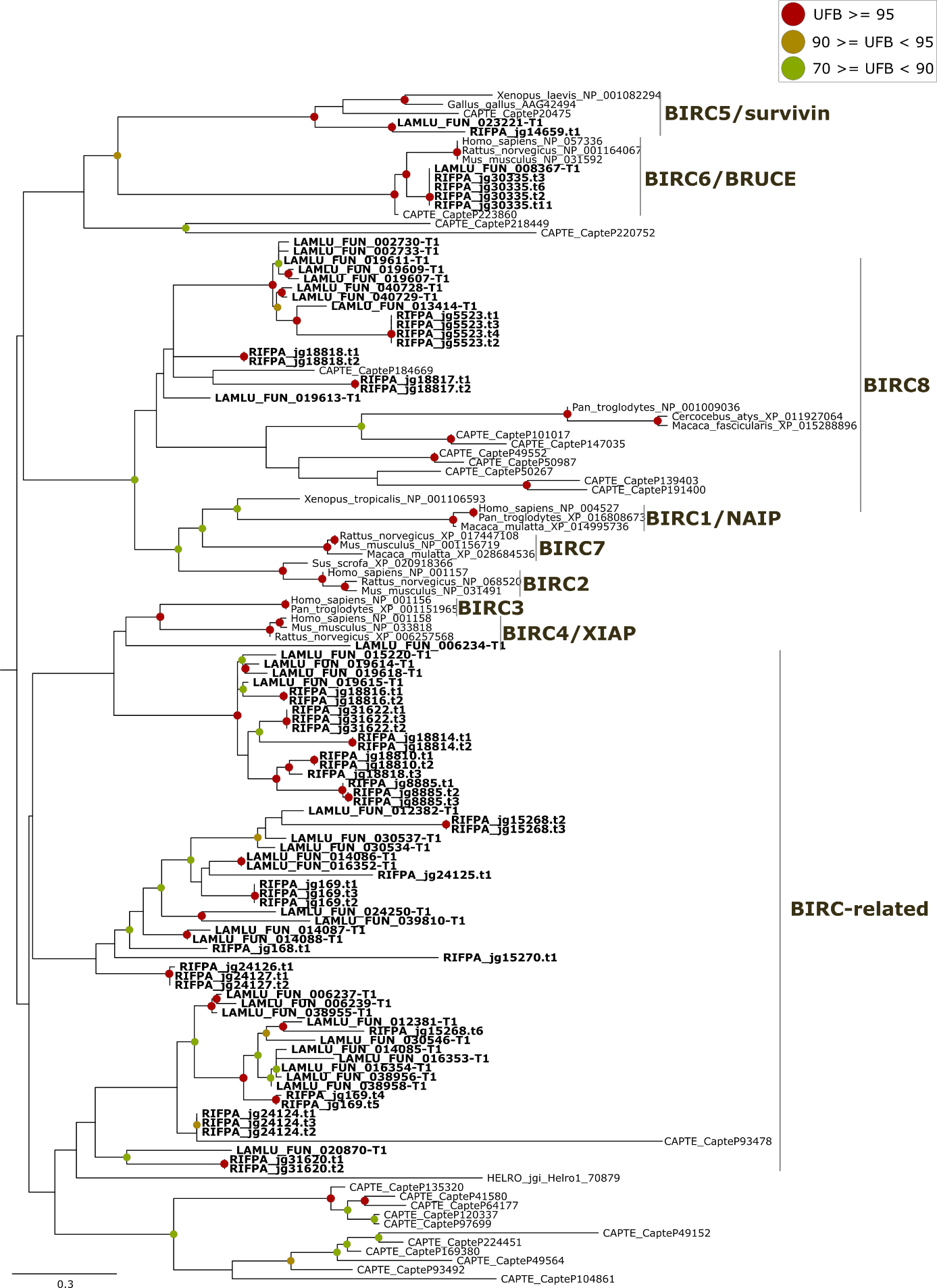
**

**Supplementary figure 57 | Phylogeny of IAP genes.** Mid-rooted maximum-likelihood phylogenetic tree inference of the IAP genes using 1000 ultrafast bootstrap replicates. The branch support values are represented by the coloured circles in the tree nodes. Red circles represent ultrafast bootstrap values >= 95. Yellow circles represent ultrafast bootstrap values >= 90 and < 95. Green circles represent ultrafast bootstrap values < 90 and >= 70. Ultrafast bootstrap values smaller than 70 are not shown. Accession numbers for NCBI database are displayed after the species names. *Capitella, Helobdella* and *Lamellibrachia* gene identification are derived from the publicly available annotated genomes.


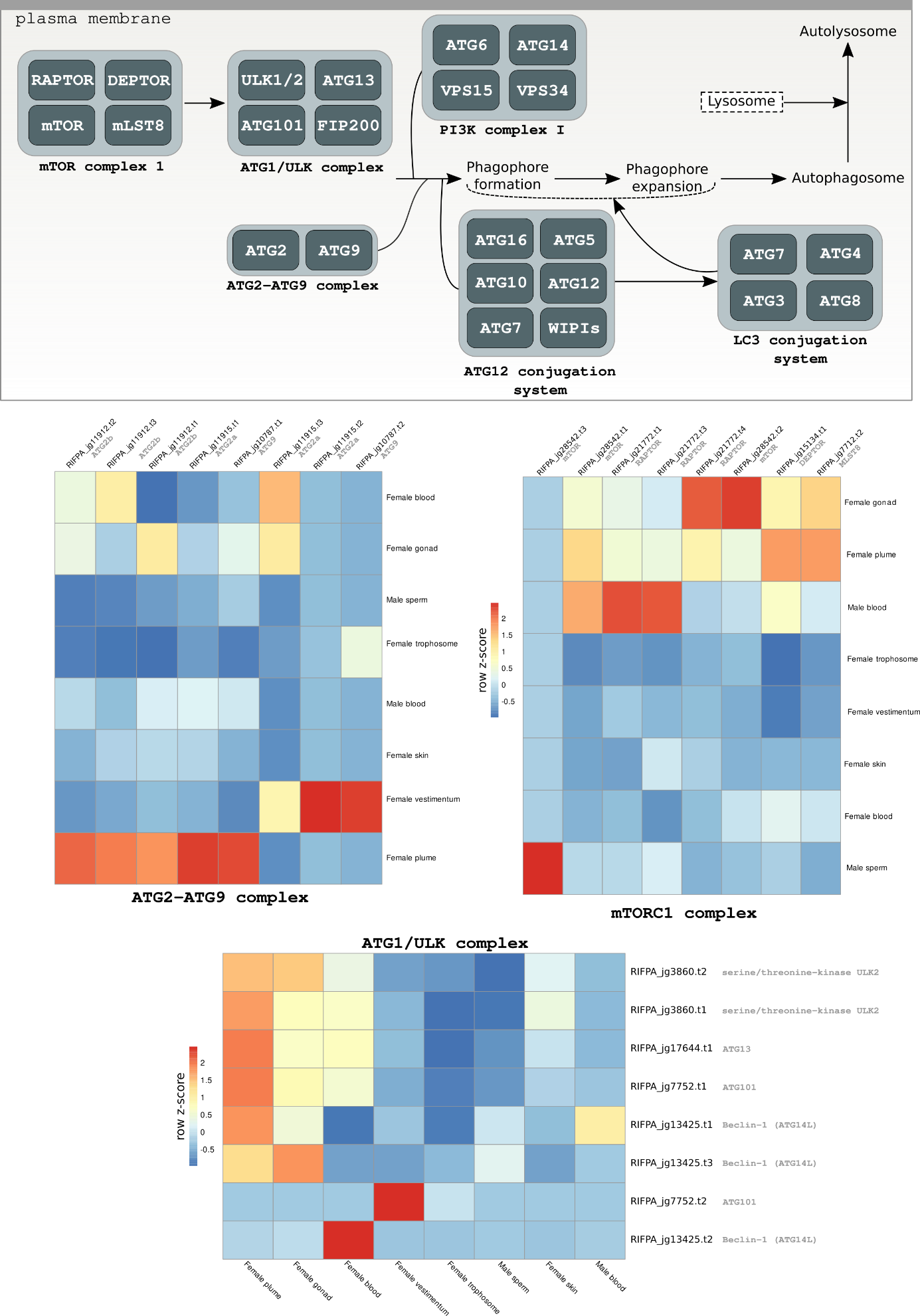


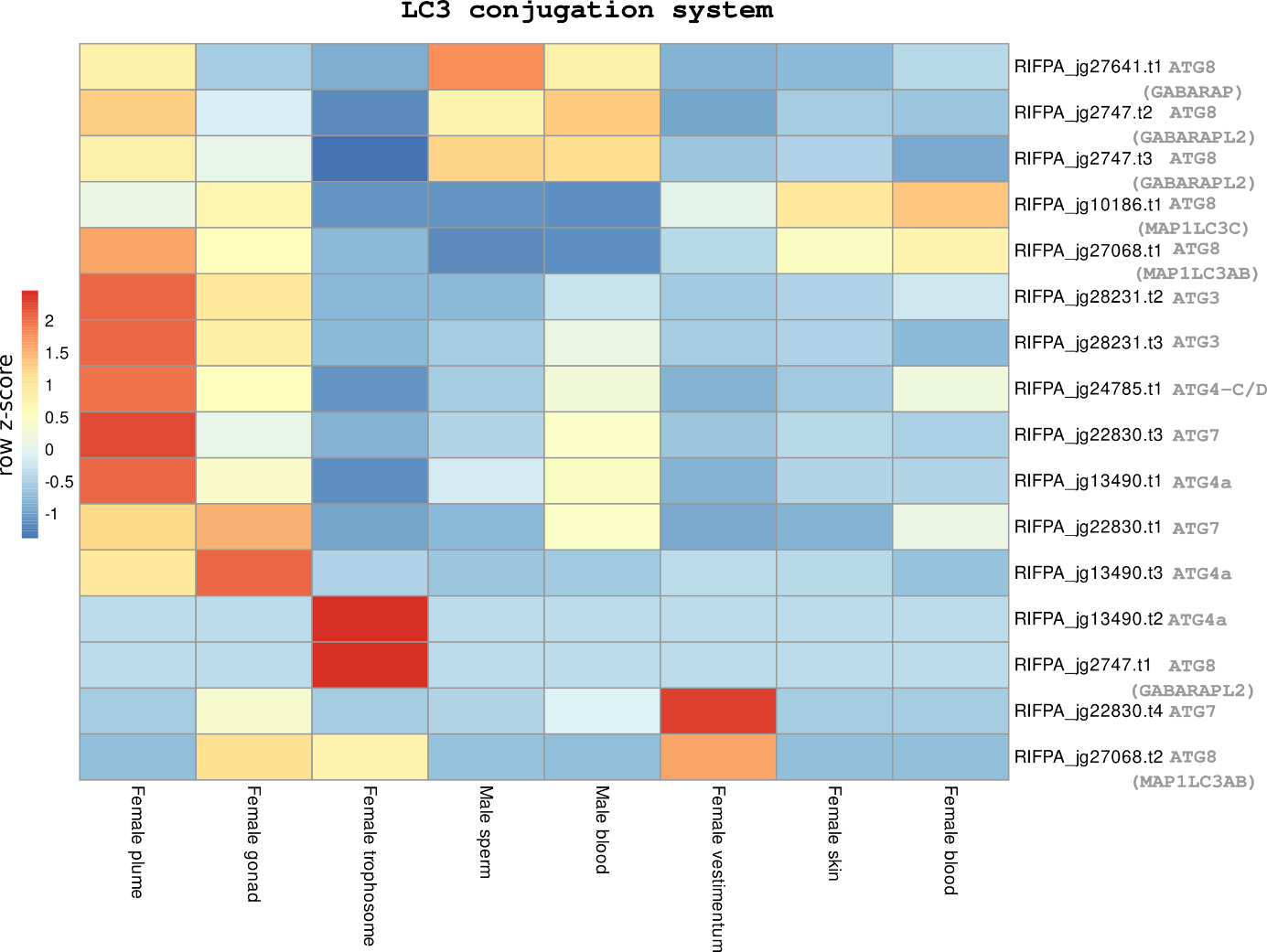

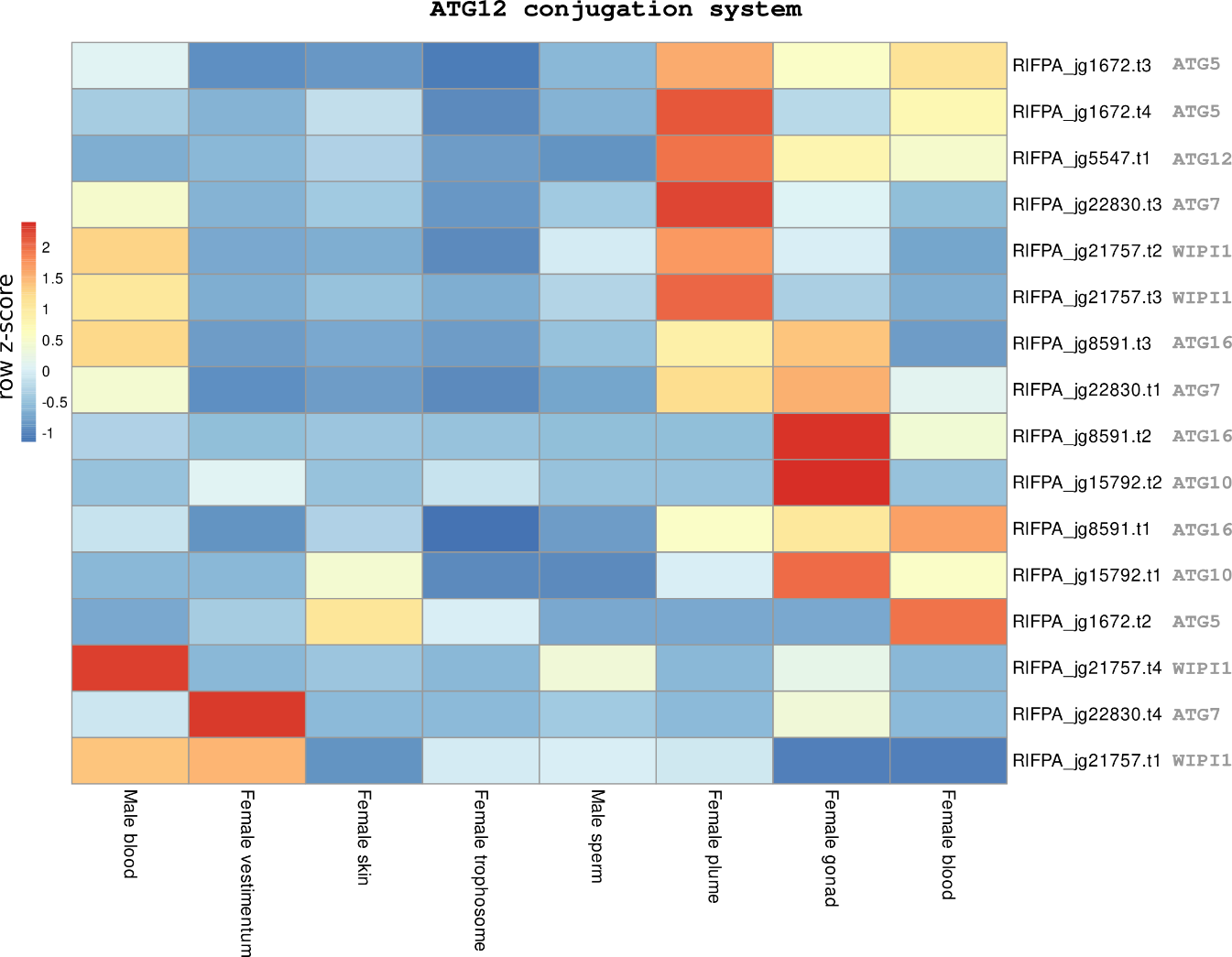


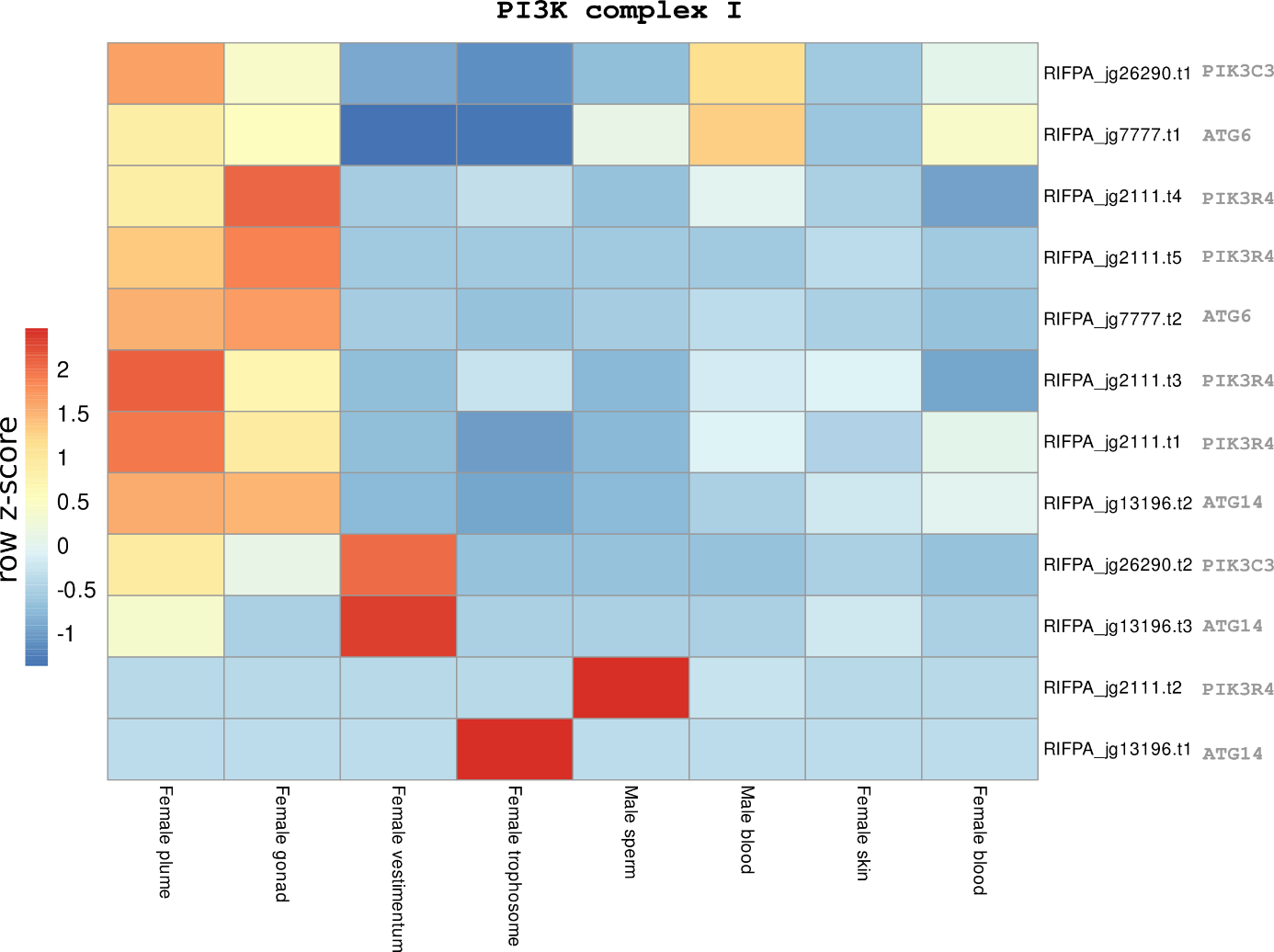


**Supplementary figure 58 | Overview of autophagy pathway in *Riftia* and gene expression of autophagy-related genes.** Overview of the core elements present in the autophagy pathway in *Riftia.* The giant tubeworm contains all the core elements commonly found in yeast, deuterostomes and lophotrochozoans. Expression profile of autophagy-related genes in the adult tissues of *Riftia pachyptila*. Colour coding in the different heatmaps reflects the expression patterns based on row Z-score calculations. It is possible to notice the high expression of autophagy-related genes in the plume and female gonad tissue.


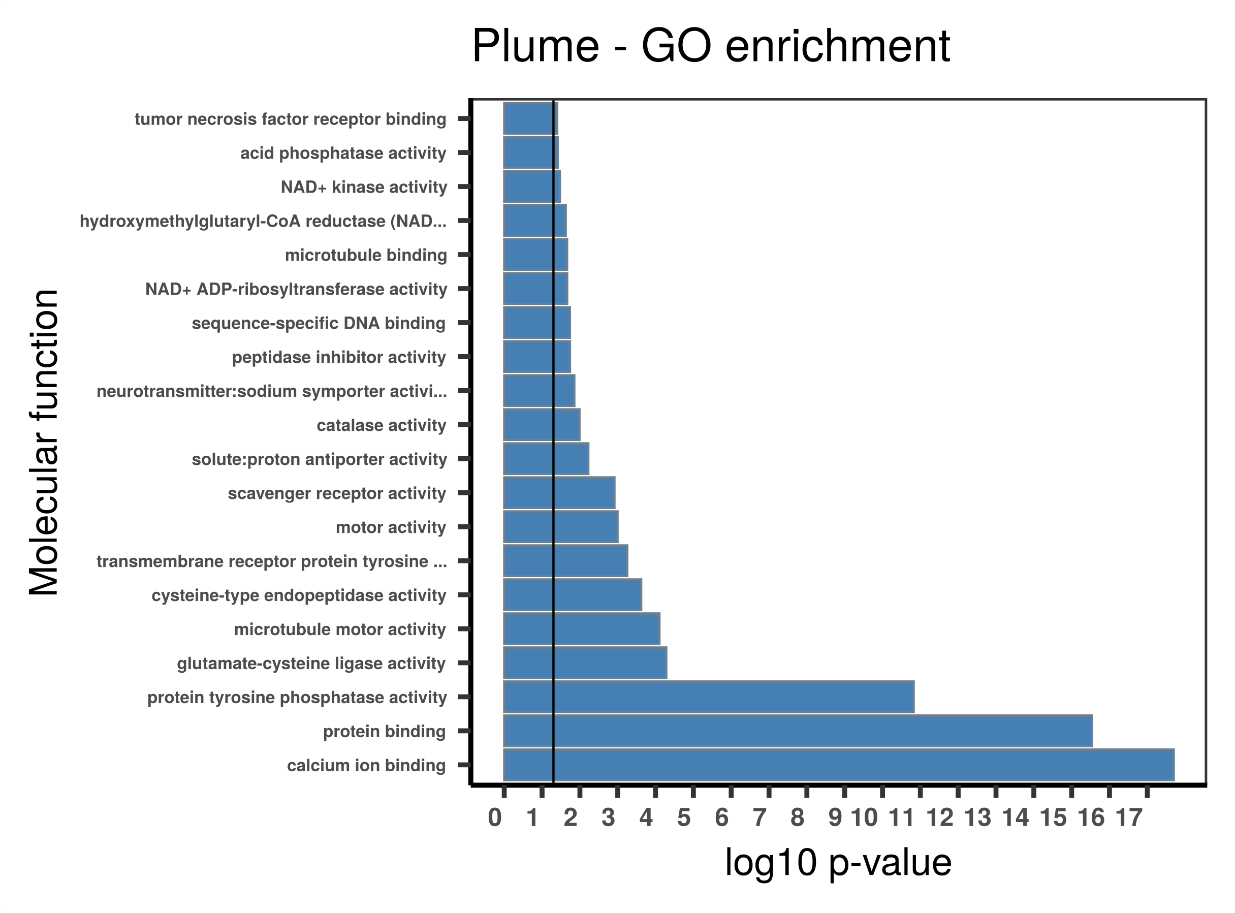

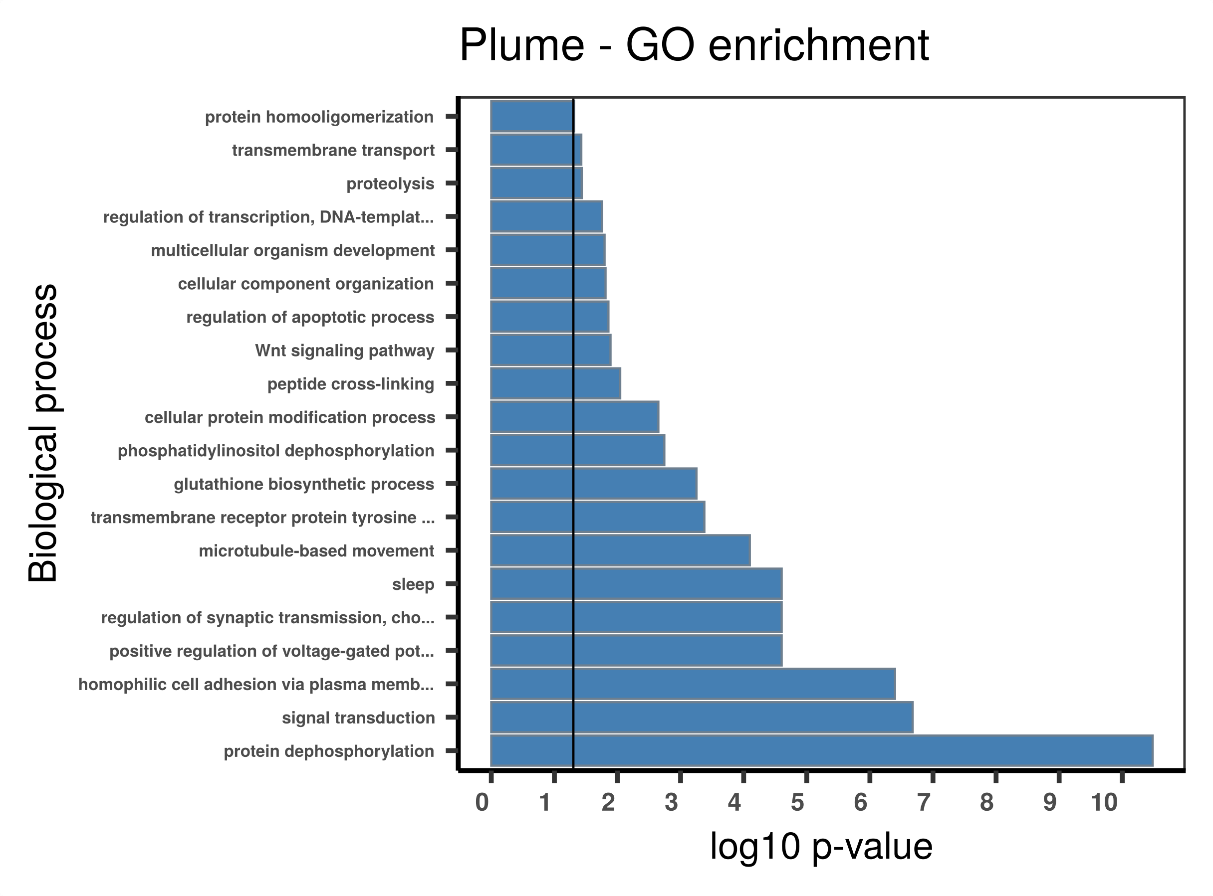


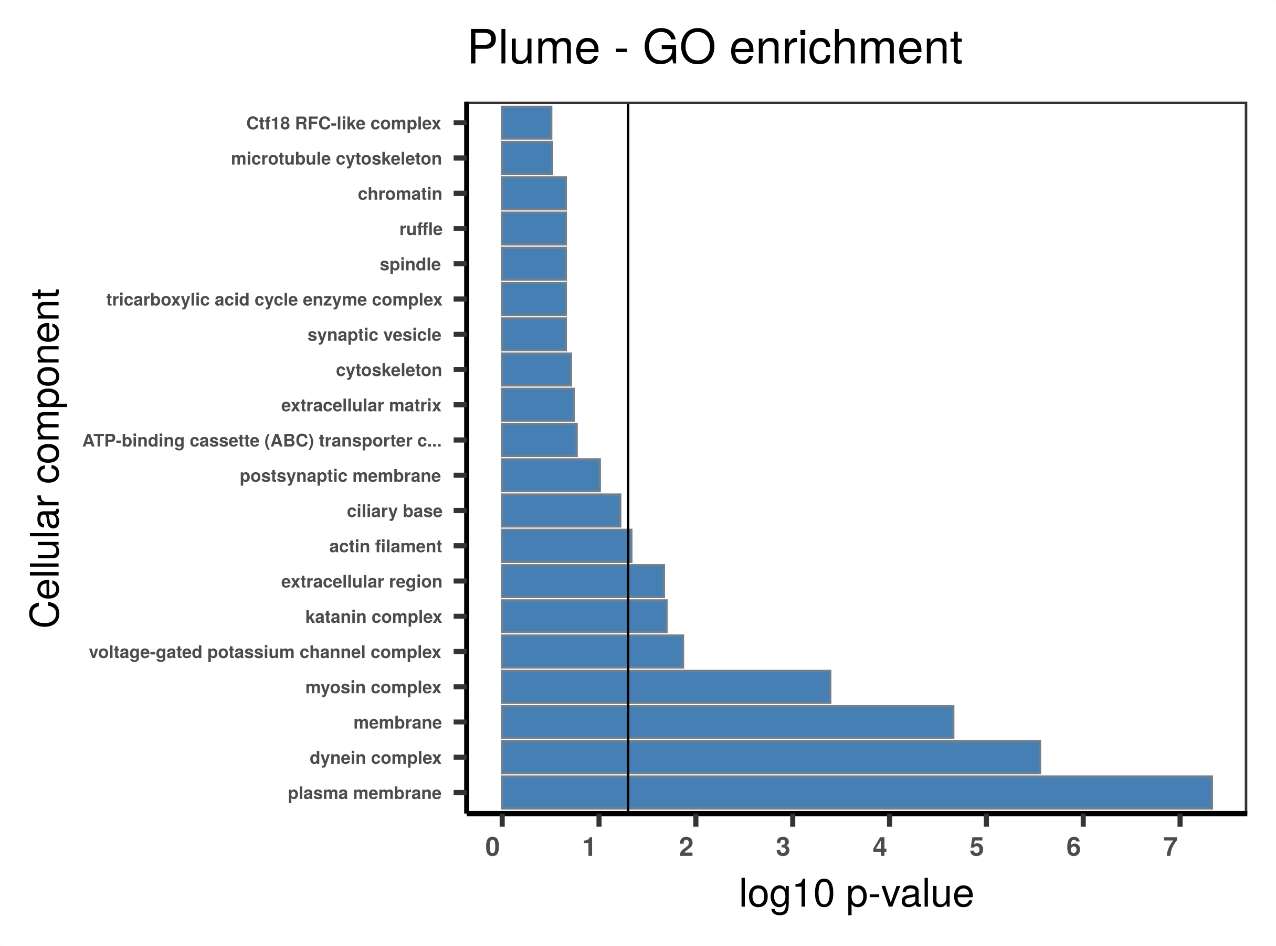


**Supplementary figure 59 | Gene set enrichment analysis with topGO using absolutely plume specific TAU genes**. Gene ontology (GO) enrichment analyses for absolutely plume specific TAU genes. The graphs correspond to the three domains of ontologies: biological process (BP), molecular function (MF) and cellular component (CC). The selected genes were analysed for enrichment in specific GO categories using the TopGO program against the background (all coding sequence genes). Y axis corresponds to enriched GO terms found in the respective domains (BP, MF and CC). X axis correspond to the log function of Fisher p-values obtained for each one of the enriched terms. The back line denotes a p-value = 0.05. P-values greater than 1,30 (log 0,05) indicate statistically significant enriched term. Genes involved in apoptosis, cytoskeleton, signal transduction and cell division are differentially expressed in the plume.


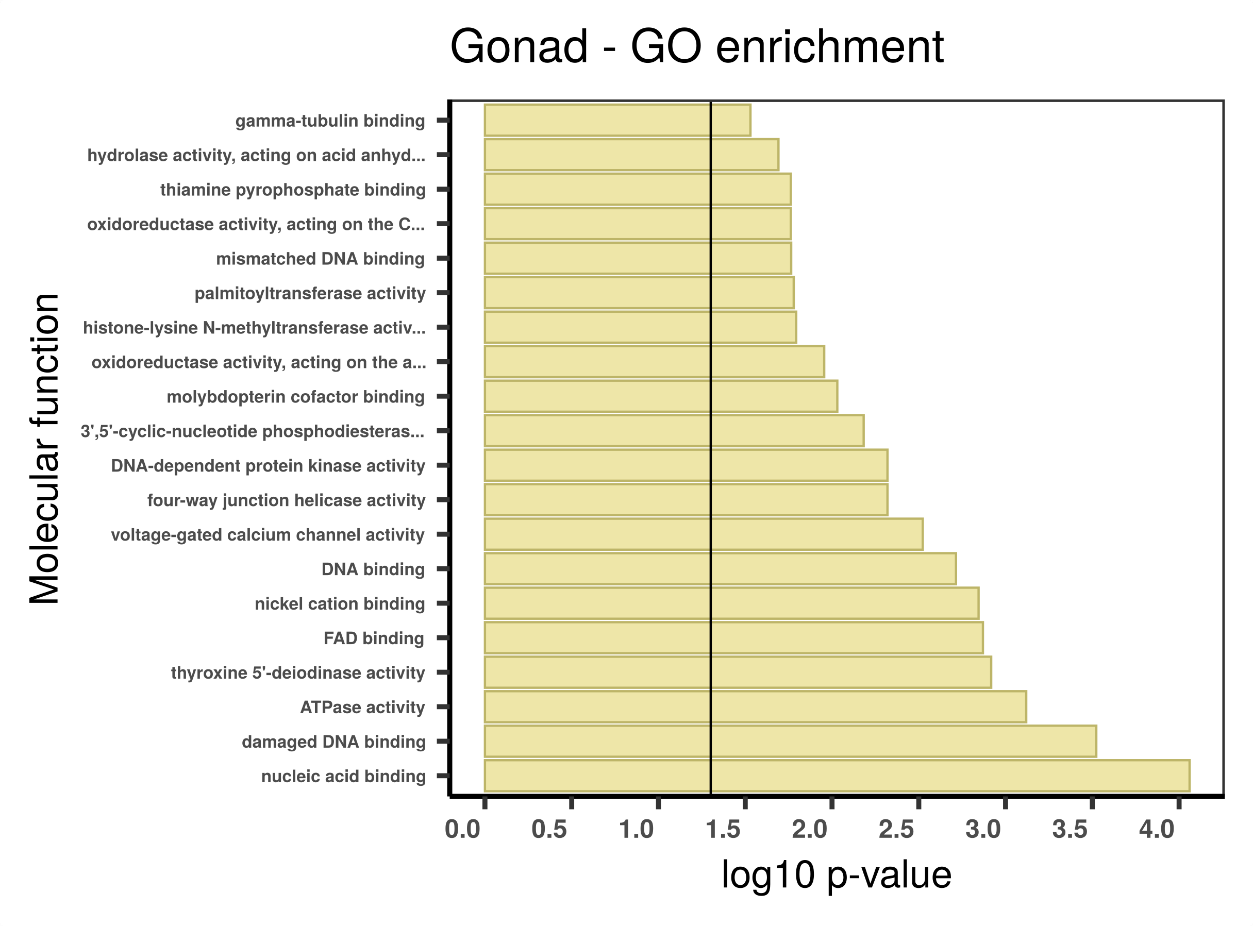

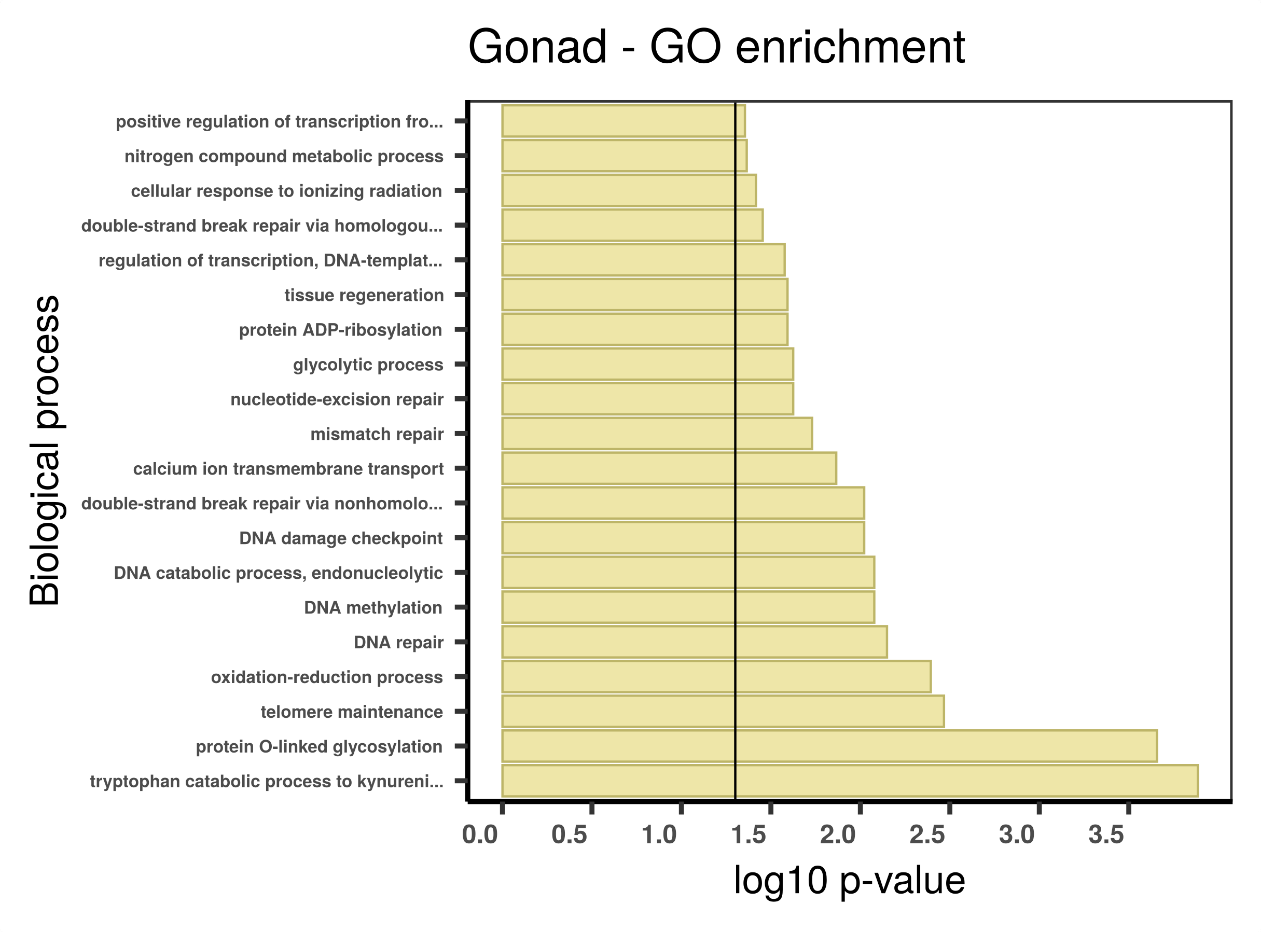


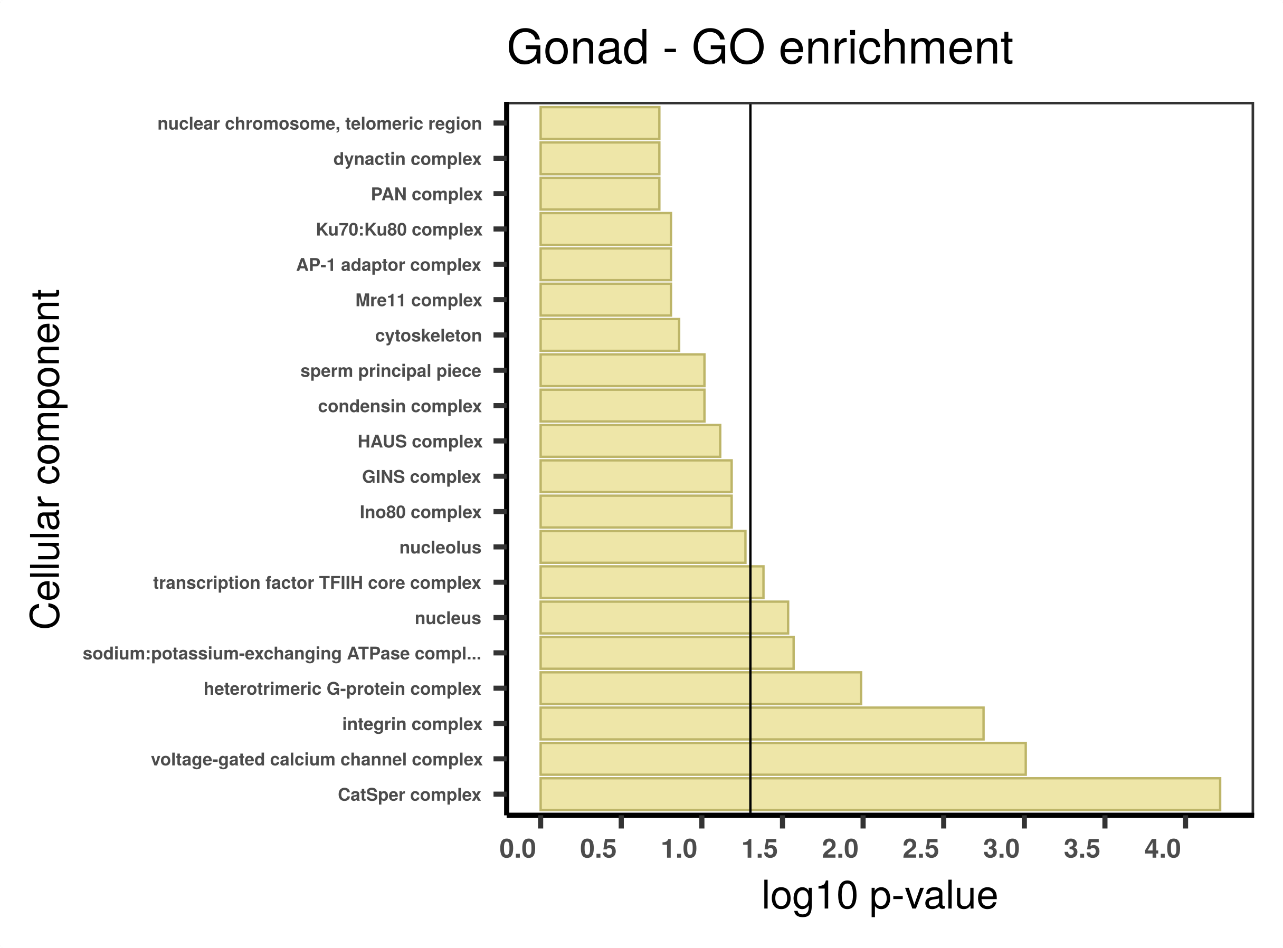


**Supplementary figure 60 | Gene set enrichment analysis with topGO using absolutely gonad specific TAU genes** Gene ontology (GO) enrichment analyses for absolutely female gonad specific TAU genes. The graphs correspond to the three domains of ontologies: biological process (BP), molecular function (MF) and cellular component (CC). The selected genes were analysed for enrichment in specific GO categories using the TopGO program against the background (all coding sequence genes). Y axis corresponds to enriched GO terms found in the respective domains (BP, MF and CC). X axis correspond to the log function of Fisher p-values obtained for each one of the enriched terms. The back line denotes a p-value = 0.05. P-values greater than 1,30 (log 0,05) indicate statistically significant enriched term. Genes involved in genome integrity are differentially expressed in female gonad tissue.


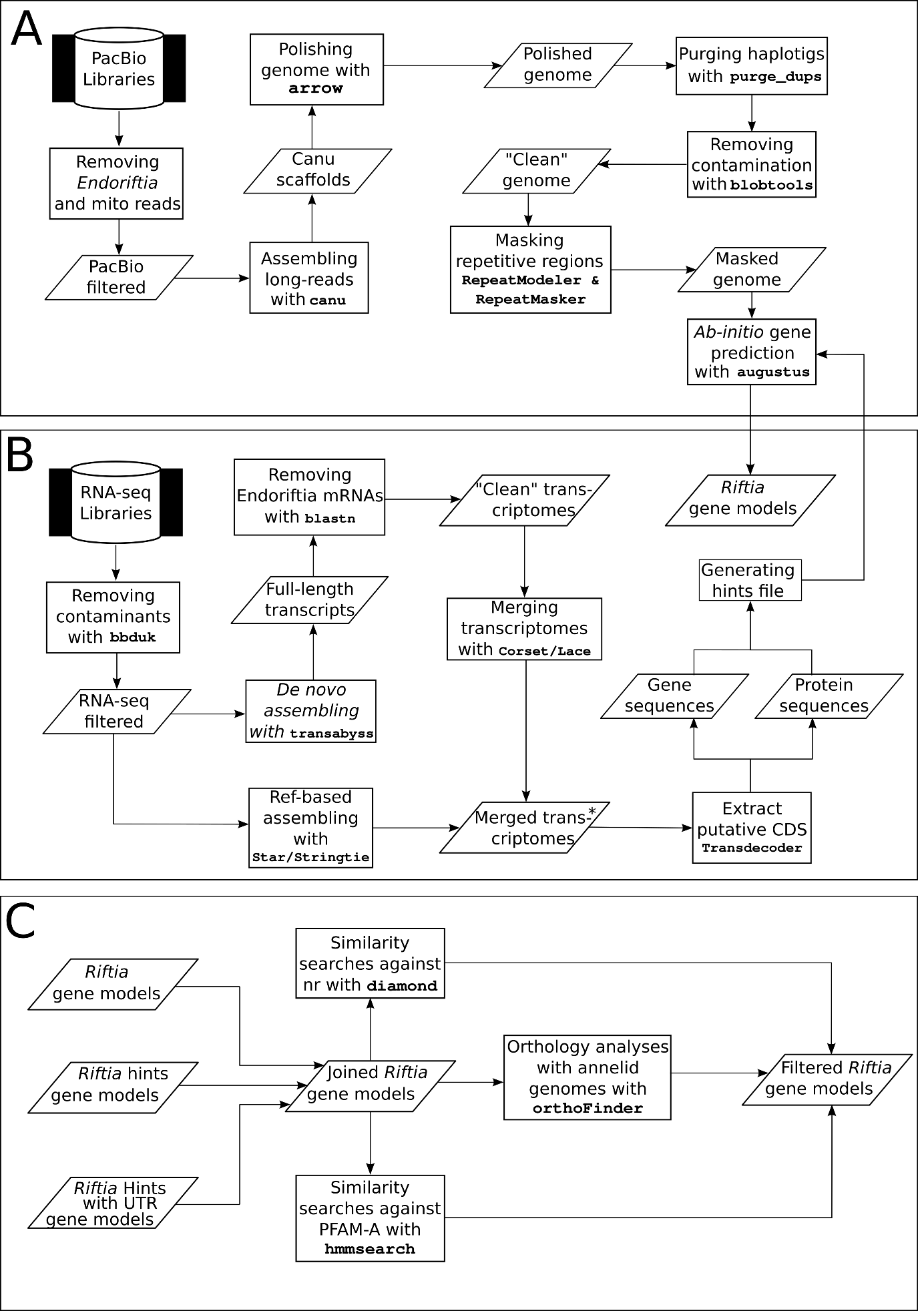


**Supplementary figure 61 | Bioinformatic workflow used in this study**. **A**, *Riftia pachyptila* genome pipeline for pre-processing, assembling and gene prediction. Mitochondrial and *Ca.* Endoriftia persephone reads were removed from the five PacBio libraries. The filtered long reads were assembled, polished and finally, the haplotigs purged from the final assembly. Contamination screening was performed, and the identification and masking of the repeat regions executed with RepeatModeler and RepeatMasker, respectively. Gene prediction with the polished, cleaned, soft-masked genome was carried out. **B**, *Riftia pachyptila* transcriptome pipeline. Illumina adaptors and low-quality bases were removed from the RNA-seq libraries. Pre-processed libraries were de-novo and reference-based assembled. Putative coding sequence regions were identified after the removal of contaminant transcripts. Hint files based on the transcriptome data were used to aid the gene prediction based on genome sequences. **C**, Gene models were joined and filtered based on homology searches, orthology identification and gene expression (not shown). *Two sets of merged transcriptomes were generated: one containing all de-novo transcriptomes, and other containing all reference-based transcriptome assemblies.

# **SUPPLEMENTARY NOTES**

# **Supplementary note 1 | *Riftia* genome and transcriptome sequencing, assembling and assessment**

We collected and then, using PacBio™ technology, sequenced the whole genome and eight tissue-specific transcriptomes of the giant tubeworm *Riftia pachyptila* (short *Riftia*) (Supplementary Figures 1-3; Supplementary Table 1). The long-read sequencing associated with the developed bioinformatic pipelines produced a highly contiguous and complete *Riftia pachyptila* draft genome. The results are comparable and, in some cases, surpass the overall quality (e.g., N_50_, number of scaffolds) of other publicly available lophotrochozoan genomes (Simakov et al. 2013; Albertin et al. 2015; Luo et al. 2015; Sun et al. 2017; Wang et al. 2017; Luo et al. 2018; Belcaid et al. 2019; Calcino et al. 2019; Li et al. 2019; Sun et al. 2020). The assembled draft genome of the giant tubeworm was smaller than the predicted genome size studies (Dixon et al. 2001; Bonnivard et al. 2009) (Supplementary Figure 4). Specifically, the *Riftia* tubeworm genome is 128Mb smaller than the close relative *Lamellibrachia* *luymesi* (genome size of ~688Mb) (Li et al. 2019). To further validate the estimated genome size, a second tool called Flye (Kolmogorov et al. 2019), which implements a quite different long-read assembly algorithm compared to the primary assembler choice Canu (Koren et al., 2017), was employed. Additionally, a genome size estimation was performed with GenoneScope (http://qb.cshl.edu/genomescope/). The three independent analyses point to a giant tubeworm genome size ranging from ~510Mb to ~560Mb. Notably, though horizontal gene transfer in metazoan-bacteria endosymbiotic systems have been previously described (Nikoh and Nakabachi 2009; Husnik et al. 2013; Sloan et al. 2014; Ip et al. 2021), our analyses did not find any evidence of DNA transmission from *Candidatus* Endoriftia persephone to the giant tubeworm. However, more sensitive analyses should be employed (as described in Ip et al., 2021) to corroborate this hypothesis.

*Riftia pachyptila* genome contains the lowest repeat content among annelids with 29.9% of the genome composed of repeat regions (*Capitella*: 31%, *Helobdella*: 33%; *Lamellibrachia*: 36.92%) (Simakov et al. 2013; Li et al. 2019). As a matter of fact, *Riftia* repeat content is relatively smaller than most lophotrochozoans, being surpassed only by the scaly-foot snail *Chrysomallon squamiferum*, the brachiopod *Lingula anatina* and the limpet *Lottia gigantea* (25.5%, 22% and 21%, respectively) (Luo et al. 2015; Luo et al. 2018; Sun et al. 2020). Comparative genomic analyses showed different histories of repeat element expansions between the two tubeworms *Riftia* and *Lamellibrachia* (Li et al. 2019). The estimated proportion of repeat element classes varies significantly between the two annelids with *Lamellibrachia* showing a higher abundance of LINES, LTR and DNA elements. *Riftia* LTR and DNA repeat composition is more similar to the leech *Helobdella* and the polychaete *Capitella* than the closest relative *Lamellibrachia* suggesting an independent expansion of repeat elements in the *Lamellibrachia* lineage (Supplementary Figure 5).

With 25,984 protein coding genes and BUSCO4 (Simão et al. 2015) score of 99,37% (complete and partial) the giant tubeworm genome is the most complete annelid genome to date and ranks among the best lophotrochozoan genomes publicly available (Albertin et al., 2015; Belcaid et al., 2019; Calcino et al., 2019; Ip et al., 2021; Li et al., 2019; Luo et al., 2018, 2015; Simakov et al., 2013; Sun et al., 2020, 2017; Zhang et al., 2012). Compared to other animals that have an established symbiotic relationship with chemoautotrophic bacteria, *Riftia pachyptila* and *Chrysomallon squamiferum* (Sun et al. 2020) harbour the lowest number of coding sequence genes. The tubeworm *Lamellibrachia luymesi* (Li et al. 2019), and the bivalves *Bathymodiolus platifrons* and *Modiolus philippinarum* present between 33,584 and 38,998 coding sequence genes (Sun et al. 2017).

The number of transcripts obtained from the *de novo* assembled male (blood and sperm) and female (plume, vestimentum, trophosome, gonad, blood, and body wall) transcriptomes ranges between 122,284 (body wall - skin) and 279,949 (trophosome) (Supplementary Table 1D-F). Similarity searches with blastn (>90% identity) between the assembled transcriptomes and the genomes of *Riftia* and *Ca.* Endoriftia persephone identified the presence of endosymbiont reads across all tissues, with the majority located in the trophosome sample, as expected. The bacterial reads found in the non-trophosome tissues are certainly linked to cross-contamination during the sample extraction and collection.

The mapping rate for six out of eight assembled *de novo* transcriptomes against the unassembled transcriptome reads was higher than 95% attesting the quality of the *de novo* assemblies. Interestingly, the mapping rates for the trophosome and female blood tissues were ~62% and ~84% indicating misassemble errors and fragmentation during the *de novo* assembly procedure, since strict quality filtering were applied. This idea is further substantiated by the elevated number of Endoriftia reads in these two samples, which probably complicated the *de Bruijn* generation leading to the aforementioned problems.

An average of ~75% of the transcripts, with similarity against the *Riftia* reference genome, present in each of the eight individual *de novo* transcriptomes were annotated at a protein level against the nr database, suggesting novel uncharacterised genes present in the *Riftia* genome. A similar scenario was found in specific-tissue type transcriptomes of the scaly-foot snail *Chrysomallon squamiferum* (Sun et al. 2020). Whether these novel genes found in *Riftia* transcriptome tissues reflect the poor taxon coverage of closely related siboglinids/vestimentiferans in the protein database or lineage-specific genes, remains to be shown.

The reference-based transcriptome assemblies using the *Riftia* genome draft contain between 25,227 (skin – body wall) and 72,006 transcripts (trophosome) mirroring the trend obtained in the *de novo* reconstruction procedure. The elevated number of transcripts in the skin and trophosome compared to the other six tissues may indicate fragmentation of the reconstructed transcripts. The deep sequencing, tissue-specific transcriptomes and different gene prediction protocols enabled the identification of many alternative splice isoforms (average of ~2,2 isoforms per protein coding gene) in the *Riftia* genome.

# **Supplementary note 2 | Developmental genes and signalling molecules**

During the early development, *Riftia* metatrochophore larvae are actively and specifically infected with *Ca*. Endoriftia persephone, which triggers the development of the trophosome and the reduction of the digestive system (Bright et al. 2013). The transient larval digestive system is divided into three distinct regions, foregut, midgut, and hindgut, with the foregut and hindgut formed by the ectoderm, whereas the midgut presents an endodermal origin (Jones and Gardiner 1989; Arendt et al. 2001). Many genes involved in the bilaterian gut development have been studied in annelids, such as *Platynereis*, *Capitella*, *Helobdella*, and *Hirudo medicinalis* (Rosa et al. 2005; Kulakova et al. 2007; Boyle and Seaver 2008; Hui et al. 2009). However, no available data focusing on the genes responsible for the development of the digestive tract are available for vestimentiferans. *Brachyury*, *goosecoid*, *fork head*, and the three Parahox genes *xlox*, *gsx* and *cdx* involved in the developing foregut, midgut and hindgut are present in the giant tubeworm genome. Gene expression quantification in *Riftia* showed little or no expression of these genes in the tubeworm adult tissues. The presence of *brachyury*, *fork head*, *goosecoid*, and all ParaHox genes in *Riftia* enable future comparative studies on the molecular mechanisms and evolution guiding the transient larval digestive system (Supplementary Figures 8-10)

Hox and ParaHox genes are two of the most investigated gene families in developmental biology (Supplementary Figures 8-9). They are remarkably conserved across invertebrates and vertebrates and are commonly organized in genomic clusters. By comparing the Hox gene distribution in Annelida, it can be inferred that the last common annelid ancestor had at least 11 Hox (*hox1-5,* *lox5*, *hox7*, *lox4*, *lox2*, *post1-2* (Kulakova et al. 2007; Simakov et al. 2013; Zwarycz et al. 2016). *Riftia* contains all Hox genes, except *hox7* (*ATNP)*. Our genomic screening did not identify *hox7*, *lox2* and *lox4* in the cold-seep tubeworm. Surprisingly, the identification of *lox2* and *lox4* in *Lamellibrachia* has been recently reported (Sun et al. 2021), directly contradicting our results. Multiple sequence alignments with the identified *lox2-4* in *Lamellibrachia* together with their respective orthologs in *Riftia*, and other lophotrochozoans, indicated that both Lox genes in the cold-seep tubeworm (LAMLU_FUN_038045 and LAMLU_FUN_038046) lack the *bona-fide* homeodomain present in homeobox transcription factors. Additional protein sequence similarity searches showed, however, a high sequence similarity and conservation of the 5′-flanking regions in both *Lamellibrachia lox4* and *-2* compared to *Riftia* orthologs, indicating that the lack of homeodomain might be related to a gene prediction issue. *In-depth* investigations of the genomic and gene coding regions around the identified *lox-4* and *-2* genes identified the probable homeodomain region of *lox4* (LAMLU_FUN038046 - position 137856-138070 on scaffold 869). Unfortunately, we did not find any putative homeodomain coding region neighbouring the *lox2* gene. We did not find either any support for the presence of *lox4* and *-2* in the available *Lamellibrachia* transcriptomes (Li et al. 2019). Considering that siboglinids share the same early developmental stages and segmentation patterns (Young et al. 1996; Southward 1999; Nussbaumer et al. 2006), it is plausible that the absence of *lox-2* and *-4* in *Lamellibrachia* reflects an erroneous gene prediction, rather than a true secondary loss of those genes. Nonetheless, further investigations and more accurate gene predictions are necessary to truly corroborate their presence in the *Lamellibrachia* genome (Yanan Sun, personal communication).

Duplications and losses of Hox genes are a common feature in lophotrochozoans (e.g, *hox1-5* and *host-2* are duplicated in the nemertean *Notospermus geniculatus, hox2-4* is lost in the cephalopod *Octopus bimaculoides*) (Albertin et al., 2015; Luo et al., 2018). Within Annelida, duplications, and losses of Hox genes have also been reported. In the leech *Helobdella robusta*, *hox1* (*Lab*), *hox4* (*Dfd*), *hox5* (*Scr*) and *lox4* contains 2, 2, 4 and 2 copies, respectively, whereas *hox2* and *post1* are missing (Simakov et al. 2013). The polychaete annelids *Nereis virens* and *Capitella teleta* both contain the complete set, in contrast to *Platynereis dumerilii* (also a polychaete) which does not possess the *hox7* and *lox4* (Kulakova et al. 2007; Simakov et al. 2013). The *Riftia* genome does not show any sign of duplicated Hox and ParaHox genes, however the Hox-like elements *engrailed* (*En*) and *even-skipped* (*Eve*) present multi-copies in the giant tubeworm genome (two and four, respectively). Duplication of *en* is reported in the deep-vent snail *Chrysomallon squamiferum* (Sun et al. 2020)*. Riftia* and *Lamellibrachia* genomes contain the three ParaHox genes. The Hox cluster in *Riftia* is almost intact, with only the *post1* located in a different scaffold. The presence of the almost complete Hox gene cluster and complement, as well as other developmental genes, attest for the quality and contiguity of the *Riftia* draft genome.

The transforming growth factor-β (TGFβ) family, responsible for cell fate specification and embryonic development, contains 33 members, including bone morphogenetic proteins (BMPs), growth and differentiation factors (GDFs), activins and *nodal* (Moustakas and Heldin 2009; Massagué 2012). *Riftia* genome contains 13 TGFβ members, including lefty, reported to be a deuterostome innovation (Simakov et al. 2015). *Lefty* has been reported to be present in the nemertean *Notospermus* and the brachiopod *Lingula* (Luo et al. 2018), our analyses found its presence, in addition to *Riftia*, in the *Lamellibrachia* and *Capitella* genomes (Supplementary Figure 11). The domain composition of *lefty* in all lophotrochozoans differs from the deuterostome ortholog, with only the presence of TGF_propeptide protein domain (PF00688). The *lefty* gene models on *Riftia*, *Lamellibrachia* and *Capitella* are complete and not fragmented as previously reported in the *Notospermus* and *Lingula* (Luo et al. 2018), suggesting that the TGF_beta domain (PF00019) is indeed not present in Lophotrochozoa. Broader taxon sampling and comparative genomics are required to elucidate whether both domains were present in the last common bilaterian ancestor.

The giant tubeworm genome contains a single copy of hedgehog and notch ligands, whereas the close relative *Lamellibrachia luymesi* contains two and one copy, respectively (Supplementary Figures 12-13). Additionally, our gene screening and phylogenetic analysis identified a single copy of hedgehog and notch ligands in the polychaete *Capitella* and the leech *Helobdella robusta*, corroborating previous studies on these annelid model species (Kang et al. 2003; Rivera et al. 2005; Seaver and Kaneshige 2006; Thamm and Seaver 2008). Overall, numbers of hedgehog and notch ligands in annelids are smaller than other lophotrochozoans, such as nermerteans, phoronids and molluscs. *Phoronis australis* contains, for example, 13 hedgehog and three notch ligand homologs (Luo et al. 2018). An expansion of the Hedgehog receptor dispatched, a multipass membrane protein that facilitates the transport of cholesterol modified Hedgehog (Burke et al. 1999), was identified in the Vestimentifera lineage compared to the other two annelids herein analysed.

The Wnt gene family is involved and cell fate specification and regulation of posterior growth during early animal embryogenesis (Niehrs 2012). The giant tubeworm, *Lamellibrachia* and *Capitella* genomes have all the 12 expected lophotrochozoan Wnt genes (*wnt-A*, *wnt1*, *wnt2*, *wnt4-11*, *wnt16*), indicating that the last common annelid ancestor harboured the full Wnt lophotrochozoan complement (Cho et al. 2010; Luo et al. 2018). *Wnt3* is lost in the Protostomia lineage (Holstein 2012). *Lamellibrachia luymesi* genome present two lineage-independent duplications of *wnt7* and *wnt1*. Independent losses (*wnt-A*, *wnt9-10*) and duplications of Wnt subfamilies are also found in *Helobdella robusta* (*wnt5*, *wnt11* and *wnt16*), as previously reported (Cho et al. 2010). We identified four Wnt-receptor Frizzled subfamilies in *Riftia*, with *fz-4* containing three paralogous copies in the giant tubeworm genome. The Wnt antagonist *sFRP3/4* (Cruciat and Niehrs 2013), despite being identified in lophotrochozoans (Luo et al. 2018), is missing from *Riftia* genome (Supplementary Figures 14-15).

# **Supplementary note 3 | Orthology and gene family analyses**

The orthology analyses using 36 metazoan taxa (4 non-bilaterians, 19 lophotrochozoans, 6 ecdysozoans, 7 deuterostomes – Supplementary Table 2) showed that *Riftia* and *Lamellibrachia* present the highest number of orphans orthogroups within the annelid taxa herein analysed (*Riftia*: 8,132; *Lamellibrachia*: 10,262; *Capitella*: 4,300; *Helobdella*: 4,821). In agreement with recent studies (Fernández and Gabaldón 2020), the distribution of metazoan, bilaterian, protostomian, lophotrochozoan and lineage-specific orthogroups within Annelida follows a bimodal distribution, with a massive gain of genes at the deep (i.e., last common metazoan ancestor) and recent nodes (i.e., taxonomically restricted genes).

Analyses with topGO using *Lamellibrachia* and *Riftia* lineage-specific genes revealed different patterns of gene enrichment (Supplementary Figures 21-24; Supplementary Table 5). We found significant enrichment (p < 0.05) of terms involved in chitin synthesis and secretion in the *Riftia* genome. Chitin is the major component of the tube of *Riftia* and the most abundant biopolymer in nature (Zakrzewski et al. 2014). *Riftia* alone, during the development of its tube, is responsible for the highest production of chitin recorded in marine environments, producing ~100 times more than any other marine animal (Shillito et al. 1995; Gaill et al. 1997). Produced by specialised large multi-cellular gland cells in the plume, trunk and opisthosome region, the tubes of *Riftia* are highly stable structures, with a lower degradation rate than other vent animals, such as crabs (Shillito et al. 1993; Shillito et al. 1995; Ravaux et al. 2003). The expanded complement of chitin-related genes in *Riftia* further corroborates the important role of this complex macromolecule against toxic vent chemicals and predators in the vent ecosystem (Shillito et al. 1993; Shillito et al. 1995; Ravaux et al. 2000; Ravaux et al. 2003). These results open the possibility of comparative studies focused on the exoskeleton protein synthesis and secretion pathways on *Riftia* and other vent animals.

Among the lineage-specific genes in *Lamellibrachia*, we found enriched terms associated with G protein-coupled receptor (GPCRs) (Supplementary Table 6). GPCRs are evolutionary conserved protein families that trigger signal transduction pathways having important roles in physiological responses to hormones, neurotransmitters, and environmental inputs (Fredriksson and Schiöth 2005). Additionally, PFAM protein domain analysis showed that the complement size of GPCR (7tm_1; PF00001) proteins in *Lamellibrachia* greatly surpasses other (Albertin et al., 2015; Simakov et al., 2013) (*Lamellibrachia luymesi*: 631; *Octopus bimaculoides*: 328; *Lottia gigantea*: 267). As a matter of fact, Fisher’s exact test showed that GPCR-containing proteins are expanded (p < 0.05) in the cold-seep tubeworm in relation to *Riftia pachyptila* (308). The presence of many lineage-specific GPCRs in *Lamellibrachia* raises interesting questions about the evolutionary history of this large repertoire and its association with sensory information and homeostatic regulation in the cold-seep tubeworm. Our PFAM protein domain analysis indicated that *Capitella teleta* genome harbours the highest number of GPCR genes in lophotrochozoans (963), results corroborated by previous studies (Simakov et al. 2013; Albertin et al. 2015; Ritschard et al. 2019). Further fine-grained analyses on the evolution of GPCR families are necessary to elucidate patterns of gene gains and losses in the different nodes of the Annelida tree.

PFAM enrichment analysis in the *Riftia* genome showed the expansion of protein domains present in high-molecular mass proteins such as collagen, laminin, nidogen (Supplementary Table 6). Clustering analysis with the expanded proteins involved in the production of the basement membrane in *Riftia* showed that these proteins are all connected to each other, indicating evolutionary relatedness (Supplementary Figure 24). However, due the presence of many tandem repeats within these proteins, such as EGF, the clustering probably reflects the reoccurring low complexity repeats in these proteins.

Pairwise comparisons of the enriched/contracted PFAM terms present in the genomes of symbiotic deep-sea animals, *Riftia pachyptila*, *Lamellibrachia luymesi*, *Bathymodiolus platifrons* and *Chrysomallon squamiferum*, against selected lophotrochozoans (N = 14; Supplementary Table 6; Supplementary Figure 24A) identified few shared protein domains. Among them, the zinc-finger H2C2/Integrase H2C2 DNA-binding domains (*Riftia*: 0/127; *Lamellibrachia*: 4/115; *Bathymodiolus*: 9/136 *Chrysomallon*: 1/99) and peptidase A17 (domain not found in any symbiotic deep-sea animal). Interestingly, C1q domain-containing proteins, regarded as key players in the innate immunity and pathogen recognition (Thielens et al. 2017), are contracted in *Riftia* (2), *Lamellibrachia* (3) and *Chrysomallon* (6) in relation to other lophotrochozoans (average = 69). The genome of *Bathymodiolus*, in contrast to the other deep-vent symbiotic animals, contains an expansion of C1q domains (386), as reported in the pacific oyster *Crassostrea gigas* and in the Mediterranean mussel *Mytilus galloprovincialis* (Gerdol et al. 2011; Gerdol et al. 2015; Gerdol et al. 2019).

The *Riftia* genome contains the smallest number of transcription factors (TFs) within Annelida with a complement of 414 genes (*Lamellibrachia luymesi*: 423; *Capitella teleta*: 551; *Helobdella robusta*: 568) (Supplementary Table 3; Supplementary Figure 19). The nuclear factor zf-C4, bHLH (helix-loop-helix) and the zinc finger C2H2 domain containing genes are underrepresented in the giant tubeworm genome in comparison to the other three annelid genomes. We did not identify any notable TF lineage-specific expansion in *Riftia*. Our analyses retrieved the exact same number of homeobox domain containing genes in *Riftia* and *Lamellibrachia* (122 genes), making the homebox complement in the vestimentiferans smaller than the other two annelids. A recent study on the homebox complement of the oligochaete *Eisenia fetida* showed that the earthworm experienced many gene gains events totaling 363 homebox genes, surpassing the numbers in many deuterostomes, ecdysozoans, lophotrochozoans and annelids (Zwarycz et al. 2016). The homeobox complement herein reported is in agreement with a previous study (Zwarycz et al. 2016) (*Capitella*: 178 / our study 160; *Helobdella*: 270 / our study 246), showing the robustness of our methods.

Loss and gains of TFs have been reported across many metazoan lineages, and as two examples we could cite the cephalopod *Octopus bimaculoides* and the parasite tapeworms (Tsai et al. 2013; Albertin et al. 2015). The first shows a massive expansion of the C2H2 superfamily of zinc-fingers transcription factors, which regulate neuronal development, and the later a broad reduction of the homebox complement. These changes in the TF complement sizes are related to the large and complex nervous system and adaptation to parasitic lifestyle in the cephalopod and tubeworm, respectively. More thorough investigations are required to establish if the reduced TF numbers in *Riftia* and *Lamellibrachia* is linked to the loss of anatomical structures in these two tubeworms.

One unexpected result derived from CAFE and PFAM analyses was the contraction of the histone gene families in the giant tubeworm genome in relation to other lophotrochozoans (N = 18) (Supplementary Tables 6). As histones are essential to the transcription machinery and condensation of eukaryotic DNA, their absence from *Riftia* genome caught our attention. Furthermore, few studies indicate their presence in the giant tubeworm (Rouse et al. 2015; Hinzke et al. 2019), challenging our initial results. To investigate this question, we performed additional local similarity searches with hmmscan (Mistry et al. 2013) using the histone HMM profile as query (PF00125) and the proteomes obtained from the de-novo transcriptomes as databases. Our similarity searches identified many copies of the core histones H2B, H2A and H3 in the *Riftia* tissues (vestimentum: 16; body wall (skin): 16; male blood:17; sperm: 18; female gonad: 17; female plume:15). Subsequent multiple sequence alignments, homology searches and phylogenetic inferences with the non-redundant histones set confirmed their presence in the giant tubeworm genome. The initial erroneous genome annotation of histone genes could be attributed to the over masking of repeat regions overlapping the histone coding sequence regions (Mario Stanke personal communication, May 2020).

**
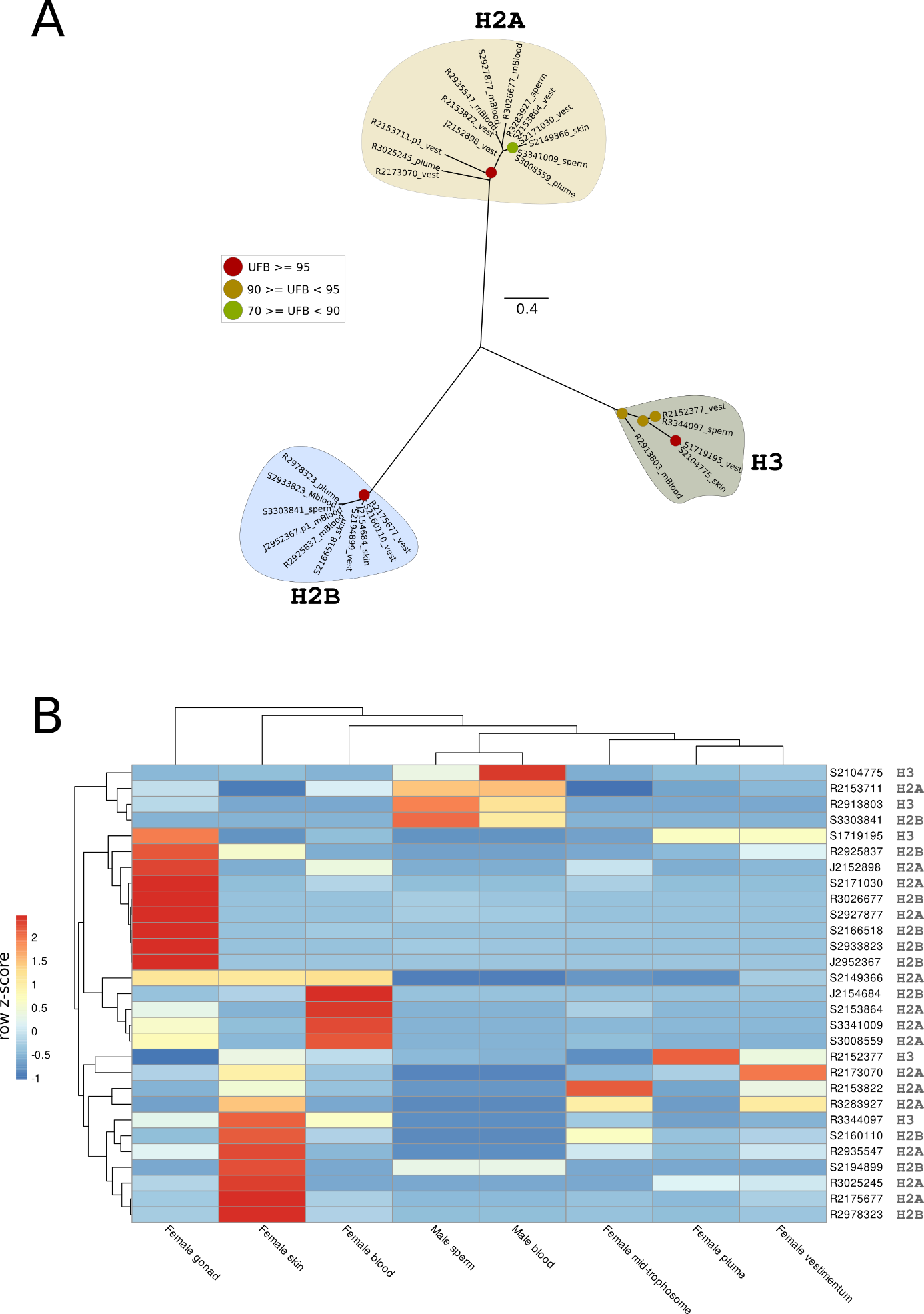
Additional supplementary figure 1 – Histone phylogeny and gene expression analysis. A,** Unrooted maximum-likelihood phylogenetic inference of the non-redundant core histone set (i.e., H2A, H2B and H3) found in different *de novo* transcriptomes. Leaf names correspond the transcript name followed by the tissue in which they were found. The branch support values are represented by the coloured circles in the tree nodes. Red circles represent ultrafast bootstrap values >= 95. Yellow circles represent ultrafast bootstrap values >= 90 and < 95. Green circles represent ultrafast bootstrap values < 90 and >= 70. Ultrafast bootstrap values smaller than 70 are not shown. **B**, Expression profile of histone genes *Riftia pachyptila*. colour coding reflects the expression patterns based on row Z-score calculations. Despite not identified in the genome prediction, histone genes, as expected, are present in the giant tubeworm genome. Histones are highly expressed on gonad and body wall (skin) tissues.

Hinzke et al. (2019) speculated that histones may have antimicrobial properties functioning as mediators of host-symbiont interactions on the trophosome and defence against environmental microbes on the plume. This hypothesis was based on previous observations that histones and histone-derived peptides modulate immune system responses in vertebrates (Fr et al. 1998; Park et al. 1998; Cho et al. 2009; Bishop et al. 2017). Contrary to the results described by Hinzke et al.(2019), we did not identify a relative high abundance of histone genes in the trophosome compared to other tissues, challenging their findings. We found that histone genes are constitutively expressed on *Riftia* tissues, with the gonad and skin presenting an elevated number of highly expressed histone genes. Expression of core histones is required during the S phase of cell-division cycle where the DNA replication commences (Mei et al. 2017). The expression of histones in the aforementioned *Riftia* tissues probably reflects the high levels of cell proliferation, especially in the skin as shown in immunohistochemical and ultrastructural cell cycle studies (Pflugfelder et al. 2009).

The expanded gene families identified by CAFE point to molecular adaptations associated with sulphide rich environments and symbiotic lifestyle in *Riftia* (Supplementary Table 4; Supplementary Figure 20). The toxicity of H2S is primarily linked to the inhibition of the cytochrome-c oxidase in the mitochondrial respiratory chain, which limits most animals to survive and reproduce in sulphide rich environments (Cooper and Brown 2008). *Riftia* has many expanded families enriched with GO terms associated with sulphur metabolism and detoxification (e.g., carbohydrate-, galactose- and heparan-sulphate-sulfotransferases). Recent comparative transcriptome and differential gene expression analyses with the shrimp *Rimicaris* sp. (Zhang et al. 2017), predominant megafauna of deep-sea hydrothermal vents, recovered a similar gene toolkit responsible for detoxification in the arthropod, indicating similar molecular mechanisms in different animals that live in similar sulphide-rich vent ecosystem.

Three expanded rapidly evolving gene families (i.e., positively selected) were detected in the *Riftia* genome: sushi-domain, glycosyltransferase, and mucin-related families (Supplementary Figure 4). Host sushi-domain proteins have been hypothesized to present symbiosis specific roles in sponges and cephalopods (Collins et al. 2012; Pita et al. 2018). Sushi domains are involved with innate immunity and self/non-self-recognition (Kirkitadze and Barlow 2001). The expansion of this gene family in *Riftia*, as well as *Lamellibrachia* (Li et al. 2019), could be associated with the recognition of the endosymbionts by the tubeworm hosts (Supplementary Figure 27). Furthermore, the expansion of glycosyltransferases is probably coupled with the enriched number of genes in *Riftia* involved in the formation of the basement membranes, such as collagen. Finally, independent expansions of mucin-related genes have been identified in other lophotrochozoans, such as phoronids and brachiopods (Luo et al. 2018). In *Lingula* and *Phoronis*, mucin genes are highly expressed in the lophophores and in the phoronid lineage they might be related to protection against predators. The function of mucus production in *Riftia* adults is less clear. However, during the symbiont infection model suggested by Nussbaumer et al., (2006), the metatrocophore larvae actively secrete a mucous coat during settlement in which the environmental free-living bacteria attach, including the *Riftia* endosymbiont. Interestingly, gene family analysis in *Lamellibrachia* recovered the same expansion of mucin-related genes (Li et al. 2019). Based on these observations, we suggest that the expanded mucin-related proteins in *Riftia* might play a role in the acquisition of the free-living endosymbionts from the environment during the larval settlement, scenario that could be generalised to *Lamellibrachia*.

Vestimentiferan specific genes present are involved in a range of biological processes, including oxygen transport, protein dephosphorylation, G protein-coupled receptor signalling pathway and cyclic nucleotide biosynthetic process.

# **Supplementary note 4 | Haemoglobin evolution**

*Riftia* possesses three complex multimeric haemoglobins (HBs), two of them dissolved in vascular blood (V1 and V2) and one in the coelomic fluid (C1) (Zal et al., 1996a, 1996a). The Hb complement of the giant tubeworm has been suggested to contain six distinct genes, from which three are classified as β1-Hb, and the remaining three belong to the paralog groups α1-, α2- and β2-Hbs(Bailly et al., 2002; Zal et al., 1997, 1996a). To further investigate the multigenic extracellular globin family in *Riftia*, we retrieved all Hb genes from the giant tubeworm genome and processed them through a phylogenetic pipeline. Using a large reference dataset (Belato et al., 2019), tree inferences and multiple sequence alignments we identified and annotated 26 extracellular Hbs in *Riftia* (Supplementary Figures 21 and 28; Figure 3). The paralog group β1-Hb is expanded on the giant tubeworm, whereas α1- and β2-Hbs groups contain only one copy each. The α2-Hb group contains two paralogous genes. The Hb complement of *Riftia* mirrored the complement of the cold-seep tubeworm *Lamellibrachia luymesi* (Li et al., 2019), suggesting an expansion of the β1-Hbs already at the base of Vestimentifera.

We propose a division of the β1-Hb chains into eight paralogous groups enriching the established classification of Baily et al (Bailly et al., 2002). In addition to the previous β1a-Hb, β1b-Hb and β1c-Hb groups, five more groups were recognised through phylogenetic analyses: β1d- to β1h-Hb. Seven out of the eight β1-Hb paralogous groups have recognisable orthologs in *Lamellibrachia luymesi*. In agreement with previous studies, the 12 conserved amino acid residues located in extracellular globins were present in the giant tubeworm and *Lamellibrachia* Hb genes (Belato et al., 2019; Gotoh et al., 1987; Li et al., 2019; Negrisolo et al., 2001; Shishikura et al., 1986; Yuasa et al., 1996) (Supplementary Figure 29) β1-Hb group members harbour a conserved motif, VNV[ADE] at positions 48-51 (considering the Cys-19, as the first residue of the alignment block), as previously reported (Bailly et al., 2002) (although variations were observed in *Riftia* and *Lamellibrachia* representatives belonging to β1d-, β1e-, β1h- and β1g-Hb groups). The residues 74-, 122- and 128-Ans, as well as 83-Glu, are relatively conserved in β-Hb groups. Free cysteine residues, associated with the sulphide binding function on vestimentiferans and other annelids living in permanent sulphide-rich environments (Bailly et al., 2002; Li et al., 2019; Pallavicini et al., 2001; Suzuki et al., 1995, 1989; Takagi et al., 1991; Yuasa et al., 1996; Zai et al., 1999; Zal et al., 1998, 1997), have been previously identified on the giant tubeworm α2- and β2-Hb chains. We found additional putative cysteine residues in β1e, β1f and β1g-Hb groups, totalling new seven Hb genes with the capability of carry H2S on the giant tubeworm. Our analyses revealed eight copies of *Lamellibrachia luymesi* genes containing putative free cysteine residues, as reported by Li et al (Li et al., 2019). Homology model generation reconfirmed the presence of free-cysteine residues, indicating the possible sulphide-binding capability of some members of the expanded tubeworm β1-Hb chain (Supplementary Figure 30). Comparisons between *Riftia* β1-Hb containing proteins and the two highest ranked templates (3WCT_D - deoxygenated haemoglobin from the tubeworm *Lamellibrachia satsuma*; 1YHU_C C1 haemoglobin from the tubeworm *Riftia pachyptila*) showed sequence similarity values ranging from 35.57% - 48.30% and 39.42% - 54.29%, respectively.

Tandem gene duplication, which is the main driving force behind the rise of multigene families, is an important mutational process in evolutionary adaptation and the evolution of eukaryotic genomes (Friedman and Hughes, 2001; Loehlin and Carroll, 2016). In lophotrochozoans, lineage-specific expansions through tandem duplications related to defence mechanisms, immune responses to pathogens and neuronal development, have been reported in nemerteans, phoronids and cephalopods, respectively (Albertin et al., 2015; Luo et al., 2018). In *Riftia*, the close chromosomal proximity of the expanded β1 chain genes and their phylogenetic relationship indicate that this multigenic family was originated through a series of tandem duplications. We identified seven genomic Hb-containing clusters on the giant tubeworm genome, from which six correspond to the β1 chain genes (Fig 3B). The position of some Hb genes in the end of scaffolds and their nested phylogetic pattern revealed in the tree inference, point to a more contiguous cluster containing the Hb chains. Additional sequencing (e.g., Hi-C method) and subsequent genome scaffolding are necessary to confidently elucidate the exact chromosomal organisation of the Hb complement in the *Riftia* genome.

Deep-sea hydrothermal vents are unstable environments marked by small-scale disturbances of the physicochemical conditions (Bright and Lallier 2010). The changes in pH, sulphide, oxygen, and temperature pose a challenge for the vent fauna, that must cope with these variations to thrive under these unpredictable conditions. As *Riftia* is extremely dependent upon its endosymbionts, the trophosome needs to be constantly nurtured with the necessary metabolites (e.g., carbon dioxide, sulphide, and oxygen) required from the bacteria to fix carbon through autotrophic pathways (Fisher et al. 1989). The transport of H2S and O2 substrates to the trophosome is carried out through the circulatory system and multimeric haemoglobin complexes. As expected, we found that representatives of all four Hb groups (α1-2, β1-2) are highly expressed in the trophosome tissue. Notably, gene expression quantification using publicly available transcriptome datasets (Hinzke et al. 2019) obtained from sulphide rich, medium, and depleted trophosome samples showed great variation of Hb expression. Many members of expanded β1 group are highly expressed on medium and sulphur-rich trophosome samples (β1a, b, c, d, e, f, g), including the Hbs containing the free-cysteine residues. The presence of highly expressed β1 genes in the trophosome has also been reported in the cold-seep tubeworm *Lamellibrachia luymesi* (Li et al. 2019). Despite the clear distinct expression patterns of the Hbs in the different trophosome samples, it is hard to associate these changes in expression levels with the exact environmental conditions which they were sampled. The qualitative classification of depleted, medium, and sulphur-rich employed by Hinzke et al. (2019) was based on the trophosome colour, which is directly linked to presence of elemental sulphur vesicles in the endosymbionts. As shown by previous studies (Fisher et al. 1988; Wilmot and Vetter 1990; Pflugfelder et al. 2005), variability of colours may occur in the same trophosome, as well as differences in metabolism of the distinct endosymbiont subpopulations within a single animal (Hinzke et al. 2021), making it difficult to clearly state the physicochemical conditions from which the trophosome samples were obtained. However, irrespective of the exact environment conditions, we could observe clearly distinct patterns of Hb gene expression, indicating a more specialised role of these genes in *Riftia*. The ubiquitous expression of the six linker genes in the sulphur-rich, medium, and depleted samples is consistent with the molecular organisation of the Hb complexes in *Riftia*, in which the linker genes act as structural non-globin component of the multimeric haemoglobin. We were unable to recover the monophyly of the linker groups, corroborating previous studies (Belato et al. 2019).

# **Supplementary note 5 | Comparative tissue-specific transcriptomics and gene expression**

Comparative transcriptome analysis of eight different *Riftia* tissues (plume, vestimentum, trophosome, body wall (skin), female gonad, sperm, and male/female blood) revealed distinct transcriptional landscapes (Supplementary Table 9). The vestimentum and body wall tissues (Supplementary Figures 25 e 26) harbour several tissue specific genes (TSGs) mainly involved in chitin metabolism responsible for the tube production and growth (Gaill et al. 1992; Shillito et al. 1993; Shillito et al. 1995). These results are not surprising, since these two organs contain conspicuous pyriform glands responsible for the chitin production and degradation (Jones 1981; Bright and Lallier 2010).

The branchial plume contains many TSGs involved with cell division and growth, signal transduction, immune system, and apoptosis. We also identified a high expression of immune response, endosomal, cell cycle, autophagy, and (anti-) apoptotic proteins in the plume (discussed into more details in the next section).

The female gonad tissue is enriched with proteins involved in genome integrity (e.g., DNA repair and damage checkpoint, telomerase maintenance and nucleotide excision repair), an important factor for subsequent internal fertilisation and healthy offspring (Additional supplementary figure 2). Interestingly, we found many TSGs in the female gonad tissue associated with methyltransferase activity indicating potential epigenetic regulation in the tubeworm female gonadal development. The sperm tissue is characterised by genes enriched with ion transmembrane transport functions, such as CatSper (Rahban and Nef 2020), which has been shown essential to mammalian sperm flagellum mobility, chemotaxis towards the egg, capacitation, and acrosome reaction (Brown et al. 2019).

The trophosome tissue is characterised by many TSGs involved with mitochondrial activity (Supplementary Figure 33) (e.g., oxidoreductase activity, mitochondrial fission, mitochondrial ribosomal translation), beta oxidation of fatty acids, oxygen transport (e.g., oxygen biding), haemoglobin metabolism (e.g., iron ion binding, iron-sulphur cluster biding, heme biding, porphyrin and tetrapyrrole metabolism, 5-aminolevulinate synthase), lysosomal activity (e.g., threonine-type endopeptidase activity, peptidyl-dipeptidase activity), protein translation (e.g., ribosome, structural constituent of ribosome), and urea cycle (e.g., hydroxyisourate hydrolase activity).

The presence of TSGs in the trophosome related to 5-aminolevulinate synthase, porphyrin metabolism, and metal ion biding indicates that this tissue harbours the enzymatic machinery necessary for haem biosynthesis. Haem is an integral part of haemoglobin molecules, which is synthesized in a multistep pathway that begins and ends in the mitochondrion. The process starts with the formation of δ-aminolevulinic acid (ALA) catalysed by the enzyme 5-aminolevulinate synthase from glycine and succinyl-CoA (Malik and Djaldetti 1979). ALA migrates to the cytoplasm and through a series of enzymatic reactions involving seven universally conserved enzymes it is converted into haem. The last two steps mediated by the enzymes protoporphyrinogen oxidase (PPOX) and ferrochelatase (FeCH), which are responsible for the dehydrogenation of trotoporphyrinogen IX and its chelation with iron to produce the haem molecule, is located inside the mitochondria (Supplementary Figure 40) (Ajioka et al. 2006; Kořený et al. 2013; Celis and DuBois 2019).

TSGs belonging to the mitochondrial carrier family were found in the trophosome, including the mitochondrial coenzyme a transporter slc25a42, solute carrier family 25 (carnitine/acylcarnitine translocase member 20), mitochondrial ornithine transporter; and tricarboxylate transport protein (Additional supplementary figure 4; Supplementary Table 11). Mitochondrial carriers are widespread in eukaryotic organisms and lineage-specific variations have been reported (e.g., 35, 58 and 67 mitochondrial carriers are reported in the yeast *Saccharomyces cerevisae,* the plant *Arabidopsis thaliana* and in humans, respectively) (Wohlrab 2006; Monné et al. 2015). Similarity searches using the PFAM model PF00153 (mitochondrial carrier protein domain) against the giant tubeworm genome identified the presence of 45 mitochondrial carriers in *Riftia* (Additional supplementary figure 4). The giant tubeworm mitochondrial carriers are involved in the transport of different substrates such as amino acids, coenzymes, and nucleotides.


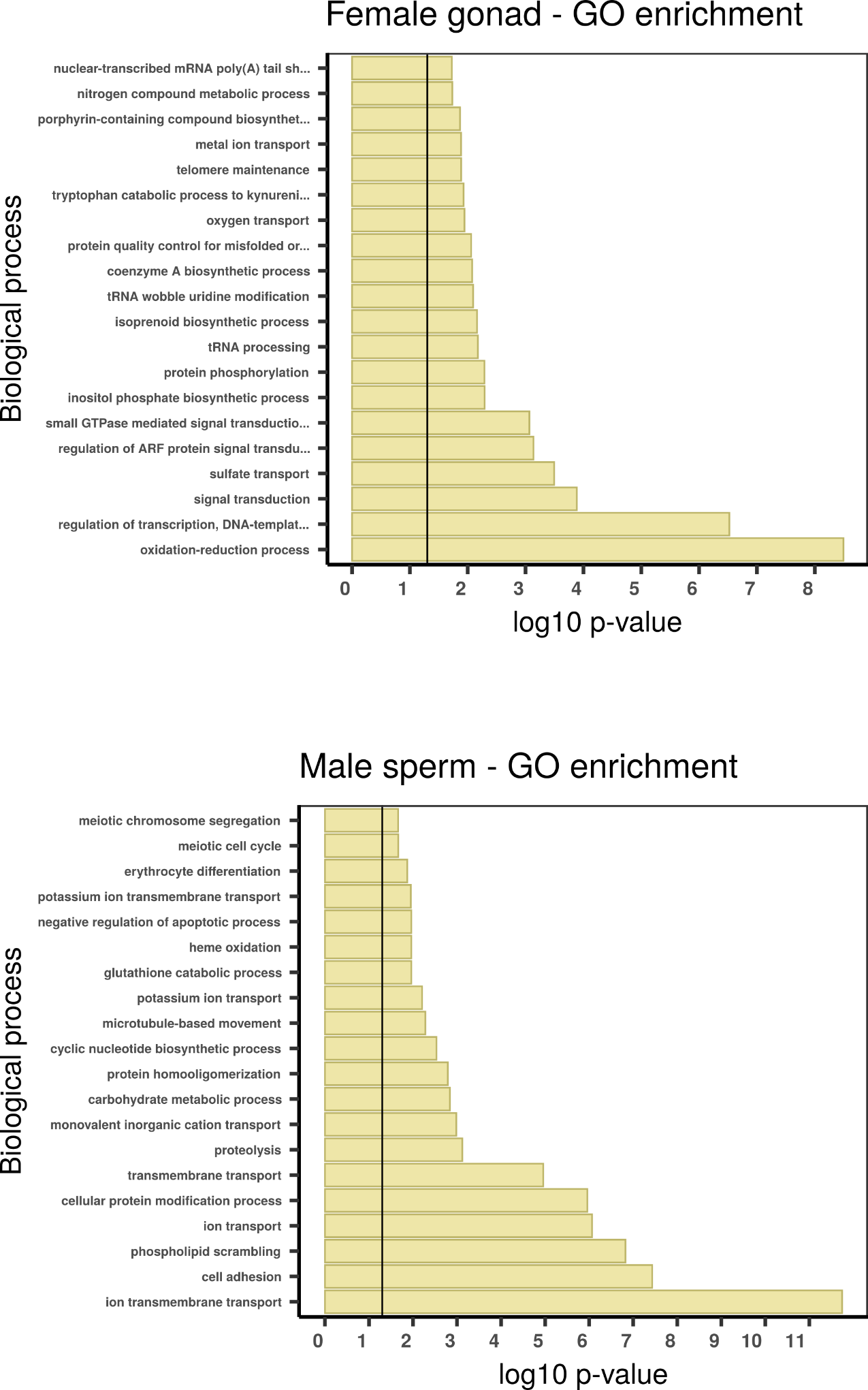


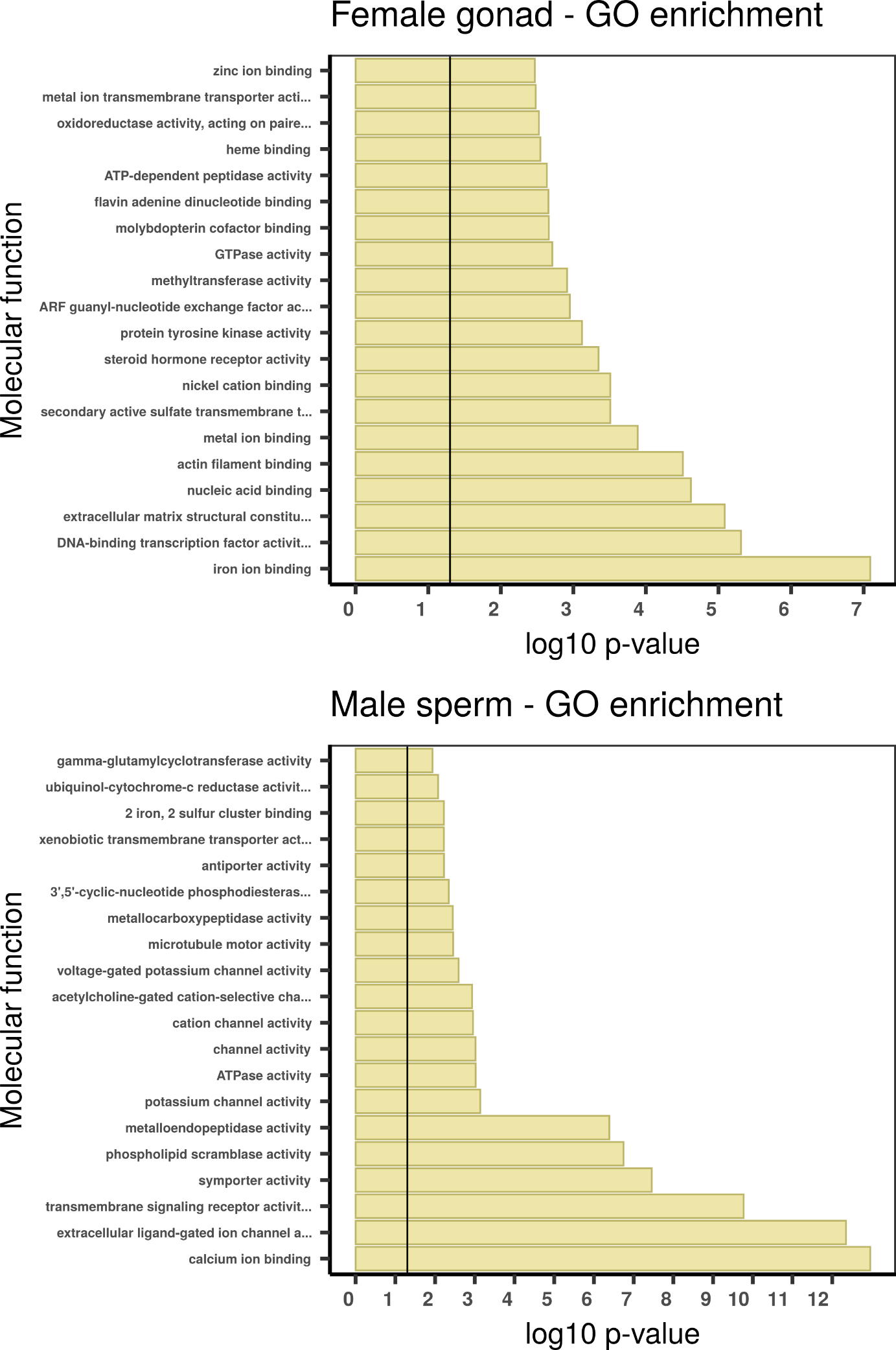


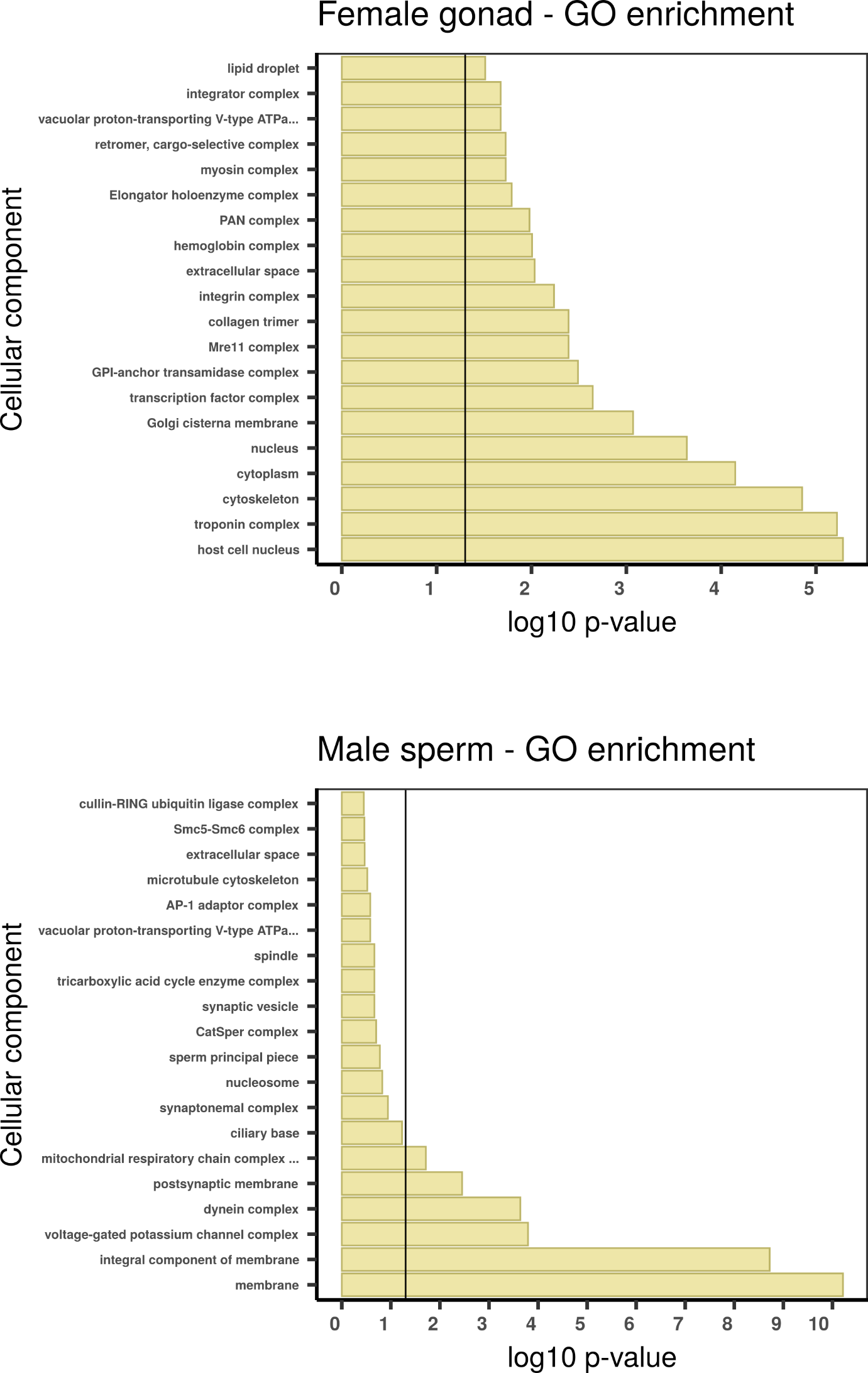


**Additional supplementary figure 2 -** Gene ontology (GO) enrichment analyses for absolutely blood specific TAU genes. The graphs correspond to the three domains of ontologies: biological process (BP), molecular function (MF) and cellular component (CC). The selected genes were analysed for enrichment in specific GO categories using the TopGO program against the background (all coding sequence genes). Y axis corresponds to enriched GO terms found in the respective domains (BP, MF and CC). X axis correspond to the log function of Fisher p-values obtained for each one of the enriched terms. The back line denotes a p-value = 0.05. P-values greater than 1,30 (log 0,05) indicate statistically significant enriched term.

Important genes part of the mitochondrial β-oxidation pathway involved in the catabolism of fatty acids were highly expressed in the trophosome, including the mitochondrial trifunctional protein and acyl-CoA dehydrogenases. The degradation of fatty acids by mitochondrial β-oxidation in trophosomal tissues has also been reported by Hinzke et al. (2019). Since acyl-CoA dehydrogenases (ACADs) constitute a multigene family, we performed similarity searches followed by phylogenetic inferences of well-annotated metazoan/lophotrochozoan ACAD proteins using homologs retrieved from the giant tubeworm and other annelid genomes (Swigoňová et al. 2009). Our analyses revealed that the *Riftia* genome contains 12 genes belonging to nine recognised ACAD subfamilies: ACAD-8 (isobutyryl-CoA dehydrogenase), -9, -10, SCAD (short-chain acyl-CoA dehydrogenase), MCAD (medium-chain acyl-CoA dehydrogenase), SBCAD (short/branched-chain acyl-CoA dehydrogenase), GCD (glutaryl-CoA dehydrogenase), IVD (isovaleryl-CoA dehydrogenase), and VLCAD (very long-chain acyl-CoA dehydrogenase) (Figure 4B, C and D). The subfamilies SCAD, MCAD, VLCAD, ACAD-9 and -10 are involved in the catabolism of fatty acids (Ye et al. 2004; Swigoňová et al. 2009), the remaining 4 families participate in the degradation of amino acids. We did not identify in *Riftia* any gene belonging to the LCAD subfamily (long-chain acyl-CoA dehydrogenase).

Genes involved with the TCA cycle (e.g., succinyl-CoA synthetase, dihydrolipoyl lysine-residue acetyltransferase) and oxidative phosphorylation process (e.g., NADH Dehydrogenase, ATP synthase) (Supplementary Figure 9) also present tissue-specificity in the trophosome. The intense activity of the mitochondrial respiratory chain in the trophosome is corroborated by the high expression of superoxide dismutase 2 genes (*sod2*; located in the mitochondrial matrix – Supplementary Figure 38; Supplementary Table 9) which neutralise highly reactive superoxide radicals protecting the tissue against oxidate stress (Miao and St. Clair 2009). Other antioxidant system, methionine sulfoxide reductase (Supplementary Table 9) (Sreekumar et al. 2011) also plays a role in the prevention of oxidative damage in the trophosome.

As *Riftia* is nutritionally dependent on its chemolithoautotrophic endosymbionts, which are deficient in polyunsaturated fatty acids (PUFAs), it has been questioned how the giant tubeworm obtains these biomolecules. PUFAs are precursors of a number of molecules and have important roles in inflammatory/immune responses, and membrane fluidity (Wallis et al. 2002). Surprisingly, contrary to most vertebrates which lack the enzymatic machinery required to synthesise PUFAs, *Riftia* contains the ω3- desaturase gene and is capable of desaturate and elongate fatty acids from the endosymbionts to gain access to PUFAs (Phleger et al. 2005; Liu et al. 2017). As fatty acids provide useful insights into the vent trophodynamics, nutritional strategies, and adaptation to abiotic factors (e.g., homeoviscous adaptation (Sinensky, 1974)) we screened the giant tubeworm, and closely related annelid taxa, for fatty acid desaturase genes. We identified eight fatty acid desaturases in *Riftia*, including the ω3-desaturase gene previously reported (Liu et al., 2017) (Additional supplementary figure 6A). Interestingly, based on our phylogenetic inferences, a clade has been recovered containing two *Riftia* paralog sequences classified as ω3-desaturase with a one-to-one orthology relationship with the cold seep tubeworm *Lamellibrachia.* The gene model RIFPA_jg1449 is 100% identical to the previously described ω3-desaturase gene and 84% identical to the *Lamellibrachia* ortholog. The second putative ω3-desaturase gene (RIFPA_jg32120) is 47% similar to the ω3-desaturase described by Liu et al. (2017). Whether this newly identified paralog present a ω3-desaturase activity remains to be shown. The remaining six genes and their isoforms are involved in sphingolipid, stearoyl-CoA, and acyl-CoA desaturase activities. Despite desaturases are ubiquitously expressed in tubeworm tissues, tissue-specificity was observed (Additional supplementary figure 6B). The presence of ω3-desaturase genes in annelids (including vestimentiferans), molluscs, and nematodes are in accordance with recent reports that invertebrates have the ability to produce PUFAs (Kabeya et al. 2018).

We identified in the *Riftia* genome the presence of 17 distinct cathepsin genes (Supplementary Figures 34-35) (cathepsin-C:1; cathepsin-Unc:1; cathepsin-B:2; cathepsin-L:4; cathepsin-Z:3; cathepsin-O:1; cathepsin-F:1; cathepsin-S:4), whereas the close relative *Lamellibrachia* contains 26 representatives (cathepsin-C:1; cathepsin-Unc:1; cathepsin-B:5; cathepsin-L:7; cathepsin-Z:3; cathepsin-O:1; cathepsin-F:2; cathepsin-S:6). Members of five out of the eight different cathepsins family are highly expressed in the trophosome (cathepsins-C/L/B/S/Z). The high expression of cathepsins in different trophosome samples belonging to *Riftia* and *Lamellibrachia* has been described also by Hinzke et al. (2019) and Li et al. (2019), respectively. Additionally, we identified moderate/high expression of several other lysosomal proteins, such as glycosidases (hexosaminidase alpha - *hexA*), sulphatases (heparan N-sulfatase - *sgsh*), proteases (legumain - *lgmn*), phosphatases (acid phosphatase 5, tartrate resistant – *acp5*), sphingomyelinases (sphingomyelin phosphodiesterase 1 – *smpd1*), and lipases (phospholipase A2 group XV – *lypla3*) in trophosome, which are probably also involved in the endosymbiont digestion.


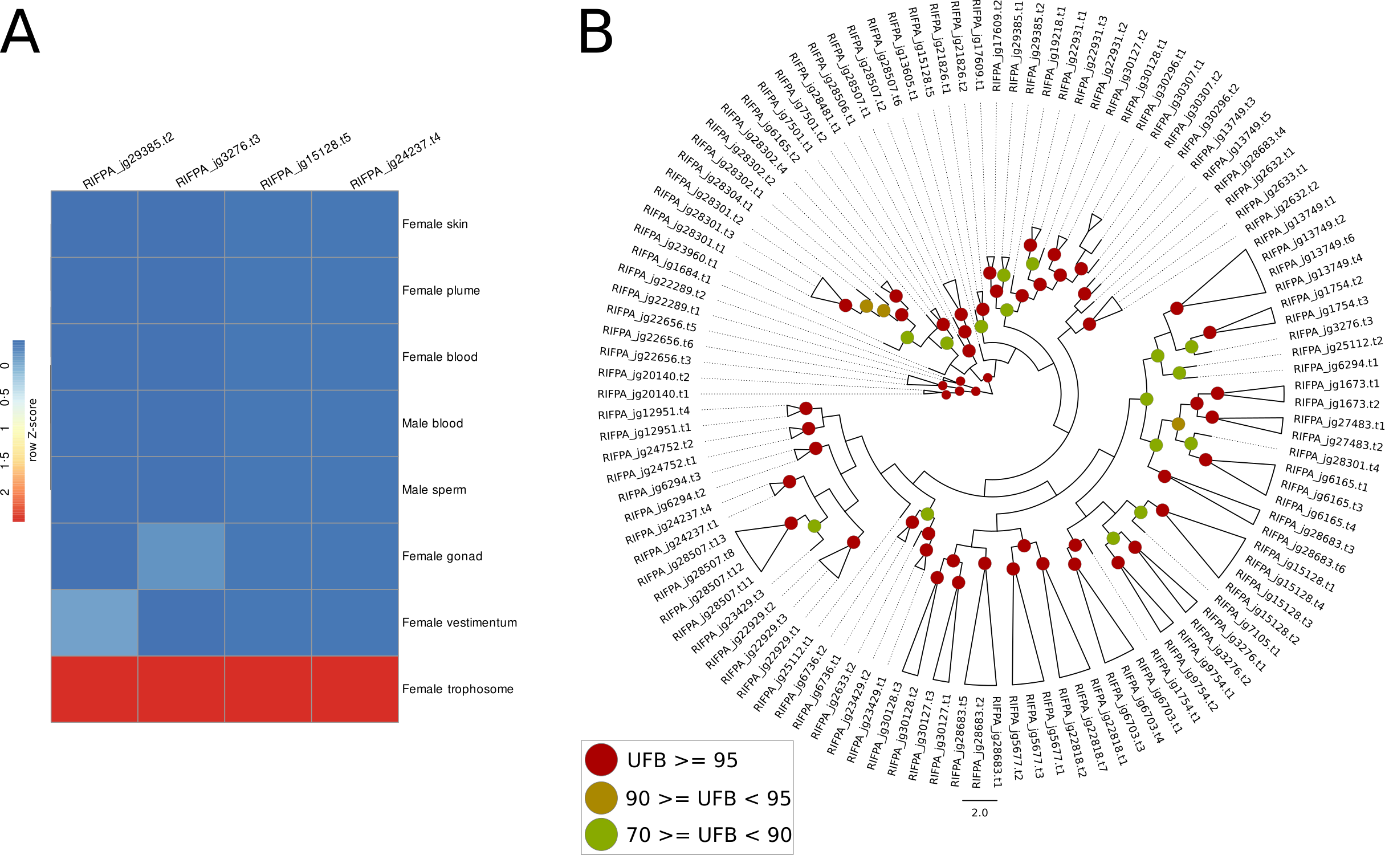
Surprisingly, the male and female blood transcriptomes present distinct transcriptional landscapes, as showed by the few shared GO terms (Additional supplementary figure 4). The shared terms are related to cation/anion transport and cell adhesion, probably reflecting the transfer of metabolites and transport of coagulation factors through the circulatory system, respectively. The female blood TSGs are involved with haem-, oxygen-, and insulin-like binding activities, in agreement with the transport of gaseous substances and hormones in this tissue. Many male blood TSGs, however, point to cell divisions events. Gene expression analyses of key elements involved in the cell cycle showed that several cyclins and cyclin-dependent kinases are expressed in the blood samples, in agreement with the GO enrichment analysis (Supplementary Figure 54). The enrichment of cell cycle genes in the blood transcriptomes might be explained by the sampling of multipotential hematopoietic stem cells during the tissue collection. As the blood samples were obtained after the dissection of the trunk region of the tubeworms, we speculate that hematopoietic cells present either in the blood vessels and/or trophosomal peritoneal membrane were sampled (Southward et al. 2005; Hartenstein 2006; Nakahama et al. 2008).

**Additional supplementary figure 3** – Gene expression and phylogeny of mitochondrial carriers present in the giant tubeworm genome. **A**, expression profile of four selected mitochondrial carrier genes in *Riftia pachyptila*. Colour coding reflects the expression patterns based on row Z-score calculations. B, phylogeny of 45 mitochondrial carriers found in the giant tubeworm genome. The branch support values are represented by the coloured circles in the tree nodes. Red circles represent ultrafast bootstrap values >= 95. Yellow circles represent ultrafast bootstrap values >= 90 and < 95. Green circles represent ultrafast bootstrap values < 90 and >= 70. Ultrafast bootstrap values smaller than 70 are not shown.


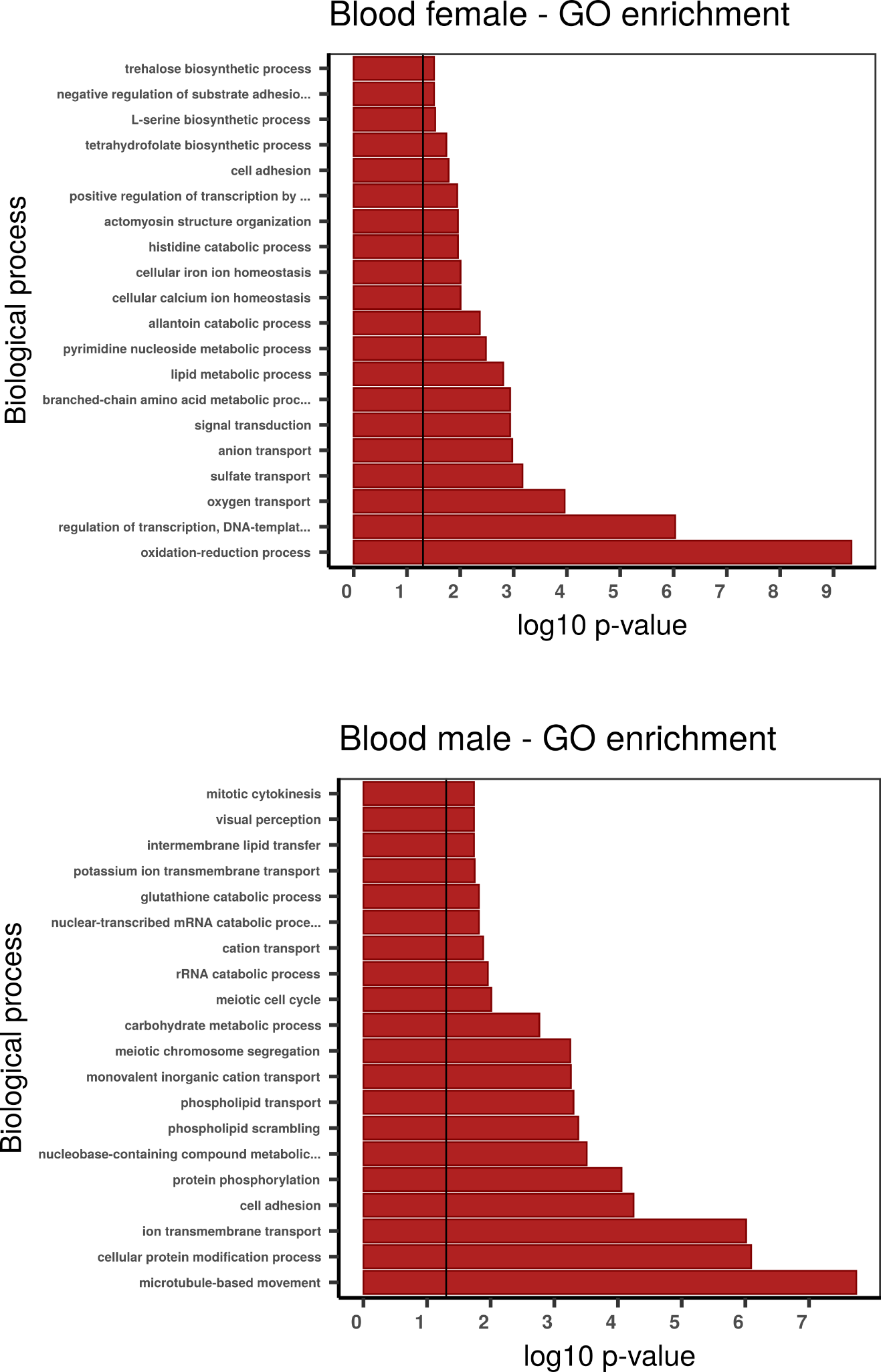


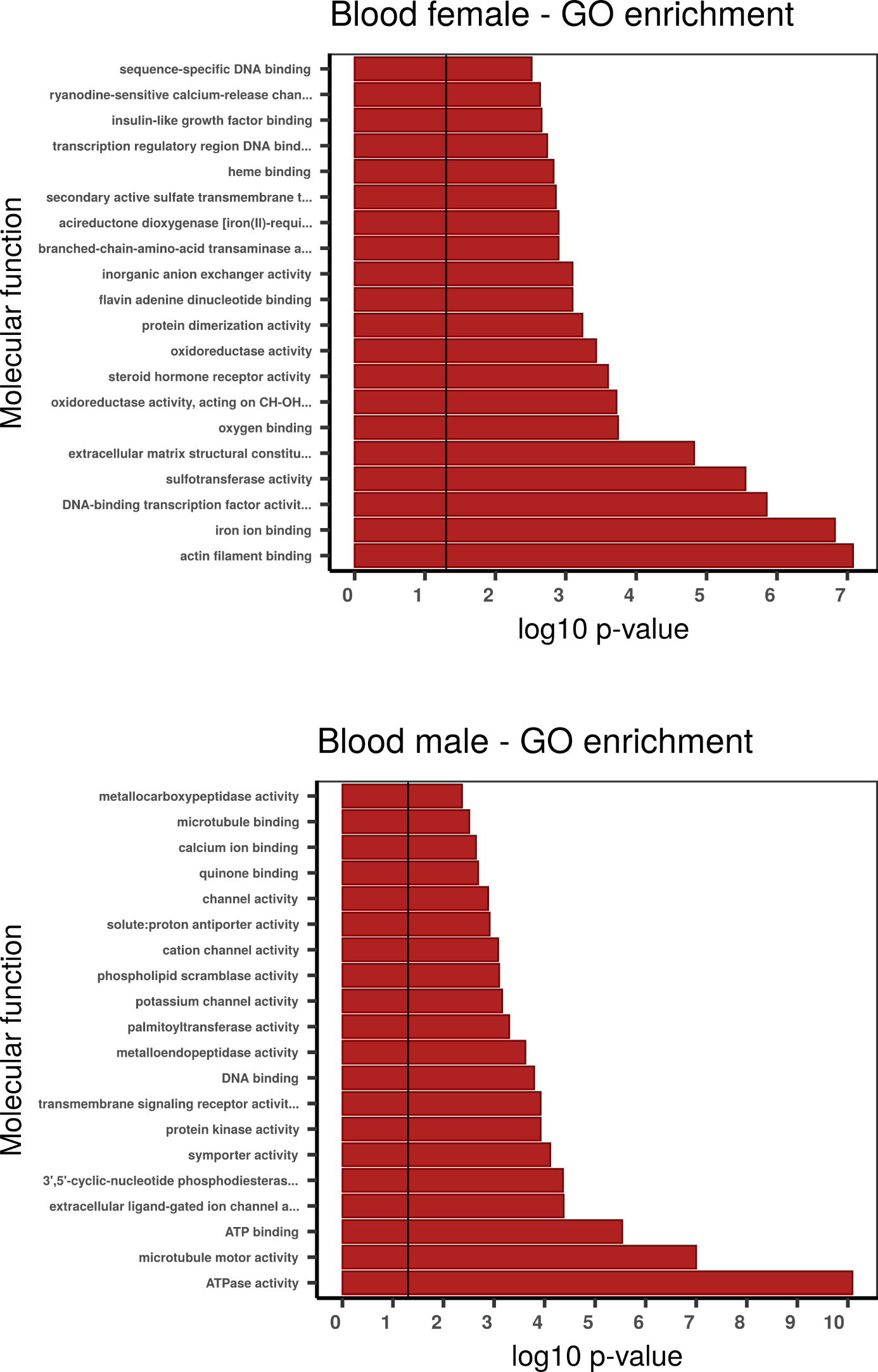


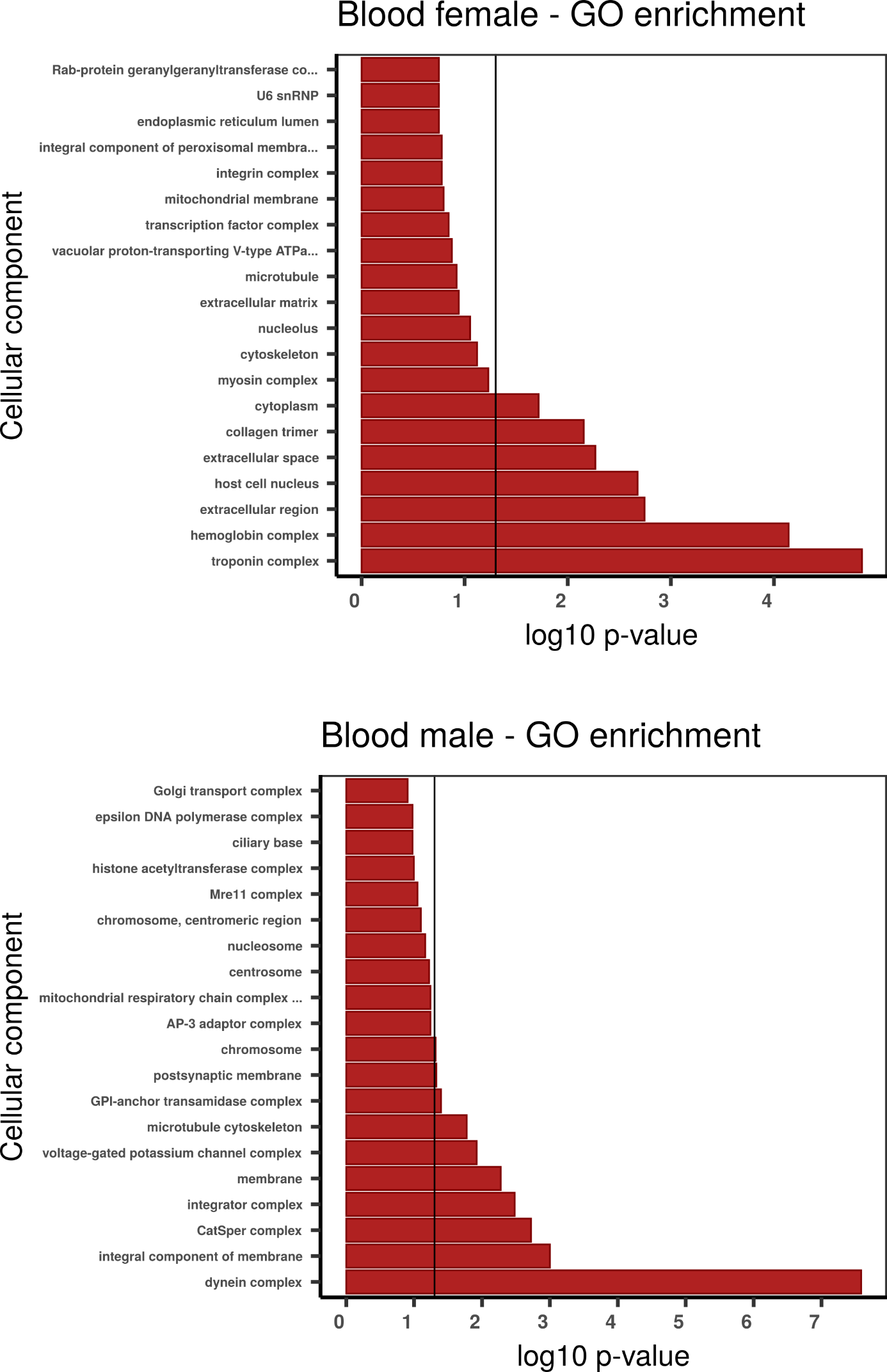


**Additional supplementary figure 4 -** Gene ontology (GO) enrichment analyses for absolutely blood specific TAU genes. The graphs correspond to the three domains of ontologies: biological process (BP), molecular function (MF) and cellular component (CC). The selected genes were analysed for enrichment in specific GO categories using the TopGO program against the background (all coding sequence genes). Y axis corresponds to enriched GO terms found in the respective domains (BP, MF and CC). X axis correspond to the log function of Fisher p-values obtained for each one of the enriched terms. The back line denotes a p-value = 0.05. P-values greater than 1,30 (log 0,05) indicate statistically significant enriched term.

# **Supplementary note 6 | Nitrogen metabolism and excretion**

To investigate the nitrogen metabolism in *Riftia*, we identified and quantified the gene expression of several enzymes related to the purineolytic (AMP deaminase), uricolytic (urease, uricase, xanthine dehydrogenase, OHCU decarboxylase, HIU hydrolase, allantoinase, and allantoicase), taurine/hypotaurine (taurocyamine kinase), purine (phosphoribosyl pyrophosphate amidotransferase, glycinamide ribonucleotide transformylase, phosphoribosylformylglycinamidine synthase, phosphoribosylaminoimidazole carboxylase and phosphoribosylamino-imidazolesuccinocarboxamide synthase, adenylosuccinate lyase, 5-Aminoimidazole-4-carboxamide ribonucleotide formyltransferase, guanine monophosphate synthase, adenylosuccinate synthase, hypoxanthine phosphoribosyltransferase, and adenine phosphoribosyltransferase), pyrimidine (CAD, dihydrooratate dehydrogenase, uridine-5 monophosphate synthase), polyamine pathways (spermine, spermidine), as well as the urea (arginase, argininosuccinate lyase, argininosuccinate synthase, and ornithine carbamoyltransferase) and ammonia cycles (glutamate dehydrogenase, glutamine synthetase) (Supplementary Figures 41-45).

The genes AMP deaminase, urease, OHCU decarboxylase, HIU hydrolase, allantoinase, allantoicase, mitochondrial taurocyamine kinase, and the four investigated enzymes in the urea cycle are present as single copy in the giant tubeworm genome, whereas the genes xanthine dehydrogenase and the cytoplasmatic taurocyamine kinase contain four and five distinct copies each, respectively. Three xanthine dehydrogenases/oxidases paralogs (RIFPA_jg12112, RIFPA_jg12113 and RIFPA_jg12115) and four cytoplasmatic taurocyamine kinase genes (RIFPA_jg16631, RIFPA_jg16632, RIFPA_jg16633, RIFPA_jg16634) are located in chromosomal clusters in *Riftia* (Figure 5A). All orthologs of the aforementioned genes were also identified in the cold seep tubeworm. The genes allantoinase, urease and the cytoplasmatic taurocyamine kinase contain one additional copy in *Lamellibrachia* compared to *Riftia,* totalling two (LAMLU_FUN_033197-T1, LAMLU_FUN_020286-T1), two (LAMLU_FUN_032122-T1, LAMLU_FUN_032120-T1) and six paralogs (Supplementary Figure 43), respectively.

Overall, the genes involved in the urea cycle and uricolytic pathway are ubiquitously expressed in all tubeworm tissues. Particularly in the trophosome, the urea cycle enzymes argininosuccinate synthase/lyase, arginase, ornithine carbomoyltransferase, and key components of the uricolytic pathway (urease, 5-hydroxyisourate hydrolase, uricase, allantoicase, allantoinase, OHCU decarboxylase, xanthine dehydrogenase) are highly/moderately expressed (Figure 41). These results agree with biochemical analyses which show an elevated concentration of uric acid, urea, and ammonia in the trophosome in comparison to other symbiont-free tissues (e.g., plume, blood, body wall) (Cian et al. 2000), and with a more recent metaproteomic and comparative transcriptomic study (Hinzke et al. 2019). These results point to different metabolic processes concerning the nitrogen metabolism in the trophosome and their importance in the host biology and/or endosymbiotic association.

Phosphagen kinases (PKs) constitute an evolutionary conserved family of phosphoryl transfer enzymes with important roles in energy homeostasis (Conejo et al., 2008; Ellington, 2001). PKs catalyse the reversible transfer of gamma phosphory group of ATP to guanidino compounds, such as taurocyamine and arginine (Uda et al. 2005; Uda et al. 2006; Suzuki et al. 2009). Hitherto in *Riftia*, one cytoplasmatic and one mitochondrial phosphotaurocyamine kinase have been described as the major phosphagen kinase system of the energy metabolism (Uda et al., 2005). We identified four additional copies of taurocyamine kinase in the *Riftia* genome. They are highly expressed in all tubeworm tissues, with the exception of the trophosome and skin (body wall) (Supplementary Figure 44). The cold seep tubeworm genome harbours six copies (five cytosolic and one mitochondrial) of taurocyamine kinases, whereas *Capitella* contains one and two copies of the mitochondrial and cytosolic types, respectively. We did not identify any taurocyamine kinase gene in the leech *Helobdella*. The finding of additional taurocyamine kinase genes in *Riftia* could clarify the regulation of glycogenolysis and intracellular energy transport, which are important factors that account for the tubeworm large size and rapid growth.

In a series of biochemical and thorough studies Minic et al. demonstrated that *Riftia* lacked the initial enzymes involved in the pyrimidine biosynthetic pathway relying on the endosymbiont for the *de novo* synthesis of pyrimidines and polyamine production (Minic et al. 2001; Minic et al. 2002; Minic and Hervé 2003; Minic and Hervé 2004). These results were challenged recently by Hinzke et al. (2019) which identified the trifunctional protein CAD (carbamoyl-phosphate synthetase 2, aspartate transcarbamylase, and dihydroorotase) and key genes in the polyamine synthesis, i.e., spermidine and spermine synthases, in the host metatranscriptome. We, through similarity searches and phylogenetic inferences, identified the CAD and the polyamine-related genes in the giant tubeworm genome, confirming that *Riftia* is indeed less dependent on the symbiont regarding the nitrogen metabolism. Additionally, in agreement with Hinzke et al. (2019), that did not detect the expression of CAD on a protein level, we observed that the CAD is lowly expressed in the eight analysed tissues (Supplementary Table 8 – Supplementary Figures 42 and 44), which probably is the explanation for Minic et al. (2002) results. In fact, all the genes involved in the pyrimidine biosynthesis, with the exception of uridine 5 monophosphate synthase, present a low expression level in the giant tubeworm tissues (Supplementary Table 8). Spermine and spermidine have been implicated in host-symbiont interactions in *Riftia*, due the restricted distribution of the enzymes catalysing these polyamines solely in trophosome metaproteomes (Hinzke et al. 2019). We, however, observed a high expression of these enzymes in all *Riftia* tissues (Supplementary Table 8; Supplementary Figures 42 and 44), corroborating their important roles in the host overall homeostasis (e.g., protein and nucleic acid synthesis, protection from oxidative damage, cell proliferation, apoptosis, and differentiation (Pegg, 2016)). Both vestimentiferans, *Riftia* and *Lamellibrachia*, contain one single copy of the CAD and spermidine synthase gene, whereas a duplication of the gene spermine synthase is observed in tubeworms (Supplementary Table 8; Supplementary Figures 42 and 44). These results show that the *de novo* pyrimidine biosynthesis and polyamine production is also maintained in the cold seep tubeworm. The key enzymes involved in the *de novo* purine synthesis are present in the giant tubeworm genome (Supplementary Table 8; Supplementary Figure 42).

*Riftia* requires a high demand of nitrogen because of the high biomass and growth rates (Lutz et al. 1994). These requirements are usually achieved by the level of environmental nitrate and ammonia in the environment (Johnson et al. 1988; Lilley et al. 1993), or by the active digestion of the symbionts (Girguis et al. 2000). We identified and quantified the gene expression of important genes containing the glutamine synthetase and glutamate dehydrogenase protein domains in the giant tubeworm genome and closely related annelid specifies and lophotrochozoans (Supplementary Table 8; Supplementary Figure 45; Figure 5). These enzymes are required to incorporate ammonium into organic matter. Glutamine synthetase and glutamate dehydrogenase show an elevated expression in eight tubeworm tissues herein investigated, especially in the plume and gonad tissues. The relative contribution of ammonium derived from the diffusion into the host by the endosymbionts (i.e., reduction of nitrate into nitrate and then ammonia by Endoriftia (Girguis et al. 2000)), or from the degradation of symbionts by lysosomal activity is still elusive. We identified in annelids the presence of genes belonging to two distinct clades of glutamine synthetase group I: the lengsins and a group so far containing only prokaryotic sequences. (Figure 5C; Supplementary Figure 43). Lengsins constitute a group of glutamine synthetase genes which were co-opted to non-enzymatic roles in the vertebrate eye lens (Wyatt et al. 2006). The giant tubeworm and *Lamellibrachia* genomes contain seven and 13 copies of lengsins, respectively. Surprisingly, only annelid sequences and poriferan sequences clustered together with hitherto prokaryotic glutamine synthetase I sequences in the phylogenetic tree (Figure 5). The glutamine synthetase group II, traditionally found in eukaryotes, includes two paralog copies of *Riftia* and *Lamellibrachia* genes. Vestimentiferans possess the highest number of glutamine synthethase-containing genes in all 18 investigated lophotrochozoans, highlighting the importance of the ammonia assimilation in the tubeworms. Lengsins are highly expressed in the *Riftia* trophosome.


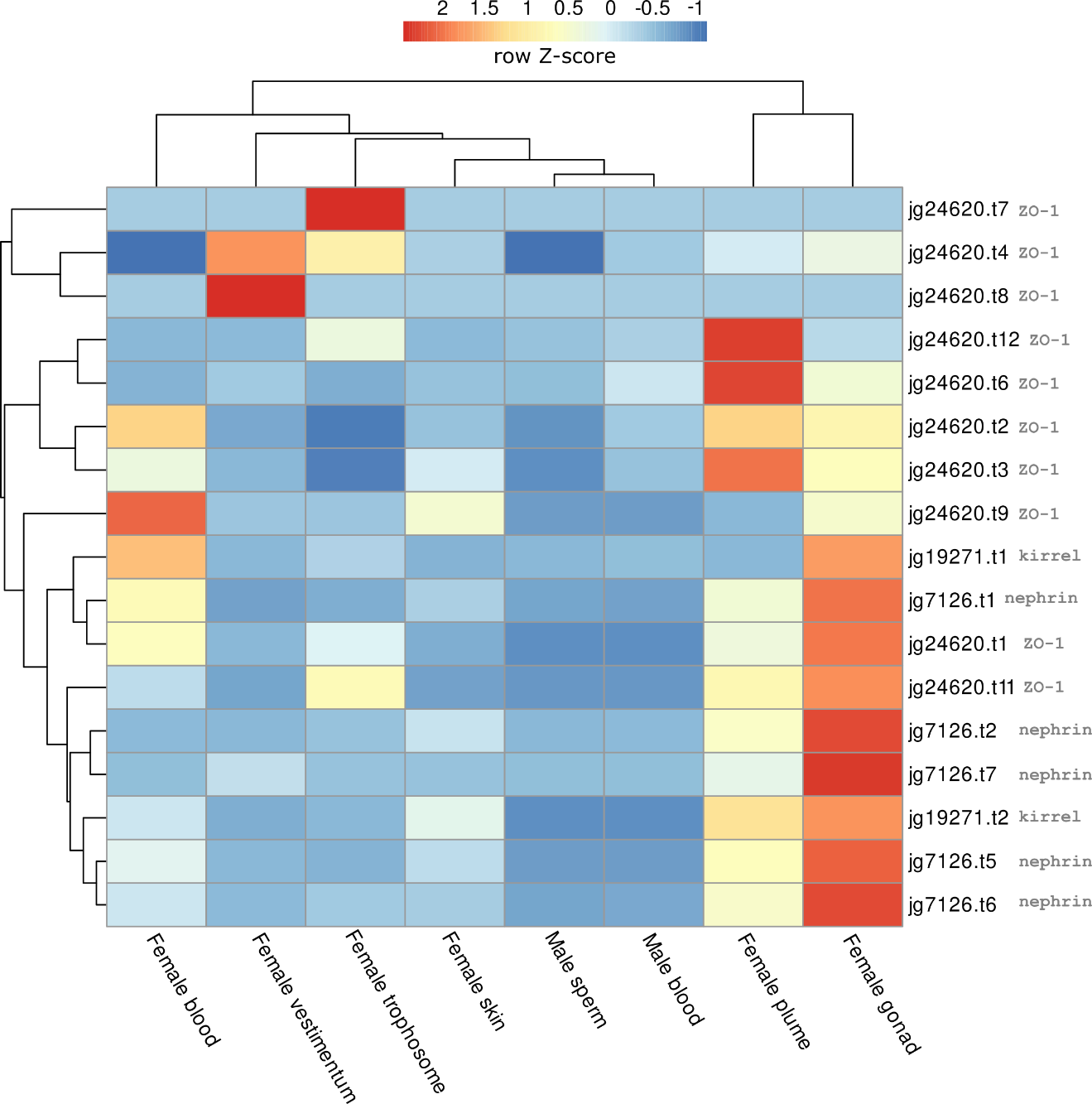
In polychaetes, the site of the ammonia excretion and/or the mechanisms in which this nitrogenous waste is secreted are still poorly understood, with few studies publicly available (Smith et al. 1987; Smart and Von Dassow 2009; Thiel et al. 2016; Rimskaya-Korsakova et al. 2018; Weihrauch and Allen 2018; Rimskaya-Korsakova et al. 2020). A recent study investigated the expression important structural genes (*nephrin*, *kirrel* and *zo1*) responsible for the formation of filtering sites in the excretory organs of lophotrochozoans, including the polychaete *Owenia fusiformis,* ecdysozoans and deuterostomes taxa (Gąsiorowski et al. 2021). We screened the genome of the giant tubeworm for the presence of these three structural genes and quantified their gene expression in the eight tissue-specific transcriptomes (Additional supplementary figure 5).

**Additional supplementary figure 5.** Expression profile of three important structural genes (*nephrin, kirrel* and *ZO-1*) related to excretory organs in *Riftia pachyptila*. Colour coding reflects the expression patterns based on row Z-score calculations. Desaturases are ubiquitously expressed in all tubeworm tissues.

We identified in *Riftia* and *Capitella* a unique copy of each one of the structural genes *nephrin* (RIFPA_jg7126, CAPTE_P184830), *kirrel* (RIFPA_jg19271, CAPTE_P184834), and *ZO-1* (RIFPA_jg2460, CAPTE_P221876). Duplications of *kirrel* (LAMLU_FUN_007962-T1, LAMLU_FUN_024093-T1) and *ZO-1* (LAMLU_FUN_029123-T1, LAMLU_FUN_033606-T1) were identified in the *Lamellibrachia,* and a possible secondary loss of *kirrel* and *nephrin* is present in the *Helobdella* genome. *Nephrin* and *ZO-1* are present as single copies in the *Lamellibrachia* and *Helobdella* genomes, respectively (LAMLU_FUN_008794-T1, HELRO_176655).

All three genes are highly expressed mainly in the female gonad, plume, and blood tissues of *Riftia*, in agreement previous studies that show that these proteins interact together in the filtering cells of ultrafiltration excretory organs (Gerke et al. 2003; Huber et al. 2003; Liu et al. 2003; Gąsiorowski et al. 2021). The gene expression results, especially in the plume region, are surprising, since the putative site of ultrafiltration in vestimentiferans is located in the large protonephridial excretory tree part of the vestimental region (Schulze 2001; Bright and Lallier 2010; Rimskaya-Korsakova et al. 2018).

The storage of nitrogenous waste in the trophosome may be explained, at least in part, by the unusual excretory organ found in vestimentiferans. Recently, a microanatomical study identified protonephridia in *Ridgeia pisceseae* (Schulze 2001; Rimskaya-Korsakova et al. 2018). In fact, it is highly unusual that these giant tubeworms do not develop metanephridia, known to be the dominant form of excretory organ in large polychaetes with a well-developed blood vascular system (Ruppert and Smith 1988; Bartolomaeus and Quast 2005). While in metanephridium the podocytes facilitate filtration from the blood to the coelomic fluid, in protonephridia only coelomic fluid is filtered (Ruppert and Smith 1988).

# **Supplementary note 7 | Cell proliferation, innate immune system, apoptosis, and autophagy**

Immunohistochemical and ultrastructural cell cycle investigations have shown that in vestimentiferans, including *Riftia*, cell proliferation activities are higher than in any other characterised invertebrate (Pflugfelder et al. 2009). We investigated key regulatory enzymes present in the cell cycle (G1, S, G2 and M) with special focus on cyclin-dependent kinases (CDKs), *smad4* and *smad2* (Supplementary Figure 46). CDKs are serine/threonine kinases which depend on their regulatory subunits, cyclins, to perform catalytic activities (Lim and Kaldis 2013; Malumbres 2014). *Smad4* and *smad2* serve as the central mediator of TGF- β signalling pathway and are involved in a wide range of cellular processes, including uncontrolled proliferation leading to cancer initiation (Nakao et al. 1997; Zhao et al. 2018). Additionally, *smad4* has been shown to undergo an independent expansion and present signs of positive selection in the cold seep tubeworm *Lamellibrachia luymesi* (Li et al. 2019), which may play important roles controlling the high proliferation rates in vestimentiferans.

We identified in the *Riftia* genome through similarity searches and phylogenetic inferences all the expected CDKs involved in the G1, S, G2 and M phases of the cell cycle and the *smad4* gene. All genes are present as single copies in the giant tubeworm genome. KEGG pathway analysis reconfirmed our findings. We identified three copies of *smad4* in *Lamellibrachia*, two in *Helobdella robusta* and one in *Capitella*, contradicting Li et al. (2019) results regarding the number of homologues in the cold-seep tubeworm and the leech. Gene expression analysis showed that the cell cycle genes are ubiquitously expressed across the studied tubeworm tissues, with non-symbiotic tissues (i.e., female gonad, blood, plume and vestimentum) showing the highest expression levels (Supplementary Figure 46). Cyclin A is highly expressed in the female gonad and blood, indicative of DNA synthesis, and G2 phase, whereas cyclin B2 is highly expressed, in vestimentum and plume, indicative of mitosis. In the trophosome, the high expression of cyclins A and B2 marking the S, G2 and M phase of mitosis have been identified, despite the overall low expression of CDKs in this tissue. These results indicate active DNA synthesis and proliferation events in this tissue, which were also detected with immunocytochemistry in the unipotent stem cells in the central and the semi-differentiated cells in the median zone of the trophosome lobule (Pflugfelder et al. 2009). The transcription factors *smad4* and *smad2* seem to be down regulated in the trophosome, an indicative of high cell proliferation activity (Samanta and Datta 2012).

Innate immunity activation is facilitated by pattern recognition receptors such as the Toll-like receptors (TLRs) and peptidoglycan receptor proteins (PRPs) to recognize microbe associated molecular patterns (Nyholm and Graf 2012). The TLRs constitute an evolutionary conserved transmembrane family of proteins involved in innate immune responses in metazoans (Janssens and Beyaert 2003; Kawai and Akira 2006; Song et al. 2012; Kawasaki and Kawai 2014; Liu et al. 2020) (Supplementary Figures 47-57). Briefly, when TLRs recognise pathogen-associated molecular patterns (PAMPs), the myeloid differentiation primary response protein 88 (*MyD88* gene) recruits various adaptor molecules (e.g., IRAK, TAB1/TAK1 complexes) that trigger the activation of the *AP1* and *NF-κB* transcription factors, that in turn, control the outcome of the innate immune responses. In lophotrochozoans, lineage-specific expansions (phoronids, bivalves) and gene loss (rotifers, planarians, and blood flukes) of TLR genes have been reported (Sun et al. 2017; Luo et al. 2018). We identified 17 distinct TLR genes in the giant tubeworm genome, with orthologous gene counterparts in the close relative *Lamellibrachia luymesi*, which contains 28 TLRs genes (Supplementary Figures 47-49). We could not clearly assign the tubeworm TLRs into the different vertebrate classes, probably due the lineage-specific duplications of these proteins in vertebrates and vestimentiferans. Additionally, *Riftia* contains all expected core proteins involved in the Toll-like receptor/MyD88 pathway, and gene expression analyses showed that TLRs and other components involved in the innate immune system are mainly expressed in the female gonad, plume, vestimentum and skin tissues. This suggests that TLR does not play a decisive role in the trophosome.

The cellular response upon innate immune system recognition and tissue homeostasis is driven either by apoptosis or autophagy. Alternatively, apoptosis (Supplementary Figures 50-57) and autophagy (Supplementary Figure 58) play important roles in host-symbiont interactions acting in the regulation of the endosymbiont populations in the deep-sea bivalve *Bathymodiolus platifrons* and the cereal weevil *Sitophilus*, respectively (Vigneron et al. 2014; Sun et al. 2017). Firstly, we screened the giant tubeworm genome for known protein domains involved with apoptotic process: caspases (Supplementary Figures 50-51), B-cell lymphoma 2 (Bcl-2; Supplementary Figure 52), the tumour necrosis factor ligands (TNF; Supplementary Figure 53) and receptors (TNFR; Supplementary Figure 54), and inhibitor of apoptosis proteins (IAP; Supplementary Figures 55-57). Caspases are intracellular cysteine proteases responsible for the controlled degradation of cells during apoptosis (Julien and Wells 2017). They are activated by extracellular ligands, such as TNF death receptor superfamily, and are involved in the extrinsic apoptosis pathway (Park et al. 2007). We identified in the *Riftia* genome 11 caspases and one para-caspase, making the giant tubeworm complement the smallest amongst annelids and molluscs. The cold-seep tubeworm contains 15 caspases and two para-caspases. Caspase expression in *Riftia* is stronger in the plume, and female gonad than in the skin and vestimentum, and in the trophosome. The Bcl-2 proteins, which are involved in the intrinsic apoptosis pathway (Czabotar et al. 2014) are highly/moderately expressed in the plume, female gonad, but not in the trophosome. *Riftia* contains a Bcl-2 complement of five genes.A total of four and six TNFRs/TNF ligands were found in the tubeworm genome, with overlapping gene expression in the plume and female gonadal tissues. In the trophosome, however, we found highly expressed a TNF ligand, but not the required corresponding TNFRs. TNFs (as well as extrinsic factors) activate caspases, intracellular cysteine proteases responsible for the controlled degradation of cells during apoptosis (Julien and Wells 2017). The IAPs, which negatively regulates caspases and cell death (Silke and Meier 2013), has been shown to be expanded in the deep-sea/seep mussel bivalves *Bathymodiolus* (130 genes) and *Modioulus* (95 genes), respectively (Sun et al. 2017). *Riftia* and *Lamellibrachia,* however, contains a relatively simple anti-apoptotic system with only 19 and 36 genes, respectively. IAP gene expression was upregulated in all giant tubeworm tissues, but mainly highly expressed in the plume and female gonad tissues. Only a few IPA genes were highly expressed in the vestimentum, skin and trophosome tissues.

Overall, tissues exposed to the environment exhibited high expression of apoptosis-related genes, while the skin and the vestimentum (protected by the tube) and the internally located trophosome appears much less involved in apoptosis. Despite a great variability in the number upregulated genes involved in the apoptotic events, all studied tubeworm tissues showed high expression of IAPs. This may be due to the fact that all samples consisted of multiple different cell types some of which might be upregulated in apoptosis, others inhibited (e.g., skin is composed of the epidermis, gland, nervous, and muscle cells). Even in the trophosome, despite the presence of blood vessels and aposymbiotic sheet cells, only the peripheral region of the tissue’s lobules was found to die during terminal differentiation through apoptotic processes, while the more central region was not involved in massive apoptosis (Bright and Sorgo 2003; Pflugfelder et al. 2009). These differences in the morphological and ultrastructural organisation of the trophosome is certainly followed by variation in gene expression levels of apoptotic genes.

While phagocytes are described to clear the tissue from apoptotic cell remains (deCathelineau and Henson 2003), the terminal step of autophagy is lysosomal degradation of either sequestered cytosolic material present in autolysosomes (membrane-bound vesicles fused with lysosomes), or the direct uptake of components by the lysosomes, as found in macro- and microautophagic processes (Glick et al. 2010; Hansen et al. 2018), respectively. An additional and more complex type of autophagy, named chaperone-mediated autophagy, involves the degradation of targeted proteins through their translocation across the lysosomal membrane with the aid of chaperone proteins (Glick et al. 2010; Hansen et al. 2018). To date, the complete autophagic pathway has not been identified in any annelid genome and remains unknown. Furthermore, this study and others show that *Riftia* (Hinzke et al. 2019), and a closely related vestimentiferan (Li et al. 2019), present a high expression of lysosomal hydrolase genes in the trophosome, implicating that the tubeworms digest their endosymbionts for nutrition. To elucidate the autophagy pathway in *Riftia*, we identified through similarity searches and phylogenetic analyses the core genes (Atg proteins – autophagy related proteins (Klionsky 2012)) involved in macroautophagy process (hereafter termed autophagy), since this is the predominant and most studied form of autophagy (Mizushima and Komatsu 2011) (Supplementary Figure 58).

This type of programmed cell death starts with the key upstream regulators present in the mTORC1 complex, which phosphorylates the Atg1/ULK1 complex initiating the autophagy (Wong et al. 2013). The membrane nucleation, phagophore formation and expansion are mediated by Atg proteins present in the PI3K complex I, ATG12 and LC3/GABARAP conjugation systems (Glick et al., 2010). *Atg2*, and *atg9* also participate in the autophagosome formation by transferring phospholipids and expanding the newly synthesized phagophore (Velikkakath et al. 2012; Zhou et al. 2017; Gómez-Sánchez et al. 2018; Kotani et al. 2018; Osawa et al. 2019). Lastly, the autophagosome maturation is achieved with the fusion of the autophagosome with lysosomes to form the autolysosome. *Riftia* contains all the core elements commonly found in yeast, human and bivalve autophagy pathways, demonstrating the high conservation of this mechanism in distantly related eukaryotic taxa (Ohsumi 2014; Picot et al. 2020).

The gene family Atg2 contains two paralogs in the giant tubeworm genome (*atg2a* and *atg2b*) and the same scenario is found mammals (Velikkakath et al. 2012; Tamura et al. 2017). Two paralogous sequences of Atg4 (*atg4a, atg4cd*) are present in *Riftia.* The cold-seep tubeworm contains three paralogs, whereas the worm *Caenorhabditis elegans* and humans present two and four copies, respectively. These results indicate different patterns of gene duplication in different closely related species, as well as in protostome, and deuterostome lineages. Atg8 gene family contains four members in *Riftia*, *Lamellibrachia*, *Capitella* and *Helobdella* (*gabarap*, *gabarapl2*, *map1lc3ab*, *map1lc3c*), indicating a conservation of gene numbers of this autophagic gene family in annelids. The Atg8 family has undergone different gene gain and loss events throughout the metazoan tree, with noticeable expansions of two (GABARAP and LC3/MAP1LC3) out of the three subfamilies in vertebrates (Shpilka et al. 2011). The Gate16/GABARAPL2 subfamily is represented by a single copy gene in most of the animals, except for sponges (*Amphimedon queenslandica*) and echinoderms (*Stronglycentrotus purpuratus*) which contains two paralogous genes (Shpilka et al. 2011). All the remaining autophagy-related genes are present as single copy in the vestimentiferans *Riftia* and *Lamellibrachia*. Gene expression analyses showed that Atg genes present different levels of expression in the eight tubeworm tissues, with the female plume, gonad and blood samples harbouring most of the highly expressed genes. The trophosome, however, only shows upregulation of a few genes only, indicating that autophagy is not a widespread mechanism of control death in this tissue.

Lysozymes have an important dual role in invertebrate-endosymbiont interactions, not only controlling the intracellular symbiont population, but also accessing nutritional resources during the process (Nakabachi et al. 2005; Nishikori et al. 2009; Xue et al. 2010; Futahashi et al. 2013; Kim et al. 2014; Detree et al. 2016). As previously mentioned, lysosomal degradation is involved in distinct controlled cell death mechanisms involving recognition and elimination of self and nonself (i.e., apoptosis and autophagy). However, other different pathways, such as the endocytic, employ the lysosomal system for the digestion of macromolecules (Bröker et al. 2004; Guicciardi et al. 2004; Mariño et al. 2014; Hu et al. 2015; de Castro et al. 2016; Man and Kanneganti 2016; Wong et al. 2017). To identify important early, late and recycling endosomal genes in the giant tubeworm genome, we performed multiple sequence similarity searches using metazoan Rab GTPases (Stenmark 2009), and other key genes (transferrin, EEA1, M6PR) (Brown et al. 1986; Mu et al. 1995; Mayle et al. 2012) as queries, followed by phylogenetic inferences. We found that key genes involved in the different stages of endosome maturation are present as single copy in the giant and cold-seep tubeworm genomes (Supplementary Figure 37). The endosomal genes are expressed in all *Riftia* tissues, with the plume and the trophosome harbouring distinct highly expressed early and late endosomal genes. This suggest that endosomal degradation is prevalent in the trophosome but also in aposymbiotic hosts tissue, most strongly expressed in the plume and the female gonad.

Taking into consideration all the aforementioned results, it is hard to definite state, solely based on gene expression analyses and orthology inferences, the exact scenario behind the endosymbiont digestion and trophosome tissue homeostasis in *Riftia*. To make matters worse, autophagic and apoptotic events may occur simultaneously within the same cell and the trophosome contains distinct proliferative and degenerative regions (Bright and Sorgo 2003; Pflugfelder et al. 2009; Mariño et al. 2014). Nonetheless, as previously mentioned, we did not identify an abundant expression of autophagic, apoptotic, and immune-related genes in the trophosome, but only a high expression of lysosomal hydrolases, and moderate/highly expression of endosomal genes in this tissue. These results point that endosymbiont digestion in *Riftia* is probably independent of innate immune system recognition and programmed cell death events, in a process similar to endosome maturation, as suggested by Hinzke et al. (2019). We hypothesized a mechanism that involves the fusion of the double membrane symbiosomes with lysosomes (coined here as symbio-lysosome structure) followed by the direct digestion of the endosymbionts.


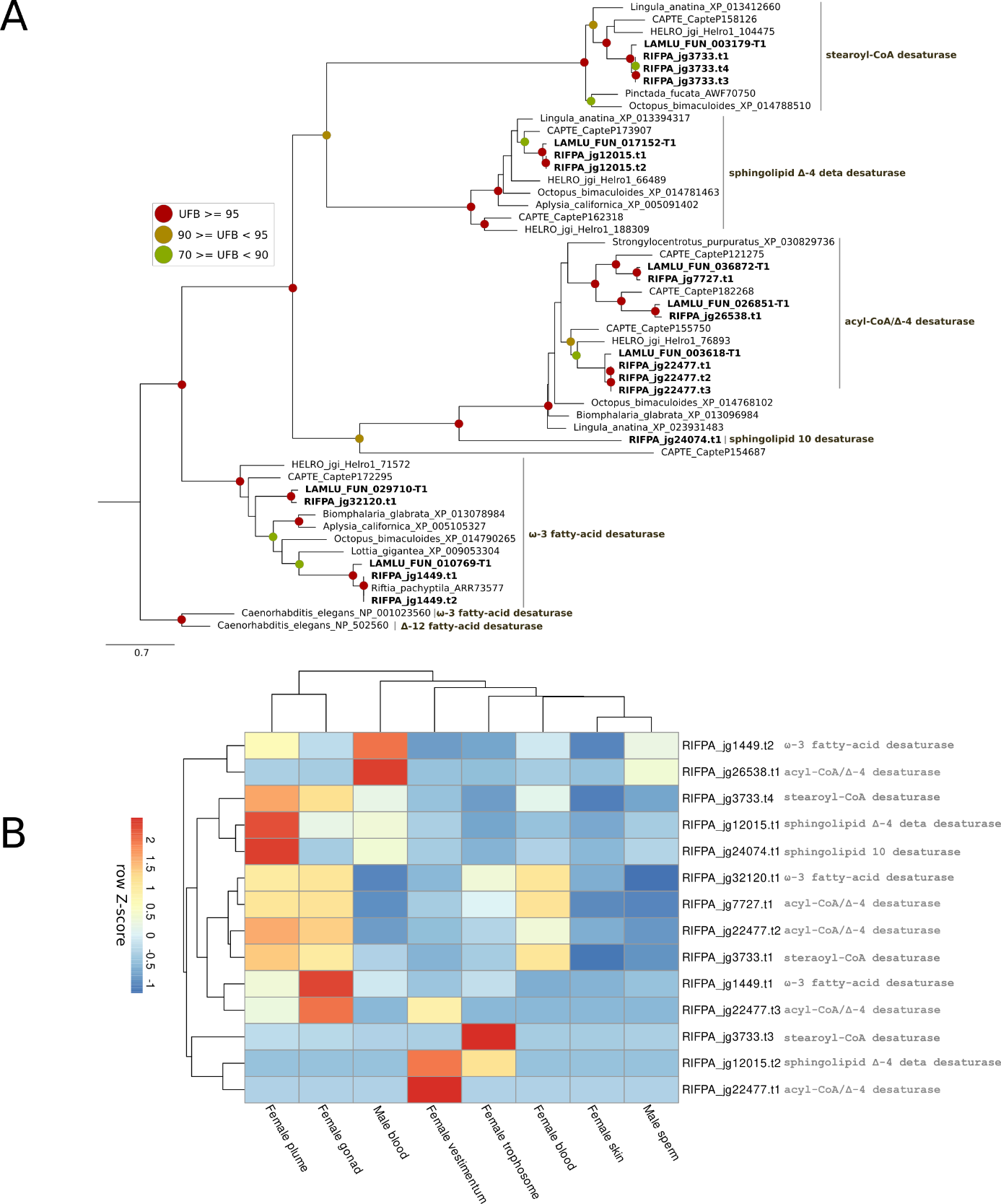


**Additional supplementary figure 6** – Phylogeny and gene expression of fatty acid desaturases in the giant tubeworm genome. **A**, Phylogeny of eight desaturases identified in the the giant tubeworm genome. The branch support values are represented by the coloured circles in the tree nodes. Red circles represent ultrafast bootstrap values >= 95. Yellow circles represent ultrafast bootstrap values >= 90 and < 95. Green circles represent ultrafast bootstrap values < 90 and >= 70. Ultrafast bootstrap values smaller than 70 are not shown. Accession numbers for NCBI database are displayed after the species names. *Capitella, Helobdella* and *Lamellibrachia* gene identification are derived from the publicly available annotated genomes. **B**, expression profile of fatty acid desaturases in the genome of *Riftia pachyptila*. Colour coding reflects the expression patterns based on row Z-score calculations. Desaturases are ubiquitously expressed in all tubeworm tissues.

The high expression of programmed cell-death, immune system and cell cycle genes in the plume and female gonad of the giant tubeworm raises interesting questions about the proposed model of symbiosis between the deep-sea *Bathymodiolus platifrons* and its methane oxidising endosymbiont. Sun et al. (2017) showed high expression levels of gene families related to immune recognition, endocytosis, and caspase-mediated apoptosis in the gills of *B. platifrons*, suggesting evolutionary adaptations of the deep-sea mussel towards its endosymbionts. Our analysis, however, did not identify an abundant expression of proliferation, immune system, and controlled cell death (i.e., apoptosis and autophagy) gene markers in the trophosome. Two alternative hypotheses can be drawn from these results. First, the similar transcriptional landscapes of the gills and the plume, in *B. platifrons* and *Riftia*, respectively, indicate similar defence mechanisms against pathogens and homeostasis of the respiratory organs, rather than the control of the endosymbionts in the bivalve. Second, these two lophotrochozoans display distinct mechanisms to control, digest and maintain the symbiont populations within their respective endosymbiont harbouring tissues. We argue, that as the trophosome is internally located in the trunk region of the giant tubeworm not contacting the deep-sea vent fluids, it represents a more “sterile” and controlled environment to understand the molecular dynamics behind the host-endosymbiont mutualism. Cell atlases of vestimentiferan trophosome tissues obtained from single cell transcriptomic analyses (Aldridge and Teichmann 2020) are required to completely unravel the biology of this tissue.

The cold-seep tubeworms *Lamellibrachia luymesi*, *Seepiophila jonesi*, and *Escarpia laminate* present an extraordinarily long-life span (Lutz et al. 1994; Fisher et al. 1997; Bergquist et al. 2000; Cordes et al. 2007; McClain et al. 2012) rendering them, together with the bivalve *Arctica islandica* (Ridgway and Richardson 2011), the most-long lived marine invertebrates described to date. The evolution of longevity in these animals might be favoured by the stable and long-lasting hydrocarbon seep environment (Sibuet and Olu 1998), absence of lethal predation (Bergquist et al. 2003), and physiological adaptations (Pflugfelder et al. 2009). Differences in the molecular coordination between cell proliferation/turnover and death/growth have been hypothesized to account for the alternative life strategies of the fast-growing short-lived *Riftia* and the slow-growing long-living *Lamellibrachia* (Pflugfelder et al. 2009). Here, in addition to the full characterisation of the cell-cycle and death pathways described in the previous paragraphs, we screened the genome of the giant tubeworm and *Lamellibrachia* for the presence of other lifespan-regulating genes (i.e., insulin- and TOR-signalling-pathway genes, and sirtuins (Sir)) (Uno and Nishida 2016).

Both tubeworm genomes contain a single copy of insulin ligand/receptor genes (ligand: RIFPA_jg14616 and LAMLU_FUN_019591-T1; receptor RIFPA_jg13005 and LAMLU_FUN_010854-T1), and *Sir-2* (RIFPA_jg26579 and LAMLU_FUN_009556-T1), a member of an evolutionary conserved protein family involved in cell survival, senescence, and metabolism (Dang 2014; Ye et al. 2016). Additional paralogs belonging to the classes I, II, III and IV (*Sir1-, 3-, 4-, 5-, 6-* and *-7*) were also identified in *Riftia* and *Lamellibrachia*, attesting their conservation in vestimentiferans. The *forkhead-A* (*foxa*) transcription factor, a master regulator of the TOR signalling (Sheaffer et al. 2008), is found as a tandem duplication in the tubeworm genomes (RIFPA_jg12092 and RIFPA_jg12093 – LAMLU_FUN_029846-T1 and LAMLU_FUN_029847-T1).

Furthermore, it has been proposed that the extended lifespan of the seep-dwelling vestimentiferans might be associated with the genomic expansion of superoxide dismutase genes, specifically *SOD1* and *SOD2*, in Lamellibrachia compared to other lophotrochozoans (Li et al. 2019). This stems from the fact that knockouts/inactivation and gene silencing of *SOD1*- and *-2* in mice and Drosophila, respectively, have shortened the lifespan in these animals (Li et al. 1995; Sentman et al. 2006; Oka et al. 2015). Homology searches of SOD genes in the giant tubeworm genome could not, however, corroborate Li’s et al. (2019) hypothesis since the SOD complement in both *Riftia* and *Lamellibrachia* is similar (Supplementary Figure 38). Additionally, our conservative PFAM_scan analysis showed that the number of SOD-domain containing proteins (Sod_Cu: PF00080; Sod_Fe_C: PF02777 Sod_Fe_N: PF00081) is actually higher in other lophotrochozoans (e.g., the bivalves *Crassostrea*, *Modiolus*, *Mizuhopecten*) than in vestimentiferans (Supplementary Table 6).

# **SUPPLEMENTARY MATERIAL AND METHODS**

# **1 GENOME SEQUENCING AND ASSEMBLY**

## **1.1 Sample collection, genomic DNA extraction and Sequencing Strategy**

*Riftia* genomic DNA was obtained from a piece of vestimentum tissue belonging to single worm collected at the hydrothermal vent site Tica, East Pacific Rise (Alvin dive 4839, 9º 50.398 N, 104º 17.506 W, 2514 m depth, 2016) (Supplementary Figures 1, 2). The preparation of the vestimentum sample was performed by grinding 100mg of frozen tissue with a pre-chilled mortar. After a fine powder was obtained, the tissue was added into a 50 mL conical tube with 9.5 mL of Buffer G2, 19 μL of RNAse A (200 μg/mL final concentration), and 0.5 mL of QIAGEN protease (all reagents except for RNAse A are part of the QIAGEN Blood and Cell Culture Midi Kit (catalog #13343)). The tissue and the reagents were thoroughly mixed in a vortexer, and subsequently incubated at 50 °C for two hours. Lysate was immediately loaded onto an equilibrated QIAGEN Genomic-tip upon completion of incubation step. The Genomic-tip was double washed with 7.5 mL of Buffer QC and then genomic DNA was eluted with 5 mL of Buffer QF (pre-warmed to 50 °C). High-molecular weight DNA was precipitated using 3.5 mL of isopropanol and centrifuged at 5,000x g for 15 minutes at 4 °C. The supernatant was carefully removed without disturbing the pellet. The sample was air-dried for ten min and resuspended in in 200 μL of Tris-HCl, pH 8.5. DNA was dissolved overnight a shaker. Finally, the DNA was quantified and stored at -80 °C. PacBio libraries were generated with Sequel technology for a *de novo* large insert library (30kb) using the SMRTbell Express Template Kit 2.0 and purified *Riftia* DNA by the University of Minnesota Genomics Center (St. Paul, MN, USA).

## **1.2 Genome pre-processing**

The five PacBio libraries were individually converted from the native BAM to fastq file format using bam2fastq tool v1.3.0 present in the “PacBio Secondary Analysis Tools on Bioconda” (<https://github.com/PacificBiosciences/pbbioconda>). The resulting libraries were then mapped with minimap v2.17-r941 (Li 2018) against a custom database of contaminants composed of the closed genome of the *Cand.* Endoriftia persephone and the *Riftia pachyptila* reference mitochondrial genome (Accession number: NC_026860). All PacBio read sequences that failed to align against any of the references were stored in a file and used in the assembly procedure.

## **1.3 Genome assemblies**

The long filtered PacBio reads were assembled with canu v1.8 (Koren et al. 2017) using the parameters optimised for Sequel chemistry and heterozygosity (see <https://github.com/marbl/canu/issues/1470> for details). An alternative assembly, as a purpose of benchmarking and quality control, was produced using the tool flye v2.5 under default parameters (Kolmogorov et al. 2019). The quality assessment of the draft genomes was performed with quast v5.0.2 (Gurevich et al. 2013).

## **1.4 Genome post-processing**

### **1.4.1 Polishing**

To obtain a high-quality consensus draft genome and call variant types (e.g., insertion and deletions), the native PacBio BAM files were aligned against the genome assemblies with pbmm2 tool v1.0.0 (<https://github.com/PacificBiosciences/pbmm2>), a minimap2 SMRT wrapper for PacBio data present in the “PacBio Secondary Analysis Tools on Bioconda” (<https://github.com/PacificBiosciences/pbbioconda>). The mapped results together with the assembly file, were submitted to arrow v2.3.3 (<https://github.com/pacificbiosciences/genomicconsensus/>) for genome polishing and variant calling. The quality assessment of the polished high-quality draft genomes was performed with quast v5.0.2 (Gurevich et al. 2013).

### **1.4.2 Purging haplotigs and contig overlaps**

The removal of haplotigs and contig overlaps was performed manually with purge_dups using the recommended pipeline available at: <https://github.com/dfguan/purge_dups>. The read depth cutoffs were manually adjusted based on histogram plots (e.g., lower, and upper bounds for read depth) in order to avoid overpurging. The read depth was calculate aligning the PacBio original data against the polished genomes with minimap v2.17-r941 (Li 2018). The quality assessment of the purged polished high-quality draft genome was performed with quast v.5.0.2 (Gurevich et al. 2013).

### **1.4.3 Contamination screening with blobtools**

To further assess microbial contamination, a blobplot was generated following the recommended “Workflow A” available at <https://blobtools.readme.io/docs/the-blobtools-workflows#section-workflow-a>. The blobtools v1.1.1 (Laetsch and Blaxter 2017) was executed with: (1) a coverage file obtained by mapping the five filtered long-read PacBio libraries (i.e., libraries without mitochondrial and *Cand.* Endoriftia persephone bacterial reads) against the purged polished genome; (2) the *Riftia pachyptila* purged polished high-quality draft genome; and (3) a hit file containing NCBI TaxIDs resulted from the alignment of the draft tubeworm genome against the nt database (57,030,965 sequences) using the blastn v2.8.1+ (Camacho et al. 2009). Two scaffolds assigned to bacterial TaxIDs were carefully inspected using three additional methods:

1. Similarity search approach: the predicted protein coding sequences from the two suspicious scaffolds were aligned against the NCBI nr database (229,636,095 sequences) with diamond blastp v0.9.25.126 (Buchfink et al. 2015). The TaxIDs from the best diamond blastp hits, were checked for consistent and narrow patterns of bacterial phyletic distribution with MEGAN v6.18.5 (Huson et al. 2016).
2. Gene architecture and gene density approach: the presence of intronless genes (i.e., single exon proteins) and the gene density in the suspicious scaffolds were analysed, as prokaryotic genomes present a much higher gene density than eukaryotes and do not contain any introns.
3. Genomic completeness approach: the suspicious scaffolds were checked for bacterial marker genes with checkM v1.1.2 (Parks et al. 2015).

## **1.5 Mitochondrial genome assembly and annotation**

The PacBio reads mapped against the *Riftia* reference mitochondrial genome were assembled using flye v2.5 (Kolmogorov et al. 2019). The polishing of the extrachromossomal genome was performed as described in section 1.4.1. The fully reconstructed mitochondrial genome was annotated with MITOS2 (Bernt et al. 2013: 2) (<http://mitos2.bioinf.uni-leipzig.de/index.py>) and GeSeq (Tillich et al. 2017) (<https://chlorobox.mpimp-golm.mpg.de/geseq.html>) online servers. Manual curation of the mitogenome was performed using the previous *Riftia* mitogenomes available at NCBI as references. The circular plots generated with ConcatMap (<https://github.com/darylgohl/ConcatMap>) and circos (Krzywinski et al. 2009).

# **2 TRANSCRIPTOME SEQUENCING AND ASSEMBLY**

## **2.1 Sample collection, total RNA extraction and Sequencing Strategy**

One female *Riftia* (24 cm length) was collected from the hydrothermal vent Rebecca’s Roost (27 0.64548684 N, 111 24.41801816 W, 2012 m depth), Guaymas Basin, Feburary 27 2019 during SuBastian dive 231. One male (75 cm length) was collected from a vent close to Big Pagoda (27 0.82596514 N, 111 24.66462214 W, 2028 m depth), Guaymas Basin, March 1 2019 during SuBasian dive 233. Tissue samples from one female (plume, vestimentum, body wall (skin), trophosome, blood and gonad) and one male individual (sperm and blood) were obtained, immerged in RNAprotect Bacterial Reagent (Quiagen) prior freezing at -80 degrees. The RNA from the RNAprotect stabilized tissue samples was purified using RNeasy Plus Mini Kit (Qiagen Cat. No. 74134). Sample disruption and homogenization was performed as follows: not more than 30mg of RNAlater stabilized tissue was transferred to a sterile RNase free 2ml reaction tube, which was pre-cooled by liquid nitrogen. The tissue samples were disrupted by grinding them to a fine powder under liquid nitrogen; 600ul of RTL buffer was added and the lysate was transferred to a QIAshredder spin column (Qiagen Cat. No. 79656) for homogenization. The following gDNA elimination and RNA purification steps were performed following the suppliers instructions, and finally, the purified RNA (30ul) was immediately stored -80° C.The RNA samples were sent to the Vienna Biocenter Core Facility (VBCF: <https://www.viennabiocenter.org/facilities/next-generation-sequencing/>) and stranded paired-end RNA-seq libraries (2x150 pb) of the eight adult tissues were constructed using the NEB/poly-A kit followed by an ultra-deep sequencing with the Illumina NovaSeq SP platform.

**2.2 Transcriptome pre-processing**

The quality of the RNA-seq libraries was assessed with fastqc v0.11.8 (<https://www.bioinformatics.babraham.ac.uk/projects/fastqc/>). Based on the reported statistics obtained from bbduk v38.42 (<https://sourceforge.net/projects/bbmap/>) we selected the most appropriated parameters to filter the raw paired-end RNA-seq libraries. Illumina adapter sequences as well as poor quality bases/reads were trimmed from the tubeworm transcriptomic libraries and the final high-quality RNA-seq libraries used in the subsequent analyses.

## **2.3 Transcriptome assembly**

The eight high-quality paired-end RNA-seq libraries were individually assembled using two distinct approaches: (1) *de novo* strategy with transabyss v2.0.1 (Robertson et al. 2010), and (2) reference-based strategy using the draft *Riftia pachyptila* genome, STAR aligner v2.7.1a and Stringtie v2.0.6 (Dobin et al. 2013; Kovaka et al. 2019).

### **2.3.1 *De novo* transcriptome assembly**

The filtered and trimmed RNA-seq libraries were assembled using transabyss v.2.0.1 (Robertson et al. 2010) with default parameters, paired-end and strand-specific modes activated and the minimum transcript length defined as 200 base pairs. The quality assessment of the *de novo* transcriptome was performed with quast v5.0.2 (Gurevich et al. 2013).

### **2.3.2 Reference-based assembly**

The filtered and trimmed RNA-seq libraries were mapped against the hard-masked draft genome of *Riftia pachyptila* with STAR v2.7.1a (Dobin et al. 2013). The mapping results in BAM format were sorted and index with samtools v1.9-138 (Li et al. 2009) and the transcriptomes reconstructed with Stringtie v2.0.6 (Kovaka et al. 2019). To obtain a global non-redundant transcriptome, Stringtie v2.0.6 was executed again with the “transcript merge mode” invoked, using as input the eight reference-based reconstructed tissue specific transcriptomes. The quality assessment of the global non-redundant transcriptome was performed with quast v5.0.2 (Gurevich et al. 2013).

## **2.4 *De novo* transcriptome post-processing**

### **2.4.1 Removal of endosymbiont contamination**

To remove any possible endosymbiont contamination, the *de novo* transcriptome assemblies were submitted to blastn v2.8.1+ (Camacho et al. 2009) similarity searches against a custom database composed of the combined *Riftia pachyptila* hard-masked draft genome and the closed genome of *Cand.* Endoriftia Persephone (De Oliveira, under revision). Transcripts that aligned exclusively to the reference bacterial genome or mapped to both bacterial and annelid genomes (tagged as “suspicious transcripts”) were removed from the transcriptomes.

### **2.4.2 Generating a *de novo* global non-redundant transcriptome**

In order to obtain a global *de novo* non-redundant transcriptome, a “SuperTranscript” approach was employed (Davidson et al. 2017a; Davidson et al. 2017b) using the tools Bowtie v2.3.4.3, Corset v1.09 (<https://github.com/Oshlack/Corset>) and Lace v1.13 (<https://github.com/Oshlack/Lace>) (Langmead and Salzberg 2012; Davidson and Oshlack 2014). Briefly, the trimmed high-quality RNA-seq paired-end reads were multi-mapped against the *de novo* transcriptomes using Bowtie v2.3.4.3 and the mapping results used to cluster the transcripts into genes with Corset. Finally, Lace v1.13 was used to remove the redundancy and build the global *de novo* “SuperTranscriptome”. The quality assessment of the *de-novo* global non-redundant transcriptome was performed with quast v5.0.2 (Gurevich et al. 2013).

## **2.5 Prediction of the coding sequence regions**

The global *de novo* and reference-based non-redundant transcriptomes, as well as the individual tissue-specific *de novo* transcriptomes were searched for candidate coding sequence regions (CDS) with TransDecoder.LongOrfs and TransDecoder.Predict v.5.5.0 (https://github.com/TransDecoder/TransDecoder). TransDecoder runs were carried out following three criteria: (1) the coding sequence regions must contain a minimum of 100 amino acids; (2) only the top strand must be analysed, due the strand-specificity of the RNA-seq libraries; and (3) CDS with homology against PFAM-A v32.0 (17,929 pHMMs - <http://hmmer.org/>) and/or Swiss-Prot v2019_11 (561,568 sequences - “UniProt,” 2019) databases were retained in the final output. The homology searches were performed with hmmscan v3.1b2 and blastp v2.8.1+ (Camacho et al. 2009; Mistry et al. 2013), and all the file conversions required from TransDecoder to work properly were achieved following the TransDecoder online tutorial (<https://github.com/TransDecoder/TransDecoder/wiki>). The quality of the final predicted proteomes obtained from the global non-redundant *de novo* and reference-based transcriptomes was measured with BUSCO4 (Simão et al. 2015).

# **3 GENOME ANNOTATION**

## **3.1 Identification of interspersed repetitive regions and low complexity DNA**

*De novo* transposable element families were modeled and identified in the tubeworm genome with RepeatModeler v2.0 (A.F.A Smit, R. Hubley & P. Green, *RepeatMasker Open-4.0*), which employs three complementary *de novo* repeat finding software RECON, RepeatScout and LtrHarvest/Ltr_retriever (Bao 2002; Price et al. 2005). The masking of the interspersed repetitive regions and low complexity DNA in the *Riftia pachyptila* genome was performed with RepeatMasker v4.0.9 using the custom database of repetitive sequences generated by RepeatModeler v2.0. The Repeat landscape was generated using the accessory scripts calcDivergenceFromAlign.pl and createRepeatLandscape.pl available at the RepeatMasker v4.0.9 package.

## **3.2 *Ab initio* gene predictions**

The *ab initio* gene predictions were performed with AUGUSTUS v3.3.3 (Stanke and Morgenstern 2005; Hoff and Stanke 2018). The *Riftia* gene models required for training AUGUSTUS were obtained using an unsupervised procedure implemented by GeneMark-ES version 4.48_3.60_lic (Lomsadze et al. 2014) followed by homology searches with diamond v0.9.25.126 (Buchfink et al. 2015) against nr database. The hints files, i.e., extrinsic structural/positional information obtained from the RNA-seq data about gene, intron, and exon boundaries, as well as the correct coding reading frame, were obtained using the combined *de novo* and reference based TransDecoder results (see section 2.5) and a comprehensive bioinformatic protocol (Hoff and Stanke 2018). Finally, the *Riftia* UTR gene models were retrieved from the Transdecoder gff3 genome annotation files and included only genes with complete coding sequence regions and both 3’- and 5’ UTR features annotated. These genes were further filtered through sequence similarity searches against the nr database using diamond blastp v0.9.25.126. A total of three gene predictions were performed: (1) gene prediction without any hints files; (2) gene prediction with hints file without UTR model, and; (3) gene prediction with hints file and UTR model activated. The three gene predictions were merged with the tool joingenes, and the coding sequence regions (in both amino acid and nucleotide format) were extract with getAnnoFastaFromJoingenes.py (both softwares available as auxiliary tools in the AUGUSTUS V3.3.3 package).

## **3.3 Filtering AUGUSTUS gene model predictions**

The merged AUGUSTUS prediction was further submitted to homology searches against the PFAM-A v32.0 and nr databases using hmmsearch v3.1b2 and diamond blastp v0.9.25.126 tools. Additionally, the eight trimmed and filtered tubeworm tissue specific transcriptomes were mapped against the merged AUGUSTUS gene models with kallisto v0.46.1 (Bray et al. 2016), and orthology inferences were performed with orthoFinder v2.3.8 (Emms and Kelly 2019) using the *Riftia pachyptila* AUGUSTUS predicted proteins and other four publicly available annelid genomes (Simakov et al. 2013; Paul et al. 2018; Li et al. 2019). Only gene models with sequence similarity against the protein databases, and/or orthology and evidence of gene expression were retained. The quality of the combined AUGUSTUS gene prediction was measured with BUSCO4 (Simão et al. 2015).

## **3.4 Protein annotation**

The predicted protein sequences were scanned for protein domains and functional sites using Interproscan 5.39-77.0 (Jones et al. 2014) and 16 publicly available databases (TIGRFAM v15.0, SFLD v4, Phobius v1.01, SUPERFAMILY v1.75, Gene3D v4.2.0, Hamap v2019_01, ProSiteProfiles v2019_01, Coils v2.2.1, SMART v7.1, CDD v3.17, PRINTS v42.0, ProSitePatterns v2019_01, Pfam v32.0, MobiDBLite v2.0, PIRSF v3.02, TMHMM v2.0c). The prediction of tRNA and signal peptide sequences were performed with tRNAscan-SE 2.0.5 and signalP v5.0b, respectively (Nawrocki and Eddy 2013; Lowe and Chan 2016; Almagro Armenteros et al. 2019).

## **3.5 Protein family analyses (PFAM)**

- 1. The pfam_scan.pl script (<ftp://ftp.ebi.ac.uk/pub/databases/Pfam//Tools/PfamScan.tar.gz>) was used to align selected lophotrochozoan protein sequences against a database of HMM profiles (Pfam-A v.33.0) using the standard hmmscan program (v3.1b2) (Mistry et al. 2013). This software, in additional to the standard hmmscan searches, clusters query proteins which share a common evolutionary ancestor whilst showing only the most significant match within the given PFAM clan.

## **3.6 *Riftia* gene toolkits essential for development, homeostasis, and body patterning**

### **3.6.1 Antennapedia class**

Using blastp v2.8.1+ the predicted *Riftia pachyptila* and *Lamellibrachia luymesi* protein sequences were searched against a well-curated catalog of metazoan Antennapedia class genes (Simakov et al. 2013; Zwarycz et al. 2016; Luo et al. 2018; Li et al. 2019). Additionally, Antennapedia gene ortholog representatives were downloaded from the NCBI protein database when necessary. The tubeworms protein candidates were aligned together with the metazoan homologues with mafft v7.450 (Katoh and Standley, 2013) and the multiple sequence alignments were manually trimmed to remove gappy and dissimilar regions. Independent molecular phylogenetic analyses were performed with iqtree v1.6.11 combining ModelFinder, tree search, 1000 ultra-fast bootstrap and SH-aLRT test replicates (Nguyen et al. 2015; Kalyaanamoorthy et al. 2017; Hoang et al. 2018).

### **3.6.2 Transcription factors**

Unique lophotrochozoan non-overlapping genes containing protein domains with similarity to the largest transcription factor families (bZIP, p53, homeobox, NuclearFactor, bHLH and zfc2h2) (Degnan et al. 2009; Vaquerizas et al. 2009; Schmitz et al. 2016) were quantified based on the results of the pfam_scan.pl analyses (section 3.5).

### **3.6.3 Signalling, amino/fatty acid biosynthesis, endocytosis-, autophagy-, apoptosis- and immune-related gene toolkits**

*Riftia pachyptila* genes containing protein domains associated with known signalling molecules (Wnt ligand and their Frizzled receptors, Hedgehog, Patched, Notch and TGF-beta), amino/fatty acid biosynthesis (mitochondrial beta oxidation), endocytosis (RAB), autophagy (ATG, ULK), apoptosis-, and immune-related genes (Caspases, pro-caspases, Tumor Necrosis Factor ligands/receptor, Inhibitor of apoptosis, BCL, Toll-like, Superoxide dismutase) were retrieved from the pfam_scan.pl results (section 3.5). The orthology of the selected proteins were confirmed through similarity searches against the NCBI nr database (229,636,095 sequences) using diamond blastp v0.9.25.126 and phylogenetic inferences with iqtree v1.6.11, as described previously (section 3.6.1). The protein diagrams were drawn using IBS v.1.0.3 software (Liu et al. 2015) and the clustered heatmaps generated with the R package pheatmap (v1.0.12) (<https://www.rdocumentation.org/packages/pheatmap/versions/1.0.12/topics/pheatmap>).

### **3.6.4 Haemoglobin gene identification, characterisation, phylogenetic inferences and gene expression quantification**

Using blastp v2.8.1+ (Camacho et al. 2009) the predicted *Riftia pachyptila* protein sequences were searched against a well-curated publicly available metazoan haemoglobin and linker sequences. The tubeworm haemoglobin candidates were interrogated for the presence of the globin domain (PF00042) with hmmalign v3.1b2 and proteins without a hit were excluded from the analyses. Manual inspection and characterisation of the signature diagnostic residues/motifs in the haemoglobin chain and linker sequences were performed following previous works (Zal et al. 1996; Zal et al. 1997; Bailly et al. 2002; Bailly et al. 2003; Flores et al. 2005; Waits et al. 2016; Li et al. 2019). Multiple sequence alignments, trimming and phylogenetic analyses were carried out as described in the section 3.5. The resulting trees were midpoint rooted using Figtree (<http://tree.bio.ed.ac.uk/software/figtree/>). Additionally, to investigate the haemoglobin gene expression across different environmental conditions (sulphur rich and sulphur depleted) we downloaded six publicly available trophosome transcriptomes from SRA (<https://www.ncbi.nlm.nih.gov/sra>) (accession numbers: SRR8949066 to SRR8949071). The transcriptome libraries were pre-processed as described in section 2.2 (Transcriptome pre-processing). Quantification was performed with kallisto tool (Bray et al. 2016). The clustered heatmap was generated as previously described (section 3.6.3).

### **3.6.5 Homology model generation of *Riftia* Hb**

RIFPA_jg20259.t3-HB1c-SG1 mature sequence (Met63-Pro213), was modelled after the deoxygenated 400 kda haemoglobin structure from *Lamellibrachia satsuma* (pdb ID: 3WCT, resolution 2.4Å, sequence identity: 35.6%). Template was selected based on sequence and secondary structure similarity using HH-Pred online. Three‐dimensional protein structures for the full complex were generated by homology modelling using the Prime program implemented in the Schrödinger Drug Discovery (v2020.2) software suite with standard options using secondary-structure prediction option to guide the query – template alignment. Protein complexes were prepared using the protein preparation tool and further refined with a restrained minimization step, allowing the heavy atoms to vary within 0.5 Å of limit, to fix sterical clashes. All models were inspected for Ramachandran outliers, which were fixed by energy minimization. All illustrations of structures were made with PyMol v2.4 (<https://pymol.org/2/>).

# **4 COMPARATIVE GENOMICS AND GENE FAMILIES ANALYSES**

## **4.1 Orthology**

36 representative animal species broadly distributed across the metazoan tree were selected for gene family analyses: *A. queenslandica*, *M. leidyi*, *N. vectensis*, *T. adhaerens* (non-bilaterian group); *A. vaga*, *B. platifrons*,  *B. glabrata*, *C. teleta*, *C. squamiferum*, *C. gigas*, *E. multilocularis*, *H. robusta*, *L. luymesi, L. anatina*, *L. gigantea, M. philippinarum*, *N. geniculatus*, *O. bimaculoides*, *M. yessoensis*, *P. australis*, *R. pachyptila*, *S. mansoni*, *S. mediterranea* (lophotrochozoan group); *B. floridae*, *C. intestinalis*, *D. rerio*, *G. gallus*, *H. sapiens*, *M. musculus*, *S. purpuratus*, *X. tropicalis* (deuterostomian group); *A. gambiae*, *C. elegans*, *D. pulex*, *D. melanogaster*, *T. castaneum* (ecdysozoan group). Redundancy was removed from the gene sets based on the gene coordinates specified in the gff3 files and only the longest isoform for each gene was kept. Orthofinder v2.3 (Emms and Kelly 2019) was used to define the orthogroups, and groups belonging only to *Capitella+Helobdella+Lamelibrachia+Riftia, Lamellibrachia+Riftia* and only *Riftia* were considered: Annelida-, Vestimentifera- and *Riftia*-specific, respectively. Enrichment analysis for Gene Ontology and protein annotation with PANTHER HMM scoring tool were performed with the *Riftia*-, *Lamellibrachia* and Vestimentifera-specific gene families as described in the section 4.2. To identify statistically significant gene family expansions/contractions in *Riftia* compared to other lophotrochozoans a second round of Orthofinder v2.3.8 was performed using 18 lophotrochozoan representatives (all except the bdelloid rotifer *Adineta vaga*, which was removed due the tetraploidy nature of its genome (Flot et al. 2013; Nowell et al. 2018), and *Tribolium castaneum* as outgroup. Finally, to identify the Annelida gene family core and the shared orthogroups only among annelids, a final instance of orthofinder was invoked using *C. teleta, H. robusta, L. luymesi* and *R. pachyptila.*

## **4.2 Gene family expansions/contractions with CAFE**

The 67 single-copy ortholog groups shared among the 18 lophotrochozoans *+ T. castaneum* were individually aligned with mafft v7.450 (Katoh and Standley 2013). The resulting 67 multiple sequence alignment files were concatenated into a protein supermatrix using FASconCAT-G v1.04 (Kück and Meusemann 2010), and subsequently trimmed with BMGE v1.12 (Criscuolo and Gribaldo 2010). The final concatenated trimmed alignment with 23,975 positions was submitted to Bayesian phylogenetic inferences using Phylobayes v4.1b with CAT-GTR model defined and a starting lophotrochozoan tree generated by FastTree version 2.1.3-SSE3 (De Bie et al. 2006; Lartillot and Philippe 2006; Benton et al. 2009; Price et al. 2010; Han et al. 2013).Three calibrations points based on fossil data were used based on Benton et al., (2009): (1) *C. teleta –* *H. robusta* = 581 MYA – 305 MYA; (2) *C. teleta –* *L. gigantea* = 581 MYA – 531 MYA; (3) *L. gigantea –* *B. glabrata* = 531 MYA – 470 MYA. The age of the root (i.e., Protostomia split) was set as 600 MYA. The ultrametric tree was obtained after 31,665 rounds discarding the initial 7,916 rounds as burn-in (25%). Gene family expansions and contractions were statistically analysed using the time tree generated by Phylobayes v4.1b and the lophotrochozoan orthology inference described in the section 4.1 with the CAFE v4.2.1 (De Bie et al. 2006). CAFE was executed with four different lambda values and the appropriate error model. The contracted/expanded gene families were annotated with Interproscan 5.39-77.0 (Jones et al. 2014) and the Enrichment analysis for Gene Ontology was performed as described in the section 5.2. Rapidly evolving gene families in *R. pachyptila* were annotated using PANTHER HMM scoring tool v2.2 (<ftp://ftp.pantherdb.org/hmm_scoring/current_release/>) with PANTHER_hmmscore database v15 (Thomas 2003; Han et al. 2013; Mi et al. 2017).

## **4.3 Gene family expansion/contraction with PFAM**

Annotation of the PFAM domains was performed with 18 selected lophotrochozoan taxa (section 4.1) using the pfam_scan.pl (section 3.5). Protein domains associated with transposable elements (e.g., Helicase, Helitron, DDE_Tnp) and identified as DUF (domain of unknown function) were removed from the analysis. As described in Albertin et al. (2019) and Sun et al. (2017), reoccurring identical domains present in the same protein sequence were counted only once. An iterative two-tailed Fisher’s exact test was implemented in R (<https://www.r-project.org/>) to identify protein domain contractions/expansions. The obtained p-values were corrected using Benjamini and Hochberg method (Benjamini and Hochberg 1995) and only domains with a significant p-value of < 0.01 were further investigated. Four sets of comparisons were performed:

1. Set 1: *Riftia* vs. *Lamellibrachia*;
2. Set 2: *Riftia* vs. remaining non-vestimentiferan lophotrochozoans (background average domain counts of 16 taxa – all except *L. luymesi* and *R. pachyptila*);
3. Set 3: *Lamellibrachia* vs. remaining non-vestimentiferan lophotrochozoans (background average domain counts of 16 taxa – all except *L. luymesi* and *R. pachyptila*);
4. Set 4: Hydrothermal vents lophotrochozoans with symbiotic association (i.e., *B. platifrons, C. squamiferum*, *R. pachyptila* and *L. luymesi*) vs. remaining lophotrochozoan species (background average domain counts of 12 taxa).

## **4.4 Clustering of *Riftia* expanded PFAM-containing genes with clans**

A dataset composed of the *Riftia pachyptila* expanded PFAM-containing genes was clustered during approximately 21,000 rounds with the program clans, a Java application for visualising protein families based on all-against all comparisons (<ftp://ftp.tuebingen.mpg.de/pub/protevo/CLANS/>) (Frickey and Lupas 2004). The final 3D maps were collapsed to 2D after the clustering for easier visualisation.

## **4.5 Synonymous and non-synonymous substitution rates analyses**

Non-synonymous (Ka) and synonymous (Ks) substitution rates were calculated with the stand-alone version of KaKs_calculator v.2 using the orthofinder v2.3.8 results for the Lophotrochozoa and Annelida set (Wang et al. 2010; Emms and Kelly 2019). A modified version of pal2nal.pl (Suyama et al. 2006) software, called Epal2nal v.13 and available at the ParaAT v2.0 package (<https://bigd.big.ac.cn/tools/paraat/>) (Z. Zhang et al. 2012), was used to generate codon alignments in axt format required by KaKs_calculator. Additionally, potential genes under positive selection in *R. pachyptila* genome were screened in the Annelida and Lophotrochozoa orthogroups using the adaptive branch site-random effects likelihood (aBS-REL) model implemented in HyPhy v. 2.5.15 (Pond et al. 2005; Smith et al. 2015). Only single-copy genes (1:1 orthologs) without any inconsistencies between the nucleotide and protein sequences were used in the analyses.

# **5 GENE EXPRESSION ANALYSES**

## **5.1 Gene expression quantification and identification of absolutely tissue specific genes**

The eight pre-processed *Riftia pachyptila* transcriptome libraries were pseudoaligned against the merged filtered AUGUSTUS gene models with kallisto v.0.46.1 to collect the gene expression data expressed as TPM counts (transcripts per million) (Bray et al. 2016; Hoff and Stanke 2018). Normalisation within and across tissues were independently performed before calculating the tissue specificity tau values (see <https://rdrr.io/github/roonysgalbi/tispec/f/vignettes/UserGuide.Rmd>) (Yanai et al. 2005; Kryuchkova-Mostacci and Robinson-Rechavi 2016). To mitigate possible sex-specific differences in the gene expression levels, tau calculations were performed using only the tubeworm female tissues.

## **5.2 Gene set enrichment analyses**

The absolutely tissue specific genes (genes expressed only in a single tissue defined by a tau value of 1) were submitted to enrichment analyses for Gene Ontology with topGO v2.36.0 (<https://bioconductor.org/packages/release/bioc/html/topGO.html>) using Fisher’s exact test against the *R. pachyptila* background (i.e., complete set of *Riftia* genes) coupled with weight01 algorithm (Alexa et al., 2006).

# **REFERENCES**

Ajioka RS, Phillips JD, Kushner JP. 2006. Biosynthesis of heme in mammals. *Biochim. Biophys. Acta BBA - Mol. Cell Res.* 1763:723–736.

Albertin CB, Simakov O, Mitros T, Wang ZY, Pungor JR, Edsinger-Gonzales E, Brenner S, Ragsdale CW, Rokhsar DS. 2015. The octopus genome and the evolution of cephalopod neural and morphological novelties. *Nature* 524:220–224.

Aldridge S, Teichmann SA. 2020. Single cell transcriptomics comes of age. *Nat. Commun.* 11:4307.

Almagro Armenteros JJ, Tsirigos KD, Sønderby CK, Petersen TN, Winther O, Brunak S, von Heijne G, Nielsen H. 2019. SignalP 5.0 improves signal peptide predictions using deep neural networks. *Nat. Biotechnol.* 37:420–423.

Arendt D, Technau U, Wittbrodt J. 2001. Evolution of the bilaterian larval foregut. *Nature* 409:81–85.

Bailly X, Jollivet D, Vanin S, Deutsch J, Zal F, Lallier F, Toulmond A. 2002. Evolution of the Sulfide-Binding Function Within the Globin Multigenic Family of the Deep-Sea Hydrothermal Vent Tubeworm Riftia pachyptila. *Mol. Biol. Evol.* 19:1421–1433.

Bailly X, Leroy R, Carney S, Collin O, Zal F, Toulmond A, Jollivet D. 2003. The loss of the hemoglobin H2S-binding function in annelids from sulfide-free habitats reveals molecular adaptation driven by Darwinian positive selection. *Proc. Natl. Acad. Sci.* 100:5885–5890.

Bao Z. 2002. Automated De Novo Identification of Repeat Sequence Families in Sequenced Genomes. *Genome Res.* 12:1269–1276.

Bartolomaeus T, Quast B. 2005. Structure and development of nephridia in Annelida and related taxa. In: Bartolomaeus T., Purschke G, editors. Morphology, Molecules, Evolution and Phylogeny in Polychaeta and Related Taxa. Developments in Hydrobiology. Dordrecht: Springer Netherlands. p. 139–165. Available from: https://doi.org/10.1007/1-4020-3240-4_9

Belato FA, Schrago CG, Coates CJ, Halanych KM, Costa-Paiva EM. 2019. Newly Discovered Occurrences and Gene Tree of the Extracellular Globins and Linker Chains from the Giant Hexagonal Bilayer Hemoglobin in Metazoans.Eyre-Walker A, editor. *Genome Biol. Evol.* 11:597–612.

Belcaid M, Casaburi G, McAnulty SJ, Schmidbaur H, Suria AM, Moriano-Gutierrez S, Pankey MS, Oakley TH, Kremer N, Koch EJ, et al. 2019. Symbiotic organs shaped by distinct modes of genome evolution in cephalopods. *Proc. Natl. Acad. Sci.* 116:3030–3035.

Benjamini Y, Hochberg Y. 1995. Controlling the False Discovery Rate: A Practical and Powerful Approach to Multiple Testing. *J. R. Stat. Soc. Ser. B Methodol.* 57:289–300.

Benton MJ, Donoghue PCJ, Asher RJ. 2009. Calibrating and constraining molecular clocks. :53.

Bergquist DC, Ward T, Cordes EE, McNelis T, Howlett S, Kosoff R, Hourdez S, Carney R, Fisher CR. 2003. Community structure of vestimentiferan-generated habitat islands from Gulf of Mexico cold seeps. *J. Exp. Mar. Biol. Ecol.* 289:197–222.

Bergquist DC, Williams FM, Fisher CR. 2000. Longevity record for deep-sea invertebrate. *Nature* 403:499–500.

Bernt M, Donath A, Jühling F, Externbrink F, Florentz C, Fritzsch G, Pütz J, Middendorf M, Stadler PF. 2013. MITOS: Improved de novo metazoan mitochondrial genome annotation. *Mol. Phylogenet. Evol.* 69:313–319.

Bishop BM, Juba ML, Russo PS, Devine M, Barksdale SM, Scott S, Settlage R, Michalak P, Gupta K, Vliet K, et al. 2017. Discovery of Novel Antimicrobial Peptides from Varanus komodoensis (Komodo Dragon) by Large-Scale Analyses and De-Novo-Assisted Sequencing Using Electron-Transfer Dissociation Mass Spectrometry. *J. Proteome Res.* 16:1470–1482.

Bonnivard E, Catrice O, Ravaux J, Brown SC, Higuet D. 2009. Survey of genome size in 28 hydrothermal vent species covering 10 families. *Genome* 52:524–536.

Boyle MJ, Seaver EC. 2008. Developmental expression of foxA and gata genes during gut formation in the polychaete annelid, Capitella sp. I. *Evol. Dev.* 10:89–105.

Bray NL, Pimentel H, Melsted P, Pachter L. 2016. Near-optimal probabilistic RNA-seq quantification. *Nat. Biotechnol.* 34:525–527.

Bright M, Eichinger I, von Salvini-Plawen L. 2013. The metatrochophore of a deep-sea hydrothermal vent vestimentiferan (Polychaeta: Siboglinidae). *Org. Divers. Evol.* 13:163–188.

Bright M, Lallier F. 2010. The biology of vestimentiferan tubeworms. In: Gibson, RN, Atkinson, RJA, Gordon, JDM, editors. Oceanography and Marine Biology: An animal review, Vol 48. Vol. 48. Oceanography and Marine Biology. CRC PRESS-TAYLOR & FRANCIS GROUP. p. 213–265. Available from: https://hal.archives-ouvertes.fr/hal-01250932

Bright M, Sorgo A. 2003. Ultrastructural reinvestigation of the trophosome in adults of Riftia pachyptila (Annelida, Siboglinidae). *Invertebr. Biol.* 122:347–368.

Bröker LE, Huisman C, Span SW, Rodriguez JA, Kruyt FAE, Giaccone G. 2004. Cathepsin B Mediates Caspase-Independent Cell Death Induced by Microtubule Stabilizing Agents in Non-Small Cell Lung Cancer Cells. *Cancer Res.* 64:27–30.

Brown SG, Publicover SJ, Barratt CLR, Martins da Silva SJ. 2019. Human sperm ion channel (dys)function: implications for fertilization. *Hum. Reprod. Update* 25:758–776.

Brown WJ, Goodhouse J, Farquhar MG. 1986. Mannose-6-phosphate receptors for lysosomal enzymes cycle between the Golgi complex and endosomes. *J. Cell Biol.* 103:1235–1247.

Buchfink B, Xie C, Huson DH. 2015. Fast and sensitive protein alignment using DIAMOND. *Nat. Methods* 12:59–60.

Burke R, Nellen D, Bellotto M, Hafen E, Senti K-A, Dickson BJ, Basler K. 1999. Dispatched, a Novel Sterol-Sensing Domain Protein Dedicated to the Release of Cholesterol-Modified Hedgehog from Signaling Cells. *Cell* 99:803–815.

Calcino AD, de Oliveira AL, Simakov O, Schwaha T, Zieger E, Wollesen T, Wanninger A. 2019. The quagga mussel genome and the evolution of freshwater tolerance. *DNA Res.* 26:411–422.

Camacho C, Coulouris G, Avagyan V, Ma N, Papadopoulos J, Bealer K, Madden TL. 2009. BLAST+: architecture and applications. *BMC Bioinformatics* 10:421.

de Castro M, Bunt G, Wouters F. 2016. Cathepsin B launches an apoptotic exit effort upon cell death-associated disruption of lysosomes. *Cell Death Discov.* 2:16012.

Celis AI, DuBois JL. 2019. Making and breaking heme. *Curr. Opin. Struct. Biol.* 59:19–28.

Cho JH, Sung BH, Kim SC. 2009. Buforins: Histone H2A-derived antimicrobial peptides from toad stomach. *Biochim. Biophys. Acta BBA - Biomembr.* 1788:1564–1569.

Cho S-J, Vallès Y, Giani VC, Seaver EC, Weisblat DA. 2010. Evolutionary Dynamics of the wnt Gene Family: A Lophotrochozoan Perspective. *Mol. Biol. Evol.* 27:1645–1658.

Cian MD, Regnault M, Lallier FH. 2000. Nitrogen metabolites and related enzymatic activities in the body fluids and tissues of the hydrothermal vent tubeworm Riftia pachyptila. *J. Exp. Biol.* 203:2907–2920.

Collins AJ, Schleicher TR, Rader BA, Nyholm SV. 2012. Understanding the Role of Host Hemocytes in a Squid/Vibrio Symbiosis Using Transcriptomics and Proteomics. *Front. Immunol.* [Internet] 3. Available from: http://journal.frontiersin.org/article/10.3389/fimmu.2012.00091/abstract

Conejo M, Bertin M, Pomponi SA, Ellington WR. 2008. The Early Evolution of the Phosphagen Kinases—Insights from Choanoflagellate and Poriferan Arginine Kinases. *J. Mol. Evol.* 66:11–20.

Cooper CE, Brown GC. 2008. The inhibition of mitochondrial cytochrome oxidase by the gases carbon monoxide, nitric oxide, hydrogen cyanide and hydrogen sulfide: chemical mechanism and physiological significance. *J. Bioenerg. Biomembr.* 40:533–539.

Cordes EE, Bergquist DC, Redding ML, Fisher CR. 2007. Patterns of growth in cold-seep vestimenferans including Seepiophila jonesi: a second species of long-lived tubeworm. *Mar. Ecol.* 28:160–168.

Criscuolo A, Gribaldo S. 2010. BMGE (Block Mapping and Gathering with Entropy): a new software for selection of phylogenetic informative regions from multiple sequence alignments. *BMC Evol. Biol.* 10:210.

Cruciat C-M, Niehrs C. 2013. Secreted and Transmembrane Wnt Inhibitors and Activators. *Cold Spring Harb. Perspect. Biol.* [Internet] 5. Available from: https://www.ncbi.nlm.nih.gov/pmc/articles/PMC3578365/

Czabotar PE, Lessene G, Strasser A, Adams JM. 2014. Control of apoptosis by the BCL-2 protein family: implications for physiology and therapy. *Nat. Rev. Mol. Cell Biol.* 15:49–63.

Dang W. 2014. The controversial world of sirtuins. *Drug Discov. Today Technol.* 12:e9–e17.

Davidson NM, Hawkins ADK, Oshlack A. 2017a. SuperTranscripts: a data driven reference for analysis and visualisation of transcriptomes. *Genome Biol.* 18:148.

Davidson NM, Hawkins ADK, Oshlack A. 2017b. Erratum to: SuperTranscripts: a data driven reference for analysis and visualisation of transcriptomes. *Genome Biol.* 18:160.

Davidson NM, Oshlack A. 2014. Corset: enabling differential gene expression analysis for de novoassembled transcriptomes. *Genome Biol.* 15:410.

De Bie T, Cristianini N, Demuth JP, Hahn MW. 2006. CAFE: a computational tool for the study of gene family evolution. *Bioinformatics* 22:1269–1271.

deCathelineau AM, Henson PM. 2003. The final step in programmed cell death: phagocytes carry apoptotic cells to the grave.Cotter T, editor. *Essays Biochem.* 39:105–117.

Degnan BM, Vervoort M, Larroux C, Richards GS. 2009. Early evolution of metazoan transcription factors. *Curr. Opin. Genet. Dev.* 19:591–599.

Detree C, Chabenat A, Lallier FH, Satoh N, Shoguchi E, Tanguy A, Mary J. 2016. Multiple I-Type Lysozymes in the Hydrothermal Vent Mussel Bathymodiolus azoricus and Their Role in Symbiotic Plasticity. *PLOS ONE* 11:e0148988.

Dixon D, Dixon L, Pascoe P, Wilson J. 2001. Chromosomal and nuclear characteristics of deep-sea hydrothermal-vent organisms: correlates of increased growth rate. *Mar. Biol.* 139:251–255.

Dobin A, Davis CA, Schlesinger F, Drenkow J, Zaleski C, Jha S, Batut P, Chaisson M, Gingeras TR. 2013. STAR: ultrafast universal RNA-seq aligner. *Bioinformatics* 29:15–21.

Doležel J, Bartoš J, Voglmayr H, Greilhuber J. 2003. Letter to the editor. *Cytometry A* 51A:127–128.

Ellington WR. 2001. Evolution and Physiological Roles of Phosphagen Systems. *Annu. Rev. Physiol.* 63:289–325.

Emms DM, Kelly S. 2019. OrthoFinder: phylogenetic orthology inference for comparative genomics. *Genome Biol.* 20:238.

Fernández R, Gabaldón T. 2020. Gene gain and loss across the metazoan tree of life. *Nat. Ecol. Evol.* 4:524–533.

Fisher CR, Childress JJ, Arp AJ, Brooks JM, Distel D, Favuzzi JA, Macko SA, Newton A, Powell MA, Somero GN, et al. 1988. Physiology, morphology, and biochemical composition of Riftia pachyptila at Rose Garden in 1985. *Deep Sea Res. Part Oceanogr. Res. Pap.* 35:1745–1758.

Fisher CR, Childress JJ, Minnich E. 1989. Autotrophic Carbon Fixation by the Chemoautotrophic Symbionts of Riftia pachyptila. *Biol. Bull.* 177:372–385.

Fisher CR, Urcuyo IA, Simpkins MA, NIx E. 1997. Life in the Slow Lane: Growth and Longevity of Cold-seep Vestimentiferans. *Mar. Ecol.* 18:83–94.

Flores JF, Fisher CR, Carney SL, Green BN, Freytag JK, Schaeffer SW, Royer WE. 2005. Sulfide binding is mediated by zinc ions discovered in the crystal structure of a hydrothermal vent tubeworm hemoglobin. *Proc. Natl. Acad. Sci.* 102:2713–2718.

Flot J-F, Hespeels B, Li X, Noel B, Arkhipova I, Danchin EGJ, Hejnol A, Henrissat B, Koszul R, Aury J-M, et al. 2013. Genomic evidence for ameiotic evolution in the bdelloid rotifer Adineta vaga. *Nature* 500:453–457.

Fr R, K B, Jw K, Wc C, D G, Yr M. 1998. Potential role of epithelial cell-derived histone H1 proteins in innate antimicrobial defense in the human gastrointestinal tract. *Infect. Immun.* 66:3255–3263.

Fredriksson R, Schiöth HB. 2005. The Repertoire of G-Protein–Coupled Receptors in Fully Sequenced Genomes. *Mol. Pharmacol.* 67:1414–1425.

Frickey T, Lupas A. 2004. CLANS: a Java application for visualizing protein families based on pairwise similarity. *Bioinformatics* 20:3702–3704.

Futahashi R, Tanaka K, Tanahashi M, Nikoh N, Kikuchi Y, Lee BL, Fukatsu T. 2013. Gene Expression in Gut Symbiotic Organ of Stinkbug Affected by Extracellular Bacterial Symbiont. *PLOS ONE* 8:e64557.

Gaill F, Shillito B, Lechaire JP, Chanzy H, Goffinet G. 1992. The chitin secreting system from deep sea hydrothermal vent worms. *Biol. Cell* 76:201–204.

Gaill F, Shillito B, Ménard F, Goffinet G, Childress J. 1997. Rate and process of tube production by the deep-sea hydrothermal vent tubeworm Riftia pachyptila. *Mar. Ecol. Prog. Ser.* 148:135–143.

Gąsiorowski L, Andrikou C, Janssen R, Bump P, Budd GE, Lowe CJ, Hejnol A. 2021. Molecular evidence for a single origin of ultrafiltration-based excretory organs. *Curr. Biol.*:S0960982221007557.

Gerdol M, Greco S, Pallavicini A. 2019. Extensive Tandem Duplication Events Drive the Expansion of the C1q-Domain-Containing Gene Family in Bivalves. *Mar. Drugs* 17:583.

Gerdol M, Manfrin C, De Moro G, Figueras A, Novoa B, Venier P, Pallavicini A. 2011. The C1q domain containing proteins of the Mediterranean mussel Mytilus galloprovincialis: A widespread and diverse family of immune-related molecules. *Dev. Comp. Immunol.* 35:635–643.

Gerdol M, Venier P, Pallavicini A. 2015. The genome of the Pacific oyster Crassostrea gigas brings new insights on the massive expansion of the C1q gene family in Bivalvia. *Dev. Comp. Immunol.* 49:59–71.

Gerke P, Huber TB, Sellin L, Benzing T, Walz G. 2003. Homodimerization and Heterodimerization of the Glomerular Podocyte Proteins Nephrin and NEPH1. *J. Am. Soc. Nephrol.* 14:918–926.

Girguis PR, Lee RW, Desaulniers N, Childress JJ, Pospesel M, Felbeck H, Zal F. 2000. Fate of nitrate acquired by the tubeworm Riftia pachyptila. *Appl. Environ. Microbiol.* 66:2783–2790.

Glick D, Barth S, Macleod KF. 2010. Autophagy: cellular and molecular mechanisms. *J. Pathol.* 221:3–12.

Gómez-Sánchez R, Rose J, Guimarães R, Mari M, Papinski D, Rieter E, Geerts WJ, Hardenberg R, Kraft C, Ungermann C, et al. 2018. Atg9 establishes Atg2-dependent contact sites between the endoplasmic reticulum and phagophores. *J. Cell Biol.* 217:2743–2763.

Guicciardi ME, Leist M, Gores GJ. 2004. Lysosomes in cell death. *Oncogene* 23:2881–2890.

Gurevich A, Saveliev V, Vyahhi N, Tesler G. 2013. QUAST: quality assessment tool for genome assemblies. *Bioinformatics* 29:1072–1075.

Han MV, Thomas GWC, Lugo-Martinez J, Hahn MW. 2013. Estimating Gene Gain and Loss Rates in the Presence of Error in Genome Assembly and Annotation Using CAFE 3. *Mol. Biol. Evol.* 30:1987–1997.

Hansen M, Rubinsztein DC, Walker DW. 2018. Autophagy as a promoter of longevity: insights from model organisms. *Nat. Rev. Mol. Cell Biol.* 19:579–593.

Hartenstein V. 2006. Blood Cells and Blood Cell Development in the Animal Kingdom. *Annu. Rev. Cell Dev. Biol.* 22:677–712.

Hinzke T, Kleiner M, Breusing C, Felbeck H, Häsler R, Sievert SM, Schlüter R, Rosenstiel P, Reusch TBH, Schweder T, et al. 2019. Host-Microbe Interactions in the Chemosynthetic Riftia pachyptila Symbiosis. 10:20.

Hinzke T, Kleiner M, Meister M, Schlüter R, Hentschker C, Pané-Farré J, Hildebrandt P, Felbeck H, Sievert SM, Bonn F, et al. 2021. Bacterial symbiont subpopulations have different roles in a deep-sea symbiosis. *eLife* 10:e58371.

Hoang DT, Chernomor O, von Haeseler A, Minh BQ, Vinh LS. 2018. UFBoot2: Improving the Ultrafast Bootstrap Approximation. *Mol. Biol. Evol.* 35:518–522.

Hoff KJ, Stanke M. 2018. Predicting Genes in Single Genomes with AUGUSTUS. *Curr. Protoc. Bioinforma.*:e57.

Holstein TW. 2012. The Evolution of the Wnt Pathway. *Cold Spring Harb. Perspect. Biol.* [Internet] 4. Available from: https://www.ncbi.nlm.nih.gov/pmc/articles/PMC3385961/

Hu Y-B, Dammer EB, Ren R-J, Wang G. 2015. The endosomal-lysosomal system: from acidification and cargo sorting to neurodegeneration. *Transl. Neurodegener.* 4:18.

Huber TB, Schmidts M, Gerke P, Schermer B, Zahn A, Hartleben B, Sellin L, Walz G, Benzing T. 2003. The Carboxyl Terminus of Neph Family Members Binds to the PDZ Domain Protein Zonula Occludens-1 *. *J. Biol. Chem.* 278:13417–13421.

Hui JH, Raible F, Korchagina N, Dray N, Samain S, Magdelenat G, Jubin C, Segurens B, Balavoine G, Arendt D, et al. 2009. Features of the ancestral bilaterian inferred from Platynereis dumerilii ParaHox genes. *BMC Biol.* 7:43.

Husnik F, Nikoh N, Koga R, Ross L, Duncan RP, Fujie M, Tanaka M, Satoh N, Bachtrog D, Wilson ACC, et al. 2013. Horizontal Gene Transfer from Diverse Bacteria to an Insect Genome Enables a Tripartite Nested Mealybug Symbiosis. *Cell* 153:1567–1578.

Huson DH, Beier S, Flade I, Górska A, El-Hadidi M, Mitra S, Ruscheweyh H-J, Tappu R. 2016. MEGAN Community Edition - Interactive Exploration and Analysis of Large-Scale Microbiome Sequencing Data.Poisot T, editor. *PLOS Comput. Biol.* 12:e1004957.

Ip JC-H, Xu T, Sun J, Li R, Chen C, Lan Y, Han Z, Zhang H, Wei J, Wang H, et al. 2021. Host-Endosymbiont Genome Integration in a Deep-Sea Chemosymbiotic Clam. *Mol. Biol. Evol.* [Internet]. Available from: https://academic.oup.com/mbe/advance-article/doi/10.1093/molbev/msaa241/5909661

Janssens S, Beyaert R. 2003. Role of Toll-Like Receptors in Pathogen Recognition. *Clin. Microbiol. Rev.* 16:637–646.

Johnson KS, Childress JJ, Hessler RR, Sakamoto-Arnold CM, Beehler CL. 1988. Chemical and biological interactions in the Rose Garden hydrothermal vent field, Galapagos spreading center. *Deep Sea Res. Part Oceanogr. Res. Pap.* 35:1723–1744.

Jones ML. 1981. Riftia pachyptila Jones: Observations on the Vestimentiferan Worm from the Galápagos Rift. *Science* 213:333–336.

Jones ML, Gardiner SL. 1989. On the Early Development of the Vestimentiferan Tube Worm Ridgeia sp. and Observations on the Nervous System and Trophosome of Ridgeia sp. and Riftia pachyptila. *Biol. Bull.* 177:254–276.

Jones P, Binns D, Chang H-Y, Fraser M, Li W, McAnulla C, McWilliam H, Maslen J, Mitchell A, Nuka G, et al. 2014. InterProScan 5: genome-scale protein function classification. *Bioinformatics* 30:1236–1240.

Julien O, Wells JA. 2017. Caspases and their substrates. *Cell Death Differ.* 24:1380–1389.

Kabeya N, Fonseca MM, Ferrier DEK, Navarro JC, Bay LK, Francis DS, Tocher DR, Castro LFC, Monroig Ó. 2018. Genes for de novo biosynthesis of omega-3 polyunsaturated fatty acids are widespread in animals. *Sci. Adv.* 4:eaar6849.

Kalyaanamoorthy S, Minh BQ, Wong TKF, von Haeseler A, Jermiin LS. 2017. ModelFinder: fast model selection for accurate phylogenetic estimates. *Nat. Methods* 14:587–589.

Kang D, Huang F, Li D, Shankland M, Gaffield W, Weisblat DA. 2003. A hedgehog homolog regulates gut formation in leech (Helobdella). *Development* 130:1645–1657.

Katoh K, Standley DM. 2013. MAFFT Multiple Sequence Alignment Software Version 7: Improvements in Performance and Usability. *Mol. Biol. Evol.* 30:772–780.

Kawai T, Akira S. 2006. TLR signaling. *Cell Death Differ.* 13:816–825.

Kawasaki T, Kawai T. 2014. Toll-Like Receptor Signaling Pathways. *Front. Immunol.* [Internet] 5. Available from: https://www.ncbi.nlm.nih.gov/pmc/articles/PMC4174766/

Kim JK, Han SH, Kim C-H, Jo YH, Futahashi R, Kikuchi Y, Fukatsu T, Lee BL. 2014. Molting-associated suppression of symbiont population and up-regulation of antimicrobial activity in the midgut symbiotic organ of the Riptortus–Burkholderia symbiosis. *Dev. Comp. Immunol.* 43:10–14.

Kirkitadze MD, Barlow PN. 2001. Structure and flexibility of the multiple domain proteins that regulate complement activation. *Immunol. Rev.* 180:146–161.

Klionsky DJ. 2012. Look people, “Atg” is an abbreviation for “autophagy-related.” That’s it. *Autophagy* 8:1281–1282.

Kolmogorov M, Yuan J, Lin Y, Pevzner PA. 2019. Assembly of long, error-prone reads using repeat graphs. *Nat. Biotechnol.* 37:540–546.

Koren S, Walenz BP, Berlin K, Miller JR, Bergman NH, Phillippy AM. 2017. Canu: scalable and accurate long-read assembly via adaptive *k* -mer weighting and repeat separation. *Genome Res.* 27:722–736.

Kořený L, Oborník M, Lukeš J. 2013. Make It, Take It, or Leave It: Heme Metabolism of Parasites. *PLoS Pathog.* [Internet] 9. Available from: https://www.ncbi.nlm.nih.gov/pmc/articles/PMC3547853/

Kotani T, Kirisako H, Koizumi M, Ohsumi Y, Nakatogawa H. 2018. The Atg2-Atg18 complex tethers pre-autophagosomal membranes to the endoplasmic reticulum for autophagosome formation. *Proc. Natl. Acad. Sci. U. S. A.* 115:10363–10368.

Kovaka S, Zimin AV, Pertea GM, Razaghi R, Salzberg SL, Pertea M. 2019. Transcriptome assembly from long-read RNA-seq alignments with StringTie2. *Genome Biol.* 20:278.

Kryuchkova-Mostacci N, Robinson-Rechavi M. 2016. A benchmark of gene expression tissue-specificity metrics. *Brief. Bioinform.*:bbw008.

Krzywinski M, Schein J, Birol İ, Connors J, Gascoyne R, Horsman D, Jones SJ, Marra MA. 2009. Circos: An information aesthetic for comparative genomics. *Genome Res.* 19:1639–1645.

Kück P, Meusemann K. 2010. FASconCAT: Convenient handling of data matrices. *Mol. Phylogenet. Evol.* 56:1115–1118.

Kulakova M, Bakalenko N, Novikova E, Cook CE, Eliseeva E, Steinmetz PRH, Kostyuchenko RP, Dondua A, Arendt D, Akam M, et al. 2007. Hox gene expression in larval development of the polychaetes Nereis virens and Platynereis dumerilii (Annelida, Lophotrochozoa). *Dev. Genes Evol.* 217:39–54.

Laetsch DR, Blaxter ML. 2017. BlobTools: Interrogation of genome assemblies. *F1000Research* 6:1287.

Langmead B, Salzberg SL. 2012. Fast gapped-read alignment with Bowtie 2. *Nat. Methods* 9:357–359.

Lartillot N, Philippe H. 2006. Computing Bayes Factors Using Thermodynamic Integration.Lewis P, editor. *Syst. Biol.* 55:195–207.

Li H. 2018. Minimap2: pairwise alignment for nucleotide sequences.Birol I, editor. *Bioinformatics* 34:3094–3100.

Li H, Handsaker B, Wysoker A, Fennell T, Ruan J, Homer N, Marth G, Abecasis G, Durbin R, 1000 Genome Project Data Processing Subgroup. 2009. The Sequence Alignment/Map format and SAMtools. *Bioinformatics* 25:2078–2079.

Li Y, Huang T-T, Carlson EJ, Melov S, Ursell PC, Olson JL, Noble LJ, Yoshimura MP, Berger C, Chan PH, et al. 1995. Dilated cardiomyopathy and neonatal lethality in mutant mice lacking manganese superoxide dismutase. *Nat. Genet.* 11:376–381.

Li Y, Tassia MG, Waits DS, Bogantes VE, David KT, Halanych KM. 2019. Genomic adaptations to chemosymbiosis in the deep-sea seep-dwelling tubeworm Lamellibrachia luymesi. *BMC Biol.* 17:91.

Lilley MD, Butterfield DA, Olson EJ, Lupton JE, Macko SA, McDuff RE. 1993. Anomalous CH 4 and NH 4 + concentrations at an unsedimented mid-ocean-ridge hydrothermal system. *Nature* 364:45–47.

Lim S, Kaldis P. 2013. Cdks, cyclins and CKIs: roles beyond cell cycle regulation. *Development* 140:3079–3093.

Liu G, Kaw B, Kurfis J, Rahmanuddin S, Kanwar YS, Chugh SS. 2003. Neph1 and nephrin interaction in the slit diaphragm is an important determinant of glomerular permeability. *J. Clin. Invest.* 112:209–221.

Liu G, Zhang Huanxin, Zhao C, Zhang Honghai. 2020. Evolutionary History of the Toll-Like Receptor Gene Family across Vertebrates.Enard D, editor. *Genome Biol. Evol.* 12:3615–3634.

Liu H, Wang H, Cai S, Zhang H. 2017. A Novel ω3-Desaturase in the Deep Sea Giant Tubeworm Riftia pachyptila. *Mar. Biotechnol.* 19:345–350.

Liu W, Xie Y, Ma J, Luo X, Nie P, Zuo Z, Lahrmann U, Zhao Q, Zheng Y, Zhao Y, et al. 2015. IBS: an illustrator for the presentation and visualization of biological sequences. *Bioinformatics* 31:3359–3361.

Lomsadze A, Burns PD, Borodovsky M. 2014. Integration of mapped RNA-Seq reads into automatic training of eukaryotic gene finding algorithm. *Nucleic Acids Res.* 42:e119–e119.

Lowe TM, Chan PP. 2016. tRNAscan-SE On-line: integrating search and context for analysis of transfer RNA genes. *Nucleic Acids Res.* 44:W54–W57.

Luo Y-J, Kanda M, Koyanagi R, Hisata K, Akiyama T, Sakamoto H, Sakamoto T, Satoh N. 2018. Nemertean and phoronid genomes reveal lophotrochozoan evolution and the origin of bilaterian heads. *Nat. Ecol. Evol.* 2:141–151.

Luo Y-J, Takeuchi T, Koyanagi R, Yamada L, Kanda M, Khalturina M, Fujie M, Yamasaki S, Endo K, Satoh N. 2015. The Lingula genome provides insights into brachiopod evolution and the origin of phosphate biomineralization. *Nat. Commun.* 6:8301.

Lutz RA, Shank TM, Fornari DJ, Haymon RM, Lilley MD, Von Damm KL, Desbruyeres D. 1994. Rapid growth at deep-sea vents. *Nature* 371:663–664.

Malik Z, Djaldetti M. 1979. 5-aminolevulinic acid stimulation of porphyrin and hemoglobin synthesis by uninduced friend erythroleukemic cells. *Cell Differ.* 8:223–233.

Malumbres M. 2014. Cyclin-dependent kinases. *Genome Biol.* 15:122.

Man SM, Kanneganti T-D. 2016. Regulation of lysosomal dynamics and autophagy by CTSB/cathepsin B. *Autophagy* 12:2504–2505.

Mariño G, Niso-Santano M, Baehrecke EH, Kroemer G. 2014. Self-consumption: the interplay of autophagy and apoptosis. *Nat. Rev. Mol. Cell Biol.* 15:81–94.

Massagué J. 2012. TGFβ signalling in context. *Nat. Rev. Mol. Cell Biol.* 13:616–630.

Mayle KM, Le AM, Kamei DT. 2012. The Intracellular Trafficking Pathway of Transferrin. *Biochim. Biophys. Acta* 1820:264–281.

McClain CR, Allen AP, Tittensor DP, Rex MA. 2012. Energetics of life on the deep seafloor. *Proc. Natl. Acad. Sci.* 109:15366–15371.

Mei Q, Huang J, Chen W, Tang J, Xu C, Yu Q, Cheng Y, Ma L, Yu X, Li S. 2017. Regulation of DNA replication-coupled histone gene expression. *Oncotarget* 8:95005–95022.

Mi H, Huang X, Muruganujan A, Tang H, Mills C, Kang D, Thomas PD. 2017. PANTHER version 11: expanded annotation data from Gene Ontology and Reactome pathways, and data analysis tool enhancements. *Nucleic Acids Res.* 45:D183–D189.

Minic Z, Hervé G. 2003. Arginine Metabolism in the Deep Sea Tube Worm *Riftia pachyptila* and Its Bacterial Endosymbiont. *J. Biol. Chem.* 278:40527–40533.

Minic Z, Hervé G. 2004. Biochemical and enzymological aspects of the symbiosis between the deep-sea tubeworm Riftia pachyptila and its bacterial endosymbiont: Riftia pachyptila and its bacterial endosymbiont. *Eur. J. Biochem.* 271:3093–3102.

Minic Z, Pastra-Landis S, Gaill F, Hervé G. 2002. Catabolism of Pyrimidine Nucleotides in the Deep-sea Tube Worm *Riftia pachyptila*. *J. Biol. Chem.* 277:127–134.

Minic Z, Simon V, Penverne B, Gaill F, Hervé G. 2001. Contribution of the Bacterial Endosymbiont to the Biosynthesis of Pyrimidine Nucleotides in the Deep-sea Tube Worm *Riftia pachyptila*. *J. Biol. Chem.* 276:23777–23784.

Mistry J, Finn RD, Eddy SR, Bateman A, Punta M. 2013. Challenges in homology search: HMMER3 and convergent evolution of coiled-coil regions. *Nucleic Acids Res.* 41:e121–e121.

Mizushima N, Komatsu M. 2011. Autophagy: Renovation of Cells and Tissues. *Cell* 147:728–741.

Monné M, Miniero DV, Daddabbo L, Palmieri L, Porcelli V, Palmieri F. 2015. Mitochondrial transporters for ornithine and related amino acids: a review. *Amino Acids* 47:1763–1777.

Moustakas A, Heldin C-H. 2009. The regulation of TGFβ signal transduction. *Development* 136:3699–3714.

Mu FT, Callaghan JM, Steele-Mortimer O, Stenmark H, Parton RG, Campbell PL, McCluskey J, Yeo JP, Tock EP, Toh BH. 1995. EEA1, an early endosome-associated protein. EEA1 is a conserved alpha-helical peripheral membrane protein flanked by cysteine “fingers” and contains a calmodulin-binding IQ motif. *J. Biol. Chem.* 270:13503–13511.

Nakabachi A, Shigenobu S, Sakazume N, Shiraki T, Hayashizaki Y, Carninci P, Ishikawa H, Kudo T, Fukatsu T. 2005. Transcriptome analysis of the aphid bacteriocyte, the symbiotic host cell that harbors an endocellular mutualistic bacterium, Buchnera. *Proc. Natl. Acad. Sci.* 102:5477–5482.

Nakahama S, Nakagawa T, Kanemori M, Fukumori Y, Sasayama Y. 2008. Direct evidence that extracellular giant hemoglobin is produced in chloragogen tissues in a beard worm, Oligobrachia mashikoi (Frenulata, Siboglinidae, Annelida). *Zoolog. Sci.* 25:1247–1252.

Nakao A, Imamura T, Souchelnytskyi S, Kawabata M, Ishisaki A, Oeda E, Tamaki K, Hanai J, Heldin CH, Miyazono K, et al. 1997. TGF-beta receptor-mediated signalling through Smad2, Smad3 and Smad4. *EMBO J.* 16:5353–5362.

Nawrocki EP, Eddy SR. 2013. Infernal 1.1: 100-fold faster RNA homology searches. *Bioinformatics* 29:2933–2935.

Nguyen L-T, Schmidt HA, von Haeseler A, Minh BQ. 2015. IQ-TREE: A Fast and Effective Stochastic Algorithm for Estimating Maximum-Likelihood Phylogenies. *Mol. Biol. Evol.* 32:268–274.

Niehrs C. 2012. The complex world of WNT receptor signalling. *Nat. Rev. Mol. Cell Biol.* 13:767–779.

Nikoh N, Nakabachi A. 2009. Aphids acquired symbiotic genes via lateral gene transfer. *BMC Biol.* 7:12.

Nishikori K, Morioka K, Kubo T, Morioka M. 2009. Age- and morph-dependent activation of the lysosomal system and Buchnera degradation in aphid endosymbiosis. *J. Insect Physiol.* 55:351–357.

Nowell RW, Almeida P, Wilson CG, Smith TP, Fontaneto D, Crisp A, Micklem G, Tunnacliffe A, Boschetti C, Barraclough TG. 2018. Comparative genomics of bdelloid rotifers: Insights from desiccating and nondesiccating species.Tyler-Smith C, editor. *PLOS Biol.* 16:e2004830.

Nussbaumer AD, Fisher CR, Bright M. 2006. Horizontal endosymbiont transmission in hydrothermal vent tubeworms. *Nature* 441:345–348.

Nyholm SV, Graf J. 2012. Knowing your friends: invertebrate innate immunity fosters beneficial bacterial symbioses. *Nat. Rev. Microbiol.* 10:815–827.

Ohsumi Y. 2014. Historical landmarks of autophagy research. *Cell Res.* 24:9–23.

Oka S, Hirai J, Yasukawa T, Nakahara Y, Inoue YH. 2015. A correlation of reactive oxygen species accumulation by depletion of superoxide dismutases with age-dependent impairment in the nervous system and muscles of Drosophila adults. *Biogerontology* 16:485–501.

Osawa T, Kotani T, Kawaoka T, Hirata E, Suzuki K, Nakatogawa H, Ohsumi Y, Noda NN. 2019. Atg2 mediates direct lipid transfer between membranes for autophagosome formation. *Nat. Struct. Mol. Biol.* 26:281–288.

Park HH, Lo Y-C, Lin S-C, Wang L, Yang JK, Wu H. 2007. The Death Domain Superfamily in Intracellular Signaling of Apoptosis and Inflammation. *Annu. Rev. Immunol.* 25:561–586.

Park IY, Park CB, Kim MS, Kim SC. 1998. Parasin I, an antimicrobial peptide derived from histone H2A in the catfish, Parasilurus asotus. *FEBS Lett.* 437:258–262.

Parks DH, Imelfort M, Skennerton CT, Hugenholtz P, Tyson GW. 2015. CheckM: assessing the quality of microbial genomes recovered from isolates, single cells, and metagenomes. *Genome Res.* 25:1043–1055.

Paul S, Arumugaperumal A, Rathy R, Ponesakki V, Arunachalam P, Sivasubramaniam S. 2018. Data on genome annotation and analysis of earthworm Eisenia fetida. *Data Brief* 20:525–534.

Pegg AE. 2016. Functions of Polyamines in Mammals. *J. Biol. Chem.* 291:14904–14912.

Pflugfelder B, Cary SC, Bright M. 2009. Dynamics of cell proliferation and apoptosis reflect different life strategies in hydrothermal vent and cold seep vestimentiferan tubeworms. *Cell Tissue Res.* 337:149–165.

Pflugfelder B, Fisher CR, Bright M. 2005. The color of the trophosome: elemental sulfur distribution in the endosymbionts of Riftia pachyptila (Vestimentifera; Siboglinidae). *Mar. Biol.* 146:895–901.

Phleger CF, Nelson MM, Groce AK, Cary SC, Coyne KJ, Nichols PD. 2005. Lipid composition of deep-sea hydrothermal vent tubeworm Riftia pachyptila, crabs Munidopsis subsquamosa and Bythograea thermydron, mussels Bathymodiolus sp. and limpets Lepetodrilus spp. *Comp. Biochem. Physiol. B Biochem. Mol. Biol.* 141:196–210.

Picot S, Faury N, Arzul I, Chollet B, Renault T, Morga B. 2020. Identification of the autophagy pathway in a mollusk bivalve, Crassostrea gigas. *Autophagy*:1–19.

Pita L, Hoeppner MP, Ribes M, Hentschel U. 2018. Differential expression of immune receptors in two marine sponges upon exposure to microbial-associated molecular patterns. *Sci. Rep.* 8:16081.

Pond SLK, Frost SDW, Muse SV. 2005. HyPhy: hypothesis testing using phylogenies. *Bioinformatics* 21:676–679.

Price AL, Jones NC, Pevzner PA. 2005. De novo identification of repeat families in large genomes. *Bioinformatics* 21:i351–i358.

Price MN, Dehal PS, Arkin AP. 2010. FastTree 2 – Approximately Maximum-Likelihood Trees for Large Alignments.Poon AFY, editor. *PLoS ONE* 5:e9490.

Rahban R, Nef S. 2020. CatSper: The complex main gate of calcium entry in mammalian spermatozoa. *Mol. Cell. Endocrinol.* 518:110951.

Ravaux J, Chamoy L, Shillito B. 2000. Synthesis and maturation processes in the exoskeleton of the vent worm Riftia pachyptila. *Mar. Biol.* 136:505–512.

Ravaux J, Zbinden M, Voss-Foucart MF, Compère P, Goffinet G, Gaill F. 2003. Comparative degradation rates of chitinous exoskeletons from deep-sea environments. *Mar. Biol.* 143:405–412.

Ridgway ID, Richardson CA. 2011. Arctica islandica: the longest lived non colonial animal known to science. *Rev. Fish Biol. Fish.* 21:297–310.

Rimskaya-Korsakova N, Dyachuk V, Temereva E. 2020. Parapodial glandular organs in Owenia borealis (Annelida: Oweniidae) and their possible relationship with nephridia. *J. Exp. Zoolog. B Mol. Dev. Evol.* 334:88–99.

Rimskaya-Korsakova NN, Karaseva NP, Temereva EN, Malakhov VV. 2018. Protonephridial Excretory System in Vestimentifera (Siboglinidae, Annelida). *Dokl. Biol. Sci.* 478:22–25.

Ritschard EA, Fitak RR, Simakov O, Johnsen S. 2019. Genomic signatures of G-protein-coupled receptor expansions reveal functional transitions in the evolution of cephalopod signal transduction. *Proc. R. Soc. B Biol. Sci.* [Internet] 286. Available from: https://www.ncbi.nlm.nih.gov/pmc/articles/PMC6408891/

Rivera AS, Gonsalves FC, Song MH, Norris BJ, Weisblat DA. 2005. Characterization of Notch-class gene expression in segmentation stem cells and segment founder cells in Helobdella robusta (Lophotrochozoa; Annelida; Clitellata; Hirudinida; Glossiphoniidae). *Evol. Dev.* 7:588–599.

Robertson G, Schein J, Chiu R, Corbett R, Field M, Jackman SD, Mungall K, Lee S, Okada HM, Qian JQ, et al. 2010. De novo assembly and analysis of RNA-seq data. *Nat. Methods* 7:909–912.

Rosa R de, Prud’homme B, Balavoine G. 2005. caudal and even-skipped in the annelid Platynereis dumerilii and the ancestry of posterior growth. *Evol. Dev.* 7:574–587.

Rouse GW, Wilson NG, Worsaae K, Vrijenhoek RC. 2015. A Dwarf Male Reversal in Bone-Eating Worms. *Curr. Biol.* 25:236–241.

Ruppert EE, Smith PR. 1988. The Functional Organization of Filtration Nephridia. *Biol. Rev.* 63:231–258.

Samanta D, Datta PK. 2012. Alterations in the Smad pathway in human cancers. *Front. Biosci. Landmark Ed.* 17:1281–1293.

Schmitz JF, Zimmer F, Bornberg-Bauer E. 2016. Mechanisms of transcription factor evolution in Metazoa. *Nucleic Acids Res.* 44:6287–6297.

Schulze A. 2001. Comparative anatomy of excretory organs in vestimentiferan tube worms (Pogonophora, Obturata). *J. Morphol.* 250:1–11.

Seaver EC, Kaneshige LM. 2006. Expression of ‘segmentation’ genes during larval and juvenile development in the polychaetes Capitella sp. I and H. elegans. *Dev. Biol.* 289:179–194.

Sentman M-L, Granström M, Jakobson H, Reaume A, Basu S, Marklund SL. 2006. Phenotypes of Mice Lacking Extracellular Superoxide Dismutase and Copper- and Zinc-containing Superoxide Dismutase *. *J. Biol. Chem.* 281:6904–6909.

Sheaffer KL, Updike DL, Mango SE. 2008. The Target of Rapamycin pathway antagonizes pha-4/FoxA to control development and aging. *Curr. Biol. CB* 18:1355–1364.

Shillito B, Lechaire JP, Gaill F. 1993. Microvilli-like Structures Secreting Chitin Crystallites. *J. Struct. Biol.* 111:59–67.

Shillito B, Lübbering B, Lechaire J-P, Childress JJ, Gaill F. 1995. Chitin Localization in the Tube Secretion System of a Repressurized Deep-Sea Tube Worm. *J. Struct. Biol.* 114:67–75.

Shpilka T, Weidberg H, Pietrokovski S, Elazar Z. 2011. Atg8: an autophagy-related ubiquitin-like protein family. *Genome Biol.* 12:226.

Sibuet M, Olu K. 1998. Biogeography, biodiversity and fluid dependence of deep-sea cold-seep communities at active and passive margins. *Deep Sea Res. Part II Top. Stud. Oceanogr.* 45:517–567.

Silke J, Meier P. 2013. Inhibitor of Apoptosis (IAP) Proteins-Modulators of Cell Death and Inflammation. *Cold Spring Harb. Perspect. Biol.* 5:a008730–a008730.

Simakov O, Kawashima T, Marlétaz F, Jenkins J, Koyanagi R, Mitros T, Hisata K, Bredeson J, Shoguchi E, Gyoja F, et al. 2015. Hemichordate genomes and deuterostome origins. *Nature* 527:459–465.

Simakov O, Marletaz F, Cho S-J, Edsinger-Gonzales E, Havlak P, Hellsten U, Kuo D-H, Larsson T, Lv J, Arendt D, et al. 2013. Insights into bilaterian evolution from three spiralian genomes. *Nature* 493:526–531.

Simão FA, Waterhouse RM, Ioannidis P, Kriventseva EV, Zdobnov EM. 2015. BUSCO: assessing genome assembly and annotation completeness with single-copy orthologs. *Bioinformatics* 31:3210–3212.

Sinensky M. 1974. Homeoviscous Adaptation—A Homeostatic Process that Regulates the Viscosity of Membrane Lipids in Escherichia coli. *Proc. Natl. Acad. Sci. U. S. A.* 71:522–525.

Sloan DB, Nakabachi A, Richards S, Qu J, Murali SC, Gibbs RA, Moran NA. 2014. Parallel Histories of Horizontal Gene Transfer Facilitated Extreme Reduction of Endosymbiont Genomes in Sap-Feeding Insects. *Mol. Biol. Evol.* 31:857–871.

Smart TI, Von Dassow G. 2009. Unusual Development of the Mitraria Larva in the Polychaete Owenia collaris. *Biol. Bull.* 217:253–268.

Smith MD, Wertheim JO, Weaver S, Murrell B, Scheffler K, Kosakovsky Pond SL. 2015. Less Is More: An Adaptive Branch-Site Random Effects Model for Efficient Detection of Episodic Diversifying Selection. *Mol. Biol. Evol.* 32:1342–1353.

Smith PR, Ruppert EE, Gardiner SL. 1987. A deuterostome-like nephridium in the mitraria larva of owenia fusiformis (polychaeta, annelida). *Biol. Bull.* 172:315–323.

Song X, Jin P, Qin S, Chen L, Ma F. 2012. The Evolution and Origin of Animal Toll-Like Receptor Signaling Pathway Revealed by Network-Level Molecular Evolutionary Analyses. *PLoS ONE* [Internet] 7. Available from: https://www.ncbi.nlm.nih.gov/pmc/articles/PMC3517549/

Southward EC. 1999. Development of Perviata and Vestimentifera (Pogonophora). *Hydrobiologia* 402:185–202.

Southward EC, Schulze A, Gardiner SL. 2005. Pogonophora (Annelida): form and function. In: Bartolomaeus T, Purschke G, editors. Morphology, Molecules, Evolution and Phylogeny in Polychaeta and Related Taxa. Developments in Hydrobiology. Dordrecht: Springer Netherlands. p. 227–251. Available from: https://doi.org/10.1007/1-4020-3240-4_13

Stanke M, Morgenstern B. 2005. AUGUSTUS: a web server for gene prediction in eukaryotes that allows user-defined constraints. *Nucleic Acids Res.* 33:W465–W467.

Stenmark H. 2009. Rab GTPases as coordinators of vesicle traffic. *Nat. Rev. Mol. Cell Biol.* 10:513–525.

Sun J, Chen C, Miyamoto N, Li R, Sigwart JD, Xu T, Sun Y, Wong WC, Ip JCH, Zhang W, et al. 2020. The Scaly-foot Snail genome and implications for the origins of biomineralised armour. *Nat. Commun.* 11:1657.

Sun J, Zhang Yu, Xu T, Zhang Yang, Mu H, Zhang Yanjie, Lan Y, Fields CJ, Hui JHL, Zhang W, et al. 2017. Adaptation to deep-sea chemosynthetic environments as revealed by mussel genomes. *Nat. Ecol. Evol.* 1:0121.

Sun Y, Sun J, Yang Y, Lan Y, Ip JC-H, Wong WC, Kwan YH, Zhang Y, Han Z, Qiu J-W, et al. 2021. Genomic Signatures Supporting the Symbiosis and Formation of Chitinous Tube in the Deep-Sea Tubeworm Paraescarpia echinospica. *Mol. Biol. Evol.* 38:4116–4134.

Suyama M, Torrents D, Bork P. 2006. PAL2NAL: robust conversion of protein sequence alignments into the corresponding codon alignments. *Nucleic Acids Res.* 34:W609–W612.

Suzuki T, Uda K, Adachi M, Sanada H, Tanaka K, Mizuta C, Ishida K, Ellington WR. 2009. Evolution of the diverse array of phosphagen systems present in annelids. *Comp. Biochem. Physiol. B Biochem. Mol. Biol.* 152:60–66.

Swigoňová Z, Mohsen A-W, Vockley J. 2009. Acyl-CoA Dehydrogenases: Dynamic History of Protein Family Evolution. *J. Mol. Evol.* 69:176–193.

Tamura N, Nishimura T, Sakamaki Y, Koyama‐Honda I, Yamamoto H, Mizushima N. 2017. Differential requirement for ATG2A domains for localization to autophagic membranes and lipid droplets. *FEBS Lett.* 591:3819–3830.

Thamm K, Seaver EC. 2008. Notch signaling during larval and juvenile development in the polychaete annelid Capitella sp. I. *Dev. Biol.* 320:304–318.

Thiel D, Hugenschütt M, Meyer H, Paululat A, Quijada-Rodriguez AR, Purschke G, Weihrauch D. 2016. Ammonia excretion in the marine polychaete *Eurythoe complanata* (Annelida). *J. Exp. Biol.*:jeb.145615.

Thielens NM, Tedesco F, Bohlson SS, Gaboriaud C, Tenner AJ. 2017. C1q: A fresh look upon an old molecule. *Mol. Immunol.* 89:73–83.

Thomas PD. 2003. PANTHER: A Library of Protein Families and Subfamilies Indexed by Function. *Genome Res.* 13:2129–2141.

Tillich M, Lehwark P, Pellizzer T, Ulbricht-Jones ES, Fischer A, Bock R, Greiner S. 2017. GeSeq - versatile and accurate annotation of organelle genomes. *Nucleic Acids Res.* 45:W6–W11.

Tsai IJ, Zarowiecki M, Holroyd N, Garciarrubio A, Sanchez-Flores A, Brooks KL, Tracey A, Bobes RJ, Fragoso G, Sciutto E, et al. 2013. The genomes of four tapeworm species reveal adaptations to parasitism. *Nature* 496:57–63.

Uda K, Fujimoto N, Akiyama Y, Mizuta K, Tanaka K, Ellington WR, Suzuki T. 2006. Evolution of the arginine kinase gene family. *Comp. Biochem. Physiol. Part D Genomics Proteomics* 1:209–218.

Uda K, Tanaka K, Bailly X, Zal F, Suzuki T. 2005. Phosphagen kinase of the giant tubeworm Riftia pachyptila: Cloning and expression of cytoplasmic and mitochondrial isoforms of taurocyamine kinase. *Int. J. Biol. Macromol.* 37:54–60.

Uno M, Nishida E. 2016. Lifespan-regulating genes in C. elegans. *Npj Aging Mech. Dis.* 2:16010.

Vaquerizas JM, Kummerfeld SK, Teichmann SA, Luscombe NM. 2009. A census of human transcription factors: function, expression and evolution. *Nat. Rev. Genet.* 10:252–263.

Velikkakath AKG, Nishimura T, Oita E, Ishihara N, Mizushima N. 2012. Mammalian Atg2 proteins are essential for autophagosome formation and important for regulation of size and distribution of lipid droplets. *Mol. Biol. Cell* 23:896–909.

Vigneron A, Masson F, Vallier A, Balmand S, Rey M, Vincent-Monégat C, Aksoy E, Aubailly-Giraud E, Zaidman-Rémy A, Heddi A. 2014. Insects Recycle Endosymbionts when the Benefit Is Over. *Curr. Biol.* 24:2267–2273.

Waits DS, Santos SR, Thornhill DJ, Li Y, Halanych KM. 2016. Evolution of Sulfur Binding by Hemoglobin in Siboglinidae (Annelida) with Special Reference to Bone-Eating Worms, Osedax. *J. Mol. Evol.* 82:219–229.

Wallis JG, Watts JL, Browse J. 2002. Polyunsaturated fatty acid synthesis: what will they think of next? *Trends Biochem. Sci.* 27:467–473.

Wang D, Zhang Y, Zhang Z, Zhu J, Yu J. 2010. KaKs_Calculator 2.0: A Toolkit Incorporating Gamma-Series Methods and Sliding Window Strategies. *Genomics Proteomics Bioinformatics* 8:77–80.

Wang S, Zhang J, Jiao W, Li J, Xun X, Sun Y, Guo X, Huan P, Dong B, Zhang L, et al. 2017. Scallop genome provides insights into evolution of bilaterian karyotype and development. *Nat. Ecol. Evol.* 1:1–12.

Weihrauch D, Allen GJP. 2018. Ammonia excretion in aquatic invertebrates: new insights and questions. *J. Exp. Biol.* [Internet] 221. Available from: https://doi.org/10.1242/jeb.169219

Wilmot DB, Vetter RD. 1990. The bacterial symbiont from the hydrothermal vent tubewormRiftia pachyptila is a sulfide specialist. *Mar. Biol.* 106:273–283.

Wohlrab H. 2006. The human mitochondrial transport/carrier protein family. Nonsynonymous single nucleotide polymorphisms (nsSNPs) and mutations that lead to human diseases. *Biochim. Biophys. Acta* 1757:1263–1270.

Wong C-O, Gregory S, Hu H, Chao Y, Sepúlveda VE, He Y, Li-Kroeger D, Goldman WE, Bellen HJ, Venkatachalam K. 2017. Lysosomal Degradation is Required for Sustained Phagocytosis of Bacteria by Macrophages. *Cell Host Microbe* 21:719-730.e6.

Wong P-M, Puente C, Ganley IG, Jiang X. 2013. The ULK1 complex. *Autophagy* 9:124–137.

Wyatt K, White HE, Wang L, Bateman OA, Slingsby C, Orlova EV, Wistow G. 2006. Lengsin is a survivor of an ancient family of class I glutamine synthetases in eukaryotes that has undergone evolutionary re-engineering for a role in the vertebrate eye lens. *Struct. Lond. Engl. 1993* 14:1823–1834.

Xue Q, Hellberg ME, Schey KL, Itoh N, Eytan RI, Cooper RK, La Peyre JF. 2010. A new lysozyme from the eastern oyster, Crassostrea virginica, and a possible evolutionary pathway for i-type lysozymes in bivalves from host defense to digestion. *BMC Evol. Biol.* 10:213.

Yanai I, Benjamin H, Shmoish M, Chalifa-Caspi V, Shklar M, Ophir R, Bar-Even A, Horn-Saban S, Safran M, Domany E, et al. 2005. Genome-wide midrange transcription profiles reveal expression level relationships in human tissue specification. *Bioinformatics* 21:650–659.

Ye X, Ji C, Zhou C, Zeng L, Gu S, Ying K, Xie Y, Mao Y. 2004. Cloning and characterization of a human cDNA ACAD10 mapped to chromosome 12q24.1. *Mol. Biol. Rep.* 31:191–195.

Ye X, Li M, Hou T, Gao T, Zhu W, Yang Y. 2016. Sirtuins in glucose and lipid metabolism. *Oncotarget* 8:1845–1859.

Young CM, Vázquez E, Metaxas A, Tyler PA. 1996. Embryology of vestimentiferan tube worms from deep-sea methane/sulphide seeps. *Nature* 381:514–516.

Zakrzewski A-C, Weigert A, Helm C, Adamski M, Adamska M, Bleidorn C, Raible F, Hausen H. 2014. Early Divergence, Broad Distribution, and High Diversity of Animal Chitin Synthases. *Genome Biol. Evol.* 6:316–325.

Zal F, Lallier FH, Green BN, Vinogradov SN, Toulmond A. 1996. The Multi-hemoglobin System of the Hydrothermal Vent Tube Worm Riftia pachyptila II. COMPLETE POLYPEPTIDE CHAIN COMPOSITION INVESTIGATED BY MAXIMUM ENTROPY ANALYSIS OF MASS SPECTRA. *J. Biol. Chem.* 271:8875–8881.

Zal F, Suzuki T, Kawasaki Y, Childress JJ, Lallier FH, Toulmond A. 1997. Primary structure of the common polypeptide chain b from the multi-hemoglobin system of the hydrothermal vent tube worm Riftia pachyptila: An insight on the sulfide binding-site. *Proteins Struct. Funct. Bioinforma.* 29:562–574.

Zhang G, Fang X, Guo X, Li L, Luo R, Xu F, Yang P, Zhang L, Wang X, Qi H, et al. 2012. The oyster genome reveals stress adaptation and complexity of shell formation. *Nature* 490:49–54.

Zhang J, Sun Q, Luan Z, Lian C, Sun L. 2017. Comparative transcriptome analysis of Rimicaris sp. reveals novel molecular features associated with survival in deep-sea hydrothermal vent. *Sci. Rep.* 7:2000.

Zhang Z, Xiao J, Wu J, Zhang H, Liu G, Wang X, Dai L. 2012. ParaAT: A parallel tool for constructing multiple protein-coding DNA alignments. *Biochem. Biophys. Res. Commun.* 419:779–781.

Zhao M, Mishra L, Deng C-X. 2018. The role of TGF-β/SMAD4 signaling in cancer. *Int. J. Biol. Sci.* 14:111–123.

Zhou C, Ma K, Gao R, Mu C, Chen L, Liu Q, Luo Q, Feng D, Zhu Y, Chen Q. 2017. Regulation of mATG9 trafficking by Src- and ULK1-mediated phosphorylation in basal and starvation-induced autophagy. *Cell Res.* 27:184–201.

Zwarycz AS, Nossa CW, Putnam NH, Ryan JF. 2016. Timing and Scope of Genomic Expansion within Annelida: Evidence from Homeoboxes in the Genome of the Earthworm *Eisenia fetida*. *Genome Biol. Evol.* 8:271–281.

# **LIST OF BIOINFORMATIC COMMANDS**

**1 GENOME PIPELINE**

**1.1 Pre-processing of databases**

**1.1.1 Converting native BAM PacBio files to fastq**

- bam2fastq -c 9 -o PacBio_library.fastq.gz PacBio_library.bam

**1.1.2 Removal of endosymbiont and tubeworm mitochondrial reads**

- minimap2 -ax map-pb Endoriftia_plus_Riftia_mito.fasta PacBio_library.fastq.gz | samtools sort -o PacBio_library_mapped_against_contaminants.bam

**1.1.3 Extraction of unmapped reads from BAM results**

- samtools view -f 4 PacBio_library_mapped_against_contaminants.bam > PacBio_library_mapped_against_contaminants_unmapped_reads.bam
- bam2fastq -c 9 -o PacBio_library_mapped_against_contaminants_unmapped_reads.fastq.gz PacBio_library_mapped_against_contaminants_unmapped_reads.bam

**1.2 Genome assemblies**

**1.2.1 *Riftia* genome assembly: canu**

- canu corMhapSensitivity=normal corOutCoverage=200 correctedErrorRate=0.105 “batOptions=-dg 3 -db 3 -dr 1 -ca 500 -cp 50” genomeSize=700m -pacbio-raw PacBio_libraries.fastq.gz

**1.2.2 *Riftia g*enome assembly: flye**

- flye -g 772m --debug --pacbio-raw PacBio_libraries.fastq.gz -o Rpa-flye

**1.3 Genome post-processing**

**1.3.1 Creating pbmm2 reference databases with genome assemblies**

- pbmm2 index assembly_result.fasta assembly_result.mmi

**1.3.2 Alignment of PacBio native data against draft genomes with pbmm2**

- pbmm2 align --log-level DEBUG --sort assembly_result.mmi list_of_PacBio_bam_files.fofn assembly_result_mapped_against_PacBio_native.bam

**1.3.3 Polishing and variant calling with arrow**

- arrow assembly_result_mapped_against_PacBio_native.bam -r assembly_result.fasta -o assembly_result_arrow.fastq -o assembly_result_arrow.fasta -o assembly_result_arrow.gff3 --annotateGFF --algorithm arrow --log-level INFO --reportEffectiveCoverage

**1.3.4 Purging haplotigs and contig overlaps with purge_dups: minimap aligment and read, base-level read depth calculations**

- for i in list_of_PacBio_databases.txt; do minimap2 -xmap-pb assembly_result_arrow.fasta $i | gzip -c - > $i.paf.gz; done
- pbcstat *.paf.gz
- calcuts -l 15 -u 130 -m 50 PB.stat > cutoffs 2>calcults.log

**1.3.5 Purging haplotigs and contig overlaps with purge_dups: self-alignment**

- split_fa assembly_result_arrow.fasta > assembly_result_arrow.fasta.split
- minimap2 -xasm5 -DP assembly_result_arrow.fasta.split assembly_result_arrow.fasta.split | gzip -c - > assembly_result_arrow.fasta.split.self.paf.gz

**1.3.6 Purging haplotigs and contig overlaps with purge_dups: purge haplotigs and overlaps**

- purge_dups -2 -T cutoffs -c PB.base.cov assembly_result_arrow.fasta.split.self.paf.gz > dups.bed 2> purge_dups.log

**1.3.7 Purging haplotigs and contig overlaps with purge_dups: get purged haplotig sequences**

- get_seqs dups.bed assembly_result_arrow.fasta > assembly_result_arrow_purged.fasta 2> assembly_result_arrow_haplotigs.fasta

**1.3.8 Contamination screening with blobtools: generating coverage file with minimap2**

- minimap2 -ax map-pb assembly_result_arrow_purged.fasta PacBio_libraries_no_contamination.fastq.gz | samtools sort -o assembly_result_arrow_mapped.bam

**1.3.9 Contamination screening with blobtools: generating hit file**

- blastn -max_target_seqs 10 -max_hsps 1 -query assembly_result_arrow_purged.fasta -outfmt '6 qseqid staxids bitscore std' -db nt -evalue 1e-25 -out hit_file_tabular.txt

**1.3.10 Contamination screening with blobtools: generating blobplot**

- blobtools create -i assembly_result_arrow_purged.fasta -b assembly_result_arrow_mapped.bam -t hit_file_tabular.txt -o blobplot --taxrule “bestsumorder”

**1.3.11 Contamination screening: diamond searches against suspicious scaffold**

- diamond blastp -d nr.dmnd --outfmt 0 -q list_of_genes_tig00019702.fasta --evalue 1e-10 --verbose --out tig00019702_blastp_vs_diamond_1e10.txt
- diamond blastp -d nr.dmnd --outfmt 0 -q list_of_genes_tig00002255.fasta --evalue 1e-10 --verbose --out tig00002255_blastp_vs_diamond_1e10.txt

**1.3.12 Contamination screening: identifying bacterial gene markers with checkm**

- checkm lineage_wf -x fasta -g -t 10 ./ output/ >checkM.log

**2.1 Mitochondrial genome assembly and annotation**

**2.1.1 Flye assembly**

- flye --genome-size 15k --pacbio-raw reads_that_mapped_against_mit_ref.fastq.gz -o RIFPA_mitochondrion

**2.1.2 Coverage plot with ConcatMap: minimap2**

- minimap2 -t 12 -ax map-pb -N 5 --secondary=no Riftia_pachyptila_mito.fasta m54098_190810_mapped_mito.fastq.gz > Riftia_pachyptila_mito_vs_reads.sam

**2.1.3 Coverage plot with ConcatMap: ConcatMap**

- ConcatMap_v1.2.py -m 500 –input_file Riftia_pachyptila_mito_vs_reads.sam --reference_length 15406 --figure_format "svg" -s 15 -l 0.002

**2.1.4 Circular plot with circos**

- circos circos.conf

**2.1.5 Synteny analysis with Sibelia**

- Sibelia -s far -o synteny_Riftia_mitochondria –gff all_mitochondria_refs.fasta

**2 TRANSCRIPTOME PIPELINE**

**2.1 Quality assessment of the raw transcriptome databases**

- fastqc transcriptome_1.fastq.gz transcriptome_2.fastq.gz

**2.2 Adapter removal and quality trimming**

**2.2.1 Adapter removal**

- bbduk ktrim=r ziplevel=9 ref=bbmap/resources/adapters.fa in=transcriptome_#.fastq.gz out=transcriptome_#_no_adapters.fastq.gz k=23 mink=11 hdist=1 tpe tbo

**2.2.2 Quality trimming**

- bbduk ziplevel=9 qtrim=rl trimq=30 minlength=30 in=transcriptome_#_no_adapters.fastq.gz out=transcriptome_#_no_adapters_filtered_trimmed.fastq.gz

**2.3 Transcriptome assembly**

**2.3.1 *De novo* transabyss assembly**

- transabyss -SS -pe transcriptome_1_no_adapters_filtered_trimmed.fastq.gz transcriptome_2_no_adapters_filtered_trimmed.fastq.gz --length 200 --cleanup 0

**2.3.2 Reference-based assembly: Generating genome database with STAR**

- STAR --runMode genomeGenerate --genomeDir ./ --genomeFastaFiles Riftia_genome_hard_masked.fasta --genomeSAindexNbases 13

**2.3.3 Reference-based assembly: RNA-seq alignment with STAR**

- STAR --runMode alignReads --outFilterScoreMinOverLread 0.1 --outFilterMatchNminOverLread 0.1 --genomeDir 001-Database/ --readFilesIn transcriptome_1_no_adapters_filtered_trimmed.fastq transcriptome_2_no_adapters_filtered_trimmed.fastq --outFileNamePrefix transcriptome_STAR_mapped_outFilter0.1 --outSAMtype BAM Unsorted --outBAMcompression 10

**2.3.4 Reference-based assembly: Sorting BAM files with samtools**

- samtools sort transcriptome_STAR_mapped_outFilter0.1-Aligned.out.bam

**2.3.5 Reference-based assembly: Indexing sorted BAM files with samtools**

- samtools index transcriptome_STAR_mapped_outFilter0.1-Aligned.out.bam

**2.3.6 Reference-based assembly: Assembling with Stringtie**

- stringtie transcriptome_STAR_mapped_outFilter0.1-Aligned.out.bam.sorted --conservative --rf -m 150 -o transcriptome.gtf -A transcriptome_gene_abundances.tab -l RPA

**2.3.7 Reference-based assembly: Merging Stringtie assemblies**

- stringtie --merge -m 150 -l RPA_merged -o Riftia_pachyptila_STAR_transcriptomes.gtf list_of_stringtie_gtf_files_to_merge.txt

**2.4 *De novo* transcriptome post-processing**

**2.4.1 Removal of endosymbiont contamination: creating BLAST database**

- makeblastdb -dbtype nucl -hash_index -parse_seqids -in Riftia_masked_plus_Endoriftia_db.fasta

**2.4.2 Removal of endosymbiont contamination: running blastn**

- blastn -evalue 1e-05 -num_descriptions 5 -num_alignments 5 -perc_identity 90 -db Riftia_masked_plus_Endoriftia_db.fasta -query transabyss_transcriptome.fasta

**2.4.3 Generating a *de-novo* global non-redundant transcriptome: creating Bowtie database**

- bowtie2-build transcriptome_transabyss.fasta transcriptome_transabyss.fasta

**2.4.4 Generating a *de-novo* global non-redundant transcriptome: multi-mapping of RNA-reads with Bowtie**

- bowtie2 -k 40 --no-unal -1 transcriptome_1_no_adapters_filtered_trimmed.fastq.gz -2 transcriptome_2_no_adapters_filtered_trimmed.fastq.gz -x transcriptome_transabyss.fasta | samtools sort -o transcriptome_mapped.bam

**2.4.5 Generating a *de-novo* global non-redundant transcriptome: clustering transcripts into genes with Corset**

- corset all_bam_files_generated_by_bowtie2.bam

**2.4.6 Generating a *de-novo* global non-redundant transcriptome: building the global *de novo* non-redundant “SuperTranscriptome” with Lace**

- Lace.py –outputDir lace_results_from_transcriptome transcriptome_transabyss.fasta transcriptome_clusterFile_generated_by_corset.txt

**2.5 Prediction of the coding sequence regions with TransDecoder and Stringtie results**

**2.5.1 Retrieving complete genes from Stringtie GTF files**

- TransDecoder-v5.5.0/util/gtf_genome_to_cdna_fasta.pl Riftia_pachyptila_transcriptomes.gtf Riftia_genome_hard_masked.fasta >Riftia_pachyptila_transcriptome_transdecoder.fasta

**2.5.2 Converting Stringtie GTF to GFF3 file**

- TransDecoder-v5.5.0/util/gtf_to_alignment_gff3.pl Riftia_pachyptila_transcriptomes.gtf >Riftia_pachyptila_transcriptomes_transdecoder.gff3

**2.5.3 Extracting all candidate coding sequence regions from Stringtie global transcriptome**

- TransDecoder.LongOrfs -t Riftia_pachyptila_transcriptomes_transdecoder.fasta -S
- TransDecoder.Predict -t Riftia_pachyptila_transcriptomes_transdecoder.fasta --retain_pfam_hits longest_orfs.pep-vs-pfam.domtblout.txt --retain_blastp_hits longest_orfs.pep-vs-uniprot-blastp.outfmt6.txt --output_dir RPA_transdecoder/

**2.5.4 Generating a genome-based Stringtie TransDecoder annotation file**

- TransDecoder-v5.5.0/util/cdna_alignment_orf_to_genome_orf.pl Riftia_pachyptila_transcriptomes_transdecoder.fasta.transdecoder.gff3 Riftia_pachyptila_transcriptomes_transdecoder.gff3 Riftia_pachyptila_transcriptomes_transdecoder.fasta

**2.5.5 Homology searches with candidate CDS against PFAM-A and SwissProt**

- blastp -query longest_orfs.pep -db uniprot_sprot.fasta -max_target_seqs 1 -outfmt 6 -evalue 1e-06 longest_orfs.pep-vs-uniprot-blastp.outfmt6.txt
- hmmscan -E 1e-06 --domtblout longest_orfs.pep-vs-pfam.domtblout Pfam-A.hmm longest_orfs.pep

**2.6 Prediction of the coding sequence regions with TransDecoder and Lace results**

**2.6.1 Generating a GTF file for Lace SuperTranscriptome: create a paf file**

- minimap2 -cx splice -C5 --secondary=no -uf -t 1 --cs Riftia_genome_hard_masked.fasta Riftia_pachyptila_SuperTranscriptomes.fasta > Riftia_pachyptila_SuperTranscriptomes_vs_Riftia_genome.paf

**2.6.2 Generating a GTF file for Lace SuperTranscriptome: convert paf to bed file**

- paftools.js splice2bed Riftia_pachyptila_SuperTranscriptomes_vs_Riftia_genome.paf > Riftia_pachyptila_SuperTranscriptomes_vs_Riftia_genome.bed

**2.6.3 Generating a GTF file for Lace SuperTranscriptome: convert bed to GTF file with bedToGenePred and genePredToGtf tools**

- bedToGenePred Riftia_pachyptila_SuperTranscriptomes_vs_Riftia_genome.bed /dev/stdout | genePredToGtf file /dev/stdin Riftia_pachyptila_SuperTranscriptomes_vs_Riftia_genome.gtf

**2.6.4 Retrieving complete genes from Lace GTF files**

- TransDecoder-v5.5.0/util/gtf_genome_to_cdna_fasta.pl Riftia_pachyptila_SuperTranscriptomes_vs_Riftia_genome.gtf Riftia_genome_hard_masked.fasta >Riftia_pachyptila_SuperTranscriptomes_transdecoder.fasta

**2.6.5 Converting Lace GTF to GFF3 file**

- TransDecoder-v5.5.0/util/gtf_to_alignment_gff3.pl Riftia_pachyptila_SuperTranscriptomes_vs_Riftia_genome.gtf > Riftia_pachyptila_SuperTranscriptomes_transdecoder.gff3

**2.6.6 Extracting all candidate coding sequence regions from Lace SuperTranscriptome**

- TransDecoder.LongOrfs -t Riftia_pachyptila_SuperTranscriptomes_transdecoder.fasta -S
- TransDecoder.Predict -t Riftia_pachyptila_SuperTranscriptomes_transdecoder.fasta --retain_pfam_hits longest_orfs.pep-vs-pfam.domtblout.txt --retain_blastp_hits longest_orfs.pep-vs-uniprot-blastp.outfmt6.txt --output_dir RPA_Super_transdecoder/

**2.6.7 Generating a genome-based Lace TransDecoder annotation file**

- TransDecoder-v5.5.0/util/cdna_alignment_orf_to_genome_orf.pl Riftia_pachyptila_SuperTranscriptomes_transdecoder.fasta.transdecoder.gff3 Riftia_pachyptila_SuperTranscriptomes_transdecoder.gff3 Riftia_pachyptila_SuperTranscriptomes_transdecoder.fasta

**2.6.8 Homology searches with candidate CDS against PFAM-A and SwissProt**

1. blastp -query longest_orfs.pep -db uniprot_sprot.fasta -max_target_seqs 1 -outfmt 6 -evalue 1e-06 longest_orfs.pep-vs-uniprot-blastp.outfmt6.txt
2. hmmscan -E 1e-06 --domtblout longest_orfs.pep-vs-pfam.domtblout Pfam-A.hmm longest_orfs.pep

**2.7 Quality assessment of the final predicted proteomes with BUSCO**

- busco -i Riftia_pachyptila_SuperTranscriptomes_transdecoder.fasta.transdecoder.pep -l metazoa_odb10 –offline -m protein
- busco -i Riftia_pachyptila_transcriptomes_transdecoder.fasta.transdecoder.pep -l metazoa_odb10 --offline -m protein

**3 ANNOTATION PIPELINE**

**3.1 Identification of the repetitive regions with RepeatModeler/RepeatMasker**

**3.1.1 Generating custom repeat library with RepeatModeler**

- RepeatModeler -genomeSampleSizeMax 560783187 -LTRStruct -database Riftia_pachyptila_genome.fasta

**3.1.2 Hard masking the *Riftia* genome with RepeatMasker**

- RepeatMasker -s -e rmblast -dir . -gff -html -a -lib Riftia_pachtyptila_repetitive_db.fasta Riftia_pachyptila_genome.fasta

**3.1.3 Soft masking the *Riftia* genome with RepeatMasker**

- RepeatMasker -s -e rmblast -dir . -gff -html -a -lib Riftia_pachtyptila_repetitive_db.fasta -xsmall Riftia_pachyptila_genome.fasta

**3.1.4 Generation of *Riftia* repeat landscape**

- calcDivergenceFromAlign.pl -s Riftia_div.txt Riftia_pachyptila_genome_masked.fasta.align
- createRepeatLandscape.pl -div Riftia_div.txt -g 560783187

**3.2 *Ab initio* gene prediction with augustus**

**3.2.1 Generating *Riftia pachyptila* intron hints file: merging STAR mapped transcriptomes**

- samtools merge -b all_mapped_star_transcriptomes.fofn -O BAM -l 9 Rpa_merged_transcriptomes_STAR.bam

**3.2.2 Generating *Riftia pachyptila* intron hints file: sorting BAM files by name**

- samtools sort -n Rpa_merged_transcriptomes_STAR.bam -O BAM -o Rpa_merged_transcriptomes_STAR.sortedByName.bam
- samtools sort Rpa_merged_transcriptomes_STAR.sortedByName.filtered.bam -o Rpa_merged_transcriptomes_STAR.sortedByName.filtered.sorted.bam

**3.2.3 Generating *Riftia pachyptila* intron hints file: filtering aligned reads from BAM**

- filterBam --uniq --paired --pairwiseAlignments --in Rpa_merged_transcriptomes_STAR.sortedByName.bam --out Rpa_merged_transcriptomes_STAR.sortedByName.filtered.bam

**3.2.4 Generating *Riftia pachyptila* intron hints file: extracting intron information and removing inappropriate splice site information from BAM file**

- bam2hints --intronsonly --in=Rpa_merged_transcriptomes_STAR.sortedByName.filtered.bam --out=Riftia_introns_hints.gff
- filterIntronsFindStrand.pl Riftia_genome_soft_masked.fasta Riftia_introns_hints.gff --score > Riftia_introns_filtered_hints.gff

**3.2.5 Generating training set gene structure: running unsupervised GeneMark-ET**

- gmes_petap.pl --verbose –sequence=Riftia_genome_soft_masked.fasta --ET=Riftia_introns_filtered_hints.gff --soft_mask 1000 --max_intron 349151 --max_contig 13119000 --min_contig 2273

**3.2.6 Generating training set gene structure: filtering GeneMark-ET predictions**

- filterGenemark.pl genemark.gtf Riftia_introns_filtered_hints.gff

**3.2.7 Generating training set gene structure: computing flanking region size of the genes**

- computeFlankingRegion.pl genemark.f.good.gtf

**3.2.8 Generating training set gene structure: converting GeneMark structures to GenBank**

- gff2gbSmallDNA.pl genemark.f.good.gtf Riftia_genome_hard_masked.fasta 124 tmp.gb

**3.2.9 Generating training set gene structure: filtering good gene models from bonafide.gtf**

- filterGenesIn_mRNAname.pl genemark.gtf tmp.gb > GeneMark_models_filtered.gb

**3.2.10 Generating *Riftia pachyptila* exonpart hints from RNA-seq data**

- bam2wig Rpa_merged_transcriptomes_STAR.sortedByName.filtered.bam > RNASeq_exonpart_Rpa_merged_transcriptomes_STAR.wig
- cat RNASeq_exonpart_Rpa_merged_transcriptomes_STAR.wig | wig2hints.pl --width=10 --margin=10 --minthresh=2 --minscore=4 --prune=0.1 --src=W --type=ep -UCSC=unstranded.track --radius=4.5 --pri=4 --strand=‘‘.’’ > Riftia_exonpart_hints.gff

**3.2.11 Generating *Riftia pachyptila* exonpart, intron, acceptor (3’) splice site, donor splice site (5’) and exon hints file from Cdna data: running blat**

- blat -noHead -mask=lower soft -minIdentity=92 Riftia_genome_soft_masked.fasta Riftia_pachyptila_non_redundant_de_novo_reference_based_cDNA_from_transdecoder.fasta cdna_vs_Riftia_genome_soft_masked.psl

**3.2.12 Generating *Riftia pachyptila* exonpart, intron, acceptor (3’) splice site, donor splice site (5’) and exon hints file from Cdna data: filtering and sorting blat alignments**

- pslCDnaFilter -ignoreNs -localNearBest=0.005 -minId=0.9 -bestOverlap cdna_vs_Riftia_genome_soft_masked.psl > cdna_vs_Riftia_genome_soft_masked.filtered.psl
- cat cdna_vs_Riftia_genomes_soft_masked.filtered.psl |sort -n -k 16,16 |sort -s -k 14,14 > cdna_vs_Riftia_genomes_soft_masked.filtered.sorted.psl

**3.2.13 Generating *Riftia pachyptila* exonpart, intron, acceptor (3’) splice site, donor splice site (5’) and exon hints file from Cdna data: converting psl file to hints format**

- blat2hints.pl --in=cdna_vs_Riftia_genomes_soft_masked.filtered.sorted.psl --minintronlen=31 --maxintronlen=349151 --trunkSS --ssOn --remove_redundant --out=Riftia_cDNA_hints.gff

**3.2.14 Generating *Riftia pachyptila* protein hints file from protein data: running genomeThreader**

- startAlign.pl --prg=gth --prot=Riftia_pachyptila_denovo_plus_reference_based_transcriptomes_clustered90.pep --genome= Riftia_genome_hard_masked.fasta

**3.2.15 Generating *Riftia pachyptila* protein hints file from protein data: converting alignment output to protein hints file:**

- align2hints.pl --minintronlen=31 --maxintronlen=349151 --in=gth.concat.aln --out=Riftia_protein_hints.gff --prg=gth

**3.2.16 Training augustus for *Riftia pachyptila*: creating a template for a new species**

- new_species.pl --species=riftia_pachyptila
- randomSplit.pl Riftia_pachyptila_gene_models_filtered.gb 200
- etraining –species=riftia_pachyptila Riftia_pachyptila_gene_models_filtered.gb.train > etrain.out

**3.2.17 Training augustus for *Riftia pachyptila*: meta parameter optimisation**

- optimize_augustus.pl –species=riftia_pachyptila Riftia_pachyptila_gene_models_filtered.gb.train
- optimize_augustus.pl –species=riftia_pachyptila Riftia_pachyptila_UTR_gene_models_filtered.gb.train --UTR=on --metapars=Augustus/config/species/riftia_pachyptila/riftia_pachyptila_metapars.utr.cfg --trainOnlyUtr=1

**3.2.18 Running *Riftia pachyptila* gene prediction with augustus**

- augustus --species=riftia_pachyptila --UTR=no --softmasking=on Riftia_soft_masked_genome.fasta –min_intron_len=31 > Riftia_pachyptila_no_hints_predictions.gtf
- augustus --species=riftia_pachyptila Riftia_soft_masked_genome.fasta --extrinsicCfgFile=extrinsic.M.RM.E.W.P.cfg --hintsfile=Riftia_pachtypila_hints_no_EP.gff --allow_hinted_splicesites=atac --alternatives-from-evidence=on --UTR=no --softmasking=on --min_intron_len=31 > Riftia_pachyptila_hints_no_UTR_predictions.gtf
- augustus –species=riftia_pachyptila Riftia_soft_masked_genome.fasta --extrinsicCfgFile=extrinsic.M.RM.E.W.P.cfg –hintsfile=Riftia_pachtypila_hints.gff --allow_hinted_splicesites=atac --alternatives-from-evidence=on --UTR=yes --softmasking=on --min_intron_len=31 > Riftia_pachyptila_hints_UTR_predictions.gtf

**3.3 Augustus post-processing results**

**3.3.1 Joining gtf annotations**

- joingenes --alternatives --genesets=Riftia_pachyptila_no_hints_predictions.gtf,Riftia_pachyptila_hints_no_UTR_predictions.gtf,Riftia_pachyptila_hints_UTR_predictions.gtf –priorities=1,2,3 --output=Riftia_pachyptila_predictions_combined.gtf

**3.3.2 Extracting coding sequence regions in amino acid and nucleotide formats**

- getAnnoFastaFromJoingenes.py -g Riftia_pachyptila_soft_masked.genome --filter_out_invalid_stops=yes -f Riftia_pachyptila_predictions_combined.gtf -o Riftia_pachyptila_predictions_combined

**3.3.3 Removing non supported gene models: PFAM searches**

- hmmsearch --cpu 12 --domtblout Riftia_pachyptila_predictions_combined_vs_PFAM-1e10_tabular.txt -E 1e-10 Pfam-A.hmm Riftia_pachyptila_predictions_combined.aa

**3.3.4 Removing non supported gene models: diamond blastp searches**

- diamond blastp --db nr.dmnd --outfmt 6 --query Riftia_pachyptila_predictions_combined.aa --out diamond_augustus_predicted_vs_nr_1e10.txt --evalue 1e-10 --max-target-seqs 1

3.3.5 Removing non supported gene models: orthofinder orthology inferences

- orthofinder -M msa -t 12 -T iqtree -f directory_with_protein_files/

**3.3.6 Removing non supported gene models: gene expression evidence**

- kallisto index -i Riftia_pachyptila_predictions_combined.codingseq.idx Riftia_pachyptila_predictions_combined.codingseq
- kallisto quant –bias -i Riftia_pachyptila_predictions_combined.codingseq.idx --rf-stranded -b 100 -o ./ Trimmed_filtered_transcriptomes_1.fastq.gz Trimmed_filtered_transcriptomes_2.fastq.gz

**3.4 Protein annotation**

**3.4.1 Interproscan**

- interproscan --iprlookup --goterms -i Riftia_pachyptila_predictions_combined_filtered.aa -f TSV,HTML,GFF3,XML --disable-precalc --pathways -d interpro_results

**3.4.2 signalp**

- signalp-v5 -batch 20000 -fasta Riftia_pachyptila_predictions_combined_filtered.aa -format long -gff3 -mature

**3.4.3 tRNA-scan**

- tRNAscan-SE -HQ -o# -f# -m# -s# -a# --detail -p Riftia_pachyptila_hard_masked Riftia_pachyptila_hard_masked.fasta

**3.4.4 Individual gene identification and phylogeny: Antennapedia cluster-, Wnt ligands/receptors-, apoptosis-, hemoglobin-related, immune-related genes**

- blastp -db tubeworms_proteins.fasta -query metazoan_queries.fasta -outfmt 6 -max_target_seqs 10 > tubeworms_candidates_10best_tabular.txt
- for i in `cat tubeworms_candidates_10best_tabular.txt|sort -u`; do blastdbcmd -entry $i -db tubeworms_proteins.fasta >> tubeworms_candidates_10best_tabular.fasta; done;
- cat tubeworms_candidates_10best_tabular.fasta metazoan_homologues.fasta > tubeworms_candidates_plus_metazoa.fasta
- mafft --maxiterate 1000 --localpair tubeworms_candidates_plus_metazoa.fasta > tubeworms_candidates_plus_metazoa.fasta.aln
- iqtree -nt AUTO -s tubeworm_best_candidates_plus_metazoa_trimmed.fasta.aln -alrt 1000 -bb 1000

**3.4.5** [**Transcription factor families (TFFs) identification**](#__RefHeading___Toc2566_556504746)

- pfam_scan.pl -e_seq 1e-05 -e_dom 1e-05 -fasta proteome.fasta -dir PFAM-A/ -outfile output.txt

**4 GENE FAMILY ANALYSIS**

**4.1 Adding unique identifier and removing redundancy with gff3 files coordinates**

- sed -i 's/>/>ID_/' metazoan_db.fasta
- pymakeMap.py -gff metazoan.gff3 -p ID -f CDS -d -k protein_id -o metazoan.chrom

**4.2 Orthofinder**

- orthofinder -f metazoan_db_directory/
- orthofinder -f lophotrochozoan_db_directory/
- orthofinder -f annelida_db_directory/

**4.3 Synonymous and non-synonymous substitution rates and positive selection analysis**

**4.3.1 KaKs_calculator**

- for i in `cat list_of_OGs.txt`; do mafft --maxiterate 1000 --localpair $i\_AA.fasta.aln > $i\_AA.fasta.aln; done;
- for i in `cat list_of_OGs.txt`; do Epal2nal.pl $i\_AA.fasta.aln $i\_CDS.fasta -output axt > $i\_CDS.fasta.axt; done;
- for i in `cat list_of_OGs.txt`; do KaKs_calculator -i $i\_CDS.fasta.axt -o $i\_CDS.fasta.axt.kaks;

**4.3.2 Hyphy aBSREL**

- for i in `cat list_of_OGs.txt`; do pal2nal.pl $i\_AA.fasta.aln $i\_CDS.fasta -output fasta > $i\_CDS.fasta.aln; done;
- for i in `cat list_of_OG.txt`; do iqtree -st CODON -s $i\_CDS_fixed.fasta.aln -m TESTONLY -nt AUTO; rm $i\_CDS_fixed.fasta.aln.iqtree; rm $i\_CDS_fixed.fasta.aln.mldist; rm $i\_CDS_fixed.fasta.aln.log; rm $i\_CDS_fixed.fasta.aln.ckp.gz; rm $i\_CDS_fixed.fasta.aln.model.gz; rm $i\_CDS_fixed.fasta.aln.bionj; done;
- for i in `cat list_of_OG.txt`; do perl -p -e "s/(RIFPA_Rpa_jg\d+.t\d+)/\$1\{tubeworm\}/" $i\_CDS_fixed.fasta.aln.treefile.renamed > $i\_CDS_fixed.fasta.aln.treefile.renamed.branch.label; done;
- for i in `cat list_of_OGs.txt`; do hyphy aBSREL --alignment $i\_CDS_fixed.fasta.aln --tree \ $i\_CDS_fixed.fasta.aln.treefile.renamed --branches tubeworm
- for i in `cat list_of_OGs_tubeworm_positive_selected.txt`; do hyphy aBSREL --alignment $i\_CDS_fixed.fasta.aln --tree \$i\_CDS_fixed.fasta.aln.treefile --branches All

**4.3.4 Annotation of positive selected genes: pantherScore2.0.pl**

- pantherScore2.2.pl -l PANTHER14.1/ -D B -V -i positive_selected_genes_lophotrochozoa.fasta -o output.panther -n -s
- pantherScore2.2.pl -l PANTHER14.1/ -D B -V -i positive_selected_genes_annelida.fasta -o output.panther -n -s

**4.4 Phylogenomics**

**4.4.1 Unifying header names across orthogroups**

- for i in `ls -1 *fa`; do perl -pe 's/(>\S{5}).*/$1/' $i > $i.renamed.fasta; done;

**4.4.2 Multiple sequencing alignments**

- for i in `cat list.txt`; do mafft --maxiterate 1000 --localpair $i.renamed.fasta > $i.renamed.fasta.aln; done;

**4.4.3 FASconCAT and multiple sequencing alignment trimming**

- FASconCAT-G_v1.04.pl
- BMGE.jar -i Lophotrochozoa_supermatrix.fasta -m BLOSUM30 -h 1 -b 1 -t AA -of Lophotrochozoa_supermatrix.trimmed.fasta

**4.4.4 Bayesian inference: generating Lophotrochozoa starting tree and phylogenetic run**

- FastTree Lophotrochozoa_supermatrix.trimmed.fasta > Lophotrochozoa.tree
- pb -d Lophotrochozoa_supermatrix.trimmed.phy -T Lophotrochozoa.tree -r outgroup.txt -ln -cal calibrations.txt -gtr -cat -rp 600 0 -prior lophotrochozoa_run
- readdiv -x 7916 100 lophotrochozoa_run

**4.5 Expanded/contracted gene families**

**4.5.1. CAFE analysis: preparing orthogroups for CAFE**

- python cafetutorial_clade_and_size_filter.py -i Orthogroups.GeneCount.tsv -o Orthogroups.GeneCount.filtered.tsv -s
- ./cafe_filtered_families.sh
- ./cafe_large_families.sh
- ./cafe_filtered_families_lambda.sh
- ./caferror.sh
- ./cafe_filtered_families_error_many_lambdas.sh
- cafetutorial_report_analysis.py -r 0 -i report_file.cafe -l large_families.cafe -o output_report

**4.5.2 Annotation of the rapidly evolving gene families: pantherScore2.0.pl**

- pantherScore2.2.pl -l PANTHER14.1/ -D B -V -i rapidly_evolving_gene_families.fasta -o output.panther -n -s

**4.6 Clustering of *Riftia pachyptila* expanded PFAM-domain containing genes**

- clans -in PFAM_domain_containing_genes.fasta -eval 1e-5 -cpu 10 -blastpath "blastp"

**4.6 Global view of *Riftia pachyptila* proneuropeptide and prohormone complement**

- signalp -fasta Riftia_pachyptila_proteins.fasta -gff3 -mature
- hmmsearch -E 1e-10 –domtblout Riftia_pachyptila_mature_with_cleavage_vs_PFAM-A.txt Pfam-A.hmm Riftia_pachyptila_proteins_mature_with_cleavage_sites.fasta
- clans -in Riftia_general_cluster.fasta -eval 1e-10 -blastpath psiblast
- clans -in Riftia_central_cluster.fasta -eval 1e-10 -blastpath blastp

**5 GENE EXPRESSION QUANTIFICATION**

**5.1 Kallisto: creating index**

- kallisto index -i Riftia_pachyptila_predictions_combined.codingseq.idx Riftia_pachyptila_predictions_combined.codingseq

**5.2 Kallisto: quantification**

- for i in `cat list_of_trimmed_filtered_transcriptomes.txt`; do kallisto quant --bias -i Riftia_pachyptila_predictions_combined.codingseq.idx --rf-stranded –plaintext -b 100 -o ./ $i\_trimmed_filtered_1.fastq.gz $i\_trimmed_filtered_2.fastq.gz

**5.3 Tissue specificity quantification: tau values**

- Rscript tau_female_tissues.R

**5.4 GO enrichment analyses**

- Rscript goTerms_female_tissues.R

**6 ADDITIONAL COMMAND LINE APPLICATIONS USED**

**6.1 Quality assessment with quast**

- quast.py -m 1 file1.fasta file2.fasta

**6.2 Generating Cdna, cds and protein datasets for augustus**

**6.2.1 Combining transdecoder predicted protein files from *de novo* and reference-based approaches**

- cat predicted_proteins_transdecoder_from_de_novo_assemblies.pep predicted_proteins_transdecoder_from_reference_based_assemblies.pep > combined_proteins_from_de_novo_reference_based_assemblies_transdecoder.pep

**6.2.2 Removing redundancy from combined protein dataset**

- cd-hit -i combined_proteins_from_de_novo_reference_based_assemblies_transdecoder.pep -o Riftia_pachyptila_denovo_plus_reference_based_transcriptomes_clustered90.pep -c 0.9

**6.2.3 Retrieving matching cDNA fasta files: creating blast database cDNA files**

- makeblastdb -dbtype nucl -hash_index -parse_seqids -in Riftia_pachyptila_denovo_plus_reference_based_transcriptomes_cDNA.fasta

**6.2.3 Retrieving matching cDNA fasta files: retrieving sequences with blastdbcmd**

- blastdbcmd -entry_batch list_of_fasta_headers_to_retrieve.txt -db Riftia_pachyptila_denovo_plus_reference_based_transcriptomes_cDNA.fasta > Riftia_pachyptila_non_redundant_de_novo_reference_based_cDNA_from_transdecoder.fasta

**6.3 Removing redundant gene structures from augustus training sets**

**6.3.1 Converting training gene structure gtf file to a protein sequences**

- gtf2aa.pl Riftia_genome_hard_masked.fasta training_genes.gtf proteins.fasta

**6.3.2 Removing redundancy from training gene amino acid sequences**

- aa2nonred.pl proteins.fasta proteins.nr.fasta

**6.3.3 Filtering and retrieving non-redundant loci**

- diamond blastp nr.dmnd --outfmt 6 --query proteins.nr.fasta --out nr-proteins-vs-diamond-1e10.full.txt --evalue 1e-10
- cut -f1 nr-proteins-vs-diamond-1e10.full.txt |sort -u > nonred.lst
- cat training_genes.gb | perl -ne 'if ($_ =~ m/LOCUS\s+(\S+)\s/) {$txLocus = $1;} elsif ($_ = m/\/gene=\"(\S+)\"/) { $txInGb3{$1} = $txLocus } if(eof()) { foreach (keys %txInGb3) { print "$_\t$txInGb3{$_}\n"; } }' > loci.lst
- grep -f nonred.lst loci.lst | cut -f2 > nonred.loci.lst
- filterGenesIn.pl nonred.loci.lst training_genes.gb > training_genes_filtered.gb
